# The *Trichoderma harzianum *demon: complex speciation history resulting in coexistence of hypothetical biological species, recent agamospecies and numerous relict lineages

**DOI:** 10.1186/1471-2148-10-94

**Published:** 2010-04-01

**Authors:** Irina S Druzhinina, Christian P Kubicek, Monika Komoń-Zelazowska, Temesgen Belayneh Mulaw, John Bissett

**Affiliations:** 1 Research Area of Gene Technology and Applied Biochemistry, Institute of Chemical Engineering, Vienna University of Technology, Getreidemarkt 9-1665, A-1060 Vienna, Austria; 2 Agriculture and Agri-Food Canada, Eastern Cereal and Oilseed Research Center, Central Experimental Farm, Ottawa, Ontario K1A 0C6, Canada

## Abstract

**Background:**

The mitosporic fungus *Trichoderma harzianum *(*Hypocrea*, Ascomycota, Hypocreales, Hypocreaceae) is an ubiquitous species in the environment with some strains commercially exploited for the biological control of plant pathogenic fungi. Although *T. harzianum *is asexual (or anamorphic), its sexual stage (or teleomorph) has been described as *Hypocrea lixii*. Since recombination would be an important issue for the efficacy of an agent of the biological control in the field, we investigated the phylogenetic structure of the species.

**Results:**

Using DNA sequence data from three unlinked loci for each of 93 strains collected worldwide, we detected a complex speciation process revealing overlapping reproductively isolated biological species, recent agamospecies and numerous relict lineages with unresolved phylogenetic positions. Genealogical concordance and recombination analyses confirm the existence of two genetically isolated agamospecies including *T. harzianum *sensu stricto and two hypothetical holomorphic species related to but different from *H. lixii*. The exact phylogenetic position of the majority of strains was not resolved and therefore attributed to a diverse network of recombining strains conventionally called 'pseudoharzianum matrix'. Since *H. lixii *and *T. harzianum *are evidently genetically isolated, the anamorph - teleomorph combination comprising *H. lixii/T. harzianum *in one holomorph must be rejected in favor of two separate species.

**Conclusions:**

Our data illustrate a complex speciation within *H. lixii *- *T. harzianum *species group, which is based on coexistence and interaction of organisms with different evolutionary histories and on the absence of strict genetic borders between them.

## Background

The unique nature of fungi, when the closely related organisms exploit incomparably different strategies for reproduction (mostly sexual vs. exclusively asexual vs. sexual and asexual), leads to existence of a variety of overlapping species concepts. Some fungal species can be differentiated based on sexual compatibility of their members, which are in turn reproductively isolated from the other species (i.e. the biological species concept). The situation is more complicated with those fungi which either do not mate in vitro or have lost the ability to reproduce sexually in nature. In addition, the taxonomy of almost every large group of fungi suffers from certain historical biases which usually come from the applied value of the organisms. The introduction of molecular methods in evolutionary mycology has already resulted in the dramatic taxonomic changes [1] though the effort is still needed to rich the clarity for individual genera. The mitosporic genus *Trichoderma *(*Hypocrea*, Ascomycota, Hypocreales, Hypocreaceae), a fungus beneficially used in agriculture, is a striking example for this: the number of its morphologically recognized species (i.e. the morphological species concept) is still around 30 [2-4], while the application of genealogical concordance between unlinked DNA loci (i.e. the phylogenetic species concept) resulted in 100 phylogenetic species recognized by 2006 [5] and this number is growing quickly.

The special scientific interest to this genus is largely connected with the modern pace towards the development of the market for organic farm products, which already covers approximately 2% of total world farmland and more than 10% in some European countries such as Austria, Switzerland and Sweden [6]. The management of organic farms requires the integration of biological pest control (*bio*control) in agricultural practices. The control or prevention of some plant diseases by such mycoparasitic fungi or '*bio*fungicides' such as *Trichoderma *is an attractive alternative to the use of chemical fungicides [7] and therefore is an important component of modern organic farming. These fungi not only antagonize plant pathogens [8,9], but are also rhizosphere competent and can enhance plant growth in endophyte-like associations [10]. However, as the prerequisite to applying biofungicides to farm fields, the biology of every active agent should be understood. Some molecular aspects of *Trichoderma *mycoparasitism and endophytism - such as the role and regulation of formation of cell wall hydrolytic enzymes and antagonistic secondary metabolites - have been intensively investigated [8]. On the other hand, the genetic stability of the fungus in the environment, its population structure, reproduction strategies and geographic distribution, have received little attention and remain poorly studied.

The asexual (or anamorphic) *T. harzianum *is the most familiar *Trichoderma *species as it is the most frequent *Trichoderma *sp. in the majority of samples worldwide [11-13]. It has often even been synonymzed with *Trichoderma *biocontrol agents in general [14], as it is the principal component in several commercial biofungicide formulations. Although the biology of *T. harzianum *has not been studied in detail, it was studied taxonomically. It was originally defined as a "species aggregate" [15], but Chaverri *et al*. [16] reported that it consists of seven genetic lineages which would fulfil the basic criteria of cryptic phylogenetic species within a large morphological species. The latter authors also stated that *H. lixii *is a teleomorph (sexual stage) of *T. harzianum *thus raising the probability of genetic recombination in nature. Despite the detected genetic polymorphism, the authors proposed the existence of a single although complex *H. lixii/T harzianum *species. However, no more definitive data have been published on the reproduction, global geographic distribution and speciation within the *H. lixii/T. harzianum*.

In this paper we reveal the complex speciation history within *H. lixii/T. harzianum *sensu Chaverri *et al*. [16] and show that it consists of several hypothetical biological species, some recent agamospecies and numerous relict lineages with a monophyletic origin altogether. We also show that *T. harzianum *sensu stricto and *H. lixii *s.s. are genetically isolated and therefore are two separate species.

## Results

### Sample set

**Figure 1:**
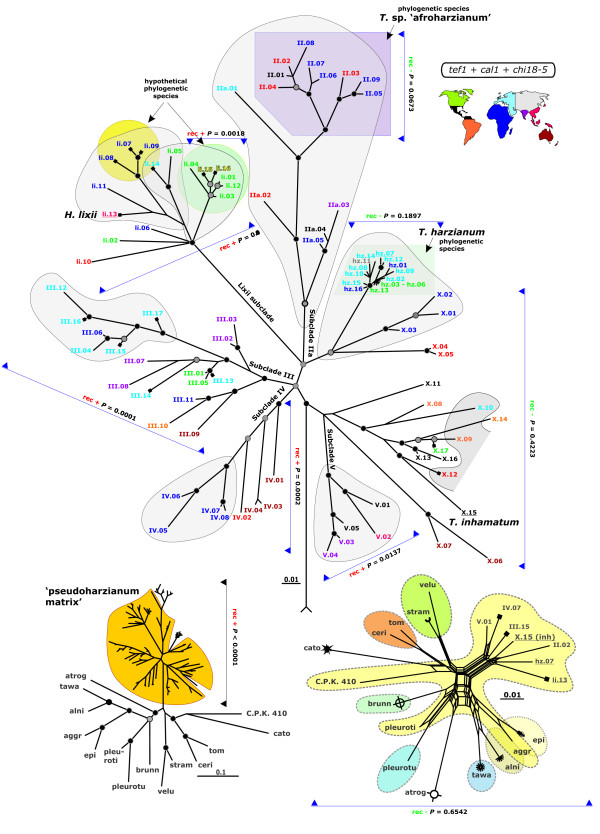
**Multilocus phylogenetic map of *H. lixii - T. harzianum *species complex**. The main body of the figure shows the radial Bayesian tree resulting from the analysis of the concatenated sequences of *tef1*, *cal1 *and *chi18-5*. Nodes supported by posterior probabilities (PP) > 0.94 and 0.89 < PP < 0.95 are indicated by black and grey dots respectively. Names of subclades, as used in the text, are written along the most internal branches leading to them. Full squares at OTUs indicate teleomorph isolates. The color code indicates the geographic region from which the isolates were obtained, as explained in the right top inset. Isolates with yellow color indicate uncertain geographic origin. The *ex*-type strains are underlined. The four putative phylogenetic species are indicated by rectangular and round backgrounds for agamospecies and holomorphic species, respectively. Isolates for which a recombination history was detected are grouped by irregular shape frames with grey background. Isolates used and results from the PHT test are shown by arrows and the respective P values, 'rec +' specifying positive recombination result and 'rec -'specifying no recombination detected. The left bottom inset shows the results from the same analysis when representative strains of the all studied *H. lixii/T. harzianum *and the representative strains of the Harzianum-Catoptron Clade were also included. The yellow background separates strains of the 'pseudoharzianum matrix'. Other symbols are used as above, but no geographic color code is applied. The right bottom inset shows the recombination network, determined by SplitsTree (NJ mode) from the combined dataset. Taxa with "harzianum"-like morphology are shown by a light yellow background and dotted lines; other anamorph morphologies are shown by different colours and dotted lines; individual teleomorph morphologies are indicated by different end symbols. Representative sequences for species from Harzianum-Catoptron Clade may be retrieved from NCBI Entrez search engine using [species strain locus] keywords.

As a first prerequisite for this study we established a representative sample set, originally comprising > 300 strains of worldwide origin which were identified as *H. lixii/T. harzianum *by the ITS1 and 2 barcode [11] and diagnostic morphophysiological characteristics [15,16]. The sample was condensed to 93 strains which were representative for the total genetic and geographic variation observed. The final sample set included 10 strains which were collected as *Hypocrea *teleomorphs from decaying wood (Table 1), whereas the strains isolated as *Trichoderma *anamorphs were predominantly from various soil ecosystems. Representative strains of all neighbour species (the Harzianum-Catoptron Clade; [17,18]) were included in order to determine the speciation history within the clade, to set up genetic borders for morphological *H. lixii *and *T. harzianum *species, and to be used as a negative control for recombination analyses. The spans of the morphological species (separately considered for teleomorphs and anamorphs) are shown on the right inset in Figure 1. Species names abbreviations presented in Figure 1 correspond to atrog - *H. atrogelatinosa *CBS 237.63, tawa - *H. tawa *CBS 246.63, alni - *H. alni *CBS 120633, aggr - *T. aggressivum *CBS 433.95, epi - *H. epimyces *CBS 120534, pleuroti - *T. pleuroticola *Z.D. 56, pleurotu - *T. pleurotum *C.P.K. 2113, brunn - *H. brunneoviridis *CBS 120928, velu - *T. velutinum *DAOM 320014, stram - *H. straminea *G.J.S. 02-84, ceri - *T. cerinum *DAOM 230012, tom - *T. tomentosum *Z.D. 28, cato - *H. catoptron *G.J.S. 02-76, C.P.K. 410 - *T*. sp. C.P.K. 410.

**Table 1:** Strains of *H. lixii/T. harzianum *species complex used in this study.

					NCBI GenBank Assession No.		
					
	Strain no.	Other collections	Origin	Habitat	*tef1*	*cal1*	*chi18-5*
	T. harzianum*						

hz.01**	C.P.K. 1052	DAOM231646	Kirstenbosch, South Africa	soil	AY605766	FJ577720	EF191257
hz.02	C.P.K. 1070	DAOM 222343	Ireland	commercial mushroom bed	AY605778	FJ577729	EF191265
hz.03	C.P.K. 1087	J.B.T 1244	Almonte, ON, Canada	*Salix *sp. wood	AY605784	FJ577733	EF191269
hz.04	C.P.K. 1093	J.B.T 2181	Labelle, PQ, Canada	dead wood	AY605787	FJ577734	EF191271
hz.05	C.P.K. 1099	DAOM 222183	Leamington, ON, Canada	wood floor of mushroom farm	AY605790	FJ577736	EF191272
hz.06	C.P.K. 1104	DAOM 176235B	Vancouver Island, Canada	on jelly fungus	AY605793	FJ577738	EF191275
hz.07	C.P.K. 204 *ex*-type	CBS 226.95	Sheffield, UK	garden soil	AY605833	FJ577684	AF276646
hz.08	C.P.K. 206	TUB F-477	Ural, Russia	park soil	AY605830	FJ577685	AF399269
hz.09	C.P.K. 2111	SzMC 3203	Hungary	mushroom farm	EF116562	FJ577754	EF191248
hz.10	C.P.K. 217	TUB F-690	Moscow, Russia	park soil	AY605829	FJ577686	AY605873
hz.11	C.P.K. 261	TUB F-743	Krasnoyarsk, Siberia	cultivated soil	AY605827	FJ577691	AF399270
hz.12	C.P.K. 265	TUB F-750	Vladimir, Russia	garden soil	EF113554	FJ577692	FJ623079
hz.13	C.P.K. 1116	DAOM 167088	Kananaskis, AB, Canada	alpine eutric brunisol	EF191324	FJ577742	EF191280
hz.14	C.P.K. 360	IMI 359823	North Ireland, UK	*Agaricus *cultivation	AY605832	FJ577701	AY605883
hz.15	J.B. RO11-1	C.P.K. 2654	Brasov, Romania	dried river bed	EF191337	FJ577767	EF191293
hz.16	C.P.K. 1818	PPRC J12	Jimma, Ethiopia	red soil	EF116558	FJ577750	FJ623101

	**Lixii subclade**						

li.01	C.P.K. 1068	DAOM 229959	Wisconsin, USA	A1 horizon, prairie soil	AY605776	FJ577727	EF191263
li.02	C.P.K. 1081	DAOM 229903	Wisconsin, USA	A1 horizon, maple forest soil	AY605782	FJ577731	EF191267
li.03	C.P.K. 1102	DAOM 222136	Campbellville, ON, Canada	commercial mushroom bed	AY605792	FJ577737	EF191274
li.04	C.P.K. 1107	DAOM 222137	Leamington, ON, Canada	commercial mushroom bed	AY605796	FJ577739	EF191276
li.05	C.P.K. 1108	DAOM 222151	Temple, PA, USA	commercial mushroom bed	AY605797	FJ577740	EF191277
li.06	C.P.K. 1110	DAOM 190830	Kingston, ON, Canada	foam insulation	EF191323	FJ577741	EF191278
**li.07**	**C.P.K. 1720**	**G.J.S. 05-82**	**Cameroon**	**unknown**	**EF191326**	**FJ577747**	**EF191282**
**li.08**	**C.P.K. 1722**	**G.J.S. 05-22**	**Cameroon**	**unknown**	**EF191327**	**FJ577748**	**EF191283**
**li.09**	**C.P.K. 1724**	**G.J.S. 05-32**	**Cameroon**	**unknown**	**EF191328**	**FJ577749**	**EF191284**
li.10	C.P.K. 588		Nam Lenk river, Laos	river bank soil	FJ577778	FJ577703	FJ623084
li.11	C.P.K. 838	CBS 115334	El-Fayum, Egypt	wheat field	AY605837	FJ577715	FJ623092
li.12	C.P.K. 1069	DAOM 229907	Konza Prairie, KS, USA	A1 horizon, tallgrass soil	AY605777	FJ577728	EF191264
**li.13**	**C.P.K. 2784 *ex*-type**	**G.J.S. 97-96**	**Saraburi Prov., Thailand**	**from decayed *Ganoderma *sp.**	**FJ716622**	**FJ577772**	**FJ623107**
**li.14**	**C.P.K. 1596**	**W.M.J. 2317**	**Styria, Austria**	**unknown**	**FJ577785**	**FJ577744**	**FJ623099**
**li.15**	**C.P.K. 334**	**G.J.S. 98-65**	**Unknown**	**unknown**	**FJ577776**	**FJ577699**	**FJ623081**
**li.16**	**C.P.K. 335**	**G.J.S. 98-64**	**Unknown**	**unknown**	**FJ577777**	**FJ577700**	**FJ623082**

	**Subclade II *T*. sp. 'afroharzianum'**						

II.01	C.P.K. 238		Costa Rica	maize field	AY605841	FJ577687	EF191250
II.02	C.P.K. 807	NR5555	Japan	unknown	AY605842	FJ577711	AF399263
II.03	C.P.K. 808	NR6839	Japan	unknown	AY605843	FJ577712	AF399264
II.04	C.P.K. 246	CBS 115344	Phillipines	maize field	EF191319	FJ577690	EF191253
II.05	C.P.K. 845		Banha, Egypt	wheat field	AY605844	FJ577716	FJ623093
II.06	C.P.K. 2618	PPRC RW14	Holleta, Ethiopia	coffe rhizosphere	FJ577788	FJ577758	FJ623103
II.07	C.P.K. 51	PPRI 3772	South Africa	unknown	AY605845	FJ577681	AY605868
II.08	C.P.K. 1061	DAOM 231421	Kigali, Rwanda	clay soil	AY605770	FJ577723	FJ623096
II.09	C.P.K. 2624	PPRC RW20	Harerge, Ethiopia	coffee rhizosphere	FJ716621	FJ577759	FJ623104

	**Subclade II a**						

IIa.01	J.B. SERB24-1	DAOM 233401	Bežanijska Kosa, Serbia	chernozem soil, corn field	EF191339	FJ577769	EF191295
IIa.02	C.P.K. 1095	DAOM 230766	Bali, Indonesia	park soil	AY605788	FJ577735	FJ623097
IIa.03	C.P.K. 274	TUB F-771	Ghaze, Nepal	forest soil	AY605834	FJ577695	AY605880
IIa.04	C.P.K. 245	CBS 115343	Costa Rica	maize field	EF191318	FJ577689	EF191252
IIa.05	C.P.K. 2710	PPRC S23	Dilla, Ethiopia	soil	FJ577790	FJ577771	FJ623106

	**Subclade III**						

III.01	C.P.K. 1075	DAOM 229908	Wisconsin, USA	A1 horizon, forest soil	EF191322	FJ577730	EF191266
III.02	C.P.K. 276	TUB F-773	Nepal	bark	AY605850	FJ577696	AY605881
III.03	C.P.K. 272	TUB F-769	Nepal	bark	AY605849	FJ577694	AY605879
III.04	C.P.K. 2301	UNISS 13b-11	Cuglieri, Sardinia	forest land	EF488114	FJ577755	EF392736
III.05	C.P.K. 2646	J.B. GA3804	Georgia, USA	unknown	EF191329	FJ577760	EF191285
III.06	J.B. RSA122	DAOM 231651	Kirstenbosch, South Africa	soil under *Erica*	EF191338	FJ577768	EF191294
III.07	C.P.K. 271	TUB F-768	Geirigan, Nepal	on *Quercus*	AY605847	FJ577693	AF399267
III.08	C.P.K. 291	TUB F-776	Ghaze, Nepal	bark of nut tree	AY605848	FJ577697	AF399265
III.09	C.P.K. 1084	DAOM 229978	Western Australia	on *Eucalyptus*	FJ716620	FJ577732	EF191268
III.10	J.B.PER62	DAOM 234005	Cusco, Peru	soil under *Eucalyptus*	EF191336	FJ577766	EF191292
III.11	C.P.K. 2673	PPRC R12	Woreda Gera, Ethiopia	coffee plantation	FJ577789	FJ577770	FJ623105
III.12	C.P.K. 2313	UNISS 13b-13	Cuglieri, Sardinia	forest area	EF488113	FJ577756	EF392737
**III.13**	**C.P.K. 939**	**W.M.J. 2274**	**Bavaria, Germany**	**soil**	**FJ577783**	**FJ577718**	**FJ623095**
**III.14**	**C.P.K. 1935**	**W.M.J. 2585**	**Bavaria, Germany**	**on *Fagus sylvatica***	**EF392741**	**FJ577752**	**EF392744**
**III.15**	**C.P.K. 1599**	**W.M.J. 2322**	**Styria, Austria**	**unknown**	**FJ577786**	**FJ577745**	**FJ623100**
**III.16**	**C.P.K. 1941**	**W.M.J. 2786**	**Lower Austria, Austria**	**on *Sambucus nigra***	**EF392742**	**FJ577753**	**EF392745**
**III.17**	**C.P.K. 1934**	**W.M.J. 2545**	**Lower Austria, Austria**	**on *Fagus sylvatica***	**EF392740**	**FJ577751**	**EF392743**

	**Subclade IV**						

IV.01	C.P.K. 590		Atherton, Australia	rhizosphere	EF191320	FJ577704	EF191254
IV.02	C.P.K. 693	TUB F-961***	Beijing, China	park soil	AY857271	FJ577707	FJ623087
IV.03	J.B. NZ1-2	DAOM 233825	Urupakapaka, New Zealand	soil under *Leptospermum*	EF191330	FJ577761	EF191286
IV.04	J.B. NZ7-2	DAOM 233821	Mt. Pureora, New Zealand	soil	EF191332	FJ577763	EF191288
IV.05	C.P.K. 2610	PPRC RW6	Bako, Ethiopia	coffee rhizosphere	FJ577787	FJ577757	FJ623102
IV.06	C.P.K. 53	PPRI 3909	South Africa	unknown	EF113551	FJ577682	EF191249
IV.07	C.P.K. 1044	DAOM 231412	Kigali, Rwanda	sandy clay cultivated soil	AY605764	FJ577719	EF191255
IV.08	C.P.K. 1058	DAOM 231435	Kigali, Rwanda	parkland soil	EF191321	FJ577721	EF191258

	**Subclade V**						

V.01	C.P.K. 1065	DAOM 231405	Isla Mujeres, Q.R., Mexico	sandy soil	AY605774	FJ577725	EF191261
V.02	C.P.K. 646	TUB F-613***	Hookena, Hawai	decaying grass	FJ577780	FJ577706	FJ623086
V.03	C.P.K. 727	TUB F-1082***	Trivandrum, India	plant debris	FJ577781	FJ577709	FJ623088
V.04	C.P.K. 743	TUB F-1236***	Embudu, Maldives	dead bark	FJ577782	FJ577710	FJ623089
V.05	C.P.K. 1059	DAOM 231425	Cancun, Q.R., Mexico	plant soil	EF605768	FJ577722	EF191259

	**Subclade X'/no clade**						

X.01	C.P.K. 836		El-Fayum, Egypt	cotton field	AY605838	FJ577713	FJ623090
X.02	C.P.K. 837	CBS 115333	El-Fayum, Egypt	maize field	AY605839	FJ577714	FJ623091
X.03	C.P.K. 1505	UNISS10.5M	Aoujeft, Mauritania	soil	FJ577784	FJ577743	FJ623098
X.04	C.P.K. 3408****		Papua New Guinea	deep sea sediment	FJ577791	FJ577773	FJ623108
X.05	C.P.K. 3409****		Papua New Guinea	deep sea sediment	FJ577792	FJ577774	FJ623109
X.06	J.B. NZ11-1	DAOM 233829,	Kichappes, New Zealand	soil	EF191333	FJ577764	EF191289
X.07	J.B. NZ2-4	DAOM 233823	Urupakapaka, New Zealand	soil under tree fern	EF191331	FJ577762	EF191287
X.08	C.P.K. 596		Victoria, Brazil	soil	FJ577779	FJ577705	FJ623085
X.09	C.P.K. 709	TUB F-1035	Iguazo Falls, Brazil	tropical rain forest	AY605851	FJ577708	AY605884
X.10	C.P.K. 878	Z.D. 57	Iran	soil	AY602977	FJ577717	FJ623094
X.11	C.P.K. 1066	DAOM 231402	Kaua village, Q.R., Mexico	garden soil	AY605775	FJ577726	EF191262
**X.12**	**C.P.K. 1717**	**G.J.S. 05-62**	**Vietnam**	**unknown**	**EF191325**	**FJ577746**	**EF191281**
X.13	C.P.K. 1064	DAOM 231408	Chichen Itza, Q.R., Mexico	forest soil	AY605773	FJ577724	EF191260
X.14	J.B. PER1-2	DAOM 233966	Iquitos, Peru	soil	EF191334	FJ577765	EF191290
X.16	C.P.K. 239	CBS 115342	Costa Rica	maize field	EF191317	FJ577688	EF191251
**X.17**	**C.P.K. 333**	**G.J.S. 91-159**	**unknown**	**unknown**	**FJ577775**	**FJ577698**	**FJ623080**

	***T. inhamatum***						

X.15	C.P.K. 202 *ex*-type	CBS 273.78	Villavicenco, Colombia	maize field soil	AY605853	FJ577683	AF399271

**Figure 2:**
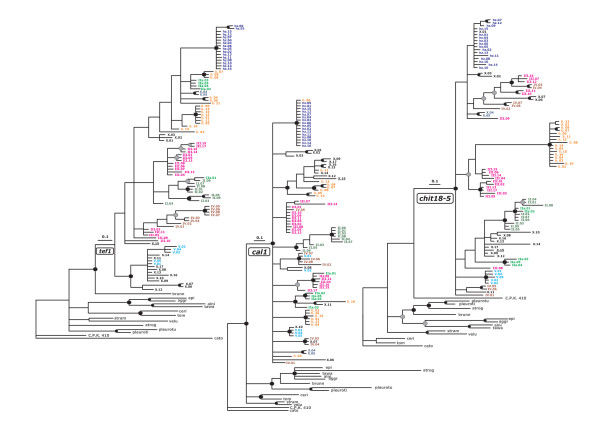
**Single loci trees**. Bayesian trees resulting from analysis of individual sequence sets of *tef1*, *cal1 *and *chi18-5*, respectively. Branching points supported by posterior probabilities > 0.94 are indicated by bold dots, and those supported by > 0.89 but < 0.96 by grey dots. Isolates attributed to subclades on Figure 1 are shown by colours. Species name abbreviation as on Figure 1.

Strains used in this work were either obtained by one of the authors, or taken from previous published work [cf. refs. [11,13,16-19] and citations therein]. "Other collection" names are names that have been used for these isolates in earlier papers. * corresponds to species or clades revealed in this study; ** strain code used in Figures 1 and 2; *** strains obtained in the course of a bilateral research project between TU Vienna to C.P.K. and the Technical University of Budapest (Hungary) to George Szakacs; **** only DNA material available, supplied by Katja Fisch (University of Bonn, Germany); **bold **font highlights strains isolated from teleomorphs;

### Detection of phylogenetic species

We screened among five potential phylogenetic markers for their ability to differentiate within our sample. Besides the finally analyzed loci the initial set included exon fragments of RNA polymerase (*rpb2*) and translation elongation factor 1-alpha (*tef1*) genes. However, only three unlinked loci were sufficiently variable - intron containing regions of the calmodulin (*cal1*) and *tef1 *genes and the coding fragment of the GH18 chitinase gene (*chi18-5)*. Their nucleotide characteristics are given in Additional file 1. We analyzed them as a combined dataset as well as individually.

As seen in Figures 1 and 2, all phylograms confirmed a monophyletic origin for selected 93 strains. Despite the monophyletic origin, the level of intraspecies genetic polymorphism (deduced from the branch lengths) within *H. lixii/T. harzianum *sensu Chaveri *et al*. [16] was comparable with that of interspecific variation within the whole Harzianum-Catoptron Clade (Figure 1 left inset). The application of the genealogical concordance phylogenetic species recognition concept [19], using the criteria of Dettman *et al*. [20] (i.e. that a clade is an independent evolutionary lineage [phylogenetic species] if it is supported in at least one gene tree and not contradicted in any of the others) identified two distinct phylogenetic species - one was a clade that contained the *ex*-type strain of *T. harzianum *(hz.07, = CBS 226.95), which is supported by high posterior probabilities (PP) in *tef1*, *cal1 *and in the combined phylograms and not contradicted in the *chi18-5 *tree (Figures 1 and 2). It includes 15 strains isolated as *Trichoderma *anamorphs from North America, Europe, Siberia and South Africa. Because it contains the *ex*-type strain, we consider it to represent *T. harzianum *sensu stricto and will further term it "*T. harzianum" *throughout the manuscript. The second phylogenetic species, supported with high significance by all three individual tree topologies, contains anamorphic strains of tropical, mainly African origin, and we thus term it *T*. sp. nov.'afroharzianum nom. prov.' (for convenience T. sp. 'afroharzianum', Figures 1 and 2).

The *ex*-type strain of *H. lixii *(li13 = G.J.S. 97-96), the putative teleomorph of *T. harzianum*, occurs in a large clade (Lixii subclade, Figure 1), which is statistically supported in the combined phylogram. However, a comparison of individual gene trees (Figure 2) reveals that this separation is mainly caused by the *chi18-5 *topology, whereas it is contradicted in the *tef1 *and the *cal1 *trees. Nevertheless, a small subclade, which includes both teleomorphs and anamorphs from North America, is supported in the *cal1 *and *tef1 *tree and not contradicted in the *chi18-5 *tree, and we therefore consider this holomorphic subclade as a hypothetical phylogenetic species (*H*. sp. 'paralixii nom. prov.', Figure 1).

The phylogenetic position of the 14 strains in our sample isolated as *Hypocrea *teleomorphs proved to be diverse. One of European origin (li14) clustered in the vicinity of *H*. sp. 'paralixii'. The other European strains were found as terminal branches of Subclade III in the combined phylogram, but this relationship was not supported by individual loci. Two other teleomorph isolates from Vietnam (X.12) and the USA (X.17) could not be attributed to any clade either. Three teleomorph isolates from Cameroon (li07, li08 and li09) formed a supported terminal clade in the *tef1 *and *cal1 *trees, and this was not contradicted by *chi18-5*. We ranked them as another hypothetic phylogenetic species *H*. sp. "cameroonense nom. prov." (Figure 1).

The phylogenetic position of the remaining strains was discordant in trees derived from single loci. We will use the term 'pseudoharzianum matrix' to specify them along with hypothetical phylogenetic species throughout the rest of the paper (Figure 1).

In order to test whether they would be co-specific with any of the "*H. lixii*" lineages identified by Chaverri *et al*. [16], we also included the twelve teleomorph strains investigated by them. The *chi18-5 *allele was omitted in this analysis. The results showed that 9 of these 12 teleomorph isolates also clustered within the Lixii subclade or in Subclade III (data not shown). Of the remaining strains, one (G.J.S. 85-119) was found in Subclade IV, one (G.J.S. 97-106) formed a sister lineage to *T*. sp. 'afroharzianum' within Subclade IIa, and one strain had an unresolved position in the 'pseudoharzianum matrix'.

The detected phylogenetic species, hypothetical phylogenetic species and newly introduced terms with and without taxonomic significance are listed in Table 2.

**Table 2:** Conventional nomenclature derived from phylogenetic analyses

Phylogenetic species:	Taxonomic value
*T. harzianum*	yes
*T*. sp. 'afroharzianum nom. prov.'	yes, awaiting description

**Hypothetical phylogenetic species:**	

*H*. sp. 'paralixii nom. prov.'	no, awaiting verification
*H*. sp. 'cameroonense nom. prov.'	no, awaiting verification

**Lone lineages with unresolved phylogenetic position but belonging to established taxonomic species:**	

*H. lixii*	yes
*T. inhamatum*	yes

**Above species taxonomic units:**	

Harzianum - Catoptron Clade	yes
*H. lixii *- *T. harzianum *species complex or aggregate = *H. lixii/T. harzianum *sensu Chaveri *et al*., [16]	no

**Conventional terms for groups of strains with unresolved relations:**	

'pseudoharzianum matrix' = *H*. sp. 'pseudoharzianum nom. prov. dub.'	no
Lixii Subclade	no
Subclades IIa, III, IV, V and X	no

### Recombination between and within *T. harzianum*, *T*. sp. 'afroharzianum' and strains of the "pseudoharzianum matrix"

The uneven distribution of teleomorph isolates in the *H. lixii - T. harzianum *species complex, particularly their absence in *T. harzianum *s.s. and *T*. sp. 'afroharzianum' but their presence in the Lixii subclade, may suggest that either the tested strains are virtually genetically identical and likely clonal (= clonal sterility) or that all teleomorph stains within the sample were completely reproductively isolated (= interspecific sterility). As these fungi can not be crossed in vitro, we applied three alternative computational methods for detection of genetic recombination from sequence data. To this end we used the *T. harzianum *strains as a control sample for clonal sterility and seven representative strains of species from the Harzianum-Catoptron Clade which were genetically most distant to *H. lixii *and *T. harzianum *as a control for interspecific sterility.

First, the partition homogeneity test (PHT; [21]) was used to examine the congruence between gene trees. This test produces artificial datasets by multiple (10 000) re-sampling and random swapping of observed datasets and subsequent construction of maximum-parsimony trees for every newly sampled 'gene' sequence. For clonally reproducing populations (= no sexual recombination), the sums of the lengths of the gene trees for the observed and re-sampled data should be similar. However, under recombination the sums of the tree lengths should be longer than that for the actual data because of introduction of homoplasy into unlinked sites. This test confirmed our analysis of topologies of single locus trees - the clades containing *T. harzianum *and *T*. sp. 'afroharzianum' showed congruence of data suggesting their clonality (Figure 1). Other clades, which were incongruent in different trees, provided evidence for recombination. However, recombination was also not detected with strains occurring at unresolved positions on the combined tree (X.01 - X.16) including the *ex*-type strain of *T. inhamatum *(X.15 = CBS 273.78) and two strains isolated as teleomorphs (Figure 1). As none of these strains fulfilled the criteria of a phylogentic species, we assume that they represent multiple relict lineages which are incompatible of recombination either due to the loss of sexuality or due to mating incompatibility evolved in a course of habitat isolation. The relatively long genetic distances between them support this view. The controls (= representatives of Harzianum-Catoptron Clade) produced essentially negative results (Figure 1, right inset).

As a second means, we used the index of association (IA) test on a subset of 'clone corrected' data (i.e. individuals with identical alleles of the three loci were excluded so that each haplotype was represented only once; cf. [22]). In this test, complete panmixia (sexual compatibility resulting in recombination) would be indicated by a value of 0 (= the null hypothesis). This value was neither obtained with the complete dataset nor with any of the individual clades (data not shown). Yet the Lixii subclade, and Subclades III and IV gave values lower than 1, thus supporting the null hypothesis of sexual compatibility between strains within them. In contrast *T. harzianum *and *T*. sp. 'afrohazianum' yielded values above 1, rejecting the recombination. Subclade V was not analyzed as it consisted of only two concatenated haplotypes. In accordance with results from PHT, IA values for strains at unresolved positions (X.01 - X.16) also showed no evidence for recombination.

Finally we applied the Phi-test, which uses the pairwise homoplasy index (PHI, Ö) to detect refined incompatibility [23]. This method assumes the infinite sites model of evolution [24] in which the detection of incompatibility for a pair of sites indicates recombination. Application of this test to the same subsamples based on phylogenetic species and clades of the combined tree confirmed the results of the previous analyses, and also detected no recombination in *T. harzianum*, *T*. sp. 'afroharzianum' and between representative strains of the Harzianum-Catoptron Clade (*P *= 0.1897, *P *= 0.2773 and *P *= 0.3406, respectively). All other subclades showed positive recombination signals (*P *< 0.05). The corresponding network, produced by Splitstree, is shown on the inset on the right bottom of Figure 1.

Since the Phi-test is a very robust means which can detect recombination even in the presence of recurrent mutation, we decided to use this method to define the borders of recombining populations. To this end, we first set up a non-recombining sample consisting of the most terminal strains of a clade to be investigated, and the most distant strains from other phylogenetic species. Then we gradually added phylogenetically closer strains until evidence for recombination was detected (*P *< 0.05). To determine the outer border of the recombining population, we excluded the first strain which indicated recombination and started to add phylogenetically more distant isolates from the same subclade or clade. Such approach has the advantage that it also allows the analysis of small subclades which can not be analysed alone due to insufficient data. This technique was applied to all meaningful combinations of species/clades/subclades which were present on the phylograms shown (around 90 individual tests). The positive results (recombination, P < 0.05) obtained are shown by shadowed areas on the main part of Figure 1, and provided interesting insights into the reproduction history of the clades found. For example, although the isolates of *T. harzianum *were proven to be clonal by all three methods used, they still revealed a recombination history with strains X.01 - X.03 from Northern Africa which occupied an otherwise unresolved phylogenetic position in the *tef1*, *cal1 *and *chi18-5 *trees. Another presumably recent agamospecies, *T*. sp. 'afroharzianum', exhibits a history of recombination with five phylogenetically plesiotypic strains of different geographic origins (Figure 1). Not all strains of the Lixii subclade showed a recombination history in this test. Nevertheless two groups with positive recombination signal were detected - one includes strains of *H*. sp. 'paralixii' and two temperate strains from North America and Europe; the second covers the latter two strains, *H*. sp. 'cameroonense' and the *ex*-type strain of *H. lixii*. A limited recombination was also detected for strains from Subclade III occupying terminal positions - three Austrian teleomorphs and two anamorphic strains isolated from Sardinian soils were recombining with each other but not with strains occupying basal branches of the subclade. Similarly, the four terminal strains from a Subclade IV (all from Africa) display a history of recombination. In addition, all strains of the panmictic subclade V showed evidence for recombination. This result corresponds to the fact that they occupy conflicting positions in the single gene trees but belong to the same subclade of *ech18-5 *phylogram indicating their common evolutionary path. The most genetically polymorphic isolates of the 'pseudoharzianum matrix', which formed almost no phylogenetic structure (Subclade X), showed no evidence for recombination signal when they were confronted either with each other or with other subclades. However, when some of them were analysed together with representatives of the Harzianum - Catoptron Clade, a significant recombination signal was detected indicating traces of interspecific sexual compatibility (open shadow area on Figure 1).

### Population divergence and stability

To estimate the degree of differentiation within the 'pseudoharzianum matrix', we applied methods for analysis of populations and computed the F_ST _values [25] for the main subclades of the combined phylogram. Qualitatively, an F_ST _value in the range close to 0 indicates low differentiation (= fixation of characters) between populations (compared populations are composed of equally different individuals) and close to 1 indicates great differentiation when populations are composed of similar individuals but there is a big difference between populations [26]. F_ST _values between *T. harzianum *and other subclades were in the range of 0.67 - 0.83 indicating essential genetic separation of this species. The F_ST _value between Subclades III and IV of the 'pseudoharzianum matrix' was low (0.17) which may indicate that some genetic exchange still occurs between their strains.

The population parameters θ (for haploids 2 Nm, where N is effective population size and m is the mutation rate per site and generation) and population growth rates were calculated using LAMARC package (see Methods). Consistent with the occurrence of *H. lixii *- *T. harzinum *species complex as one of the most frequent taxon of the genus, the growth rate of most subclades is large, which is indicative of a large effective population size and population expansion (data not shown). The recombining subclades had the highest growth rate. Only *T. harzianum *exhibited a significantly smaller value for population growth (5.90) and also a 5-20-fold smaller θ-value than the other subclades. This suggests that this species apparently occupies a niche with a limited potential for expansion.

## Discussion

The perception of fungal species as dynamic entities which arise, persist for a longer or shorter period, modify, decline and then become extinct and replaced by other species leads to a variety of existing species concepts in mycology. In addition, the taxonomy of almost every large fungal genus is biased either because of its importance to mankind (e.g. for convenient differentiation of pathogenic or industrial organisms) or by its history of taxonomic description. Here we have analysed *H. lixii *- *T. harzianum *species complex, one of the most commonly sampled groups of fungi because of its dominant presence in the majority of soil ecosystems worldwide and its occupation of a broad diversity of ecological niches. From the results of this paper, the evolutionary success of the *H. lixii - T. harzianum *species aggregate may be attributed to the very complex structures of the contemporary populations of these fungi, which can be differentiated into nearly all possible 'types' of fungal species: reproductively isolated biological species, sympatric and allopatric phylogenetic species, recent agamospecies and numerous relict lineages with unresolved phylogenetic positions. This complexity comes from the fact that many of these species 'types' are overlapping and therefore it frequently happens that two closely related organisms become attributed to different species recognized based on incomparable criteria. Our data illustrate a speciation history within *H. lixii *- *T. harzianum *species complex, which is based on coexistence and interaction of organisms with different evolutionary strategies and on the absence of strict genetic borders between them.

The genealogical concordance phylogenetic species recognition [GCPSR, [19]] concept is the most advanced approach to defining species in modern fungal taxonomy. Yet our results demonstrate that even this method has its limits within a population of isolates with different recombination histories. Basically, the whole of the *H. lixii *- *T. harzianum *species complex might be considered as a single species as its monophyletic origin is supported by all three loci tested in this paper. Chaveri *et al*. [16] used the indistinguishable morphology of these fungi in favour of such a conclusion. However, this approach appears to be invalid when *T. harzianum *sensu Chaveri *et al*. [16] is compared with its genetically closest neighbouring species. The genetic distances calculated within *H. lixii *- *T. harzianum *species complex are comparable to the distances between well diverged species of the Harzianum-Catoptron Clade, which are characterized by different phylogenies, morphologies, physiologies and ecological characteristics [17]. Within the *H. lixii - T. harzianum *complex, a conservative application of the GCPSR concept justifies two anamorphic phylogenetic species (*T. harzianum *and *T*. sp. 'afroharzianum'), and does not contradict postulating two further holomorphic phylogenetic species (*H*. sp. 'paralixii' and *H*. sp. 'cameroonensis'). No consistent phylogenetic structure could be detected, however, in the remaining strains.

The results from this work also shed new light on the concept of the *H. lixii/T. harzianum *holomorph and the synonymization of *T. inhamatum *with *T. harzianum*. All methods used clearly differentiated *T. harzianum *sensu stricto from the remaining isolates as an agamospecies with global distribution in temperate ecosystems. Since *T. harzianum *and *H. lixii *are genetically isolated and evidently do not appear in the same life cycle, the holomorph *H. lixii/T. harzianum *must therefore be rejected. Also, the unclear phylogenetic position of the *ex*-type strain of *H. lixii*, and the fact that teleomorphs with '*H. lixii*' morphology are also present in subclade III which is reproductively isolated from *H. lixii *sensu stricto, render the naming of most of the isolates investigated here as "*H. lixii*" doubtful. Finally, we show that *T. inhamatum *is a separate phylogenetic lineage only distantly related to *H. lixii *or to *T. harzianum*, and its name should therefore be maintained.

Recombination is a powerful evolutionary force that merges historically distinct genotypes. Yet the extent of recombination within many organisms is unknown, and even determining its presence within a set of homologous sequences is a difficult question. Molecular traces of sexual recombination were detected for the majority of tested strains and phylogenetic species, and were correlated with the occurrence of most of the teleomorph isolates within recombining clades. This finding is the opposite of what has been seen with other apparently asexual fungi such as the human pathogens *Coccidioides posadasii *[27], *C. immitis *[28] and *Paracoccidioides brasiliensis *[29], the facultative pathogen *Aspergillus fumigatus *[22], and the mycotoxin producer *A. flavus *[30]. In all these cases, teleomorphs were not found, whereas phylogenetic evidence for recombination was obtained. In our sample, such a situation was seen only for Subclade V which is composed of evidently recombining strains exclusively isolated as anamorphs.

In this study, we introduced a trial-and-error approach to detect borders of recombination within a sample. The limited distribution of sexual recombination within the phylogenetic clades underlines that these fungi pass through periods of sexual and asexual recombination. Based on the unresolved structure of the "pseudoharzianum matrix" in the individual and combined phylogenetic trees, we originally expected sexual compatibility between all of its strains. Yet our data showed that distantly related strains from the same subclades had already lost their ability for genetic exchange. The most striking example was the absence of recombination between the *H. lixii ex*-type strain and two other teleomorphic strains from the same subclade, which further supports the postulation of *H*. sp. 'paralixii' as a separate albeit closely related taxon.

With the exception of *H*. sp. 'paralixii' and Subclade IV (whose isolates were exclusively derived from Africa, South-East Asia, Australia and New Zealand), all other clades exhibited a global geographic distribution. While a worldwide distribution of fungi was at one time believed to be the rule, the application of molecular genetic methods has recently shown that most of these globally distributed taxa actually consist of several allopatric cryptic species. To the best of our knowledge, the only other similar finding of panmixis for a mitosporic fungus has recently been presented for *A. fumigatus *[22]. The fact that most of the clades detected in this study could maintain (in part) a panmictic population is interesting, because it is known (e.g. for insects) that dispersal and subsequent allopatric speciation can occur in very short times, even in the absence of severe population bottlenecks [31,32]. This lack of allopatric speciation - which otherwise seems to occur in several other species of *Hypocrea/Trichoderma *[cf. [33,34]] - must be due to a continuous, unrestricted gene flow, whose mechanism warrants being identified.

*T. harzianum *sensu Chaveri *et al*. [16] is one of several *Trichoderma *species which are successfully used in biological control of plant pathogenic fungi. The results from this study show that the respective strains must be members of one of several phylogenetic species with different recombination frequencies. In a preliminary test of a few commercially used "*T. harzianum*" biocontrol agents, none of them showed the gene sequences characteristic for the strains from the clonal *T. harzianum *and *T*. sp. 'afroharzianum' clades, and therefore may be members of the recombining populations and phylogenetic species (C.P. Kubicek, unpublished data). Recombination of a biocontrol strain clearly could have a significant impact on its stability in the field. A study aiming at clarifying this situation is currently in progress.

Fungi belonging to 'pseudoharzianum matrix' are abundantly isolated from various environments and are frequently selected as biocontrol strains. Therefore their correct identification is of great importance. This study shows that because some of them are able to recombine while others are probably sexually incompatible, their attribution to a single taxon would be very doubtful. We suggest the introduction of the provisional temporary name *H*. sp. 'pseudoharzianum nom. prov. dub.' in order to separate strains belonging to 'pseudoharzianum matrix' from phylogenetic species such as *T. harzianum *s.s., *T*. sp. 'afroharzianum', *H. lixii *s.s. and *T. inhamatum*, which can be correctly identified by the analysis of *tef1 *4^th ^large intron sequence using either the similarity search tools or phylogeny. The development of an integrated bioinformatic tool for the haplotype-based identification of fungi within *H. lixii - T. harzianum *species complex and for the potential prediction of their mycoparasitic abilities has been started in the laboratory of the corresponding author.

## Conclusions

In this work we did not defeat the 'harzianum demon'. However, we demonstrated its power, span and perhaps its limits, and believe that the current study provides some understanding of the forces driving speciation in these fungi. A major challenge of future work will be the elaboration of standardized methods by which the phylogenetic species and the 'pseudoharzianum matrix' detected here can be conveniently differentiated with predictable biological activities. Preliminary data in our laboratory showed that the use of 95-carbon source phenotype microarrays may be helpful in this regards (J. Bissett and I.S. Druzhinina, unpublished data).

## Methods

### Strains and gene sequences

The strains, sequences and NCBI GenBank accession numbers are listed in Table 1, and are maintained in long-term storage facilities at Vienna University of Technology (Austria) and ECORC (Canada) laboratories.

### DNA extraction, PCR amplification and sequencing

Mycelia were harvested after 2 - 4 days of growth on malt extract agar at 25°C and genomic DNA was isolated using QIAGEN DNeasy^® ^Plant Maxi Kit following the manufacturer's protocol. Amplification of DNA fragments comprising ITS1 and 2 and the 5.8S rRNA gene, the endochitinase *chit18-5 *(= *ech42*; [35]) and the large 4^th ^intron of *tef1*, amplicon purification and sequencing was performed as described in detail previously [36]. Previously published sequences used for phylogenetic analyses in this study were retrieved from GeneBank and are identified by their respective accession numbers.

### Phylogenetic analyses

For the phylogenetic analysis DNA sequences were aligned with Clustal X 1.81 [37]. As the 4^th ^large intron of *tef1 *is highly polymorphic, the alignment contained several ambiguous areas which could contain homoplasiuos characters resulted from multiple substitutions and/or saturation. In order to detect such areas we have processed the concatenated alignment using the Gblocks server [[38], URL: http://molevol.cmima.csic.es/castresana/Gblocks_server.html]. When the default stringent parameters of Gblocks were applied, nearly complete locus of the *tef1 *intron has been removed (not shown); when the less stringent options were used, only 10% of the alignment was removed leaving the considerable part of *tef1 *intron in the analysis (Additional file 2). The poorly aligned areas of *tef1 *selected by Gblocks have been carefully edited manually by inserting extra gap columns in order to reduce the difference between sequences originating from hypothetically homoplasious characters. The original and edited alignments are available from Additional file 3. The possibility of intragenic recombination, which would prohibit the use of the respective loci for phylogenetic analysis, was tested by linkage disequilibrium based statistics as implemented in DnaSP 4.50.3 [39]. The neutral evolution of coding fragments (*cal1 *and *chi18-5*) was tested by Tajima test implemented in the same software. The interleaved NEXUS files were formatted using PAUP*4.0b10 [40]. The best nucleotide substitution model for the each locus was determined using jMODELTEST [41]. As Akaike and Bayesian Information criteria (AIC [42] and BIC [43], respectively) selected different nucleotide substitution models for every locus and due to the relatively small size of individual datasets (1379 characters per 107 sequences for the biggest) the unconstrained GTR + I + G substitution model was applied to all sequence fragments (Additional file 1). Metropolis-coupled Markov chain Monte Carlo (MCMC) sampling was performed using MrBayes v. 3.0B4 with two simultaneous runs of four incrementally heated chains that performed 5 million generations. The length of run (number of generations) for each dataset was determined using AWTY graphical system [44] to check the convergence of MCMC. Bayesian posterior probabilities (PP) were obtained from the 50% majority rule consensus of trees sampled every 100 generations after removing the first trees using the "burnin" command. Number of discarded generations was determined for every run based on visual analysis of the plot showing generation versus the log probability of observing the data. According to the protocol of Leache and Reeder [45] PP values lower than 0.95 were not considered significant while values below 0.9 are not shown on the resulting phylograms. Phylograms calculated based on alignments trimmed in Gblocks are given in Additional file 4. The phylogenetic tree inferred based on the complete corrected alignment was used for the data interpretation. Model parameters summaries after MCMC run and burning first samplings as well as nucleotide characteristics of used loci are collected in Additional file 1.

### Detection of Recombination

The congruence or incongruence of the three gene genealogies was used to infer recombination between isolates, thereby excluding isolates of the "*sensu stricto*" group (see Results). To this end, three different tests were employed: the incongruence length difference/partition homogeneity test (ILD/PHT) [21,46] using a score of *P *< 0.05 to reject the null hypothesis of congruence between loci; the Index of Association [IA; [47]] test, in which the data were compared to the IAs of artificially recombined datasets [27,48]; and the Phi-test implemented in SplitsTree, which uses the pairwise homoplasy index, PHI (= Φ) statistic, to detect refined incompatibility indicating recombination [23].

In addition we applied split decomposition implemented in the SplitsTree program, version 4.0 [49], using pairwise distances under the Kimura 3-ST model [24]. This method visualizes recombination events by depicting the shortest pathway linking sequences, rather than forcing them into a bifurcating tree [49].

### Population divergence and stability

Population growth parameters and theta (θ) values were inferred using the program LAMARC 2.0 [50]. All polymorphic sites were used to assess the population parameters. To estimate the population growth parameter, we used 10 initial chains with 2,000 genealogies sampled and two final chains with 20,000 genealogies sampled. Population parameters were inferred using both Bayesian and Maximum Likelihood criteria.

## Authors' contributions

ISD wrote the final version of the paper, and performed the phylogenetic and recombination analyses and interpretations; CPK designed the study and the sample, performed the population analyses and participated in writing a draft version of the paper; MKZ and TBM performed the molecular laboratory work; JB provided additional strains and their sequences and contributed to the text of the final version of the paper. All authors read and approved the final manuscript.

## Supplementary Material

Additional file 1 **Nucleotide properties of used loci and details of phylogenetic analyses**. A table showing detailed nucleotide properties of all three loci used and of phylogenetic analyses made for all of them individually and for the concatenated data set. Click here for file

Additional file 2 **Outcome of the Gblocks analysis applied to the concatenated alignment of three loci**. A complete three loci alignment showing highly variable but poorly aligned areas of *tef1 *intron detected after the processing of the data with Gblocks. Click here for file

Additional file 3 **Multiple sequence alignments showing the results of adjustments of variable**. The poorly aligned areas of *tef1 *selected by Gblocks have been carefully edited manually by inserting extra gap columns in order to reduce the difference between sequences originating from hypothetically homoplasious characters. Click here for file

Additional file 4 **Tree files calculated based on alignments trimmed in Gblocks**. A tree file containing phylogenetic trees resulted after the analysis performed with datasets reduced by stringent and less stringent procedures of Gblocks. Click here for file
