# Supplementary material for: The Trichoderma harzianum demon: complex speciation history resulting in coexistence of hypothetical biological species, recent agamospecies and numerous relict lineages
Source: BMC Evol Biol. 2010 Apr 1;10:94. doi: 10.1186/1471-2148-10-94 (PMC2858147; doi:10.1186/1471-2148-10-94)
Supplement: Additional file 1 — Nucleotide properties of used loci and details of phylogenetic analyses. A table showing detailed nucleotide properties of all three loci used and of phylogenetic analyses made for all of them individually and for the concatenated data set. [file 1471-2148-10-94-S1.DOC]

Table 2. Nucleotide properties of used loci and details of phylogenetic analyses

| Parameters | phylogenetic marker | | | |
| --- | --- | --- | --- | --- |
|  | *tef1* | *cal1* | *chi18-5* | concatenated dataset |
| Fragment characterization | intron | exon/intron | exon | not applicable |
| Number of sequences | 107 | 107 | 107 | 107 |
| Number of characters: total/pars. inform./constant | 330 / 113 / 164 | 434 / 105 / 272 | 615 / 125 / 431 | 1379 / 343 / 867 |
| **Parameters of MCMC analysis** | | | | |
| Substitution models selected by AIC and BIC | TrN+G and K80 | TIM2+G and K80 + G | TIM1+G and HKY+G |  |
| Mean nt frequencies* A / C / G / T | 0.18 / 0.31 / 0.18 / 0.33 | 0.27 / 0.28 / 0.24 / 0.21 | 0.20 / 0.34 / 0.24 / 0.22 | not applicable |
| Substitution rates* A-C/A-G/A-T/C-G/C-T/G-T | 0.07 / 0.52 / 0.05 / 0.09 / 0.20 / 0.07 | 0.07 / 0.37 / 0.08 / 0.06 / 0.31 / 0.11 | 0.11 / 0.24 / 0.05 / 0.06 / 0.46 / 0.09 |  |
| alpha* | 0.12 | 0.11 | 0.1 | 0.25 |
| Number of generations / discarded first generations | 5 000 000 / 600 | 5 000 000 / 400 | 5 000 000 / 500 | 5 000 000 / 800 |
| Total tree length | 18.82 | 20.59 | 20.33 | 4.97 |
| **DNA polymorphism analysis** | | | | |
| Number of sites excluding gaps and missing data | 53 | 112 | 159 | 324 |
| Segregation sites / Number of haplotypes | 16 / 16 | 43 / 30 | 36 / 34 | **95 / 60** |
| Haplotype diversity / Nucleotide diversity, Pi | 0.83 / 0.04 | 0.93 / 0.04 | 0.94 / 0.03 | 0.97 / 0.04 |
| **Neutrality analysis** | | | | |
| Tajima test | n.s. (-1.18, *P*>0.1) | n.s. (-1.26, *P*>0.1) | not applicable | not applicable |

* as estimated after GTR MCMC sampling and burning AIC and BIC are Akaike Information Criterion [37] and Bayesian Information *C*riterion [38] as implemented in jMODELTEST [46]; TIM1 [47] and TIM2 [46] nucleotide substitution model with six free parameters and unequal base frequencies; TN [47] nucleotide substitution model with 5 free parameters and unequal base frequencies; K80 [48] nucleotide substitution model with one free parameter and equal base frequencies; HKY [49] nucleotide substitution model with four free parameters and unequal base frequencies; GTR [50] General Time Reversible model with eight free parameters, I - proportion of invariable sites and G - gamma rates.
