# Supplementary material for: The Trichoderma harzianum demon: complex speciation history resulting in coexistence of hypothetical biological species, recent agamospecies and numerous relict lineages
Source: BMC Evol Biol. 2010 Apr 1;10:94. doi: 10.1186/1471-2148-10-94 (PMC2858147; doi:10.1186/1471-2148-10-94)
Supplement: Additional file 2 — Outcome of the Gblocks analysis applied to the concatenated alignment of three loci. A complete three loci alignment showing highly variable but poorly aligned areas of tef1 intron detected after the processing of the data with Gblocks. [file 1471-2148-10-94-S2.PDF]

Gblocks 0.91b Results

Processed file: **pasted_alignment**
Number of sequences: **112**
Alignment assumed to be: **DNA**
New number of positions: **1258** (selected positions are underlined in blue)

```
                          10        20        30        40        50        60
                 =========+=========+=========+=========+=========+=========+
1093CAN .        GCCCGATAATTCTGCAGAGAATTTTCG-TGT-CGACAATTTTT----CATCACCCC-GAT
1052SA .         GCCCGATAATTCTGCAGAGAATTTTCG-TGT-CAACAATTTTT----CATCACCCC-GAT
1116CAN .        GCCCGATAATTCTGCAGAGAATTTTCG-TGT-CGACAATTTTT----CATCACCCC-GAT
1070EIRE .       GCCCGATAATTCTGCAGAGAATTTTCG-TGT-CGACAATTTTT----CATCACCCC-GAT
1099CAN .        GCCCGATAATTCTGCAGAGAATTTTCG-TGT-CGACAATTTTT----CATCACCCC-GAT
1104CAN .        GCCCGATAATTCTGCAGAGAATTTTCG-TGT-CGACAATTTTT----CATCACCCC-GAT
1087CAN .        GCCCGATAATTCTGCAGAGAATTTTCG-TGT-CGACAATTTTT----CATCACCCC-GAT
360UK .          GCCCGATAATTCTGCAGAGAATTTTCG-TGT-CGACAATTTTT----CATCACCCC-GAT
2111HUN .        GCCCGATA-TTCTGCAGAGAATTTTCG-TGT-C-ACAATTTTT----CATCACCCC-GAT
217RUS .         GCCCGATAATTCTGCAGAGAATTTTCG-TGT-CGACAATTTTT----CATCACCCC-GAT
204UK .          GCCCGATAATTCTGCAGAGAATTTTCG-TGT-CGACAATTTTT----CATCACCCC-GAT
206RUS .         GCCCGATAATTCTGCAGAGAATTTTCG-TGT-CGACAATTTTT----CATCACCCC-GAT
261SIB .         GCCCGATAATTCTGCAGAGAATTTTCG-TGT-CGACAATTTTT----CATCACCCC-GAT
265SIB .         GCCCGATAATTCTGCAGAGAATTTTCG-TGT-CGACAATTTTT----CATCACCCC-GAT
2784THAI .       GCCCGACAATTCTGCAGAGAATTTTCG-TGT-CGACAATTTTT----CATCACCCC-GCT
838EGY .         GCCCGACAATTCTGCAGAGAATTTTGG-TGT-CGACAATTTTT----CATCACCCC-GCT
1102CAN .        GTCCGACAATTCTGCAGAGAATTTTCG-TGT-CAACAATTTTT----CATCACCCC-GCT
588LAOS .        GTCCGACAATTCTGCAGAGAATTTTCGATGT-CGACAATTTTT----CATCACCCC-GCT
1596AT .         GTCCGACAATTCTGCAGAGAATTTTCG-TGT-CAACAATTTTT----CATCACCCC-GCT
1720CAM .        --CCGACAATTCTGCAGAGAATTTTCG-TGT-CAACAATTTTT----CATCACCCC-GCT
1722CAM .        XXXXXXXXXTTCTGCAGAGAATTTTCG-TGT-CAACAATTTTT----CATCACCCC-GCT
1724CAM .        GCCCGAC-ATTCTGCAGAGA-TTTTCG-TGT-CAACAATTTTT----CATCACCCC-GCT
334USA .         GTCCGACAATTCTGCAGAGAATTTTCG-TGT-CAACAATTTTT----CATCACCCC-GCT
335USA .         GTCCGACAATTCTGCAGAGAATTTTCG-TGT-CAACAATTTTT----CATCACCCC-GCT
1081WIS .        GCCCGACAATTCTGCAGAGAATTTTCG-TGT-CGACAATTTTT----CATCACCCC-GCT
1069WIS .        GTCCGACAATTCTGCAGAGAATTTTCG-TGT-CAACAATTTTT----CATCACCCC-GCT
1107CAN .        GTCCGACAATTCTGCAGAGAATTTTCG-TGT-CAACAATGTTT----CATCACCCC-GCT
1108PEN .        GTCCGACAATTCTGCAGAGAATTTTCG-TGT-CAACAATGTTT----CATCACCCC-GCT
1110CAN .        GTCCGACAATTCTGCAGAGAATTTTCG-TGT-CAACAATTTTT----CATCACCCC-GCT
JBSER241 .       GCCCGACAATTCTGCAGAGAATTTTCG-TGC-CGACAATTTTT----CATCACCCC-GCT
246PHI .         GCCCGACAA-----------------------------TTTTT----CATCACCCC-GCT
51SA .           GCCCGACAATTCTGCAGAGAATTTTCG-TGT-CGACAATTTTT----CATCACCCC-GCT
2618ETHI .       GCCCGACAATTCTGCAGAGAATTTTCG-TGT-CGACAATTTTT----CATCACCCC-GCT
1068WIS .        GTCCGACAATTCTGCAGAGAATTTTCG-TGT-CAACAATTTTT----CATCACCCC-GCT
238CR .          GCCCGACAATTCTGCAGAGAATTTTCG-TGT-CGACAATTTTT----CATCACCCC-GCT
NR5555 .         GCCCGACAATTCTGCAGAGAATTTTCG-TGT-CGACAATTTTT----CATCACCCC-GCT
NR6839 .         GCCCGACAATTCTTCAGAGAATTTTCG-TGT-CGACAATTTTT----CACCACCCC-GCT
2624ETHI .       GCCCGACAATTCTGCAGAGAATTTTCG-TGT-CGACAATTTTT----CACCCCCCCCGCT
845EGY .         GCCCGACAATTCTGCAGAGAATTTTCG-TGT-CGACAATTTTT----CACCCCCCCCGCT
JBPER12 .        GCCCGACGATTCTGAAGAGAATTTTCG-TGT-CGACAATTTTT----CGTCACCCC-GCT
1717VIET .       GCCCGACGATTCTGAAGAGA-TTTTCG-TGT-CGACAATTTTT----CGTCACCCC-GCT
709BRAZ .        GCCCGACGATTCTGAAGAGAATTTTCG-TGT-CGACAATTTTT----CGTCACCCC-GCT
878IRAN .        GCCCGACGATTCTGAAGAGAATTTTCG-TGT-CGACAATTTTT----CGTCACCCC-GCT
239CR .          GCCCGACGATTCTGAAGAGAATTTTCG-TGT-CGACAATTTTT----CGTCACCCC-GCT
202COM .         ACCCGACAATTCTGAAGAGAATTTTCG-TGTTCGACAATTTTT----CATCACCCC-GCT
1064MEX .        GCCCGACGATTCTGAAGAGAATTTTCG-TGT-CGACAATTTTT----CGTCACCCC-GCT
596BRAZ .        GCCCGACGATTCTGAAGAGAATTTTCG-TGT-CGACAATTTTT----CGTCACCCC-GCT
333USA .         GCCCGACGATTCTGAAGAGAATTTTCG-TGT-CGACAATTTTT----CGTCACCCC-GCT
2610ETHI .       GCCCGACAATTCTTCAGAC--TTTTTG-GGT-CGACAATTTTT----CGTCACCCC-GCT
1044RW .         GCCCGACAATTCTTCAGAC--TTTTTG-GGT-CGACAATTTTT----CGTCACCCC-GCT
JBNZ111 .        GCCCGGCGATTCTGAAGAGAATTTTCG-TGT-CGACAACTTTT----CGTCACCCC-GCT
JBNZ12 .         GCCCGACAATTCTTCAGAGAATTTTCG-TGT-CGACAATTTTT----CATCACCCC-GCT
JBNZ24 .         GCCCGGCGATTCTGAAGAGAATTTTCG-TGT-CGACAACTTTT----CGTCACCCC-GCT
693CHINA .       GCCCGACAATTC---AGAGAATTTTCG-TGT-CGACAATTTT-----CATCACCCC-GCT
1058RW .         GCCCGACAATTCTTCAGAC--TTTTTG-GGT-CGACAATTTTT----CGTCACCCC-GCT
590AUS .         ACCCGACAATTCTGCAGAGAATTTTGG-TGT-CGACAATTTTT----CATCACCCC-GCT
53SA .           GCCCGACAATTCTTCAGAC--TTTTTG-GGT-CGACAATTTTT----CGTCACCCC-GCT
1075WIS .        GCCCGACAATTCTGCAGAGAATTTTCG-TGT-CGACAATTTTT----CATCACCCC-GCT
939GER .         GCCCGACAATTCTGCAGAGAATTTTCG-TGT-CGACAATTTTT----CATCACCCC-GCT
GA3804 .         GCCCGACAATTCTGCAGAGAATTTTCG-TGT-CGACAATTTTT----CATCACCCC-GCT
1599AT .         GCCCGACAATTCTGCAGAGAATTTTCG-TGT-CGACAATTTTT----CATCACCCC-GCT
1941AT .         GCCCGACA-TTCTGCAGAGAATTTTCG-TGT-CGACAATTTTT----CATCACCCC-GCT
291NE .          GCCCGACAATTCTGCAGAGAATTTTCG-TGT-CGACAATTTTT----CATCACCCC-GCT
2301SAR .        GCCCGACAATTCTGCAGAGAATTTTCG-TGT-CGACAATTTTT----CATCACCCC-GCT
2313SAR .        GCCCGACAATTCTGCAGAGAATTTTCG-TGT-CGACAATTTTT----CATCACCCC-GCT
271NE .          GCCCGACAATTCTGCAGAGAATTTTCG-TGT-CGACAATTTTT----CATCACCCC-GCT
1935GER .        GCCCGACAATTCTGCAGAGAATTTTCG-TGT-CGACAATTTTT----CATCACCCC-GCT
JBRSA122 .       GCCCGACAATTCTGCAGAGAATTTTCG-TGT-CGACAATTTTT----CATCACCCC-GCT
276NE .          GCCCGACAATTCTGCAGAGAATTTTCG-TGT-CGACAATTTTT----CATCACCCC-GCT
272NE .          GCCCGACAATTCTGCAGAGAATTTTCG-TGT-CGACAATTTTT----CATCACCCC-GCT
2673ETHI .       GCCCGACAATTCTGCAGAGAATTTTCG-TGT-CGACAATTTTT----CATCACCCC-GCT
1084AUSW .       GCCCGACGATTCTGCAGAGAATTTTCG-TGT-CGACAATTTTT----CATCACCCC-GCT
JBPER62 .        GCCCGACAATTCTGAAGAGAATTTTCG-TGT-CGACAATTTTT----CATCACCCC-GCT
JBNZ72 .         GCCCGACAATTCTTCAGAGAATTTTCG-TGT-CGACAATTTTT----CATCACCCC-GCT
1065MEX .        GCCCGACGATTCTGAAGAGAATTTTCG-TGT-CGACAATTTTT----CGTCACCCC-GCT
646HAWAI .       GCCCGACGATTCTGAAGAGAATTTTCG-TGT-CGACAATTTTT----CGTCACCCC-GCT
727INDIA .       GCCCGACGATTCTGAAGAGAATTTTCG-TGT-CGACAATTTTT----CGTCACCCC-GCT
743MALE .        GCCCGACGATTCTGAAGAGAATTTTCG-TGT-CGACAATTTTT----CGTCACCCC-GCT
1059MEX .        GCCCGACGATTCTGAAGAGAATTTTCG-TGT-CGACAATTTTT----CGTCACCCC-GCT
1061RW .         GCCCGACAATTCTGCAGAGAATTTTCG-TGT-CGACAATTTTT----CATCACCCC-GCT
1066MEX .        GCCCGACGATTCTGAAGAGAATTTTCG-TGT-CGACAATTTTT----CGTCACCCC-GCT
1095BALI .       GCCCGACAATTCTGCAGAGAATTTTCG-TGT-CGACAATTTTT----CATCACCCC-GCT
1505MAUR .       GCCCGACAATTCTGCAGAGAATTTTCG-TGT-CGACAATTTTT----CATCACCCC-GCT
1818ETHI .       GCCCGATAATTCTGCAGAGAATTTTCG-TGT-CGACAATTTTT----CATCACCCC-GAT
1934AT .         GCCCGACA-TTCTGCAGAGAATTTTCG-TGT-CGACAATTTTT----CATCACCCC-GCT
245CR .          GCCCGACAATTCTGCAGAGAATTTTCG-TGT-CGACAATTTTT----CATCACCCC-GCT
2710ETHI .       GCCCGACAATTCTGCAGAGAATTTTCG-TGT-CGACAATTTTT----CATCACCCC-GCT
274NE .          GCCCGACAATTCTGCAGAGAATTTTCG-TGT-CGACAATTTTT----CATCACCCC-GCT
3408PNG .        GCCCGACAATTCTGCAGAGAATTTTCG-TGT-CAACAATTTTT----CATCACCCC-GCT
3409PNG .        GCCCGACAATTCTGCAGAGAATTTTCG-TGT-CAACAATTTTT----CATCACCCC-GCT
410MALAY .       GCCCGACAATTCTGCACAGGATTTTCG-TGT-CAACAATTTTTT--CCATCACCCC-GCT
836EGY .         GCCCGACAATTCTGCAGAGAATTTTCG-TGT-CGACAATTTTT----CATCACCCC-GCT
837EGY .         GCCCGACAATCCTACAGAGAATTTTCG-TGT-CGACAATTTTT----CATCACCCC-GCT
JBRO111 .        GCCCGATAATTCTGCAGAGAATTTTCG-TGT-CGACAATTTTT----CATCACCCC-GAT
brunneoviridis4  GTCCGACAATTCTGAAGGGAATTTTCG-TGT-CGACAACTTTT----CATCTCCCC-GCT
brunneoviridis1  GTCCGACAATTCTGAAGGGAATTTTCG-TGT-CGACAACTTTT----CATCTCCCC-GCT
pleurotumCPK211  GCCCGACGATTCTGCAGAGAATTTTCG-TGT-CGACAATTGA-----TATCACCCC-GCC
PLEUROTICOLA .   XXXXXXCGA-------------TTCG-TGT-CGACA-TTTTTC-----TCACCCC-GCT
epimycesCPK1981  GCTCGATCATTTCGAAGAGAATTTTCG-TGT-CGATAATTCTT----CATCACCCC-GCT
alniCPK3124 ...  GCTCGATCATTCTGAAGAGAATCTTCG-TGT-CGACAATTTTT----CACCACCCC-GCT
CPK5TAWA .       GCTCGATCATTCTGAAGAGAATCCTCG-TGT-CGACAATTTTT---CCACCACCCC-GCT
2974lobau .      GCCCGACAATTCTGCAGAGAATTTTCG-TGT-CGCCAATTTTT----CATCATCCC-GCT
2975lobau .      GCCCGACAATTCTGCAGAGAATTTTCG-TGT-CGCCAATTTTT----CATCATCCC-GCT
aggressivumCPK3  GCTCGATCATTCTGAAGAGAAT------TGT-CGACAATTTTT----CATCACCCC-GCT
atrogelatinosaC  GCCCGACAATTCTGAAGAGAATTTTCG-TGC-CGACAATTTTT----CATCACCCC-GCT
```

```
atrogelatinosaC  GCCCGACAATTCTGAAGAGAATTTTCG-TGC-CGACAATTTTT----CATCACCCC-GCT
cerinumCPK293 .  GCTCGACAATTCTGAATAGAATTTTCG-TGT-CAACAATTTTT----CATCACCCC-GCT
cerinumCPK854 .  GCTCGACAATTCTGAATAGAATTTTCG-TGT-CAACAATTTTT----CATCACCCC-GCT
tomentosumCPK88  GCTCGACAATTCTGAATAGAATTTTCG-TGT-CAACAATTTTT----CACCACCCC-GCT
stramineaGJS028  GCCCGACAATTCTGAAAAGAATTTTCG-TGT-CAACAACTTTT----CGTCACCCC-GCT
velutinumCPK312  GCCCGACAATTCTGAAAAGAACTTTCG-TGT-CAATAATTTTT----CGTCACCCC-GCT
catoptronGJS027  ACCCGACAATTCTGAACAGAATTTTCA-TGT-CAAATTATTTTTTTCATCACCCC-GCT
                 ###########################     ###########    ######### ###


                          70        80        90       100       110       120
                 =========+=========+=========+=========+=========+=========+
1093CAN .        TTGCATTACCCCTCCTTTG-CAGCG-ACGCAAA---TTTTTTT----GGCTGTC-GTTTG
1052SA .         TTGCATTACCCCTCCTTTG-CAGCG-ACGCAAA---TTTTTTT----GGCTGTC-GTTTG
1116CAN .        TTGCATTACCCCTCCTTTG-CAGCG-ACGCAAA---TTTTTTT----GGCTGTC-GTTTG
1070EIRE .       TTGCATTACCCCTCCTTTG-CAGCG-ACGCAAA---TTTTTTT----GGCTGTC-GTTTG
1099CAN .        TTGCATTACCCCTCCTTTG-CAGCG-ACGCAAA---TTTTTTT----GGCTGTC-GTTTG
1104CAN .        TTGCATTACCCCTCCTTTG-CAGCG-ACGCAAA---TTTTTTT----GGCTGTC-GTTTG
1087CAN .        TTGCATTACCCCTCCTTTG-CAGCG-ACGCAAA---TTTTTTT----GGCTGTC-GTTTG
360UK .          TTGCATTACCCCTCCTTTG-CAGCG-ACGCAAA---TTTTTTT----GGCTGTC-GTTTG
2111HUN .        TTGCATTACCCCTCCTTTG-CAGCG-ACGCAAA---TTTTTTT----GGCTGTC-GTTTG
217RUS .         TTGCATTACCCCTCCTTTG-CAGCG-ACGCAAA---TTTTTTT----GGCTGTC-GTTTG
204UK .          TTGCATTACCCCTCCTTTG-CAGCG-ACGCAAA---TTTTTTT----GGCTGTC-GTTTG
206RUS .         TTGCATTACCCCTCCTTTG-CAGCG-ACGCAAA---TTTTTTT----GGCTGTC-GTTTG
261SIB .         TTGCATTACCCCTCCTTTG-CAGCG-ACGCAAA---TTTTTTT----GGCTGTC-GTTTG
265SIB .         TTGCATTACCCCTCCTTTG-CAGCG-ACGCAAA---TTTTTTT----GGCTGTC-GTTTG
2784THAI .       TTGCATTACCCCTCCTTTG-CAGCG-ACGCAAAAAATTTTT-------GCTGTC-GTTTG
838EGY .         TTGCATTACCCCTCCTTTG-CAGCG-ACGCAAAA--TTTTTT-----CGCTGTC-GTTTG
1102CAN .        TTGCATTACCCCTCCTTTG-CAGCG-ACGCAAAAA-TTTTTT------GCTGTC-GTTTG
588LAOS .        TTGCATTACCCCTCCTTTG-CAGCG-ACGCAAAA--TTTTTTT-----GCTGTC-GTTTG
1596AT .         TTGCATTACCCCTCCTTTG-CAGCG-ACGCAAAAA-TTTTTT------GCTGTC-GTTTG
1720CAM .        TTGCATTACCCCTCCTTTG-CAGCG-ACGCAAA---TTTATTTTT---GCTGTC-GTTTG
1722CAM .        TTGCATTACCCCTCCTTTG-CAGCG-ACGCAAA---TTTTTTTT----GCTGTC-GTTTG
1724CAM .        TTGCATTACCCCTCCTTTG-CAGCG-ACGCAAA---TTTTTTTT----GCTGTC-GTTTG
334USA .         TTGCATTACCCCTCCTTTG-CAGCG-ACGCAAAAA-TTTTTT------GCTGTC-GTTTG
335USA .         TTGCATTACCCCTCCTTTG-CAGCG-ACGCAAAA--TTTTTTT-----GCTGTC-GTTTG
1081WIS .        TTCCATTACCCCTCCTTTG-CAGCG-ACGCAAA---TTTTTTTTTTT-GCTGTC-GTTTG
1069WIS .        TTGCATTACCCCTCCTTTG-CAGCG-ACGCAAAAA-TTTTTT------GCTGTC-GTTTG
1107CAN .        TTGCATTACCCCTCCTTTG-GAGCG-ACGCAAAA--TTTTTTT-----GCTGTC-GTTTG
1108PEN .        TTGCATTACCCCTCCTTTG-GAGCG-ACGCAAAA--TTTTTTT-----GCTGTC-GTTTG
1110CAN .        TTGCATTACCCCTCCTTTG-CAGCG-ACGCAAAAA-TTTTTT------GCTGTC-GTTTG
JBSER241 .       TTCCATTACCCCTCCTTTG-CAGCG-ACGCAAA---TTTTTTTT----GCTGTC-GTTTG
246PHI .         TTCCATTACCCCTCCTTTG-CAGCG-ACGCAAA---TTTTTTTT----GCTGTC-GTXXX
51SA .           TTCCATTACCCCTCCTTTG-CAGCG-ACGCAAA---TTTTTTTT----GCTGTC-GTTTG
2618ETHI .       TTCCATTACCCCTCCTTTG-CAGCG-ACGCAAA---TTTTTTTT----GCTGTC-GTTTG
1068WIS .        TTGCATTACCCCTCCTTTG-CAGCG-ACGCAAAAA-TTTTTT------GCTGTC-GTTTG
238CR .          TTCCATTACCCCTCCTTTC-CAGCG-ACGCAAA---TTTTTTTTT----CTGTC-GTTTG
NR5555 .         TTCCATTACCCCTCCTTTC-CAGCG-ACGCAAA---TTTTTTTTT----CTGTC-GTTTG
NR6839 .         TTCCATTACCCCTCCTTTG-CAGCG-ACGCAAA---TTTTTTTT----GCTGTC-GTTTG
2624ETHI .       TTCCATTACCCCTCCTTTG-CAGCG-ACGCAAA---TTTTTTT----CCCTGTC-GTTTG
845EGY .         TTCCATTACCCCTCCTTTG-CAGCG-ACGCAAA---TTTTTTT----CCCTGTC-GTTTG
JBPER12 .        TTCCATTACCCCTCCTTTG-CAGCG-ACGCAAA---TTTTTTT-----GCTGTC-TTTTG
1717VIET .       TTCCATTACCCCTCCTTTG-CAGCG-ACGCAAA---TTTTTTT-----GCTATC-TTTTG
709BRAZ .        TTCCATTACCCCTCCTTTG-CAGCG-ACGCAAA---TTTTTTT-----GCTGTC-TTTTG
878IRAN .        TTCCATTACCCCTCCTTTG-CAGCG-ACGCAAA---TTTTTTTT----GCTGTC-TTTTG
239CR .          TTCCATTACCACTC---TG-CAGCG-ACGCAAA---TTTTTTT-----GCTGTC-TTTTG
202COM .         TTCCATTACCCCTCCTTTG-CAGCG-ACGCAAA---TTTTTTT-----GCTGTCTGT-TG
1064MEX .        TTCCATTACCCCTCCTTTG-CAGCG-ACGCAAA---TTTTTTT-----GCTGTC-TTTTG
596BRAZ .        TTCCATTACCCCTCCTTTG-CAGCG-ACGCAAA---TTTTTTT-----GCTGTC-TTTTG
333USA .         TTCCATTACCCCTCCTTTG-CAGCG-ACGCAAA---TTTTTTT-----GCTGTC-TTTTG
2610ETHI .       TTCCATTACCCCTCCTTTG-CAGCG-ACGCAAA---TTTTTTTT----GCTGCC-GTTTG
1044RW .         TTCCATTACCCCTCCTTTG-CAGCG-ACGCAAA---TTTTTTTT----GCTGCC-GTTTG
JBNZ111 .        TTCCATTACCCCTCCTTTG-CAGCG-ACGCAAA---TTTTTTT-----GCTGTC-TTTTG
JBNZ12 .         TTCCATTACCCCTCCTTTG-CAGCG-ACGCAAA---TTTTTTTTTT--GCTGCC-GTTTG
JBNZ24 .         TTCCATTACCCCTCCTTTG-CAGCG-ACGCAAA---TTTTTTT-----GCTGTC-TTTTG
693CHINA .       TTCCATTACCCCTCCTTTG-CAGCG-ACGCAAA---TTTTTTTT----GCTGCC-GTTTG
1058RW .         TTCCATTACCCCTCCTTTG-CAGCG-ACGCAAA---TTTTTTTT----GCTGCC-GTTTG
590AUS .         TTCCATTACCCCTCCTTTG-CAGCG-ACGCAAA---TTTTTTTT----GCTGCC-GTTTG
53SA .           TTCCATTACCCCTCCTTTG-CAGCG-ACGCAAA---TTTTTTTT----GCTGCC-GTTTG
1075WIS .        TTCCATTACCCCTCCTTTG-CAGCG-ACGCAAA---TTTTTTT-----GCTGTC-GTTTG
939GER .         TTCCATTACCCCTCCTTTG-CAGCG-ACGCAAA---TTTTTTT-----GCTGTC-GTTTG
GA3804 .         TTCCATTACCCCTCCTTTG-CAGCG-ACGCAAA---TTTTTTT-----GCTGTC-GTTTG
1599AT .         TTCCATTACCCCTCCTTTG-CAGCG-ACGCAAAAA-TTTTTTT-----GCTGTC-GTTTG
1941AT .         TTCCATTACCCCTCCTTTG-CAGCG-ACGCAAAAA-TTTTTTT-----GCTGTC-GTTTG
291NE .          TTCCATTACCCCTCCTTTG-CAGCG-ACGCAAA---TTTTTTTTT----CTGTC-GTTTG
2301SAR .        TTCCATTACCCCTCCTTTG-CAGCG-ACGCAAAA--TTTTTTTT-----CTGTC-GTTTG
2313SAR .        TTCCGTTACCCCTCCTTTG-CAGCG-ACGCAAAA--TTTTTTTT----GCTGTC-GTCTG
271NE .          TTCCATTACCCCTCCTTTG-CAGCG-ACGCAAA---TTTTTTTTT----CTGTC-GTTTG
1935GER .        TTCCATTACCCCTCCTTTG-CAGCG-ACGCAAAAA-TTTTTTT-----GCTGTC-GTTTG
JBRSA122 .       TTCCATTACCCCTCCTTTG-CAGCG-ACGCAAAAA-TTTTTTT------CTGTC-GTTTG
276NE .          TTCCATTACCCCTCCTTTG-CAGCG-ACGCAAA---TTTTTTTT----GCTGCC-GTTTG
272NE .          TTCCATTACCCCTCCTTTG-CAGCG-ACGCAAA---TTTTTTTT----GCTGCC-GTTTG
2673ETHI .       TTCCATTACCCCTCCTTTG-CAGCG-ACGCAAA---TTTTTTTTT---GCTGCC-GTTTG
1084AUSW .       TTCCGTTACCCCTCCTTTG-CAGCG-ACGCAAA---TTTTTTTTTTTTGCTGTC-GTTTG
JBPER62 .        TTCCATTACCCCTCGTTTG-CAGCG-ACGCAAA---TTTTGTTT----GCTGTC-GTTTC
JBNZ72 .         TTCCATTACCCCTCCTTTG-CAGCG-ACGCAAA---TTTTTTTTTT--GCTGCC-GTTTG
1065MEX .        TTCCATTACCCCTCCTTTG-CAGCG-ACGCAAA---TTTTTTT-----GCTGTC-TTTTG
646HAWAI .       TTCCATTACCCCTCCTTTG-CAGCG-ACGCAAA---TTTTTTT-----GCTGTC-TTTTG
727INDIA .       TTCCATTACCCCTCCTTTG-CAGCG-ACGCAAA---TTTTTTT-----GCTGTC-TTTTG
743MALE .        TTCCATTACCCCTCCTTTG-CAGCG-ACGCAAA---TTTTTTT-----GCTGTC-TTTTG
1059MEX .        TTCCATTACCCCTCCTTTG-CAGCG-ACGCAAA---TTTTTTTT----GCTGTC-TTTTG
1061RW .         TTCCATTACCCCTCCTTTC-CAGCG-ACGCAAA---TTTTTTTTT----CTGTC-GTTTG
1066MEX .        TTCCATTACCCCTCCTTTG-CAGCG-ACGCAAA---TTTTTTT-----GCTGTC-TTTTG
1095BALI .       TTGCATTACCCCTCCTTTG-CAGCG-ACGCAAA---TTTTTTT-----GCTGTC-GTTTG
1505MAUR .       TTCCATTACCCCTCCTTTG-CA----ACGCAAA---TTTTTTT----GGCTGTC-GTTTG
1818ETHI .       TTGCATTACCCCTCCTTTG-CAGCG-ACGCAAA---TTTTTTT----GGCTGTC-GTTTG
1934AT .         TTCCATTACCCCTCCTTTG-CAGCG-ACGCAAAAAATTTTTT------GCTGTC-GTTTG
245CR .          TTGCATTACCCCTCCTTTG-CAGCG-ACGCAAA---TTTTTTT-----GCTGTC-GTTTG
2710ETHI .       TTGCATTACCCCTCCTTTG-CAGCG-ACGCAAA---TTTTTTT-----GCTGTC-GTTTG
274NE .          TTGCATTACCCCTCCTTTG-CAGCG-ACGCAAA---TTTTTTT-----GCTGTC-GTTTG
3408PNG .        TTGCATTACCCCTCCTTTG-CAGCG-ACGCAAAA--TTTTTTT-----GCTGTC-GTTTG
3409PNG .        TTGCATTACCCCTCCTTTG-CAGCG-ACGCAAAA--TTTTTTT-----GCTGTC-GTTTG
410MALAY .       TTCCATTACCCCTCCTTTG-CAGCG-ACGCAAA---TTTTTTTT----GCTGTC-TTTTG
836EGY .         TTCCATTACCCCTCCTTTG-CA----ACGCAAA---TTTTTTT----GGCTGTC-GTTTG
837EGY .         TTCCATTACCCCTCCTTTG-CA----ACGCAAA---TTTTTTT----GGCTGTC-GT-TG
JBRO111 .        TTGCATTACCCCTCCTTTG-CAGCG-ACGCAAA---TTTTTTT----GGCTGTC-GTTTG
brunneoviridis4  TTCCGTTACCCCTCCTTTG-CAGC-AACGCAAAA--TTTTTTTT----GCTGTGCTTTTG
brunneoviridis1  TTCCGTTACCCCTCCTTTG-CAGC-AACGCAAAA--TTTTTTTT----GCTGTGCTTTTG
pleurotumCPK211  TTCCGTTACCCCTCCTTTGGCAGC-GACGCAAA---TTTTTTTT---GGCAGCC-GTTTG
PLEUROTICOLA .   TTCCGTTACCCCTCCTTTGGCAGC-GACGCAAA----TTTTTTGT--GGCAGCCTTTTGG
epimycesCPK1981  TTCCGTTACCCCTCCTTTG-CAGC-GACACAA----TTTTTTT-----GCTGTC-GTTGG
alniCPK3124 ...  TTGGGTTACCCCTCCTTTG-CAGC-GACGCAAA---TTTTTTT-----GCTGTC-GTTTG
CPK5TAWA .       TTCAATTACCCCTCCTTTG-CAGC-GACGCAAAAA-TTTTCT------GCTGTC-GTTTG
2974lobau .      TTGCATTACCCCTCCTTTG-CAGC-GACGCAAA---TTTTTCTTT---GCTGTC-GTTTG
2975lobau .      TTGCATTACCCCTCCTTTG-CAGC-GACGCAAA---TTTTTCTTT---GCTGTC-GTTTG
aggressivumCPK3  TTCCATTACCCCTCCTTTG-CAGC-GACACAAGA--TTTTTTTT----GCTGTC-GTTTG
atrogelatinosaC  TTCCGTTACCCCTCCTTTG-CAGC-GACGCA-GA--TTTTTCT-----GCTGTC-TTTTG
```

```
atrogelatinosaC  TTCCGTTACCCCTCCTTTG-CAGC-GACGCA-GA--TTTTTCT-----GCTGTC-TTTTG
cerinumCPK293 .  TTCCATTACCCCTCCTTTG-CAGC-GACGCAAA---TTTTTTTT----GCTGYCTTTTT-
cerinumCPK854 .  TTCCATTACCCCTCCTTTG-CAGCGGACGCAAA---TTTTTTTT----GCTGTCTTTTT-
tomentosumCPK88  TTCCATTACCCCTCCTTTG-CAGC-GACGCAAA---TTTTTTT-----GCTGTC-TTTTG
stramineaGJS028  TTCCATTACCCCTCCTTTG-CAGC-GACGCAAA---TTTTTTT-----GCTGTCA-TTTG
velutinumCPK312  TTCCATTACCCCTCCTTTG-CAGC-GACGCAAA---TTTTTTT-----GCTGTC-TTTTG
catoptronGJS027  TTCCATTACCCCTCCTTTG-CAGC-GACGCAAA---TTTTTTTT----GCAGT-ATTTTG
                 ################### ##### #######   #######     ###### #####


                        130       140       150       160       170       180
                 =========+=========+=========+=========+=========+=========+
1093CAN .        -GTTTT--AGTGGGGTTT-CTC--GTGCA-CCCC-ACTAGG-TCA-CT-GCTTTTTTT--
1052SA .         -GTTTT--AGTGGGGTTT-CTC--GTGCA-CCCC-ACTAGG-TCA-CT-GCTTTTTTT--
1116CAN .        -GTTTT--AGTGGGGTTT-CTC--GTGCA-CCCC-ACTAGG-TCA-CT-GCTTTTTTT--
1070EIRE .       -GTTTT--AGTGGGGTTT-CTC--GTGCA-CCCC-ACTAGG-TCA-CT-GCTTTTTTT--
1099CAN .        -GTTTT--AGTGGGGTTT-CTC--GTGCA-CCCC-ACTAGG-TCA-CT-GCTTTTTTT--
1104CAN .        -GTTTT--AGTGGGGTTT-CTC--GTGCA-CCCC-ACTAGG-TCA-CT-GCTTTTTTT--
1087CAN .        -GTTTT--AGTGGGGTTT-CTC--GTGCA-CCCC-ACTAGG-TCA-CT-GCTTTTTTT--
360UK .          -GTTTT--AGTGGGGTTT-CTC--GTGCA-CCCC-ACTAGG-TCA-CT-GCTTTTTTT--
2111HUN .        -GTTTT--AGTGGGGTTT-CTC--GTGCA-CCCC-ACTAGG-TCA-CT-GCTTTTTTT--
217RUS .         -GTTTT--AGTGGGGTTT-CTC--GTGCA-CCCC-ACTAGG-TCA-CT-GCTTTTTTT--
204UK .          -GTTTT--AGTGGGGTTT-CTC--GTGCA-CCCC-ACTAGG-TCA-CT-GCTTTTTTT--
206RUS .         -GTTTT--AGTGGGGTTT-CTC--GTGCA-CCCC-ACTAGG-TCA-CT-GCTTTTTTT--
261SIB .         -GTTTT--AGTGGGGTTT-CTC--GTGCA-CCCC-ACTAGG-TCA-CT-GCTTTTTTT--
265SIB .         -GTTTT--AGTGGGGTTT-CTC--GTGCA-CCCC-ACTAGG-TCA-CT-GCTTTTTTT--
2784THAI .       -GTTTT--AGTGGGGTTT-CTT--GTGCACCCCC-ACTAGC-TCA-CT-GCTTTTTTTTT
838EGY .         -GTTTT--AGTGGGGTTT-CT--TGTGCA-CCCCCACTAGC-TCA-CT-GCTTTTTTTTT
1102CAN .        -GTTTT-AAGTGGGGTTT-CT--TGTGCA-CCCC-ACTAGC-TC-----G-TTTTTT---
588LAOS .        -GTTTT--AGTGGGGTTT-CT--TGTGCA-CCCCCACTAGC-TCA-CT-GCTTTTTTTT-
1596AT .         -GTTTT-AAGTGGGGTTT-CT--TGTGCA-CCCC-ACTAGC-TC-----G-TTTTTT---
1720CAM .        -GTTTT--AGTGGGGTTT-CT--TGTGCA-CCCC-ACTAGC-TCA-CT-ACTTTTTTTT-
1722CAM .        -GTTTT--AGTGGGGTTT-CT--TGTGCA-CCCC-ACTAGC-TCA-CT-ACTTTTTTTT-
1724CAM .        -GTTTT--AGTGGGGTTT-CT--TGTGCA-CCCC-ACTAGC-TCA-CT-ACTTTTTTTT-
334USA .         -GTTTT--AGTGGGGTTT-CT--TGTGCA-CCCC-ACTAGC-TC-----G-TTTTTT---
335USA .         -GTTTT--AGTGGGGTTT-CT--TGTGCA-CCCC-ACTAGC-TC-----G-TTTTTT---
1081WIS .        -GTTTT--AGTGGGGTTT-CT----TGCA-CCCC-ACTAGC-TCA-CT-ACTTTTTTT--
1069WIS .        -GTTTT--AGTGGGGTTT-CT--TGTGCA-CCCC-ACTAGC-TC-----G-TTTTTT---
1107CAN .        -GTTTT--AGTGGGGTTTT-T--TGTGCA-CCCC-ACTAGCCTC-----G-TTTTTT---
1108PEN .        -GTTTT--AGTGGGGTTTT-T--TGTGCA-CCCC-ACTAGCCTC-----G-TTTTTT---
1110CAN .        -GTTTT-AAGTGGGGTTT-CT--TGTGCA-CCCC-ACTAGC-TC-----G-TTTTTT---
JBSER241 .       -GTTTTT-AGTGGGGTT--CTC-TGTGCAACCCC-ACTAGC-TCC-CT-GCTTTTT----
246PHI .         XXXXXXXXXXXXXXXXXXXXXXXXXXXXXXXXXXXXXXXXXXXXXXXXXXXXXXXXXXXX
51SA .           -GTTTTT-AGTGGGGTT--CTC-TGTGCAACCCC-ACTAGC-TCC-CT-GCTTTTT----
2618ETHI .       -GTTTTT-AGTGGGGTT--CTC-TGTGCAACCCC-ACTAGC-TCC-CT-GCTTTTT----
1068WIS .        -GTTTT--AGTGGGGTTT-CT--TGTGCA-CCCC-ACTAGC-TC-----G-TTTTTT---
238CR .          -GTTTTT-AGTGGGGTT--CTC-TGTGCAACCCC-ACTAGC-TCC-CT-GCTTTTT----
NR5555 .         -GTTTTT-AGTGGGGTT--CTC-TGTGCAACCCC-ACTAGC-TCC-CT-GCTTTTT----
NR6839 .         -GTTTTTTAGTGGGGTT--CTC-TGTGCAACCCC-ACTAGC-TCC-CT-GCTTTTT----
2624ETHI .       -GTTTTT-AGTGGGGTT--CTC-TGTGCAACCCC-ACTAGC-TCC-CT-GCTTTTT----
845EGY .         -GTTTTT-AGTGGGGTT--CTC-TGTGCAACCCC-ACTAGC-TCC-CT-GCTTTTT----
JBPER12 .        -GTTTT--AGTGGGGTTT-CT--TGTGCA-CCCC-ACTAGC-TCA-CT-GCTTTTTTTTT
1717VIET .       -GTTTT--AGTGGGGTTT-CT--TGTGCA-CCCC-ACTAGC-TCA-CT-GCTTTTTTTT-
709BRAZ .        -GTTTT--AGTGGGGTTT-CT--TGTGCA-CCCC-ACTAGC-TCA-CT-GCTTTTTTTTT
878IRAN .        -GTTTT--AGTGGGGTTT-CT--TGTGCA-CCCC-ACTAGC-TCA-CT-GCXXXXXXXXX
239CR .          -GTTTT--AGTGGGGTTT-CT--TGTGCA-CCCC-ACTAGC-TCA-CT-GCTTTTTTTTT
202COM .         -GTTTT--AGTGGGGTTT-CT--TGTGCA-CCCC-ACTAGC-TCA-CT-G-TATTTTT--
1064MEX .        -GTTTT--AGTGGGGTTT-CT--TGTGCA-CCCC-ACTAGC-TCA-CT-GCTTTTTTTTT
596BRAZ .        -GTTTT--AGTGGGGTTT-CT--TGTGCA-CCCC-ACTAGC-TCA-CT-GCTTTTTTTTT
333USA .         -GTTTT--AGTGGGGTTT-CT--TGTGCA-CCCC-ACTAGC-TCA-CT-GCTTTTTTTTT
2610ETHI .       A-TTTTT-AGTGGGGTT--CTC-TGTGCAACCCC-ACTAGC-TCA-CT-GCTTTTTT---
1044RW .         A-TTTTT-AGTGGGGTT--CTC-TGTGCAACCCC-ACTAGC-TCA-CT-GCTTTTTT---
JBNZ111 .        -GCTTT--AGTGGGGTTTT-T--TGTGCA-CCCC-ACTAGC-TCA-CT-GCATT------
JBNZ12 .         A-TTTTT-AGTGGGGTT--CTC-TGTGCAACCCC-ACTAGC-TCA-CT-GCTTTTTTTT-
JBNZ24 .         -GCTTT--AGTGGGGTTT--T-TTGTGCA-CCCC-ACTAGC-TCA-CT-GCATT------
693CHINA .       AGTTTT--AGTGGGGTT--CTC-TGTGCAACCCC-ACTAGC-TCA-CT-GCTTTTTT---
1058RW .         A-TTTTT-AGTGGGGTT--CTC-TGTGCAACCCC-ACTAGC-TCA-CT-GCTTTTTT---
590AUS .         A-TTTTT-AGTGGGGTT--CTC-TGTGCAACCCC-ACTAGC-TCA-CT-GCTTTTTT---
53SA .           A-TTTTT-AGTGGGGTT--CTC-TGTGCAACCCC-ACTAGC-TCA-CT-GCTTTTTT---
1075WIS .        -GTTTTT-AGTGGGGTT--CTC-TGTGCAACCCC-ACTAGC-TCA-CT-GCTTTTT----
939GER .         -GTTTTT-AGTGGGGTT--CTC-TGTGCAACCCC-ACTAGC-TCA-CT-GCTTTTT----
GA3804 .         -GTTTTT-AGTGGGGTT--CTC-TGTGCAACCCC-ACTAGC-TCA-CT-GCTTTTT----
1599AT .         -GTTTTT-AGTGGGGTT--CTC-TGTGCAACCCC-ACTAGC-TCA-CT-GCTTTTT----
1941AT .         -GTTTTT-AGTGGGGTT--CTC-TGTGCAACCCC-ACTAGC-TCA-CT-GCTTTTT----
291NE .          -GTTTTT-AGTGGGGTT--CTC-TGTGCAACCCC-ACTAGC-TCA-CT-GCTTTTT----
2301SAR .        -GTTTTT-AGTGGGGTT--CTC-TGTGCAACCCC-ACTAGC-TCA-CT-GCTTTTT----
2313SAR .        -GTTTTT-AGTGGGGTT--CTC-TGTGCAACCCC-ACTAGC-TCA-CT-GCTTTTT----
271NE .          -GTTTTT-AGTGGGGTT--CTC-TGTGCAACCCC-ACTAGC-TCA-CT-GCTTTTT----
1935GER .        -GTTTTT-AGTGGGGTT--CTC-TGTGCAACCCC-ACTAGC-TCA-CT-GCTTTTT----
JBRSA122 .       -GTTTTT-AGTGGGGTT--CTC-TGTGCAACCCC-ACTAGC-TCA-CT-GCTTTTT----
276NE .          A-TTTTT-AGTGGGGTT--CT-TTGTGCAACCCC-ACTAGC-TCA-CT-GCTTTTTTT--
272NE .          A-TTTTT-AGTGGGGTT--CT-TTGTGCAACCCC-ACTAGC-TCA-CT-GCTTTTTTT--
2673ETHI .       A-TTTTT-AGTGGGGTT--CTT-TGTGCAACCCC-ACTAGC-TCA-CT-GCTTTTTTT--
1084AUSW .       A-TTTT--AGTGGGGTTT-CT--TGTGCA-CCCC-ACTAGC-TCA-CT-GCATTTTTTT-
JBPER62 .        -GTTTT--AGTGGGGTTT-CT--TGTGCA-CCCC-ACTAGC-TCA-CT-GCTTTTTTT--
JBNZ72 .         A-TTTTT-AGTGGGGTT--CTC-TGTGCAACCCC-ACTAGC-TCA-CT-GCTTTTTTTT-
1065MEX .        -GTTTT--AGTGGGGTTT-CT--TGTGCA-CCCC-ACTAGC-TCA-CT-GCTTTTTTTTT
646HAWAI .       -GTTTT--AGTGGGGTTT-CT--TGTGCA-CCCC-ACTAGC-TCG-CT-GCTTTTTTTTT
727INDIA .       -GTTTT--AGTGGGGTTT-CT--TGTGCA-CCCC-ACTAGC-TCA-CT-GCTTTTTTTTT
743MALE .        -GTTTT--AGTGGGGTTT-CT--TGTGCA-CCCC-ACTAGC-TCA-CT-GCTTTTTTTTT
1059MEX .        -GTTTT--AGTGGGGTTT-CT--TGTGCA-CCCC-ACTAGC-TCA-CT-GCTTTTTTTTT
1061RW .         -GTTTTT-AGTGGGGTT--CTC-TGTGCAACCCC-ACTAGC-TCC-CT-GCTTTTT----
1066MEX .        -GTTTT--AGTGGGGTTT-CT--TGTGCA-CCCC-ACTAGC-TCA-CT-GCTTTTTTTTT
1095BALI .       -GTTTT--AGTGGGGTTT-CT--TGTGCA-CCCC-ACTAGC-TCA-CT-A-ATTTTTTT-
1505MAUR .       A-TTTT--AGTGGGGTTT-CT--TGTGCA-CCCC-ACTAGC-TCA-C----TTTTTTT--
1818ETHI .       -GTTTT--AGTGGGGTTT-CTC--GTGCA-CCCC-ACTAGG-TCA-CT-GCTTTTTTT--
1934AT .         -GTTTTT-AGTGGGGTT--CTC-TGTGCAACCCC-ACTAGC-TCA-CT-GCTTTTT----
245CR .          -GTTTT--AGTGGGGTTT-CT--TGTGCA-CCCC-ACTAGC-TCA-CT-ACTTTTTTT--
2710ETHI .       -GTTTT--AGTGGGGTTT-CT--TGTGCA-CCCC-ACTAGC-TCA-CT-ACTTTTTTT--
274NE .          -GTTTT--AGTGGGGTTT-CT--TGTGCA-CCCC-ACTAGC-TCA-CT-ACTTTTTTT--
3408PNG .        -GTTTT--AGTGGGGTTT-CT--TGTGCA-CCCCCACTAGC-TCA--TGGCTTTTTTT--
3409PNG .        -GTTTT--AGTGGGGTTT-CT--TGTGCA-CCCCCACTAGC-TCA--TGGCTTTTTTT--
410MALAY .       -GTTTT--AGTGGGGTT-GCT--GGTGCA-CCCC-ACTAGC-TCA-CT-GCTTTTTTT--
836EGY .         -GTTTT--AGTGGGGTTT-CT--TGTGCA-CCCC-ACTAGC-TCA-C----TTTTTTT--
837EGY .         -GTTTT--AGTGGGGTTT-CT--TGTGCA-CCCC-ACTAGC-TCA-CT---TTTTTT---
JBRO111 .        -GTTTT--AGTGGGGTTT-CTC--GTGCA-CCCC-ACTAGG-TCA-CT-GCTTTTTTT--
brunneoviridis4  -GTTTT--AGTGGAGTTT-CT--TGTGCA-CCCC-ACTAGC-TCA-CT-GCTTTTTT---
brunneoviridis1  -GTTTT--AGTGGAGTTT-CT--TGTGCA-CCCC-ACTAGC-TCA-CT-GCTTTTTT---
pleurotumCPK211  -GCTTT--GGTGGGGTTT--T-GTGTGCA-CCCC-ACTAGC-TCG-CT-GCTTTTTTTTT
PLEUROTICOLA .   --CTTT--GGTGGGGTTT-C--GTGTGCA-CCCC-ACTAGC-TCA-CT-GCTTTTTT---
epimycesCPK1981  -GTTTT--AGTGGGGTTCCC---TGTGCA-CCCC-ACTAGC-TCA-CT-GCCTTTTTTT-
alniCPK3124 ...  -GTTTT--AGTGGGGT-CCCT--TGTGTA-CCCC-ACTAGC-TCA-CT-GCTTTTTTT--
CPK5TAWA .       GGTTT---AGTGGGGTT-CCT--TGTGCA-CCCC-ACTAGC-TCA-CT-GCTTTTTTT--
2974lobau .      -GTTTT--AGTGGGGTTTC-T--TGTGCA-CCCC-ACTAGC-TCA--TGGCTTTTTTT--
2975lobau .      -GTTTT--AGTGGGGTTTC-T--TGTGCA-CCCC-ACTAGC-TCA--TGGCTTTTTTT--
aggressivumCPK3  -GTTTT--AGTGGGGTT-CCT--TGTGCA-CCCCCACTAGC-TCA-CT-GCTTTTTTTTT
atrogelatinosaC  -GTTTC--AGTGGGGTT-CC-C-TGCACA-CCCC-ACTGGC--CAGCT-GCTTTTT----
```

```
atrogelatinosaC  -GTTTC--AGTGGGGTT-CC-C-TGCACA-CCCC-ACTGGC--CAGCT-GCTTTTT----
cerinumCPK293 .  -GTTTT--AGTGGGGTT--CTC-TGCGTA-CCCC-ACTAAC-TCA-CT-GCTTTTTTT--
cerinumCPK854 .  -GTTTT--AGTGGGGTT--CTC-TGCGTA-CCCC-ACTAAC-TGA-CT-G-TTTTTT---
tomentosumCPK88  A-TTTT--AGTGGGGTT--CTC-TGTGTA-CCCC-ACTAGC-TCA-CT-GCTTTTT----
stramineaGJS028  -GTTTT--AGTGGGGTTTCC---TGCGCA-CCCC-ACTAG--TCAACT-GCTTTTTT---
velutinumCPK312  -GTTTT--AGTGGGGTT-CC-C-TGCGCA-CCCC-ACTAGC-T-AACT-GC-TGTT----
catoptronGJS027  A-TTTT--AGTGGGGTT-GC-C-TCTGCA-CCCC-ACTAAC-TTG-CT-ACTTGTTT---
                  #####  ##########     ######      ######        #########


                        190       200       210       220       230       240
                 =========+=========+=========+=========+=========+=========+
1093CAN .        ----------CT--------GCTTCGCT-C-T-TACTG-CCCAGCCATCATTCAACGTGC
1052SA .         ----------CT--------GCTTCGCT-C-T-TACTG-CCCAGCCATCATTCAACGTGC
1116CAN .        ----------CT--------GCTTCGCT-C-T-TACTG-CCCAGCCATCATTCAACGTGC
1070EIRE .       ----------CT--------GCTTCGCT-C-T-TACTG-CCCAGCCATCATTCAACGTGC
1099CAN .        ----------CT--------GCTTCGCT-C-T-TACTG-CCCAGCCATCATTCAACGTGC
1104CAN .        ----------CT--------GCTTCGCT-C-T-TACTG-CCCAGCCATCATTCAACGTGC
1087CAN .        ----------CT--------GCTTCGCT-C-T-TACTG-CCCAGCCATCATTCAACGTGC
360UK .          ----------CT--------GCTTCGCT-C-T-TACTG-CCCAGCCATCATTCAACGTGC
2111HUN .        ----------CT--------GCTTCGCT-C-T-TACTG-CCCAGCCATCATTCAACGTGC
217RUS .         ----------CT--------GCTTCGCT-C-T-TACTG-CCCAGCCATCATTCAACGTGC
204UK .          ----------CT--------GCTTCGCT-C-T-TACTG-CCCAGCCATCATTCAACGTGC
206RUS .         ----------CT--------GCTTCGCT-C-T-TACTG-CCCAGCCATCATTCAACGTGC
261SIB .         ----------CT--------GCTTCGCT-C-T-TACTG-CCCAGCCATCATTCAACGTGC
265SIB .         ----------CT--------GCTTCGCT-C-T-TACTG-CCCAGCCATCATTCAACGTGC
2784THAI .       TT--------CT--------GCTTCGCT-C-T-CACTT-CCCAGCCATCATTCAACGTGT
838EGY .         ----------CT--------GCTTCGCT-C-T-CACTT-CCCGGCCATCATTCAACGTGC
1102CAN .        ----------CT--------GCTTCGCT-C-T-CACTT-CCCAGCCATCATTCAACGTAT
588LAOS .        ----------CT--------GCTTCGCT-C-T-CACTT-CCCAGCCATCATTCAACGTGT
1596AT .         ----------CT--------GCTTCGCT-C-T-CACTT-CCCAGCCATCATTCAACGTAT
1720CAM .        ----------CT--------GCTTCGCT-C-T-CACTT-CCCAGCCATCATTCAACGTGC
1722CAM .        ----------CT--------GCTTCGCT-C-T-CACTT-CCCAGCCATCATTCAACGTGC
1724CAM .        ----------CT--------GCTTCGCT-C-T-CACTT-CCCAGCCATCATTCAACGTGC
334USA .         ----------CT--------GCTTCGCT-C-T-CACTT-CCCAGCCATCATTCAACGTAT
335USA .         ----------CT--------GCTTCGCT-C-T-CACTT-CCCAGCCATCATTCAACGTAT
1081WIS .        ----------CT--------ACTTTGCT-C-T-CACTT-CCCAGCCATCATTCAACATGT
1069WIS .        ----------CT--------GCTTCGCT-C-T-CACTT-CCCAGCCATCATTCAACGTAT
1107CAN .        ----------CT--------GCTTCGCT-C-T-CACTT-CCCAGCCATCATTCAACGTGC
1108PEN .        ----------CT--------GCTTCGCT-C-T-CACTT-CCCAGCCATCATTCAACGTGC
1110CAN .        ----------CT--------GCTTCGCT-C-T-CACTT-CCCAGCCATCATTCAACGTAT
JBSER241 .       ---------CCT--------GCTTCACT-C-T-CACTT-CCTCGTCATCATTCAACGTGC
246PHI .         XXXXXXXXXXXXXXXXXXXXXXXXXXXXXXXXXXXXXXXXXXXXXXXXXXXXXXXXXXXX
51SA .           ---------CCT--------GCTTCACT-C-T-CACTT-CCTCGTCATCATTCAACGTGC
2618ETHI .       ---------CCT--------GCTTCACT-C-T-CACTT-CCTCGTCATCATTCAACGTGC
1068WIS .        ----------CT--------GCTTCGCT-C-T-CACTT-CCCAGCCATCATTCAACGTAT
238CR .          ---------CCT--------GCTTCACT-C-T-CACTT-CCTCGTCATCATTCAACGTGC
NR5555 .         ---------CCT--------GCTTCACT-C-T-CACTT-CCTCGTCATCATTCAACGTGC
NR6839 .         ---------CCT--------GCTTCACT-C-T-CAGTT-CTTCGTCATCATTCAACGTGC
2624ETHI .       ---------CCT--------GCTTCACT-C-T-CAGTT-CTTCGTCATCATTCAACGTGC
845EGY .         ---------CCT--------GCTTCACT-C-T-CAGTT-CTTCGTCATCATTCAACGTGC
JBPER12 .        TTT--------TG-------GCTTCACT-C-T-CACTT-CCCCGCCAT---TCAACGTAC
1717VIET .       ---------CCT-TTTTT--GCTTCACT-C-T-CACTT-CCCAGCCAT---TCAACGTGC
709BRAZ .        TTTTT------TG-------GCTTCACT-C-T-CACTT-CCCCGCCAT---TAAACGTAC
878IRAN .        XXXXXXXXXXXXXXXXXXXXXXXXXXXXXXXXXXXXXXXXXXXXXXXXXXXXXXXXXXXX
239CR .          TTTTTTTTTTTTTTTXXXXXXXXXXXXXXXXXXXXXXXXXXXXXXXXXXXXXXXXXXXXX
202COM .         ----------CT--------GCTTCACT-C----ACTT-CCCAGCCATCATTCAGCGTGT
1064MEX .        TTTTTTT----TG-------GCTTCACT-C-T-CACTT-CCCCGCCAT---TCAACGTAC
596BRAZ .        TTT--------TG-------GCTTCACT-C-T-CACTT-CCCCGCCAT---TAAACGTCC
333USA .         TTTXXXXXXXXXXXXXXXXXXXXXXXXXXXXXXXXXXXXXXXXXXXXXXXXXXXXXXXXX
2610ETHI .       -----------TGT------GCTTCA-T---T-CACTT-CCCAGTCATCATTCAACGTGC
1044RW .         -----------TGT------GCTTCA-T---T-CACTT-CCCAGTCATCATTCAACGTGC
JBNZ111 .        -----------TGTTTTTTTGCTTCACT-C-T-CACTT-CCTAGCCAT---TCAACGTGC
JBNZ12 .         -----------TGT------GCTTCACT-----CACTT-CCCAGTCATCATTCAACGTGC
JBNZ24 .         -----------TGTTTTTTTGCTTCACT-C-T-CACTT-CCTAGCCAT---TCAACGTGC
693CHINA .       -----------TGT------GCTTCACA-C-T-CACTT-CCAAGTCATCATTCAACGTGC
1058RW .         -----------TGT------GCTTCA-T---T-CACTT-CCCAGTCATCATTCAACGTGC
590AUS .         -----------TGT------GCTTCACT-----CACTT-CCCAGTCATCATTCAACGTGC
53SA .           -----------TGT------GCTTCA-T---T-CACTT-CCCAGTCATCATTCAACGTGC
1075WIS .        ---------CCT--------GCTTCACT-C-T-CACTT-CCTCATCATCATTCAACACGC
939GER .         ---------CCT--------GCTTCACT-C-T-CACTT-CCTCATCATCATTCAACACGC
GA3804 .         ---------CCT--------GCTTCACT-C-T-CACTT-CCTCATCATCATTCAACACGC
1599AT .         ---------CCT--------GCTTCACT-C-T-CACTT-CCTAGTCATCATTCAACACGC
1941AT .         ---------CCT--------GCTTCACT-C-T-CACTT-CCTCGTCATCATTCAACACGC
291NE .          ---------CCT--------GCTTCACT-C-T-CACTT-CCTCGTCATCATTCAACACGC
2301SAR .        ---------CCT--------GCTTCACT-C-T-CACTT-CCTCGTCATCATTCAACACGC
2313SAR .        ---------CCT--------GCTTCACT-C-T-CACTT-CCTCGTCATCATTCAACACGC
271NE .          ---------CCT--------GCTTCACT-C-T-CACTT-CCTCGTCATCATTCAACACGC
1935GER .        ---------CCT--------GCTTCACT-C-T-CACTT-CCTCGTCATCATTCAACACGC
JBRSA122 .       ---------CCT--------GCTTCACT-C-T-CACTT-CCTCGTCATCATTCAACACGC
276NE .          -----------TGT------GCTTCACT-C-T-CACTT-CCCAGCCATCATTCAACGTGC
272NE .          -----------TGT------GCTTCACT-C-T-CACTT-CCCAGCCATCATTCAACGTGC
2673ETHI .       -----------TGT------GCTTCACT-C-T-CACTT-CCCAGCCATCATTCAACGTGC
1084AUSW .       ----------C---------GCTTCACT-C-T-CACTT-CCCAGCCAT---TCAACGTGC
JBPER62 .        ---------CCT--------GCTTCACT-C-T-CACTT-CCCAGCCATCATTCAACGGGC
JBNZ72 .         -----------TGT------GCTTCACT-----CACTT-CCCAGTCATCATTCAACGTGC
1065MEX .        -----------TG-------GCTTCACT-C-T-CACTT-CCCCGCCAT---TCAACGTAC
646HAWAI .       TTT--------TG-G-----GTTTCCCT-C-T-CXXXXXXXXXXXXXXXXXXXXXXXXXX
727INDIA .       TTTTTTTTTTTTG-G-----GTTTCCXXXXXXXXXXXXXXXXXXXXXXXXXXXXXXXXXX
743MALE .        TTT--------TG-G-----GTTTCCXXXXXXXXXXXXXXXXXXXXXXXXXXXXXXXXXX
1059MEX .        TTTT-------TG-------GCTTCACT-C-T-CACTT-CCCCGCCAT---TCAACGTAC
1061RW .         ---------CCT--------GCTTCACT-C-T-CACTT-CCTCGTCATCATTCAACGTGC
1066MEX .        TT---------TG-------GCTTCACT-C-T-CACTT-CCCCGCCAT---TCAACGTAC
1095BALI .       ----------CT--------GCTTCGCT-C-T-CACTT-CCCAGCCATCATTCAACGTGC
1505MAUR .       -------CCCCT--------GCTTCACT-C-T-CACTT-CCCAGCCATCATTCAACGTGT
1818ETHI .       ----------CT--------GCTTCGCT-C-T-TACTG-CCCAGCCATCATTCAACGTGC
1934AT .         ---------CCT--------GCTTCACT-C-T-CACTT-CCTAGTCATCATTCAACACGC
245CR .          ----------CT--------GCTTCGCT-C-T-CACTT-CCCAGCCATCATTCAACGTGC
2710ETHI .       ----------CT--------GCTTCGCT-C-T-CACTT-CCCAGCCATCATTCAACGTGC
274NE .          ----------CT--------GCTTCGCT-C-T-CACTT-CCCAGCCATCATTCAACGTGC
3408PNG .        ----------CT--------GCTTCGCT-C-T-CACTT-CCCAACCATCATTCAACGTGC
3409PNG .        ----------CT--------GCTTCGCT-C-T-CACTT-CCCAACCATCATTCAACGTGC
410MALAY .       ----------C----------CTTCAC------CACTG-CC-AGTCATCATTCAACGATC
836EGY .         -------CCCCT--------GCTTCACT-C-T-CACTT-CCCAGCCATCATTCAACGTGT
837EGY .         -------CCCCT--------GCTTCACT-C-T-CACTT-CCCAGCCATCATTCAACGTGT
JBRO111 .        ----------CT--------GCTTCGCT-C-T-TACTG-CCCAGCCATCATTCAACGTGC
brunneoviridis4  ------------GT------GCTTGACTCT---CACTTCGCCAGTCATCATTCAACGTGT
brunneoviridis1  ------------GT------GCTTGACTCT---CACTTCGCCAGTCATCATTCAACGTGT
pleurotumCPK211  ----------CT--------GCTTCACTCCCCCACTGGCCAGTCATGATTCAACGTGC
PLEUROTICOLA .   ----------CT--------GCTTCACTCTC-CCACTG-CCCAGTCATCATTCAACGTGC
epimycesCPK1981  ------------GT------GCTTCACTCTC---ACTG-CCCAGCCGTCGTTCAACGTGC
alniCPK3124 ...  ------------GT------GCTTCACTCTC---ACTG-CTCAGCCATCATTCAGCGCGC
CPK5TAWA .       ------------GG------GCTTCACTTTC---ACTG-GCCCGTCGTCATTCAACGCAC
2974lobau .      ----------CT--------GCTTCGCTCTC---ACTT-CCCAGCCATCATTCAACGTGC
2975lobau .      ----------CT--------GCTTCGCTCTC---ACTT-CCCAGCCATCATTCAACGTGC
aggressivumCPK3  ------------GT------GCTTCACTATC---ACTA-CCCAGCCGTCGTTCAACGTGC
atrogelatinosaC  ------------GT------GCTTCACTGTC---ACTG-TTCAGTCGTCATTCAACGTGC
```

```
atrogelatinosaC  ------------GT------GCTTCACTGTC---ACTG-TTCAGTCGTCATTCAACGTGC
cerinumCPK293 .  ----------CT--------GTTTCGCTCTC---ACTA-CCCAGTCGTCATTCAACGCGC
cerinumCPK854 .  ----------CT--------GTTTCGCTCTC---ACTA-CCCAGGCATCATTCAACGCGC
tomentosumCPK88  ----------CT--------GTTTTGCTCTC---GCTA-CCCAGTCGTCATTCAACGCGC
stramineaGJS028  ------------GT------GCTTCACTTTC---ACTG-CCCAGTCATCATTCAACGTGC
velutinumCPK312  ----------CTGT------GCTTCACTC-----ACTG-CCTAGCCAACTTTCAACGTGC
catoptronGJS027  -------------------ACCTTCACAATC---ACTG-CACAGTCGGCATTCAACGGTC
                                     ########      ##### ####################


                        250       260       270       280       290       300
                 =========+=========+=========+=========+=========+=========+
1093CAN .        T--CTGCGT-CT--CATCACTTTCAGCGATGCTAACCAC--TTTT-CCATCAATAGGAAG
1052SA .         T--CTGCGT-CT--CATCACTTTCAGCGATGCTAACCAC--TTTT-CCATTAATAGGAAG
1116CAN .        T--CTGCGT-CT--CATCACTTTCAGCGATGCTAACCAC--TTTT-CCATCAATAGGAAG
1070EIRE .       T--CTGCGT-CT--CATCACTTTCAGCGATGCTAACCAC--TTTT-CCATCAATAGGAAG
1099CAN .        T--CTGCGT-CT--CATCACTTTCAGCGATGCTAACCAC--TTTT-CCATCAATAGGAAG
1104CAN .        T--CTGCGT-CT--CATCACTTTCAGCGATGCTAACCAC--TTTT-CCATCAATAGGAAG
1087CAN .        T--CTGCGT-CT--CATCACTTTCAGCGATGCTAACCAC--TTTT-CCATCAATAGGAAG
360UK .          T--CTGCGT-CT--CATCACTTTCAGCGATGCTAACCAC--TTTT-CCATCAATAGGAAG
2111HUN .        T--CTGCGT-CT--CATCACTTTCAGCGATGCTAACCAC--TTTT-CCATTAATAGGAAG
217RUS .         T--CTGCGT-CT--CATCACTTTCAGCGATGCTAACCAC--TTTT-CCATCAATAGGAAG
204UK .          T--CTGCGT-CT--CATCACTTTCAGCGATGCTAACCAC--TTTT-CCATCAATAGGAAG
206RUS .         T--CTGCGT-CT--CATCACTTTCAGCGATGCTAACCAC--TTTT-CCATCAATAGGAAG
261SIB .         T--CTGCGT-CT--CATCACTTTCAGCGATGCTAACCAC--TTTT-CCATCAATAGGAAG
265SIB .         T--CTGCGT-CT--CATCACTTTCAGCGATGCTAACCAC--TTTT-CCATCAATAGGAAG
2784THAI .       T--CTGTGT-C---CGTCAC------------TAACCAC--TTTT-CCATCAATAGGAAG
838EGY .         T--CTGTGT--TT-CGTCACTTTCAGCGATGCTAACCGC--TTTT-CTATCAATAGGAAG
1102CAN .        T--CTGTGT-CT--CGTCACTTTCAGCGATGCTAACCAC--TTTT-CCATCAATAGGAAG
588LAOS .        T--CTGTGT-CT--CGTCACTTTCAGCGATGCTAACCAC--TTTT-CCATCAATAGGAAG
1596AT .         T--CTGTGT-CT--CGTCACTTTCAGCGATGCTAACCAC--TTTT-CCATCAATAGGAAG
1720CAM .        T--CCGTGT-----CATCACTTTCAGCGATGCTAACCAC--TTTT-CCATCAATAGGAAG
1722CAM .        T--CCGTGT-----CATCACTTTCAGCGATGCTAACCAC--TTTT-CCATCAATAGGAAG
1724CAM .        T--CCGTGT-----CATCACTTTCAGCGATGCTAACCAC--TTTT-CCATCAATAGGAAG
334USA .         T--CTGTGT-CT--CGTCACTTTCAGCGATGCTAACCAC--TTTT-CCATCAATAGGAAG
335USA .         T--CTGTGT-CT--CGTCACTTTCAGCGATGCTAACCAC--TTTT-CCATCAATAGGAAG
1081WIS .        T--CTATGT-CT--CGTCACTTTCAACGATGCTAACCACAATTT--CCATCAATAGGAAG
1069WIS .        T--CTGTGT-CT--CGTCACTTTCAGCGATGCTAACCAC--TTTT-CCATCAATAGGAAG
1107CAN .        T--CTGTGT-----CATCACTTTCAGCGATGCTAACCAC--TTTT-CCATCAATAGGAAG
1108PEN .        T--CTGTGT-----CATCACTTTCAGCGATGCTAACCAC--TTTT-CCATCAATAGGAAG
1110CAN .        T--CTGTGT-CT--CGTCACTTTCAGCGATGCTAACCAC--TTTT-CCATCAATAGGAAG
JBSER241 .       T--CTGCGT-CTTTGGTCA--TTCAGCGACGCTAACCAC--TTTT-ACATCAATAGGAAG
246PHI .         XXXXXXXXXXXXXXXXXXXXXXXXXXXXXXXXXXXXXXXXXXXXXXXXXXXXXXXXXXXX
51SA .           T--CTGCGT-CTTTGGTCA--TTCAGCGACGCTAACCAC--TTTT-ACATCAATAGGAAG
2618ETHI .       T--CTGCGT-CTTTGGTCA--TTCAGCGACGCTAACCAC--TTTT-ACATCAATAGGAAG
1068WIS .        T--CTGTGT-CT--CGTCACTTTCAGCGATGCTAACCAC--TTTT-CCATCAATAGGAAG
238CR .          T--CTGCGT-CTTTGGTCA--TTCAGCGACGCTAACCAC--TTTT-CCATCAATAGGAAG
NR5555 .         T--CTGCGT-CTTTGGTCA--TTCAGCGACGCTAACCAC--TTTT-CCATCAATAGGAAG
NR6839 .         T--CTGCGT-CTTTGGTCA--TTCAGCGATGCTAACCAC--TTTT-CCATCAATAGGAAG
2624ETHI .       T--CTGCGT-CTTTGGTCA--TTCAGCGATGCTAACCAC--TTTT-CCATCAATAGGAAG
845EGY .         T--CTGCGT-CTTTGGTCA--TTCAGCGATGCTAACCAC--TTTT-CCATCAATAGGAAG
JBPER12 .        T--CTGTGT-CTTTGGTCA--TTCAGAGATGCTAACCAC--TTTT-CCGTCAATAGGAAG
1717VIET .       T--CTGCGT-CTTTGGTCA--TTGAGCGATGCTAACCAC--TTTT-CCATCAATAGGAAG
709BRAZ .        T--CTGTGT-CTTTGGTCA--TTCAGCGATGCTAACCAC--TTTT-CCGTCAATAGGAAG
878IRAN .        XXXXXXXXXXXXXXXXXXXXXXXXXXXXXXXXXXXXXXXXXXXXXXXXXXXXXXXXXXXX
239CR .          XXXXXXXXXXXXXXXXXXXXXXXXXXXXXXXXXXXXXXXXXXXXXXXXXXXXXXXXXXXX
202COM .         T--CTGTGTCCTT-GGTCA--TTCAGCGATGCTAACCAC--TTTT-CCATCAATAGGAAG
1064MEX .        T--CTGTGT-CTTTGGTCA--TTCAGCGATGCTAACCAC--TTTT-CCATCAATAGGAAG
596BRAZ .        T--CTGTGT--TTTGGTCA--TTCAGCGATGCTAACCAC--TTTT-CCATCAATAGGAAG
333USA .         XXXXXXXXXXXXXXXXXXXXXXXXXXXXXXXXXXXXXXXXXXXXXXXXXXXXXXXXXXXX
2610ETHI .       T--CTGTGT-CTTTGGTTA--TTCAACGATGCTAACCAC--TTTT-CCATCAATAGGAAG
1044RW .         T--CTGTGT-CTTTGGTTA--TTCAACGATGCTAACCAC--TTTT-CCATCAATAGGAAG
JBNZ111 .        T--CTGCGT-CTTTGGTCA-TTT-AGCGATGCTAACCAC--TTTT-CCACCAACAGGAAG
JBNZ12 .         T--CTGTGT-CTTTGATCA--TTCAACGATGCTAACCAC--TTTT-CCATCAATAGGAAG
JBNZ24 .         T--CTGCGT-CTTTGGTCA-TTT-AGCGATGCTAACCAC--TTTT-CCACCAACAGGAAG
693CHINA .       T--CTGTGT-CTTTGGTCA--TTCAACGATGCTAACCAC--TTTT-CCATCAATAGGAAG
1058RW .         T--CTGTGT-CTTTGGTTA--TTCAACGATGCTAACCAC--TTTT-CCATCAATAGGAAG
590AUS .         T--CTGTGT-CTTTGGTCA--TTCAACGATGCTAACCAC--TTTT-CCATCAATAGGAAG
53SA .           T--CTGTGT-CTTTGGTTA--TTCAACGATGCTAACCAC--TTTT-CCATCAATAGGAAG
1075WIS .        T--CTGTGT-CTTTGGTCA--TTCAGCGATGCTAACCAC--TTTT-CCATCAATAGGAAG
939GER .         T--CTGTGT-CTTTGGTCA--TTCAGCGATGCTAACCAC--TTTT-CCATCAATAGGAAG
GA3804 .         T--CTGTGT-CTTTGGTCA--TTCAGCGATGCTAACCAC--TTTT-CCATCAATAGGAAG
1599AT .         T--TTGTGG-CTTTGGTCA--TTCAGCGATGCTAACCAC--TTTT-CCATCAATAGGAAG
1941AT .         T--TTGTGG-CTTTGGTCA--TTCAGCGATGCTAACCAC--TTTT-CCATCAATAGGAAG
291NE .          T--CTGTGT-CTTTGGTCA--TTCAGCGATGCTAACCAC--TTTT-CCATCAATAGGAAG
2301SAR .        T--CTGTGT-CTTTGGTCA--TTCAGCGATGCTAACCAC--TTTT-CCATCAATAGGAAG
2313SAR .        T--CTGTGT-CTTTGGTCA--TTCAGCGATGCTAACCAC--TTTT-CCATCAATAGGAAG
271NE .          T--CTGTGT-CTTTGGTCA--TTCAGCGATGCTAACCAC--TTTT-CCATCAATAGGAAG
1935GER .        T--TTGTGG-CTTTGGTCA--TTCAGCGATGCTAACCAC--TTTT-CCATCAATAGGAAG
JBRSA122 .       T--CTGTGT-CTTTGGTCA--TTCAGCGATGCTAACCAC--TTTT-CCATCAATAGGAAG
276NE .          T--CTGTGT-CTTT-----------GCGATGCTAACCAC--TTTTTCCATCAATAGGAAG
272NE .          T--CTGTGT-CTTT-----------GCGATGCTAACCAC--TTTTTCCATCAATAGGAAG
2673ETHI .       T--CTGTGT-CTTTGGTCA--TTCAGCGATGCTAACCAC--TTTTTCCATCAATAGGAAG
1084AUSW .       T--CTGTGT-CTTTGGTCA--TTCAGCGATGCTAACCAC--TTTT-CCATCAATAGGAAG
JBPER62 .        T--CTGTGT-CT--CGTGACTTTCAGCGATGCTAACCAC--TTTT-CCATCAATAGGAAG
JBNZ72 .         T--CTGTGT-CTTTGATCA--TTCAACGATGCTAACCAC--TTTT-CCATCAATAGGAAG
1065MEX .        T--CTGTGT--TTTGGTCA--TTCAGCGATGCTAACCAC--TTTT-CCATCAATAGGAAG
646HAWAI .       XXXXXXXXXXXXXXXXXXXXXXXXXXXXXXXXXXXXXXXXXXXXXXXXXXXXXXXXXXXX
727INDIA .       XXXXXXXXXXXXXXXXXXXXXXXXXXXXXXXXXXXXXXXXXXXXXXXXXXXXXXXXXXXX
743MALE .        XXXXXXXXXXXXXXXXXXXXXXXXXXXXXXXXXXXXXXXXXXXXXXXXXXXXXXXXXXXX
1059MEX .        T-ACTGTGT-CTTTGGTCA--TTCAGCGATGCTAACCAC--TTTT-CCATCAATAGGAAG
1061RW .         T--CTGCGT-CTTTGGTCA--TTCAGCGACGCTAACCAC--TTTT-CCATCAATAGGAAG
1066MEX .        T--CTGTGT-CTTTGGTCA--TTCAGCGATGCTAACCAC--TTTT-CCATCAATAGGAAG
1095BALI .       T--CTGTGT-----CATCACTTTCAGCGATGCTAACCAC--TTTT-CCATCAATAGGAAG
1505MAUR .       T--CTGTGT-CT--CGTCACTTTCAGCAATGCTAACCAC--TTTT-CCATCAATAGGAAG
1818ETHI .       T--CTGCGT-CT--CATCACTTTCAGCGATGCTAACCAC--TTTT-CCATCAATAGGAAG
1934AT .         T--TTGTGG-CTTTGGTCA-TT-CAGCGATGCTAACCAC--TTTT-CCATCAATAGGAAG
245CR .          T--CTGTGT-----CATCACTTTCAGCGATGCTAACCAC--TTTT-CCATCAATAGGAAG
2710ETHI .       T--CTGTGT-----CATCACTTTCAGCGATGCTAACCAC--TTTT-CCATCAATAGGAAG
274NE .          T--CTGTGT-----CATCACTTTCAGCGATGCTAACCAC--TTTT-CCATCAATAGGAAG
3408PNG .        T--CTGTGT-CT--CGTCACTTTCAGCGATGCTAACCAC--TTTT-CCATCAATAGGAAG
3409PNG .        T--CTGTGT-CT--CGTCACTTTCAGCGATGCTAACCAC--TTTT-CCATCAATAGGAAG
410MALAY .       T--CTGCG-CCTT--ATCGCTTTCAGTGAAGCTAACCAC--TTTTT-CATCAATAGGAAG
836EGY .         T--CTGTGT-CT--CGTCACTTTCAGCAATGCTAACCAC--TTTT-CCATCAATAGGAAG
837EGY .         T--CTGTGT-CT--CGTCACTTTCAGCAATGCTAACCAC--TTTT-CCATCAATAGGAAG
JBRO111 .        T--CTGCGT-CT--CATCACTTTCAGCGATGCTAACCAC--TTTT-CCATCAATAGGAAG
brunneoviridis4  T--CCGTGT-CTTTGGTCA--TTCAGCGATGCTAACCAC--TTTTT-CATCAATAGGAAG
brunneoviridis1  T--CCGTGT-CTTTGGTCA--TTCAGCGATGCTAACCAC--TTTTT-CATCAATAGGAAG
pleurotumCPK211  T--CTGTGT-----CGCCA--TTCAGCGATGCTAACCAC--TTTT-CCATCAATAGGAAG
PLEUROTICOLA .   T--CTGTGT-CT--CGCCA--TTCAGCGATGCTAACCAC--TTTT-CCATCAATAGGAAG
epimycesCPK1981  T--CTGT---CT--CGTCA--TTCAGCGATGCTAACCAC--TTTTTCCATCAATAGGAAG
alniCPK3124 ...  T--CTGTCT-CT--CATCA--TGCAGCGATGCTGACCAC--TTTT-CCATCAATAGGAAG
CPK5TAWA .       T--CTGTCT-CT--CGTCA--TTCAGCGATGCTAACCGC--TTTT-CCATCAATAGGAAG
2974lobau .      T--CGGTGT-CT--CGTCACTTTCAGCGATGCTAACCAC--CTTT-CCATCAATAGGAAG
2975lobau .      T--CGGTGT-CT--CGTCACTTTCAGCGATGCTAACCAC--CTTT-CCATCAATAGGAAG
aggressivumCPK3  T--CTGTCT-CT--CGTCA--TCCAGTGATGCTAACCAC--TTTTTCCATCAATAGGAAG
atrogelatinosaC  C--CTGTGT-CT--CGTCAGTTTCAGCGATGCTAACCAC--TTTT-CCATCAATAGGAAG
```

```
atrogelatinosaC   C--CTGTGT-CT--CGTCAGTTTCAGCGATGCTAACCAC--TTTT-CCATCAATAGGAAG
cerinumCPK293 .   T--TTATGT-CT--ACTCACTTCCAGCGATGCTAACCAC--TTTT-CCATCAATAGGAAG
cerinumCPK854 .   T--TTGTGT-CT--TGTCACTTCCAGCGATGCTAATCAC--TTTT-CCATCAATAGGAAG
tomentosumCPK88   T--TTGTGT-CT--TGTCACTTTCAGCGATGCTAACCAC--TTTT-TCATCAATAGGAAG
stramineaGJS028   C--CTACGT-C----GTCACTTTCAGCGATGCTAACCAC--TTTT-CCATCAATAGGAAG
velutinumCPK312   T--CTGTAT-TC--CGTTACTTTCAACGATGCTAACCAC--TTT-CTCATCAATAGGAAG
catoptronGJS027   TCCATCCAT-GCCTTTTCATTTTCAACGATGCTAACCAC--TTTT-CCATCAATAGGAAG
                  #  ######      ########################        #############


                         310       320       330       340       350       360
                  =========+=========+=========+=========+=========+=========+
1093CAN .         CCGCCGAACTCGGCAAGGGTTCCTT-CATCGTTTCTGGTGGC-AATTCGGAC-GGTTT-G
1052SA .          CCGCCGAACTCGGCAAGGGTTCCTT-CATCGTTTCTGGTGGC-AATTCGGAC-GGTTT-G
1116CAN .         CCGCCGAACTCGGCAAGGGTTCCTT-CATCGTTTCTGGTGGC-AATTCGGAC-GGTTT-G
1070EIRE .        CCGCCGAACTCGGCAAGGGTTCCTT-CATCGTTTCTGGTGGC-A-TTCGGAC-GGTTT-G
1099CAN .         CCGCCGAACTCGGCAAGGGTTCCTT-CATCGTTTCTGGTGGC-AATTCGGAC-GGTTT-G
1104CAN .         CCGCCGAACTCGGCAAGGGTTCCTT-CATCGTTTCTGGTGGC-AATTCGGAC-GGTTT-G
1087CAN .         CCGCCGAACTCGGCAAGGGTTCCTT-CATCGTTTCTGGTGGC-AATTCGGAC-GGTTT-G
360UK .           CCGCCGAACTCGGTAAGGGTTCCTT-CATCGTTTCTGGTGGC-AATTCGGAC-GGTTT-G
2111HUN .         CCGCCGAACTCGGCAAGGGTTCCTT-CATCGTTTCTGGTGGC-AATTCGGAC-GGTTT-G
217RUS .          CCGCCGAACTCGGTAAGGGTTC-TTTCA-CGTTTCTGGTGGC-A-TTCGGAC-GGTTT-G
204UK .           CCGCCGAACTCGGTAAGGGTTC-TTTCATCGTTTCTGGTGGC-AATTCGGAC-GGTTT-G
206RUS .          CCGCCGAACTCGGTAAGGGTTC-TTTCATCGTTTCTGGTGGC-AATTCGGAC-GGTTT-G
261SIB .          CCGCCGAACTCGGTAAGGGTTC-TTTCATCGTTTCTGGTGGC-A-TTCGGAC-GGTTT-G
265SIB .          CCGCCGAACTCGGCAAGGGTTCCTT-CATCGTTTCTGGTGGC-A-TTCGGAC-GGTTT-G
2784THAI .        CCGCCGAACTCGGTAAGGGTTCCTT-CATCGTTTCTGGTGGC-A-TTCGGAC-GGTTT-G
838EGY .          CCGCCGAACTCGGTAAGGGTTCCTT-CATCGTTTCTGGTAGC-A-TTCGGAC-GATTT-G
1102CAN .         CCGCCGAACTCGGCAAGGGTTCCTT-CATCGTTTCTGGTGGC-AATTCGGAC-GGTTT-G
588LAOS .         CCGCCGAACTCGGCAAGGGTTCCTT-CATCGTTTCTTGTGGC-A-TTCGGAC-GGTTT-G
1596AT .          CCGCCGAACTCGGCAAGGGTTXXXXXXXXXXXXXXXXGTGGC-A-TTCGGAC-GGTTT-G
1720CAM .         CCGCCGAACTCGGCAAGGGTTXXXXXXXXXXXXXXXXXXXXX-XXXXXXXXXXXXXXXXX
1722CAM .         CCGCCGAACTCGGCAAGGGTTCCTT-CAXXXXXXXXXXXXXXX-XXXXXXXXXXXXXXXXX
1724CAM .         CCGCCGAACTCGGCAAGGGTTCCTT-CAXXXXXXXXXXXXXXX-XXXXXXXXXXXXXXXXX
334USA .          CCGCCGAACTCGGCAAGGGTTCCTT-CAXXXXXXXXXXGTGGC-A-TTCGGAC-GGTTT-G
335USA .          CCGCCGAACTCGGCAAGGGTTCCTT-CAXXXXXXXXXXGTGGC-A-TTCGGAC-GGTTT-G
1081WIS .         CCGCCGAACTCGGCAAGGGTTCCTT-CATCGTTTCTGGTGGC-AATTCGGAC-GCTTT-G
1069WIS .         CCGCCGAACTCGGCAAGGGTTCCTT-CATCGTTTCTGGTGGC-AATTCGGAC-GGTTT-G
1107CAN .         CCGCCGAACTCGGCAAGGGTTCCTT-CATCGTTTCTGGTGGC-AATTCGGAC-GGTTT-G
1108PEN .         CCGCCGAACTCGGCAAGGGTTCCTT-CATCGTTTCTGGTGGC-AATTCGGAC-GGTTT-G
1110CAN .         CCGCCGAACTCGGCAAGGGTTCCTT-CATCGTTTCTGGTGGC-AATTCGGAC-GGTTT-G
JBSER241 .        CCGCCGAACTCGGCAAGGGTTCCTT-CATCGTTTCTAGTGGC-AATTCGGAC-GGTTT-G
246PHI .          XXXXXXXXXXXXXXXXXXXXXXXXXXXXTCGTTTCTGGTGGC-AATTCGGAC-GCTTTAG
51SA .            CCGCCGAACTCGGTAAGGGTTCCTT-CATCGTTTCTAGTGGC-A-TTCGGAC-GCTTT-G
2618ETHI .        CCGCCGAACTCGGCAAGGGTTCCTT-CATCGTTTCTGGTGGC-A-TTCGGAC-GCTTT-G
1068WIS .         CCGCCGAACTCGGCAAGGGTTCCTT-CATCGTTTCTGGTGGC-AATTCGGAC-GGTTT-G
238CR .           CCGCCGAACTCGGTAAGGGTTCCTT-CAXXXXXXXXTGGTGGC-AATTCGGAC-GCTTT-G
NR5555 .          CCGCCGAACTCGGTAAGGGTTCCTT-CATCGTTTCTGGTGGC-A-TTCGGAC-GCTTT-G
NR6839 .          CCGCCGAACTCGGTAAGGGTTC-TTTCATCGTTTCTGGTGGC-A-TTCGGAC-GCTTT-G
2624ETHI .        CCGCCGAACTCGGCAAGGGTTCCTT-CATCGTTTCTGGTGGC-A-TTCGGAC-GGTTT-G
845EGY .          CCGCCGAACTCGGTAAGGGTTCCTT-CAXXXXXXXXXXXXXXX-XXXXXXXXXXXXXXT-G
JBPER12 .         CCGCCGAACTCGGCAAGGGTTCCTT-CATCGTTTCTGGTAGC-AATTCGGAC-GATTT-G
1717VIET .        CCGCCGAACTCGGCAAGGGTTCCTT-CAXXXXXXXXXXXXXXX-XXXXXXXXXXXXXXXXX
709BRAZ .         CCGCCAAACTCGGTAAGGGTTCCTTTCATCGTTTCTGGTAGC-A-TTCGGACAGATTTAG
878IRAN .         XXXXXXXXXXXXXXXXXXXXXXXXXXXXTCGTTTCTGGTGGC-A-TTCGGAC-GGTTT-G
239CR .           XXXXXXXXXXXXXXXXXXXXXXXXXXXXTCGTTTCTGGTAGC-A-TTCGGAC-GATTT-G
202COM .          CCGCCGAACTCGGTAAGGGTTCCTTTCATCGTTTCTAGTGGC-AATTCGGAC-GGTTT-G
1064MEX .         CCGCCGAACTCGGCAAGGGTTCCTT-CATCGTTTCTGGTAGC-AATTCGGAC-GATTT-G
596BRAZ .         CCGCCGAACTCGGTAAGGGTTC-TTTCATCGTTTCTGGTGGC-A-TTCGGAC-GGTTT-G
333USA .          XXXXXXXXXXXXXXXXXXXXXXXXXXXXXXXXXXXXXGTAGC-A-TTCGGAC-GATTT-G
2610ETHI .        CCGCCGAACTCGGCAAGGGTTCCTT-CATCGTTTCTGGTGGC-A-TTCGGAC-GGTTT-G
1044RW .          CCGCCGAACTCGGCAAGGGTTCCTT-CATCGTTTCTGGTGGC-AATTCGGAC-GGTTT-G
JBNZ111 .         CCGCCGAACTCGGCAAGGGTTCCTT-CATCGTTTCTTGTGGC-AATTTGGAC-GGTTT-G
JBNZ12 .          CCGCCGAACTCGGCAAGGGTTCCTT-CATCGTTTCTAGTGGC-AATTCGGAC-GGTTT-G
JBNZ24 .          CCGCCGAACTCGGCAAGGGTTCCTT-CATCGTTTCTAGTGGC-AATTCGGAC-GGTTT-G
693CHINA .        CCGCCGAACTCGGCAAGGGTTCCTT-CATCGTTTCTGGTGGC-A-TTCGGAC-GGTTT-G
1058RW .          CCGCCGAACTCGGCAAGGGTTCCTT-CATCGTTTCTGGTGGC-A-TTCGGAC-GGTTT-G
590AUS .          CCGCCGAACTCGGTAAGGGTTCCTT-CATCGTTTCTGGTGGC-A-TTCGGAC-GGTTT-G
53SA .            CCGCCGAACTCGGCAAGGGTTCCTT-CAXXXXXXXXTGGTGGC-A-TTCGGAC-GGTTT-G
1075WIS .         CCGCCGAACTCGGCAAGGGTTCCTT-CAXXXXXTTCTGGTGGC-AATTCGGAC-GGTTT-G
939GER .          CCGCCGAACTCGGCAAGGGTTCCTT-CAXXXXXXXXX-GTGGC-A-TTCGGAC-GGTTT-G
GA3804 .          CCGCCGAACTCGGCAAGGGTTCCTT-CATCGTTTCTGGTGGC-AATTCGGAC-GGTTT-G
1599AT .          CCGCCGAACTCGGCAAGGGTTCCTT-CAXXXXXXXXXXGTGGC-A-TTCGGAC-GGTTT-G
1941AT .          CCGCCGAACTCGGCAAGGGTTCCTT-CATCGTTTCTAGTGGC-A-TTCGGAC-GGTTT-G
291NE .           CCGCCGAACTCGGTAAGGGTTCCTTTCATCGTTTCTGGTGGC-A-TTCGGAC-GGTTT-G
2301SAR .         CCGCCGAACTCGGCAAGGGTTCCTT-CATCGTTTCTAGTGGC-A-TTCGGAC-GGTTT-G
2313SAR .         CCGCCGAACTCGGCAAGGGTTCCTT-CATCGTTTCTAGTGGC-A-TTCGGAC-GGTTT-G
271NE .           CCGCCGAACTCGGTAAGGGTTCCTTTCATCGTTTCTGGTGGC-AATTCGGAC-GGTTT-G
1935GER .         CCGCCGAACTCGGCAAGGGTTCCTT-CAXXXXXXXXTAGTGGC-A-TTCGGAC-GGTTT-G
JBRSA122 .        CCGCCGAACTCGGCAAGGGTTCCTT-CATCGTTTCTAGTGGC-AATTCGGAC-GGTTT-G
276NE .           CCGCCGAACTCGGTAAGGGTTC-TTTCATCGTTTCTGGTGGC-AATTCGGAC-GGTTT-G
272NE .           CCGCCGAACTCGGTAAGGGTTCCTTTCATCGTTTCTGGTGGC-AATTCGGAC-GGTTT-G
2673ETHI .        CCGCCGAACTCGGCAAGGGTTCCTT-CATCGTTTCTGGTGGC-A-TTCGGAC-GGTTT-G
1084AUSW .        CCGCCGAACTCGGCAAGGGTTCCTT-CATCGTTTCTGGTGGC-AATTCGGAC-GGTTT-G
JBPER62 .         CCGCCGAACTCGGCAAGGGTTCCTT-CATCGTTTCTGGTGGC-AATTCGGAC-GGTTT-G
JBNZ72 .          CCGCCGAACTCGGCAAGGGTTCCTT-CATCGTTTCTAGTGGC-AATTCGGAC-GGTTT-G
1065MEX .         CCGCCGAACTCGGCAAGGGTTCCTT-CATCGTTTCTGGTGGC-AATTCGGAC-GGTTT-G
646HAWAI .        XXXXXXXXXXXXXXXXXXXXXXXXXXXXTCGTTTCTGGTGGC-A-TTCGGAC-GGTTT-G
727INDIA .        XXXXXXXXXXXXXXXXXXXXXXXXXXXXTCGTTTCTGGTGGC-A-TTCGGAC-GGTTT-G
743MALE .         XXXXXXXXXXXXXXXXXXXXXXXXXXXXTCGTTTCTGGTGGC-A-TTCGGAC-GGTTT-G
1059MEX .         CCGCCGAACTCGGCAAGGGTTCCTT-CATCGTTTCTGGTGGC-AATTCGGAC-GGTTT-G
1061RW .          CCGCCGAACTCGGCAAGGGTTCCTT-CATCGTTTCTGGTGGC-A-TTCGGAC-GCTTT-G
1066MEX .         CCGCCGAACTCGGCAAGGGTTCCTT-CATCGTTTCTTGTGGC-AATTCGGAC-GGTTT-G
1095BALI .        CCGCCGAACTCGGCAAGGGTTCCTT-CATCGTTTCTTGTGGC-AATTCGGAC-GGTTT-G
1505MAUR .        CCGCCGAACTCGGCAAGGGTTCCTT-CATCGTTTCTGGTGGC-A-TTCGGAC-GGTTT-G
1818ETHI .        CCGCCGAACTCGGCAAGGGTTCCTT-CATCGTTTCTGGTGGC-A-TTCGGAC-GGTTT-G
1934AT .          CCGCCGAACTCGGCAAGGGTTCCTT-CATCGTTTCTGGTGGC-A-TTCGGAC-GGTTT-G
245CR .           CCGCCGAACTCGGTAAGGGTTCCTTTCATCGTTTCTTGTGGC-A-TTCGGAC-GGTTT-G
2710ETHI .        CCGCCGAACTCGGCAAGGGTTCCTT-CAXXXXXXXXX-GTGGC-A-TTCGGAC-GGTTT-G
274NE .           CCGCCGAACTCGGTAAGGGTTC-TTTCATCGTTTCTTGTGGC-A-TTCGGAC-GGTTT-G
3408PNG .         CCGCCGAACTCGGCAAGGGTTCCTT-CAXXXXXXTCTGGTGGC-A-TTCGGAC-GGTTT-G
3409PNG .         CCGCCGAACTCGGCAAGGGTTCCTT-CAXXXXXXTCTGGTGGC-A-TTCGGAC-GGTTT-G
410MALAY .        CCGCCGAACTCGGCAAGGGTTCCTT-CATCGTTTCTTGTGGC-A-TTCGGGC-TGTTT-G
836EGY .          CCGCCGAACTCGGTAAGGGTTCCTTTCATCGTTTCTGGTGGC-A-TTCGGAC-GGTTT-G
837EGY .          CCGCCGAACTCGGTAAGGGTTCCTT-CATCGTTTCTGGTGGC-A-TTCGGAC-GGTTT-G
JBRO111 .         CCGCCGAACTCGGCAAGGGTTCCTT-CATCGTTTCTGGTGGC-AATTCGGAC-GGTTT-G
brunneoviridis4   CCGCTGAACTCGGCAAGGGTTCCTT-CATCGTTTCTTGTGGC-A-TTCCGAC-GGTTT-G
brunneoviridis1   CCGCTGAACTCGGCAAGGGTTCCTT-CATCGTTTCTTGTGGC-A-TTCCGAC-GGTTT-G
pleurotumCPK211   CCGCCGAACTCGGCAAGGGTTCCTTGCCXCGTTTCTCGTGGC-A-TTCGGAC-GGTTT-G
PLEUROTICOLA .    CCGCCGAACTCGGCAAGGGTTCCTTCXXXXXXXXXXXXX-GC-A-TTCGGAC-GGTTT-G
epimycesCPK1981   CCGCCGAACTCGGCAAGGGTTCCTTGCTTCGTTTCTTGTGGC-A-TTCGGCC-GGTTT-G
alniCPK3124 ...   CCGCCGAACTCGGCAAGGGTTCCTTGCTXXXXXXXXXXXXGC-A-TTCGGAC-GGTTT-G
CPK5TAWA .        CCGCCGAACTCXXXXXXXXXXXXXXXXXXXXXXXXXXXXXXX-XXXXXXXXXXXXXXXXX
2974lobau .       CCGCCGAACTCGGCAAGGGTTCCTT-CATCGTTTCTGGAGGCGA-TTCGGAC-GGTTT-G
2975lobau .       CCGCCGAACTCGGCAAGGGTTCCTT-CATCGTTTCTGGAGGCGA-TTCGGAC-GGTTT-G
aggressivumCPK3   CCGCCGAACTCGGTAAGGGTTCCTTGCTXCGTTTCTTGTGGC-A-TTCGGAC-GGTTT-G
atrogelatinosaC   CCGCCGAACTCGGTAAGGGTTCCTTGCCTCGTTTCTCGTGGC-A-TTCGGAC-GGTTT-G
```

```
atrogelatinosaC  CCGCCGAACTCGGCAAGGGTTCCTTGCCTCGTTTCTTGTGGC-A-TTCGGAC-GGTTT-G
cerinumCPK293 .  CCGCCGAACTCGGTAAGGGTTCCTTGCTTCG-TTCTTGTGGC-A-TTCGGAC-GGTTT-G
cerinumCPK854 .  CCGCCGAACTCGGTAAGGGTTCCTTGCTTCG-TTCTTGTGGC-A-TTCGGAC-GGTTT-G
tomentosumCPK88  CCGCCGAACTCGGTAAGGGTTCTTTGCTTTG-TTCTTGTGGC-A-TTCGGAC-GGTTT-G
stramineaGJS028  CCGCCGAACTCGGCAAGGGTTCCTTGCCTCGTTTCTAGTGGC-A-TTCGGAC-GATTT-G
velutinumCPK312  CCGCTGAACTCGGCAAGGGTTCCTTGCCTCGTTTCTAGTGGC-A-TTCGGGC-GATTT-G
catoptronGJS027  CCGCCGAACTTGGCAAGGGTTCCTTACCTCGTTTCTCGTGGC-A-TTCAGAC-TGTTT-G
                 ######################### ################   ####### #####


                        370       380       390       400       410       420
                 =========+=========+=========+=========+=========+=========+
1093CAN .        AGT-GC-TGACCCAGCT-GCTACAGGACAAGGACGGTGATGGTACGTAGCA-GCGAGTGA
1052SA .         AGT-GC-TGACCCAGCT-GCTACAGGACAAGGACGGTGATGGTACGTAGCA-GCGAGTGA
1116CAN .        AGT-GC-TGACCCAGCT-GCTACAGGACAAGGACGGTGATGGTACGTAGCA-GCGAGTGA
1070EIRE .       AGT-GC-TGACCCAGCT-GCTACAGGACAAGGACGGTGATGGTACGTAGCA-GCGAGTGA
1099CAN .        AGT-GC-TGACCCAGCT-GCTACAGGACAAGGACGGTGATGGTACGTAGCA-GCGAGTGA
1104CAN .        AGT-GC-TGACCCAGCT-GCTACAGGACAAGGACGGTGATGGTACGTAGCA-GCGAGTGA
1087CAN .        AGT-GC-TGACCCAGCT-GCTACAGGACAAGGACGGTGATGGTACGTAGCA-GCGAGTGA
360UK .          AGT-GC-TGACCCAGCT-GCTACAGGACAAGGACGGTGATGGTACGTAGCA-GCGAGTGA
2111HUN .        AGT-GC-TGACCCAGCT-GCTACAGGACAAGGACGGTGATGGTACGTAGCA-GCGAGTGA
217RUS .         AGT-GC-TGACCCAGCT-GCTACAGGACAAGGACGGTGATGGTACGTAGCA-GCGAGTGA
204UK .          AGT-GC-TGACCCAGCT-GCTACAGGACAAGGACGGTGATGGTACGTAGCA-GCGAGTGA
206RUS .         AGT-GC-TGACCCAGCT-GCTACAGGACAAGGACGGTGATGGTACGTAGCA-GCGAGTGA
261SIB .         AGT-GC-TGACCCAGCT-GCTACAGGACAAGGACGGTGATGGTACGTAGCA-GCGAGTGA
265SIB .         AGT-GC-TGACCCAGCT-GCTACAGGACAAGGACGGTGATGGTACGTAGCA-GCGAGTGA
2784THAI .       AGT-GC-TAACCCAGCTTGCTACAGGACAAGGACGGTGGTGGTACGTAGCA-GCGAGTGA
838EGY .         AGT-GC-TAACCCAGCT-GCTACAGGACAAGGACGGTGATGGTACGTAGCA-GCGAGTGA
1102CAN .        AGT-GC-TAACCCAGCTTGCTACAGGACAAGGACGGTGATGGTACGTAGCA-ACGAGTGA
588LAOS .        AGT-GC-TAACCCAGCTTGCTACAGGACAAGGACGGTGATGGTACGTAGCA-GCGAGTGA
1596AT .         AGT-GC-TAACCCAGCTTGCTACAGGACAAGGACGGTGATGGTACGTAGCA-GCGAGTGA
1720CAM .        XXXXXXXXXXXXXXXXXXXXXXXXXXXXXXXXA-GG-GAGGGTACGTAGCA-GCGAGTGA
1722CAM .        XXXXXXXXXXXXXXXXXXXXXXXXXXXXXXXXXXGGTGATGGTACGTAGCA-GCGAGTGA
1724CAM .        XXXXXXXXXXXXXXXXXXXXXXXXXXXXXXXXXCGGTGAGGGTACGTAGCA-GCGAGTGA
334USA .         AGT-GC-TAACCCAGCTTGCTACAGGACAAGGACGGTGATGGTACGTAGCA-ACGAGTGA
335USA .         AGT-GC-TAACCCAGCTTGCTACAGGACAAGGACGGTGATGGTACGTAGCA-ACGAGTGA
1081WIS .        AGT-GC-TAACCCAGCTTGCTACAGGACAAGGACGGTGATGGTACGTAGCA-GCGAGTGA
1069WIS .        AGT-GC-TAACCCAGCTTGCTACAGGACAAGGACGGTGATGGTACGTAGCA-ACGAGTGA
1107CAN .        AGT-GC-TAACCCAGCTTGCTACAGGACAAGGACGGTGATGGTACGTAGCA-ACGAGTGA
1108PEN .        AGT-GC-TAACCCAGCTTGCTACAGGACAAGGACGGTGATGGTACGTAGCA-GCGAGTGA
1110CAN .        AGT-GC-TGACCCAGCT-GCTACAGGACAAGGACGGTGATGGTACGTAGCA-GCGAGTGA
JBSER241 .       AGT-GC-TAACCCAGCT-GCTACAGGACAAGGACGGTGATGGTACGTAGCA-GCGAGTGA
246PHI .         AGT-GC-TAACCCAGCT-GTTACAGGACAAGGACGGTGATGGTACGTAGCA-GCGAGTGA
51SA .           AGT-GC-TAACCCAGCT-GTTACAGGACAAGGACGGTGATGGTACGTAGCA-GCGAGTGA
2618ETHI .       AGT-GC-TAACCCAGCT-GTTACAGGACAAGGACGGTGATGGTACGTAGCA-GCGAGTGA
1068WIS .        AGT-GC-TAACCCAGCTTGCTACAGGACAAGGACGGTGATGGTACGTAGCA-ACGAGTGA
238CR .          AGT-GCGTAACCCAGCT-GTTACAGGACAAGGACGGTGATGGTACGTAGCA-GCGAGTGA
NR5555 .         AGT-GC-TAACCCAGCT-GTTACAGGACAAGGACGGTGATGGTACGTAGCA-GCGAGTGA
NR6839 .         AGT-GC-TAACCCAGCT-GCTACAGGACAAGGACGGTGATGGTACGTAGCA-GCGAGTGA
2624ETHI .       AGT-GC-TAACCCAGCT-GCTACAGGACAAGGACGGTGATGGTACGTAGCA-GCGAGTGA
845EGY .         AGTAGCGTAACCCAGCT-GCTACAGGACAAGGACGGTGATGGTACGTAGCA-GCGAGTGA
JBPER12 .        AGT-GC-TAACCCAGCT-GCTACAGGACAAGGACGGTGATGGTACGTAGCA-GCGAGTGA
1717VIET .       XXXXXXXXXXXXXXXXXXXXXXXXXXXXXXXXXXXXXXXXXXXXXXXXXXXXXXXXXXXX
709BRAZ .        AGT-GC-TAACCCAGCT-GCTACAGGACAAGGACGGTGATGGTACGTAGCA-GCGAGTGA
878IRAN .        AGT-GC-TAACCCAGCTTGCTACAGGACAAGGACGGTGATGGTACGTAGCA-GCGAGTGA
239CR .          AGT-GC-TAACCCAGCT-GCTACAGGACAAGGACGGTGATGGTACGTAGCA-GCGAGTGA
202COM .         AGT-GC-TAACCCTGCT-GCTACAGGACAAGGACGGTGATGGTACGTAGCA-GCGAGTGA
1064MEX .        AGT-GC-TAACCCAGCT-GCTACAGGACAAGGACGGTGATGGTACGTAGCA-GCGAGTGA
596BRAZ .        AGT-GC-TAACCCAGCT-GCTACAGGACAAGGACGGTGATGGTACGTAGCA-GCGAGTGA
333USA .         AGT-GC-TAACCCAGCT-GCTACAGGACAAGGACGGTGATGGTACGTAGCA-GCGAGTGA
2610ETHI .       AGT-GC-TAACCCAGCT-GCTACAGGACAAGGACGGTGATGGTACGTAGCA-GCGAGTGA
1044RW .         AGT-GC-TAACCCAGCT-GCTACAGGACAAGGACGGTGATGGTACGTAGCA-GCGAGTGA
JBNZ111 .        AGT-GC-TAACTTAGCTTGCTACAGGACAAGGACGGTGATGGTACGTAGCA-GCGAGTGA
JBNZ12 .         AGT-GC-TAACCCAGCT-GCTACAGGACAAGGACGGTGATGGTACGTAGCA-GCGAGTGA
JBNZ24 .         AGT-GC-TAACCCAGCT-GCTACAGGACAAGGACGGTGATGGTACGTAGCA-GCGAGTGA
693CHINA .       AGT-GC-TAACCCAGCT-GCTACAGGACAAGGACGGTGATGGTACGTAGCA-GCGAGTGA
1058RW .         AGT-GC-TAACCCAGCT-GCTACAGGACAAGGACGGTGATGGTACGTAGCA-GCGAGTGA
590AUS .         AGT-GC-TAACCCAGCTTGCTACAGGACAAGGACGGTGATGGTACGTAGCA-GCGAGTGA
53SA .           AGT-GC-TAACCCAGCT-GCTACAGGACAAGGACGGTGATGGTACGTAGCA-GCGAGTGA
1075WIS .        AGT-GC-TAACCCAGCT-GCTACAGGACAAGGACGGTGATGGTACGTAGCA-GCGAGTGA
939GER .         AGT-GC-TAACCCAGCT-GCTACAGGACAAGGACGGTGATGGTACGTAGCA-GCGAGTGA
GA3804 .         AGT-GC-TAACCCAGCT-GCTACAGGACAAGGACGGTGATGGTACGTAGCA-GCGAGTGA
1599AT .         AGT-GC-TAACCCAGCT-GCTACAGGACAAGGACGGTGATGGTACGTAGCA-GCGAGTGA
1941AT .         AGT-GC-TAACCCAGCT-GCTACAGGACAAGGACGGTGATGGTACGTAGCA-GCGAGTGA
291NE .          AGTA-C-TAACCCAGCT-GCTACAGGACAAGGACGGTGATGGTACGTAGCA-GCGAGTGA
2301SAR .        AGT-GC-TAACCCAGCT-GCTACAGGACAAGGACGGTGATGGTACGTAGCA-GCGAGTGA
2313SAR .        AGT-GC-TAACCCAGCT-GCTACAGGACAAGGACGGTGATGGTACGTAGCA-GCGAGTGA
271NE .          AGT-GC-TAACCCAGCT-GCTACAGGACAAGGACGGTGATGGTACGTAGCA-GCGAGTGA
1935GER .        AGT-GC-TAACCCAGCT-GCTACAGGACAAGGACGGTGATGGTACGTAGCA-GCGAGTGA
JBRSA122 .       AGT-GC-TAACCCAGCT-GCTACAGGACAAGGACGGTGATGGTACGTAGCA-GCGAGTGA
276NE .          AGT-GC-TAACCCAGCT-GCTACAGGACAAGGACGGTGATGGTACGTAGCA-GCGAGTGA
272NE .          AGT-GC-TAACCCAGCT-GCTACAGGACAAGGACGGTGATGGTACGTAGCA-GCGAGTGA
2673ETHI .       AGT-GC-TAACCCAGCT-GCTACAGGACAAGGACGGTGATGGTACGTAGCA-GCGAGTGA
1084AUSW .       AGT-GC-TAACCCAGCT-GCTACAGGACAAGGACGGTGATGGTACGTAGCA-GCGAGTGA
JBPER62 .        AGT-GC-TAACCCAGCT-GCTACAGGACAAGGACGGTGATGGTACGTAGCA-GCGAGTGA
JBNZ72 .         AGT-GC-TAACCCAGCT-GCTACAGGACAAGGACGGTGATGGTACGTAGCA-GCGAGTGA
1065MEX .        AGT-GC-TAACCCAGCT-GCTACAGGACAAGGACGGTGATGGTACGTAGCA-GCGAGTGA
646HAWAI .       AGT-GC-TAACCCAGCT-GCTACAGGACAAGGACGGTGATGGTACGTAGCA-GCGAGTGA
727INDIA .       AGT-GC-TAACCCAGCTTGCTACAGGACAAGGACGGTGATGGTACGTAGCA-GCGAGTGA
743MALE .        AGT-GC-TAACCCAGCTTGCTACAGGACAAGGACGGTGATGGTACGTAGCA-GCGAGTGA
1059MEX .        AGT-GC-TAACCCAGCTTGCTACAGGACAAGGACGGTGATGGTACGTAGCA-GCGAGTGA
1061RW .         AGT-GC-TAACCCAGCT-GTTACAGGACAAGGACGGTGATGGTACGTAGCA-GCGAGTGA
1066MEX .        AGT-GC-TAACCCAGCTTGCTACAGGACAAGGACGGTGATGGTACGTAGCA-GCGAGTGA
1095BALI .       AGT-GC-TAACCCAGCTTGCTACAGGACAAGGACGGTGATGGTACGTAGCA-GCGAGTGA
1505MAUR .       AGT-GC-TAACCCAGCT-GCTACAGGACAAGGACGGTGATGGTACGTAGCA-GCGAGTGA
1818ETHI .       AGT-GC-TGACCCAGCT-GCTACAGGACAAGGACGGTGATGGTACGTAGCA-GCGAGTGA
1934AT .         AGT-GC-TAACCCAGCT-GCTACAGGACAAGGACGGTGATGGTACGTAGCA-GCGAGTGA
245CR .          AGT-GC-TAACCCAGCTTGCTACAGGACAAGGACGGTGATGGTACGTAGCA-GCGAGTGA
2710ETHI .       AGT-GC-TAACCCAGCTTGCTACAGGACAAGGACGGTGATGGTACGTAGCA-GCGAGTGA
274NE .          AGT-GC-TAACCCAGCTTGCTACAGGACAAGGACGGTGATGGTACGTAGCA-GCGAGTGA
3408PNG .        AGT-GC-TAACACAGCT-GCTACAGGACAAGGACGGTGATGGTACGTAGCA-GCGAGTGA
3409PNG .        AGT-GC-TAACACAGCT-GCTACAGGACAAGGACGGTGATGGTACGTAGCA-GCGAGTGA
410MALAY .       AGT-GC-TAACCCAGCT-GCTACAGGACAAGGACGGTGATGGTACGTAGCA-GCGAGTGA
836EGY .         AGT-GC-TAACCCAGCT-GCTACAGGACAAGGACGGTGATGGTACGTAGCA-GCGAGTGA
837EGY .         AGT-GC-TAACCCAGCT-GCTACAGGACAAGGACGGTGATGGTACGTAGCA-GCGAGTGA
JBRO111 .        AGT-GC-TGACCCAGCT-GCTACAGGACAAGGACGGTGATGGTACGTAGCA-GCGAGTGA
brunneoviridis4  AGT-GC-TAACCCAGCT-GCTACAGGACAAGGACGGTGATGGTACGTAGCA-GCGAGTGA
brunneoviridis1  AGT-GC-TAACCCAGCT-GCTACAGGACAAGGACGGTGATGGTACGTAGCA-GCGAGTGA
pleurotumCPK211  AGT-GC-TAACCCAGCT-GCTACAGGACAAGGACGGTGATGGTACGTAGCAAGCGAGTGA
PLEUROTICOLA .   AGT-GC-TAACCCAGCT-GCTACAGGACAAGGACGGTGATGGTACGTAGCAAGCGAGTGA
epimycesCPK1981  TGT-GC-TAACCCAGCTTGCTACAGGACAAGGACGGTGATGGTACGTAGCA-GCGAGTGA
alniCPK3124 ...  AGT-GC-TAACCCAGCT-GCTACAGGACAAGGACGGTGATGGTACGTAGCA-GCGAGTGA
CPK5TAWA .       XXXXXXXXXXXXXXXXXXXXXXXXXXXXXXXXXXXXXXXXXXXXXXXXXXXXXXXXXXXX
2974lobau .      AGT-GC-TAACCCAGCT-GCTACAGGACAAGGACGGTGATGGTACGTAGCA-GCGAGTGA
2975lobau .      AGT-GC-TAACCCAGCT-GCTACAGGACAAGGACGGTGATGGTACGTAGCA-GCGAGTGA
aggressivumCPK3  AGT-GC-TAACCCAGCTTGCTACAGGACAAGGACGGTGATGGTACGTAGCA-GCGAGTGA
atrogelatinosaC  AGT-GC-TAACCCAGCT-GCTACAGGACAAGGACGGTGATGGTACGTAGCA-GCGAGTGA
```

```
atrogelatinosaC  AGT-GC-TAACCCAGCT-GCTACAGGACAAGGACGGTGATGGTACGTAGCA-GCGAGTGA
cerinumCPK293 .  AGT-GC-TAACCCAGCT-GCTACAGGACAAGGACGGTGATGGTACGTAGCA-GCGAGTGA
cerinumCPK854 .  AGT-GC-TAACCCAGCT-GCTACAGGACAAGGACGGTGATGGTACGTAGCA-GCGAGTGA
tomentosumCPK88  AGT-GC-TAACCCAGCT-GCTACAGGACAAGGACGGTGATGGTACGTAGCA-GCGAGTGA
stramineaGJS028  AGT-GC-TAACCCAGCT-GCTACAGGACAAGGACGGTGATGGTACGTAGCAA-CGAGTGA
velutinumCPK312  AGT-GC-TAACCCAGCT-GCTACAGGACAAGGACGGTGATGGTACGTAGCA-GCGAGTGA
catoptronGJS027  AGT-GC-TGACCCAGCT-GCTCCAGGACAAGGACGGTGATGGTACGTAGCA-GCGAGTGA
                        ########## ################################# ########


                        430       440       450       460       470       480
                 =========+=========+=========+=========+=========+=========+
1093CAN .        CGCGACACCACCCCTCTCCCCC----TTTTGCGAACAGGCACTGGAGCACCGAATGACTT
1052SA .         CGCGACACCACCCCTCTCCCCC----TTTTGCGAACAGGCACTGGAGCACCGAATGACTT
1116CAN .        CGCGACACCACCCCTCTCCCCC----TTTTGCGAACAGGCACTGGAGCACCGAATGACTT
1070EIRE .       CGCGACACCACCCCTCTCCCCC----TTTTGCGAACAGGCACTGGAGCACCGAATGACTT
1099CAN .        CGCGACACCACCCCTCTCCCCC----TTTTGCGAACAGGCACTGGAGCACCGAATGACTT
1104CAN .        CGCGACACCACCCCTCTCCCCC----TTTTGCGAACAGGCACTGGAGCACCGAATGACTT
1087CAN .        CGCGACACCACCCCTCTCCCCC----TTTTGCGAACAGGCACTGGAGCACCGAATGACTT
360UK .          CGCGACACCACCCCTCTCCCCC----TTTTGCGAACAGGCACTGGAGCACCGAATGACTT
2111HUN .        CGCGACACCACCCCTCTCCCCC----TTTTGCGAACAGGCACTGGAGCACCGAATGACTT
217RUS .         CGCGACACCACCCCTCTCCCCC----TTTTGCGAACAGGCACTGGAGCACCGAATGACTT
204UK .          CGCGACACCACCCCTCTCCCCC----TTTTGCGAACAGGCACTGGAGCACCGAATGACTT
206RUS .         CGCGACACCACCCCTCTCCCCC----TTTTGCGAACAGGCACTGGAGCACCGAATGACTT
261SIB .         CGCGACACCACCCCTCTCCCCC----TTTTGCGAACAGGCACTGGAGCACCGAATGACTT
265SIB .         CGCGACACCACCCCTCTCCCCC----TTTTGCGAACAGGCACTGGAGCACCGAATGACTT
2784THAI .       CGCGACACCACCCCTCTCCCCC----TTTTGCGAACAGGCACCGGAGCACCGAATGACTT
838EGY .         CGCGACACCACCCCTCTCCCCC----TTTTGCGAACAGGCACCGGAGCACCGAATGACTT
1102CAN .        CGCGACACCACCCCTCCCCCCC----TTTTGCGAACAGGCACCGGAGCATCGAATGACTT
588LAOS .        CGCGACACCACCCCTC-CCCCC----TTTTGCGAATAGCTATCGGAGCACCGAGTGACTT
1596AT .         CGCGACACCACCCCTCTCCCCC----TTTTGCGAACAGGCACCGGAGCACCGAATGACTT
1720CAM .        CGCGACACCACCCCTCTCCCCC----TTTTGCGAACAGGCACCGGAGCACCGAATGACTT
1722CAM .        CGCGACACCACCCCTCTCCCCC----TTTTGCGAACAGGCACCGGAGCACCGAATGACTT
1724CAM .        CGCGACACCACCCCTCTCCCCC----TTTTGCGAACAGGCACCGGAGCACCGAATGACTT
334USA .         CGCGACACCACCCCTCCCCCCC----TTTTGCGAACAGGCACCGGAGCATCGAATGACTT
335USA .         CGCGACACCACCCCTCCCCCCC----TTTTGCGAACAGGCACCGGAGCATCGAATGACTT
1081WIS .        CGCGACACCACCCCTC-CCCCC----TTTTGCGAACAGGCACTGGAGCACCGAATGACTT
1069WIS .        CGCGACACCACCCCTCCCCCCC----TTTTGCGAACAGGCACCGGAGCATCGAATGACTT
1107CAN .        CGCGACACCACCCCTCCCCCCC----TTTTGCGAACAGGCACCGGAGCATCGAATGACTT
1108PEN .        CGCGACACCACCCCTCTCCCCC----TTTTGCGAACAGGCACCGGAGCACCGAATGACTT
1110CAN .        CGCGACACCACCCCTCTCCCCC----TTTTGCGAACAGGCACTGGAGCACCGAATGACTT
JBSER241 .       CGCGACACCACCCCTCTCCCCC----TTTTGCGAACAGGCACCGGAGCACCGAATGACTT
246PHI .         CGCGACACCACCCCTCTCCCCCC---TTT-GCGAACAGGCACCGGAGCACCGAATGACTT
51SA .           CGCGACACCACCCCTCTCCCCCC---TTT-GCGAACAGGCACCGGAGCACCGAATGACTT
2618ETHI .       CGCGACACCACCCCTCTCCCCCC---TTT-GCGAACAGGCACCGGAGCACCGAATGACTT
1068WIS .        CGCGACACCACCCCTCCCCCCC----TTTTGCGAACAGGCACCGGAGCATCGAATGACTT
238CR .          CGCGACACCACCCCTCTCCCCCC---TTT-GCGAACAGGCACCGGAGCACCGAATGACTT
NR5555 .         CGCGACACCACCCCTCTCCCCCC---TTT-GCGAACAGGCACCGGAGCACCGAATGACTT
NR6839 .         CGCGACACCACCCCTCTCCCCCC---TTT-GCGAACAGGCACCGGAGCACCGAATGACTT
2624ETHI .       CGCGACACCACCCCTCTCCCCCC---TTT-GCGAACAGGCACCGGAGCACCGAATGACTT
845EGY .         CGCGACACCACCCCTCTCCCCCC---TTT-GCGAACAGGCACCGGAGCACCGAATGACTT
JBPER12 .        CGCGACACCACCCCTCTCCCCC----TTTTGCGAACAGGCACCGGAGCACCGAATGACTT
1717VIET .       XXCGACAGCACCCCTCTCCCCC----TTTTGCGAACAGGCACCGGAGCACCGAATGACTT
709BRAZ .        CGCGACACCACCCCTCTCCCCC----TTTTGCGAACAGGCACCGGAGCACCGAATGACTT
878IRAN .        CGCGACACCACCCCTCTCCCCC----TATTGCGAACAGGCACCGGAGCACCGAATGACTT
239CR .          CGCGACACCACCCCTCTCCCCC----TTTTGCGAACAGGCACCGGAGCACCGAATGACTT
202COM .         CGCGATACCACCCCTCTCCCCC----TTTTGCGAACAGGCACCGGAGCACCGAATGACTT
1064MEX .        CGCGACACCACCCCTCTCCCCC----TTTTGCGAACAGGCACCGGAGCACCGAATGACTT
596BRAZ .        CGCGACACCACTCCTCTCCCCC----TTTTGCGAACAGGCACCGGAGCACCGAATGACTT
333USA .         CGCGACACCACCCCTCTCCCCC----TTTTGCGAACAGGCACCGGAGCACCGAATGACTT
2610ETHI .       CGCGACACCACCCCTCTCCCCC----TTTTGCGAACAGGCACCGGAGCACCGAATGACTT
1044RW .         CGCGACACCACCCCTCTCCCCCC---TTT-GCGAACAGGCACCGGAGCACCGAATGACTT
JBNZ111 .        CGCGACACCACCCCTCTCCCCC----TTTTGCGAACAGGCACCGGAGCACCGAATGACTT
JBNZ12 .         CGCGACACCACCCCTCTCCCCC----TATTGCGAACAGGCACCGGAGCACCGAATGACTT
JBNZ24 .         CGCGACACCACCCCTCTCCCCC----TATTGCGAACAGGCACCGGAGCACCGAATGACTT
693CHINA .       CTCGACACCACCCCTCTCCCCCC---TTT-GCGAACAGGCACCGGAGCACCGAATGACTT
1058RW .         CGCGACACCACCCCTCTCCCCCC---TTT-GCGAACAGGCACCGGAGCACCGAATGACTT
590AUS .         CGCGACACCACCCCTCTCCCCCC---TTT-GCGAATAGGCACCGGAGCACCGAATGACTT
53SA .           CGCGACACCACCCCTCTCCCCCC---TTT-GCGAACAGGCACCGGAGCACCGAATGACTT
1075WIS .        CGCGACACCACCCCTCTCCCCC----TTTTGCGAACAGGCACCGGAGCACCGAATGACTT
939GER .         CGCGACACCACCCCTCTCCCCC----TTTTGCGAACAGGCACCGGAGCACCGAATGACTT
GA3804 .         CGCGACACCACCCCTCTCCCCC----TTTTGCGAACAGGCACCGGAGCACCGAATGACTT
1599AT .         CGCGACACCACCCCTCTCCCCC----TTTTGCGAACAGGCACCGGAGCACCGAATGACTT
1941AT .         CGCGACACCACCCCTCTCCCCC----TTTTGCGAACAGGCACCGGAGCACCGAATGACTT
291NE .          CGCGACACCACCCCTCTCCCCC----TTTTGCGAACAGGCACCGGAGCACCGAATGACTT
2301SAR .        CGCGACACCACCCCTCTCCCCC----TTTTGCGAACAGGCACCGGAGCACCGAATGACTT
2313SAR .        CGCGACACCACCCCTCTCCCCC----TTTTGCGAACAGGCACCGGAGCACCGAATGACTT
271NE .          CGCGACACCACCCCTCTCCCCC----TTTTGCGAACAGGCACCGGAGCACCGAATGACTT
1935GER .        CGCGACACCACCCCTCTTCACC----TTTTACGAACAAACACCGGAGCACCGAATGACTT
JBRSA122 .       CGCGACACCACCCCTCTCCCCC----TTTTGCGAACAGGCACCGGAGCACCGAATGACTT
276NE .          CGCGACACCACCCCTCTCCCCC----TTTTGCGAACAGGCACCGGAGCACCGAATGACTT
272NE .          CGCGACACCACCCCTCTCCCCC----TTTTGCGAACAGGCACCGGAGCACCGAATGACTT
2673ETHI .       CGCGACACCACCCCTCTCCCCC----TTTTGCGAACAGGCACCGGAGCACCGAATGACTT
1084AUSW .       CGCGACACCACCCCTCTCCCCC----TTTTGCGAACAGGCACCGGAGCACCGAATGACTT
JBPER62 .        CGCGACACCACCCCTCTCCCCC----TTTTGCGAACAGGCACCGGAGCACCGAATGACTT
JBNZ72 .         CGCGACACCACCCCTCTCCCCC----TATTGCGAACAGGCACCGGAGCACCGAATGACTT
1065MEX .        CGCGACACCACCCCTCTCCCCCC---TTT-GCGAACAGGCACCGGAGCACCGAATGACTT
646HAWAI .       CGCGACACCACCCCTCTCCCCC----TTTTGCGAACAGGCACCGGAGCACCGAATGACTT
727INDIA .       CGCGACACCACCCCTCTCCCCC----TATTGCGAACAGGCACCGGAGCACCGAATGACTT
743MALE .        CGCGACACCACCCCTCTCCCCC----TATTGCGAACAGGCACCGGAGCACCGAATGACTT
1059MEX .        CGCGACACCACCCCTCTCCCCC----TATTGCGAACAGGCACCGGAGCACCGAATGACTT
1061RW .         CGCGACACCACCCCTCTCCCCCC---TTT-GCGAACAGGCACCGGAGCACCGAATGACTT
1066MEX .        CGCGACACCACTCCTC-CCCCC----TTTTGCGAATAGCTATCGGAGCACCGAATGACTT
1095BALI .       CGCGACACCACCCCTCCCTCC-----TTTTGCGAATAGGCACCGGAGCACCGAATGACTT
1505MAUR .       CGCGACACCACCCCTC-CCCCCC---TTT-GCGAACAGGCACCGGAGCACCGAATGACTT
1818ETHI .       CGCGACACCACCCCTCTCCCCC----TTTTGCGAACAGGCACTGGAGCACCGAATGACTT
1934AT .         CGCGACACCACCCCTCTCCCCC----TTTTGCGAACAGGCACCGGAGCACCGAATGACTT
245CR .          CGCGACACCACCCCTCCCTCC-----TTTTGCGAATAGGCACCGGAGCACCGAATGACTT
2710ETHI .       CGCGACACCACCCCTCCCTCC-----TTTTGCGAATAGGCACCGGAGCACCGAATGACTT
274NE .          CGCGACACCACCCCTCTCCCCC----TTTTGTGAATAGGCACCGGAGCACCGAATGACTT
3408PNG .        CGCGACACCACCCCTCTCCCCCC---TTTTGCGAACAGGCACCGGAGCACCGAATGACTG
3409PNG .        CGCGACACCACCCCTCTCCCCCC---TTTTGCGAACAGGCACCGGAGCACCGAATGACTG
410MALAY .       CGCGACACCACTCTTCCCCCCC----TTTTGCGAACAGGCACCAGAGCACCGAATAACTT
836EGY .         CGCGACACCACCCCTC-CTCCCC----TTTGCGAACAGGCACCGGAGCACCGAATGACTT
837EGY .         CGCGACACCACCCCTC-CTCCCC----TTTGCGAACAGGCACCGGAGCACCGAATGACTT
JBRO111 .        CGCGACACCACCCCTCTCCCCC----TTTTGCGAACAGGCACTGGAGCACCGAATGACTT
brunneoviridis4  CGCGACACCACCCCTCCCCCC-----TTTTGCGAATAGGCATCGGAGCACCGAATGACTT
brunneoviridis1  CGCGACACCACCCCTCCCCCCC----TTTTGCGAATAGGCATCGGGGCACCGAATGACTT
pleurotumCPK211  CGCGACACCACCCCTCCCCCCCCCC-TTTTGCGAATAGGCATCGAGGCACCCAATGACCT
PLEUROTICOLA .   CGCGACACCACCCCTCCCCCCC----TTTTGCGAATAGGCATCGGAGCACCGAATGACCT
epimycesCPK1981  CGCGACACCACCCCTCCCCCCC----TTTTGCGAATAGGCATCGGAGC-CCGAATGACTT
alniCPK3124 ...  CGCGACACCACCCCTCCCCCCC----TTTTGCGAATAGGCATCGGAACACCGAATGACTT
CPK5TAWA .       XXXXXXXXXXXXXXXXXXXXXXXXXXXXXXXXXXXXXXXXXXXXXXXXXXXXXXXXXXXX
2974lobau .      CGCGACACCACCCCTCCCCCCC----TTTTGCGAACAGGCACCGGAGCACCGAATGACTG
2975lobau .      CGCGACACCACCCCTCCCCCCC----TTTTGCGAACAGGCACCGGAGCACCGAATGACTG
aggressivumCPK3  CGCGACACCACCCCTCCCCTCC----TTTTGCGAATAGGCATCGGAACACCGAATGACTT
atrogelatinosaC  CGGGACCCCCCCCCTTTTTTT------TTTGCAAATAGGCATCGGAACACCGAATGACTT
```

```
atrogelatinosaC  CGCGACACCACTCTT-TCCCCCCCCC-TTTGCGAACAGGCACCGGAGCTCCGAATGACTT
cerinumCPK293 .  CGCGACACCA-TCCTCCCCCCC----TTTTGCGATCAGGCACCGGAGCGCCGAATGACTT
cerinumCPK854 .  CGCGACACCA-TCCTCCCCCCC----TTTTGCGATCAGGCACCGGAGCGCCGAATGACTT
tomentosumCPK88  CGCGACACCACCTTCCCCCCC----TTTTGCGAACAGGCACCGGAGCACCGAATGACTT
stramineaGJS028  CGCGACACCACCCCTCCCCCCC-----TTTGCGAACAGGCACCGGAGCACCGAATGACTT
velutinumCPK312  CGCGACACCACCCCTCCCCCC------TTTGCGAACAGGCACCGGAGCACCGAATGACTT
catoptronGJS027  CGCGACACAAGCCTTCCCCCCC----TTTTGCGAACAGGCACCAGAGCACCGAATGACTT
                 #####################    ################################


                        490       500       510       520       530       540
                 =========+=========+=========+=========+=========+=========+
1093CAN .        GCTGCCGGCCGATATTTT--CC-TTGCCAACCCCTTTTCATGATATCTGAGCAAA-GAAA
1052SA .         GCTGCCGGCCGATATTTT--CC-TTGCCAACCCCTTTTCATGATATCTGAGCAAA-GAAA
1116CAN .        GCTGCCGGCCGATATTTT--CC-TTGCCAACCCCTTTTCATGATATCTGAGCAAA-GAAA
1070EIRE .       GCTGCCGGCCGATATTTT--CC-TTGCCAACCCCTTTTCATGATATCTGAGCAAA-GAAA
1099CAN .        GCTGCCGGCCGATATTTT--CC-TTGCCAACCCCTTTTCATGATATCTGAGCAAA-GAAA
1104CAN .        GCTGCCGGCCGATATTTT--CC-TTGCCAACCCCTTTTCATGATATCTGAGCAAA-GAAA
1087CAN .        GCTGCCGGCCGATATTTT--CC-TTGCCAACCCCTTTTCATGATATCTGAGCAAA-GAAA
360UK .          GCTGCCGGCCGATATTTT--CC-TTGCCAACCCCTTTTCATGATATCTGAGCAAA-GAAA
2111HUN .        GCTGCCGGCCGATATTTT--CC-TTGCCAACCCCTTTTCATGATATCTGAGCAAA-GAAA
217RUS .         GCTGCCGGCCGATATTTT--CC-TTGCCAACCCCTTTTCATGATATCTGAGCAAA-GAAA
204UK .          GCTGCCGGCCGATATTTT--CC-TTGCCAACCCCTTTTCATGATATCTGAGCAAA-GAAA
206RUS .         GCTGCCGGCCGATATTTT--CC-TTGCCAACCCCTTTTCATGATATCTGAGCAAA-GAAA
261SIB .         GCTGCCGGCCGATATTTT--CC-TTGCCAACCCCTTTTCATGATATCTGAGCAAA-GAAA
265SIB .         GCTGCCGGCCGATATTTT--CC-TTGCCAACCCCTTTTCATGATATCTGAGCAAA-GAAA
2784THAI .       GCTGCCGACCGATATTTT--CC-TTGCTAGCCCCTTTTCATGATATCTGAACAAAAGGAA
838EGY .         GCTGCCGACCGATATTTT--CC-TTGCTAGCCCCTTTTCATGATATCTGAACAAAAGGAA
1102CAN .        GCTGCCGACCGATATTTT--CC-TGACCAACCCCTTTTCATGATCTCTGAGCAAA-G-AA
588LAOS .        GCTGCCGACCGATATCTT--CC-TTACCAGCCCCTTTTCATGAGATCTAAGCAAA-G-AA
1596AT .         GCTGCCGACCGATATTTC--CC-TTGCTAGCCCCTATTCACGATATCTTAGCAAAAG-AG
1720CAM .        GCTGCCGACCGATATTTT--CC-TTGCTAGCCCCTTTTCATGATATCTGAACAACAGGAA
1722CAM .        GCTGCCGACCGATATTTT--CC-TTGCTAGCCCCTTTTCATGATATCTGAACAACAGGAA
1724CAM .        GCTGCCGACCGATATTTT--CC-TTGCTAGCCCCTTTTCATGATATCTGAACAACAGGAA
334USA .         GCTGCCGACCGATATTTT--CC-TGACCAACCCCTTTTCATGATCTCTGAGCAAA-G-AA
335USA .         GCTGCCGACCGATATTTT--CC-TGACCAACCCCTTTTCATGATCTCTGAGCAAA-G-AA
1081WIS .        GCTGCCGACCGATATCTT--CC-TTGCCAACCCCTTTTCATGATATCTGAGC-AA-G-AA
1069WIS .        GCTGCCGACCGATATTTT--CC-TGACCAACCCCTTTTCATGATCTCTGAGCAAA-G-AA
1107CAN .        GCTGCCGACCGATATTTT--CC-TGACCAACCCCTTTTCATGATCTCTGAGCAAA-G-AA
1108PEN .        GCTGCCGACCGATAT-TT--CCCTTGCTAGCCCCTATTCACGATATCTTAGCAAAAG-AG
1110CAN .        GCTGCCGGCCGATATTTT--CC-TTGCCAACCCCTTTTCATGATATCTGAGCAAA-GAAA
JBSER241 .       GCTGCCGACCGATATTTA--CC-TTGCCAGCCCCTTTTCATGATATCTGAGCAAAAG-AA
246PHI .         GCTTCCGACCCATATCTT--CC-TTGACAACCCCTTTTCATGATATCTGAGCAAA-G-AA
51SA .           GCTTCCGACCCATATCTT--CC-TTGACAACCCCTTTTCATGATATCTGAGCAAA-G-AA
2618ETHI .       GCTTCCGACCCATATCTT--CC-TTGACAACCCCTTTTCATGATATCTGAGCAAA-G-AA
1068WIS .        GCTGCCGACCGATATTTT--CC-TGACCAACCCCTTTTCATGATCTCTGAGCAAA-G-AA
238CR .          GCTTCCGACCCATATCTT--CC-TTGACAACCCCTTTTCATGATATCTGAGCAAA-G-AA
NR5555 .         GCTTCCGACCCATATCTT--CC-TTGACAACCCCTTTTCATGATATCTGAGCAAA-G-AA
NR6839 .         GCTTCCGACCGATATTTT--CC-TTGCCAACCCCTTTTCATGATATCTGAGCAAA-G-AA
2624ETHI .       GCTTCCGACCGATATTTT--CC-TTGCCAACCCCTTTTCATGATATCTGAGCAAA-G-AA
845EGY .         GCTTCCGACCGATATTTT--CC-TTGCCAACCCCTTTTCATGATATCTGAGCAAA-G-AA
JBPER12 .        GCTGCCGACCGATATTTT--CC-TTGCTAGCCCCTTTTCATGATATCTGAACAAAAGGAA
1717VIET .       GCTGCCGACCGATATTTT--CC-TTGCTAGCCCCTTTTCATGATATCTGGACAAAAGGAA
709BRAZ .        GCTGCCGACCGATATTTT--CC-TTGCTAGCCCCTTTTCATGATATCTGAACAAAAGGAA
878IRAN .        GCTGCCGACCGATATTTA--CC-TTGCCAACCCCTTTTCATGATATCTGAGCGAA-G-AA
239CR .          GCTGCCGACCGATATTTT--CC-TTGCTAGCCCCCTTTCATGATATCTGAACAAAAGGAA
202COM .         GCTGCCGACCGATATTTT--CC-TTGCTAGCCCCTTTTCATGATATCTGAACAAAAGGAA
1064MEX .        GCTGCCGACCGATATTTT--CC-TTGCTAGCCCCCTTTCATGATATCTGAACAAAAGGAA
596BRAZ .        GCTGCCGACCGATATTTT--GC-TTGCCAGCCCCTTTTCATGATATCTGAGCAAA-G-AA
333USA .         GCTGCCGACCGATATTTT--CC-TTGCTAGCCCCTTTTCATGATATCTGAACAAAAGGAA
2610ETHI .       GCTGCCGACCGATATTTA--CC-TTGCCAGCCCCTTTTCATGATATCTGGGCAAAAG-AA
1044RW .         GCTGCCGACCGATATTTT--GC-TTGCCAGCCCCTTTTCATGATATTTGAGCAAA-G-AA
JBNZ111 .        GCTGCCGACCGATTTCTT--CC-TTACCAACCCCTTTTCATGATATTTGAGCAAA-G-AA
JBNZ12 .         GCTACCGACCGATAACTT--TC-TTGCCAACCCCTTTTCATGATATCTGAGCAAA-GGAA
JBNZ24 .         GCTACCGACCGATAACTT--TC-TTGCCAACCCCTTTTCATGATATCTGAGCAAA-GGAA
693CHINA .       GCTGCCGACCGATATTTT--GC-TTGCAAGCCCCTTTTCATGATATTTGAGCAAA-G-AA
1058RW .         GCTGCCGACCGATATTTT--GC-TTGCCAGCCCCTTTTCATGATATTTGAGCAAA-G-AA
590AUS .         GCTGCCGACCGATATTTT--CC-TAGCCAGCCCCTTTTCATGATATCTGAGCAAA-G-AA
53SA .           GCTGCCGACCGATATTTT--GC-TTGCCAGCCCCTTTTCATGATATTTGAGCAAA-G-AA
1075WIS .        GCTGCCGACCGATATTTA--CC-TTGCCAGCCCCTTTTCATGATATCTGAGCAAAAG-AA
939GER .         GCTGCCGACCGATATTTA--CC-TTGCCAGCCCCTTTTCATGATATCTGAGCAAAAG-AA
GA3804 .         GCTGCCGACCGATATTTA--CC-TTGCCAGCCCCTTTTCATGATATCTGAGCAAAAG-AA
1599AT .         GCTGCCGACCGATATTTA--CC-TTGCCAGCCCCTTTTCATGATATCTGAGCAAAAG-AA
1941AT .         GCTGCCGACCGATATTTA--CC-TTGCCAGCCCCTTTTCATGATATCTGAGCAAAAG-AA
291NE .          GCTGCCGACCGATATTTA--CC-TTGCCAGCCCCTTTTCATGATATCTGAGCAAAAG-AA
2301SAR .        GCTGCCGACCGATATTTA--CC-TTGCCAGCCCCTTTTCATGATATCTGAGCAAAAG-AA
2313SAR .        GCTGCCGACCGATATTTA--CC-TTGCCAGCCCCTTTTCATGATATCTGAGCAAAAG-AA
271NE .          GCTGCCGACCGATATTTA--CC-TTGCCAGCCCCTTTTCATGATATCTGAGCAAAAG-AA
1935GER .        GCTGCCGACCGATATTTA--CC-TTGCCAGCCCCTTTTCATGATATCTGAGCAAAAG-AA
JBRSA122 .       GCTGCCGACCGATATTTA--CC-TTGCCAGCCCCTTTTCATGATATCTGAGCAAAAG-AA
276NE .          GCTGCCGACCGATATTTA--CC-TTGCCAGCCCCTTTTCATGATATCTGAGCAAAAG-AA
272NE .          GCTGCCGACCGATATTTA--CC-TTGCCAGCCCCTTTTCATGATATCTGAGCAAAAG-AA
2673ETHI .       GCTGCCGACCGATATTTA--CC-TTGCCAGCCCCTTTTCATGATATCTGAGCAAAAG-AA
1084AUSW .       GCTGCCGACCGATATTTA--CC-TTGCCAGCCCCTTTTCATGATATCTGAGCAAAAG-AA
JBPER62 .        GCTGCCGACCGATATTTA--CC-TTGCCAGCCCCTTTTCATGATATCTGAGCAAAAG-AA
JBNZ72 .         GCTACCGACCGATAACTT--TC-TTGCCAACCCCTTTTCATGATATCTGAGCAAA-GGAA
1065MEX .        GCTGCCGACCGATATTTT--GC-TTGCCAGCCCCTTTTCATGATATTTGAGCAAA-G-AA
646HAWAI .       GCTGCCGACCGATATTTT--GC-TTGCCAGCCCCTTTTCATGATATCTGAGCAAA-G-AA
727INDIA .       GCTGCCGACCGATATTTA--CC-TTGCCAACCCCTTTTCATGATATCTGAGCGAA-G-AA
743MALE .        GCTGCCGACCGATATTTA--CC-TTGCCAACCCCTTTTCATGATATCTGAGCGAA-G-AA
1059MEX .        GCTGCCGACCGATATTTA--CC-TTGCCAACCCCTTTTCATGATATCTGAGCGAA-G-AA
1061RW .         GCTTCCGACCCATATCTT--CC-TTGACAACCCCTTTTCATGATATCTGAGCAAA-G-AA
1066MEX .        GCTGCCGACCGATATCTT--CC-TTACCAGCCCCTTTTCATGAGATCTAAGCAA--G-AA
1095BALI .       GCTGCCGACCGATATCTT--CC-TTGCCAGCCCCTTTTCATGAGATCTAAGCAAA-G-AA
1505MAUR .       GCTGCCGACCGATATTTT--CC-TTGCCAGCCCCTTTTCATGATATCTGAGCAAA-G-AA
1818ETHI .       GCTGCCGGCCGATATTTT--CC-TTGCCAACCCCTTTTCATGATATCTGAGCAAA-GAAA
1934AT .         GCTGCCGACCGATATCTA--CC-TTGCCAGCCCCTTTTCATGATATCTGAGCAAAAG-AA
245CR .          GCTGCCGACCGATATCTT--CC-TTGCCAGCCCCTTTTCATGAGATCTAAGCAAA-G-AA
2710ETHI .       GCTGCCGACCGATATCTT--CC-TTGCCAGCCCCTTTTCATGAGATCTAAGCAAA-G-AA
274NE .          GCTGCCGACCGATATCTT--CC-TTGCCAGCCCCTTTTCATGAGATCTAAGCAA-AG-AA
3408PNG .        GCTGCCGACCGATTTCTT--CC-TTGCCAGCCCCTTTTCATGATATCTGAGCAAA-G-AA
3409PNG .        GCTGCCGACCGATTTCTT--CC-TTGCCAGCCCCTTTTCATGATATCTGAGCAAA-G-AA
410MALAY .       GCTGCCGACCGAAATTTC--TC-TTGCCAGCCGGTTT-CTTGATATATGAGTAAA-GAAA
836EGY .         GCTGCCGACCGATATTTT--CC-TTGCCAGCCCCTTTTCATGATATCTAAGCAAA-G-AA
837EGY .         GCTGCCGACCGATATTTT--CC-TTGCCAGCCCCTTTTCATGATATCTAAGCAAA-G-AA
JBRO111 .        GCTGCCGGCCGATATTTT--CC-TTGCCAACCCCTTTTCATGATATCTGAGCAAA-GAAA
brunneoviridis4  GCTGCCGACCGATCTTTT--CC-TTGCCAGCCCGTTT-CATGATGGACGAGCAAA-GAAG
brunneoviridis1  GCTGCCGACCGATCTTTT--CC-TTGCCAGCCCGTTT-CATGATGGACGAGCAAA-GAAG
pleurotumCPK211  GCTGCCGACCGATA-TTT-CCC-TTGCCAGCCCCTTTCCATGATA---GA-CAAGCC-AA
PLEUROTICOLA .   GCTGCCGACCGATA-TTTCTC--TTGCCAGCCCCTTTCCATGATGGACGAGCAAA-G-AA
epimycesCPK1981  GCTGCCGACCGATATTTT-CCC-TTGCCAGCCCGTTT-CATGATAGACGAGCAA--G-AA
alniCPK3124 ...  GCT-TCGACCGATA-TTT-CCC-TTGCCAGCCCGTTTT-ATGATGGACGAGCAAA-G-AA
CPK5TAWA .       XXXXXXXXXXXXXXXXXXXXXXXXXXXXXXXXXXXXXXXXXXXXXXXXXGAGAAAAA-AAA
2974lobau .      GCTGCCGACCGATTTCTT--CC-TTGCCAGCCCCTTTTCATGATATCTGGGCAAA-G-AA
2975lobau .      GCTGCCGACCGATTTCTT--CC-TTGCCAGCCCCTTTTCATGATATCTGGGCAAA-G-AA
aggressivumCPK3  GCTGCCGACCGATAATTT-CCC-TTGTCAGCCCGTTT-CATGATAGGCGAGCAAA-G-AA
atrogelatinosaC  GCTGCCGACCGATA-TTT-CCC-TTGCTGGCCTGTTT-CGGGATAAACAAGCAAA---AA
```

```
atrogelatinosaC  GCTGCCGACCGATA-TTTTCC--TTGCCAGCCCCTTTTCATGATATCTGAAGAAAAG-AA
cerinumCPK293 .  GCTGCCGACCGACA-TTTTCA--TTGCCAGCCC-TTTTCATGATATATGGGCAAA-G-AA
cerinumCPK854 .  GCTGCCGACCGACA-TTTTCA--TTGCCAGCCC-TTTTCATGATATATGGGCAAA-G-AA
tomentosumCPK88  GCTGCCGACCGACA-TTTTCC--TTGCCAGCCC-TTT-CATGATATATGAGCAAA-G-AA
stramineaGJS028  GCTGCCGACCGATAATTT-CC--TTGCCAGCCCGTTT-GATGATAGATGAGTAAA-G-AA
velutinumCPK312  GCTGCCGACCGATCATTT-CC--TTGCCAGCCCGTTT-GATGATAGATGAGTAAA-G-AA
catoptronGJS027  GCTGCCGACCGATA-TTTACC--TTGCCGGCCCATTT-CATGATATATGGTCAAA-G-AA
                 ##################     ################################   ##


                        550       560       570       580       590       600
                 =========+=========+=========+=========+=========+=========+
1093CAN .        AAGAA-----CTGACAGGCTTGACCCCGCAGGCCAGATCACCACCAAGGAGCTGGGCACC
1052SA .         AAGAA-----CTGACAGGCTTGACCCCGCAGGCCAGATCACCACCAAGGAGCTGGGCACC
1116CAN .        AAGAA-----CTGACAGGCTTGACCCCGCAGGCCAGATCACCACCAAGGAGCTGGGCACC
1070EIRE .       AAGAA-----CTGACAGGCTTGACCCCGCAGGCCAGATCACCACCAAGGAGCTGGGCACC
1099CAN .        AAGAA-----CTGACAGGCTTGACCCCGCAGGCCAGATCACCACCAAGGAGCTGGGCACC
1104CAN .        AAGAA-----CTGACAGGCTTGACCCCGCAGGCCAGATCACCACCAAGGAGCTGGGCACC
1087CAN .        AAGAA-----CTGACAGGCTTGACCCCGCAGGCCAGATCACCACCAAGGAGCTGGGCACC
360UK .          AAGAA-----CTGACAGGCTTGACCCCGCAGGCCAGATCACCACCAAGGAGCTGGGCACC
2111HUN .        AAGAA-----CTGACAGGCTTGACCCCGCAGGCCAGATCACCACCAAGGAGCTGGGCACC
217RUS .         AAGAA-----CTGACAGGCTTGACCCCGCAGGCCAGATCACCACCAAGGAGCTGGGCACC
204UK .          AAGAA-----CTGACAGGCTTGACCCCGCAGGCCAGATCACCACCAAGGAGCTGGGCACC
206RUS .         AAGAA-----CTGACAGGCTTGACCCCGCAGGCCAGATCACCACCAAGGAGCTGGGCACC
261SIB .         AAGAA-----CTGACAGGCTTGACCCCGCAGGCCAGATCACCACCAAGGAGCTGGGCACC
265SIB .         AAGAA-----CTGACAGGCTTGACCCCGCAGGCCAGATCACCACCAAGGAGCTGGGCACC
2784THAI .       AA--------CTGACAGGCTTGACCCCGCAGGCCAGATCACCACCAAGGAGCTAGGCACC
838EGY .         ---AA-----CTGACAGGCTTGACCCCGCAGGCCAGATCACCACCAAGGAGCTGGGCACC
1102CAN .        AAGAA-----CTGACAGGCTTGACCCCGCAGGCCAGATCACCACCAAGGAGCTGGGCACC
588LAOS .        AAGAA-----CTGACAGGCTTGACCCCGCAGGCCAGATCACCACCAAGGAGCTGGGCACC
1596AT .         AA--A-----CTGACAGGCTTGACCCCGCAGGCCAGATCACCACCAAGGAGCTGGGCACC
1720CAM .        ---AA-----CTGACAGGCTTGACCCCGCAGGCCAGATCACCACCAAGGAGCTGGGCACC
1722CAM .        ---AA-----CTGACAGGCTTGACCCCGCAGGCCAGATCACCACCAAGGAGCTGGGCACC
1724CAM .        ---AA-----CTGACAGGCTTGACCCCGCAGGCCAGATCACCACCAAGGAGCTGGGCACC
334USA .         AAGAA-----CTGACAGGCTTGACCCCGCAGGCCAGATCACCACCAAGGAGCTGGGCACC
335USA .         AAGAA-----CTGACAGGCTTGACCCCGCAGGCCAGATCACCACCAAGGAGCTGGGCACC
1081WIS .        AAGAA-----CTGACAGGCTTGACCCCGCAGGCCAGATCACCACCAAGGAGCTGGGCACC
1069WIS .        AAGAA-----CTGACAGGCTTGACCCCGCAGGCCAGATCACCACCAAGGAGCTGGGCACC
1107CAN .        AAGAA-----CTGACAGGCTTGACCCCGCAGGCCAGATCACCACCAAGGAGCTGGGCACC
1108PEN .        A--AA-----CTGACAGGCTTGACCCCGCAGGCCAGATCACCACCAAGGAGCTGGGCACC
1110CAN .        AAGAA-----CTGACAGGCTTGACCCCGCAGGCCAGATCACCACCAAGGAGCTGGGCACC
JBSER241 .       AAGGA-----CTGACAAGCTTGACCCCGCAGGCCAGATCACCACCAAGGAGCTGGGCACC
246PHI .         ACAAA-----CTGACAGGCTTGACCCCGCAGGCCAGATCACCACCAAGGAGCTGGGCACC
51SA .           ACGAA-----CTGACAGGCTTGACCCCGCAGGCCAGATCACCACCAAGGAGCTGGGCACC
2618ETHI .       ACGAA-----CTGACAGGCTTGACCCCGCAGGCCAGATCACCACCAAGGAGCTGGGCACC
1068WIS .        AAGAA-----CTGACAGGCTTGACCCCGCAGGCCAGATCACCACCAAGGAGCTGGGCACC
238CR .          ACGAA-----CTGACAGGCTTGACCCCGCAGGCCAGATCACCACCAAGGAGCTGGGCACC
NR5555 .         ACGAA-----CTGACAGGCTTGACCCCGCAGGCCAGATCACCACCAAGGAGCTGGGCACC
NR6839 .         ACGAA-----CTGACAGGCTTGACCCCGCAGGCCAGATCACCACCAAGGAGCTGGGCACC
2624ETHI .       ACGAA-----CTGACAGGCTTGACCCCGCAGGCCAGATCACCACCAAGGAGCTGGGCACC
845EGY .         ACGAA-----CTGACAGGCTTGACCCCGCAGGCCAGATCACCACCAAGGAGCTGGGCACC
JBPER12 .        ---AA-----CTGACAGGCTTGACCCCGCAGGCCAGATCACCACCAAGGAGCTGGGCACC
1717VIET .       ---AA-----CTGACAGGCTTGACCCCGCAGGCCAGATCACCACCAAGGAGCTGGGCACC
709BRAZ .        ---AA-----CTGACAGGCTTGACCCCGCAGGCCAGATCACCACCAAGGAGCTGGGCACC
878IRAN .        AAGAA-----CTGACAGGCTTGACCCCGCAGGCCAGATCACCACCAAGGAGCTGGGCACC
239CR .          ---AA-----CTGACAGGCTTGACCCCGCAGGCCAGATCACCACCAAGGAGCTGGGCACC
202COM .         ---AA-----CTGACAGGCTTGACCCCACAGGCCAGATCACGACCAAGGAGCTGGGCACC
1064MEX .        ---AA-----CTGACAGGCTTGACCCCGCAGGCCAGATCACCACCAAGGAGCTGGGCACC
596BRAZ .        ACGAA-----CTGACAGGCTTGACCCCGCAGGCCAGATCACCACCAAGGAGCTGGGCACC
333USA .         AA--------CTGACAGGCTTGACCCCGCAGGCCAGATCACCACCAAGGAGCTGGGCACC
2610ETHI .       AAGAA-----CTGACAGGCTTGACCCCGCAGGCCAGATCACCACCAAGGAGCTGGGCACC
1044RW .         AAGAA-----CTGACAGGCTTGACCCCGCAGGCCAGATCACCACCAAGGAGCTGGGCACC
JBNZ111 .        AGGAA-----CTGACAGACTTGACCCCGCAGGCCAGATCACCACCAAGGAGCTGGGCACC
JBNZ12 .         A-GAA-----CTGACAGGCTTGACCCCGCAGGCCAGATCACCACCAAGGAGCTGGGCACC
JBNZ24 .         A-GAA-----CTGACAGGCTTGACCCCGCAGGCCAGATCACCACCAAGGAGCTGGGCACC
693CHINA .       AAGAA-----CTGACAGGCTTGACCCCGCAGGCCAGATCACCACCAAGGAGCTGGGCACC
1058RW .         AAGAA-----CTGACAGGCTTGACCCCGCAGGCCAGATCACCACCAAGGAGCTGGGCACC
590AUS .         AAGAA-----CTGACAGGCTTGACCCCGCAGGCCAGATCACCACCAAGGAGCTGGGCACC
53SA .           AAGAA-----CTGACAGGCTTGACCCCGCAGGCCAGATCACCACCAAGGAGCTGGGCACC
1075WIS .        AAGAA-----CTGACAGGCTTGACCCCGCAGGCCAGATCACCACCAAGGAGCTGGGCACC
939GER .         AAGAA-----CTGACAGGCTTGACCCCGCAGGCCAGATCACCACCAAGGAGCTGGGCACC
GA3804 .         AAGAA-----CTGACAGGCTTGACCCCGCAGGCCAGATCACCACCAAGGAGCTGGGCACC
1599AT .         AAGGA-----CTGACAAGCTTGACCCCGCAGGCCAGATCACCACCAAGGAGCTGGGCACC
1941AT .         AAGGA-----CTGACAAGCTTGACCCCGCAGGCCAGATCACCACCAAGGAGCTGGGCACC
291NE .          AAGAA-----CTGACAGGCTTXXXXXXXXXXXXXXXXXXXXXXXXXXXXXXXXXXXXXXX
2301SAR .        AAGGA-----CTGACAAGCTTGACCCCGCAGGCCAGATCACCACCAAGGAGCTGGGCACC
2313SAR .        AAGGA-----CTGACAAGCTTGACCCCGCAGGCCAGATCACCACCAAGGAGCTGGGCACC
271NE .          AAGAA-----CTGACAGGCTTGACCCCGCAGGCCAGATCACCACCAAGGAGCTGGGCACC
1935GER .        AAGAA-----CTGACAGGCTTGACCCCGCAGGCCAGATCACCACCAAGGAGCTGGGCACC
JBRSA122 .       AAGGA-----CTGACAAGCTTGACCCCGCAGGCCAGATCACCACCAAGGAGCTGGGCACC
276NE .          AAGAA-----CTGACAGGCTTGACCCCGCAGGCCAGATCACCACCAAGGAGCTGGGCACC
272NE .          AAGAA-----CTGACAGGCTTGACCCCGCAGGCCAGATCACCACCAAGGAGCTGGGCACC
2673ETHI .       AAGAA-----CTGACAGGCTTGACCCCGCAGGCCAGATCACCACCAAGGAGCTGGGCACC
1084AUSW .       AAGAA-----CTGACAGGCTTGACCCCGCAGGCCAGATCACCACCAAGGAGCTGGGCACC
JBPER62 .        AAGAA-----CTGACAGGCTTGACCCCGCAGGCCAGATCACCACCAAGGAGCTGGGCACC
JBNZ72 .         A-GAA-----CTGACAGGCTTGACCCCGCAGGCCAGATCACCACCAAGGAGCTGGGCACC
1065MEX .        AAGAA-----CTGACAGGCTTGACCCCGCAGGCCAGATCACCACCAAGGAGCTGGGCACC
646HAWAI .       ACGAA-----CTAACCGGCTTGACCCCGCAGGCCAGATCACCACCAAGGAGCTGGGCACC
727INDIA .       AAGAA-----CTGACAGGCTTGACCCCGCAGGCCAGATCACCACCAAGGAGCTGGGCACC
743MALE .        AAGAA-----CTGACAGGCTTGACCCCGCAGGCCAGATCACCACCAAGGAGCTGGGCACC
1059MEX .        AAGAA-----CTGACAGGCTTGACCCCGCAGGCCAGATCACCACCAAGGAGCTGGGCACC
1061RW .         ACGAA-----CTGACAGGCTTGACCCCGCAGGCCAGATCACCACCAAGGAGCTGGGCACC
1066MEX .        AAGAA-----CTGACAGGCTTGACCCCGCAGGCCAGATCACCACCAAGGAGCTGGGCACC
1095BALI .       AAGAA-----CTGACAGGCTTGACCCCGCAGGCCAGATCACCACCAAGGAGCTGGGCACC
1505MAUR .       AGGAA-----CTGACAGGCTTGACCCCGCAGGCCAGATCACCACCAAGGAGCTGGGCACC
1818ETHI .       AAGAA-----CTGACAGGCTTGACCCCGCAGGCCAGATCACCACCAAGGAGCTGGGCACC
1934AT .         AAGAA-----CTGACAGGCTTGACCCCGCAGGCCAGATCACCACCAAGGAGCTGGGCACC
245CR .          AAGAA-----CTGACAGGCTTGACCCCGCAGGCCAGATCACCACCAAGGAGCTGGGCACC
2710ETHI .       AAGAA-----CTGACAGGCTTGACCCCGCAGGCCAGATCACCACCAAGGAGCTGGGCACC
274NE .          AAGAA-----CTAACAGGCTTGACCCCGCAGGCCAGATCACCACCAAGGAGCTGGGCACC
3408PNG .        AAGAA-----CTGACAGGCTTGACCCCGCAGGCCAGATCACCACCAAGGAGCTGGGCACC
3409PNG .        AAGAA-----CTGACAGGCTTGACCCCGCAGGCCAGATCACCACCAAGGAGCTGGGCACC
410MALAY .       AA-AA-----CTGACAGGCTTGACCCCGCAGGCCAGATCACCACCAAGGAGCTGGGCACC
836EGY .         AGGAA-----CTGACAGGCTTGACCCCGCAGGCCAGATCACCACCAAGGAGCTGGGCACC
837EGY .         AGGAA-----CTGACAGGCTTGACCCCGCAGGCCAGATCACCACCAAGGAGCTGGGCACC
JBRO111 .        AAGAA-----CTGACAGGCTTGACCCCGCAGGCCAGATCACCACCAAGGAGCTGGGCACC
brunneoviridis4  AAGAAAAAAACTGACAGGCTTGACCCCGCAGGCCAGATCACCACCAAGGAGCTGGGCACC
brunneoviridis1  AAGAAAAAAACTGACAGGCTTGACCCCGCAGGCCAGATCACCACCAAGGAGCTGGGCACC
pleurotumCPK211  AAAAAAAA--CTGACAGGCTTGACCCCCCAGGCCAAATCACCCCCAAGGAGCTGGGCACC
PLEUROTICOLA .   AAGAA-----CTGACAGGCTTGACCCCGCAGGCCAGATCACCACCAAGGAGCTGGGTACC
epimycesCPK1981  AAAAA-----CTGACAGGCTTGAACCCGCAGGCCAGATCACCACCAAGGAGCTGGGCACC
alniCPK3124 ...  AAAAAAAA--CTGACAGGCTTGACCCCGCAGGCCAAATCACCACCAAGGAGCTGGGCACC
CPK5TAWA .       AAAAAAA---CTGACAGGCTTGACCCCGCAGGCCAGATCACCACCAAGGAGCTGGGCACC
2974lobau .      AAGAA-----CTGACAGGCTTGACCCCGCAGGCCAGATCACCACCAAGGAGCTGGGCACC
2975lobau .      AAGAA-----CTGACAGGCTTGACCCCGCAGGCCAGATCACCACCAAGGAGCTGGGCACC
aggressivumCPK3  GAATA-----CTGACAGGCTTGACCCCGCAGGCCAGATCACCACCAAGGAGCTGGGCACC
atrogelatinosaC  AAAAAA----CTGACAGGCTTGACCCCGCAGGCCAGATTACCACCAAGGAGCTGGGCACC
```

```
atrogelatinosaC  AAA-------CTGACAGGCTTGACCCCGCAGGCCAGATCACCACCAAGGAGCTGGGTACC
cerinumCPK293 .  GAAAAA----CTGACAGGCTTGACCCCGCAGGCCAGATCACCACCAAGGAGCTGGGCACC
cerinumCPK854 .  GAAAAA----CTGACAGGCTTGACCCCGCAGGCCAGATCACCACCAAGGAGCTGGGCACC
tomentosumCPK88  AAAAA-----CTGACAGGCTTGACCCCGCAGGCCAGATCACCACCAAGGAGCTGGGCACC
stramineaGJS028  AAAAA-----CTGACAGGCTTGACCCCGCAGGCCAGATCACCACCAAGGAGCTGGGCACC
velutinumCPK312  AAAAAA----CTGACAGGCTTGACCCCGCAGGCCAGATCACCACCAAGGAGCTGGGCACC
catoptronGJS027  AAAAAAA---CTGACAGGCTTGACCCCGCAGGCCAGATCACCACCAAGGAGCTGGGCACT
                 #####     ##################################################


                        610       620       630       640       650       660
                 =========+=========+=========+=========+=========+=========+
1093CAN .        GTGATGCGCTCCCTCGGCCAGAACCCCTCCGAGTCAGAGCTCCAGGACATGATCAACGAG
1052SA .         GTGATGCGCTCCCTCGGCCAGAACCCCTCCGAGTCAGAGCTCCAGGACATGATCAACGAG
1116CAN .        GTGATGCGCTCCCTCGGCCAGAACCCCTCCGAGTCAGAGCTCCAGGACATGATCAACGAG
1070EIRE .       GTGATGCGCTCCCTCGGCCAGAACCCCTCCGAGTCAGAGCTCCAGGACATGATCAACGAG
1099CAN .        GTGATGCGCTCCCTCGGCCAGAACCCCTCCGAGTCAGAGCTCCAGGACATGATCAACGAG
1104CAN .        GTGATGCGCTCCCTCGGCCAGAACCCCTCCGAGTCAGAGCTCCAGGACATGATCAACGAG
1087CAN .        GTGATGCGCTCCCTCGGCCAGAACCCCTCCGAGTCAGAGCTCCAGGACATGATCAACGAG
360UK .          GTGATGCGCTCCCTCGGCCAGAACCCCTCCGAGTCAGAGCTCCAGGACATGATCAACGAG
2111HUN .        GTGATGCGCTCCCTCGGCCAGAACCCCTCCGAGTCAGAGCTCCAGGACATGATCAACGAG
217RUS .         GTGATGCGCTCCCTCGGCCAGAACCCCTCCGAGTCAGAGCTCCAGGACATGATCAACGAG
204UK .          GTGATGCGCTCCCTCGGCCAGAACCCCTCCGAGTCAGAGCTCCAGGACATGATCAACGAG
206RUS .         GTGATGCGCTCCCTCGGCCAGAACCCCTCCGAGTCAGAGCTCCAGGACATGATCAACGAG
261SIB .         GTGATGCGCTCCCTCGGCCAGAACCCCTCCGAGTCAGAGCTCCAGGACATGATCAACGAG
265SIB .         GTGATGCGCTCCCTCGGCCAGAACCCCTCCGAGTCAGAGCTCCAGGACATGATCAACGAG
2784THAI .       GTGATGCGCTCTCTCGGCCAGAACCCCTCCGAGTCAGAGCTTCAGGACATGATCAACGAG
838EGY .         GTGATGCGCTCTCTCGGCCAGAACCCCTCCGAGTCAGAGCTTCAGGACATGATCAACGAG
1102CAN .        GTGATGCGCTCCCTCGGCCAGAACCCCTCCGAGTCAGAGCTTCAGGATATGATCAACGAG
588LAOS .        GTGATGCGCTCCCTCGGCCAGAACCCCTCCGAGTCAGAGCTGCAGGACATGATCAACGAG
1596AT .         GTGATGCGCTCTCTCGGCCAGAACCCCTCCGAGTCAGAGCTTCAGGACATGATCAACGAG
1720CAM .        GTGATGCGCTCTCTCGGCCAGAACCCCTCCGAGTCAGAGCTTCAGGACATGATCAACGAG
1722CAM .        GTGATGCGCTCTCTCGGCCAGAACCCCTCCGAGTCAGAGCTTCAGGACATGATCAACGAG
1724CAM .        GTGATGCGCTCTCTCGGCCAGAACCCCTCCGAGTCAGAGCTTCAGGACATGATCAACGAG
334USA .         GTGATGCGCTCCCTCGGCCAGAACCCCTCCGAGTCAGAGCTCCAGGATATGATCAACGAG
335USA .         GTGATGCGCTCCCTCGGCCAGAACCCCTCCGAGTCAGAGCTTCAGGATATGATCAACGAG
1081WIS .        GTGATGCGCTCCCTCGGCCAGAACCCCTCCGAGTCAGAGCTTCAGGACATGATCAACGAG
1069WIS .        GTGATGCGCTCCCTCGGCCAGAACCCCTCCGAGTCAGAGCTTCAGGATATGATCAACGAG
1107CAN .        GTGATGCGCTCCCTCGGCCAGAACCCCTCCGAGTCAGAGCTTCAGGATATGATCAACGAG
1108PEN .        GTGATGCGCTCTCTCGGCCAGAACCCCTCCGAGTCAGAGCTTCAGGACATGATCAACGAG
1110CAN .        GTGATGCGCTCCCTCGGCCAGAACCCCTCCGAGTCAGAGCTCCAGGACATGATCAACGAG
JBSER241 .       GTGATGCGCTCTCTCGGCCAGAACCCCTCCGAGTCAGAGCTTCAGGACATGATCAACGAG
246PHI .         GTGATGCGCTCCCTCGGCCAGAACCCCTCCGAGTCAGAGCTGCAGGACATGATTAACGAG
51SA .           GTGATGCGCTCCCTCGGCCAGAACCCCTCCGAGTCAGAGCTGCAGGACATGATTAACGAG
2618ETHI .       GTGATGCGCTCCCTCGGCCAGAACCCCTCCGAGTCAGAGCTGCAGGACATGATTAACGAG
1068WIS .        GTGATGCGCTCCCTCGGCCAGAACCCCTCCGAGTCAGAGCTTCAGGATATGATCAACGAG
238CR .          GTGATGCGCTCCCTCGGCCAGAACCCCTCCGAGTCAGAGCTGCAGGACATGATTAACGAG
NR5555 .         GTGATGCGCTCCCTCGGCCAGAACCCCTCCGAGTCAGAGCTGCAGGACATGATTAACGAG
NR6839 .         GTGATGCGCTCCCTCGGCCAGAACCCCTCCGAGTCAGAGCTGCAGGACATGATTAACGAG
2624ETHI .       GTGATGCGCTCCCTCGGCCAGAACCCCTCCGAGTCAGAGCTGCAGGACATGATTAACGAG
845EGY .         GTGATGCGCTCCCTCGGCCAGAACCCCTCCGAGTCAGAGCTGCAGGACATGATTAACGAG
JBPER12 .        GTGATGCGCTCTCTCGGCCAGAACCCCTCCGAGTCAGAGCTTCAGGACATGATCAACGAG
1717VIET .       GTGATGCGCTCTCTCGGCCAGAACCCCTCCGAGTCAGAGCTTCAGGACATGATCAACGAG
709BRAZ .        GTGATGCGCTCTCTCGGCCAGAACCCCTCCGAGTCAGAGCTTCAGGACATGATCAACGAG
878IRAN .        GTGATGCGCTCTCTCGGCCAGAACCCCTCCGAGTCAGAGCTGCAGGACATGATCAACGAG
239CR .          GTGATGCGCTCTCTCGGCCAGAACCCCTCCGAGTCAGAGCTTCAGGACATGATCAACGAG
202COM .         GTGATGCGCTCTCTCGGCCAGAACCCCTCCGAGTCAGAGCTTCAGGACATGATCAACGAG
1064MEX .        GTGATGCGCTCTCTCGGCCAGAACCCCTCCGAGTCAGAGCTTCAGGACATGATCAACGAG
596BRAZ .        GTGATGCGCTCCCTCGGCCAGAACCCCTCCGAGTCAGAGCTGCAGGACATGATCAACGAG
333USA .         GTGATGCGCTCTCTCGGCCAGAACCCCTCCGAGTCAGAGCTTCAGGACATGATCAACGAG
2610ETHI .       GTGATGCGCTCCCTCGGCCAGAACCCCTCCGAGTCAGAGCTGCAGGACATGATCAACGAG
1044RW .         GTGATGCGCTCCCTCGGCCAGAACCCCTCCGAGTCAGAGCTGCAGGACATGATCAACGAG
JBNZ111 .        GTGATGCGCTCCCTCGGCCAGAACCCCTCCGAGTCAGAGCTGCAGGACATGATCAACGAG
JBNZ12 .         GTGATGCGCTCCCTCGGCCAGAACCCCTCCGAGTCAGAGCTGCAGGACATGATCAACGAG
JBNZ24 .         GTGATGCGCTCCCTCGGCCAGAACCCCTCCGAGTCAGAGCTGCAGGACATGATCAACGAG
693CHINA .       GTGATGCGCTCTCTCGGCCAGAACCCCTCCGAGTCAGAGCTTCAGGACATGATCAACGAG
1058RW .         GTGATGCGCTCCCTCGGCCAGAACCCCTCCGAGTCAGAGCTGCAGGACATGATCAACGAG
590AUS .         GTGATGCGCTCCCTCGGCCAGAACCCCTCCGAGTCAGAGCTTCAGGACATGATCAACGAG
53SA .           GTGATGCGCTCCCTCGGCCAGAACCCCTCCGAGTCAGAGCTGCAGGACATGATCAACGAG
1075WIS .        GTGATGCGCTCCCTCGGCCAGAACCCCTCCGAGTCAGAGCTGCAGGACATGATCAACGAG
939GER .         GTGATGCGCTCCCTCGGCCAGAACCCCTCCGAGTCAGAGCTGCAGGACATGATCAACGAG
GA3804 .         GTGATGCGCTCCCTCGGCCAGAACCCCTCCGAGTCAGAGCTGCAGGACATGATCAACGAG
1599AT .         GTGATGCGCTCTCTCGGCCAGAACCCCTCCGAGTCAGAGCTTCAGGACATGATCAACGAG
1941AT .         GTGATGCGCTCTCTCGGCCAGAACCCCTCCGAGTCAGAGCTTCAGGACATGATCAACGAG
291NE .          XXXXXXXXXXXXXXXXXXXXXXXXXXXXXXXXXXXXXXXXXXXXXXXXXXXXXXXXXXXX
2301SAR .        GTGATGCGCTCTCTCGGCCAGAACCCCTCCGAGTCAGAGCTTCAGGACATGATCAACGAG
2313SAR .        GTGATGCGCTCTCTCGGCCAGAACCCCTCCGAGTCAGAGCTTCAGGACATGATCAACGAG
271NE .          GTGATGCGCTCCCTCGGCCAGAACCCCTCCGAGTCAGAGCTGCAGGACATGATCAACGAG
1935GER .        GTGATGCGCTCCCTCGGCCAGAACCCCTCCGAGTCAGAGCTGCAGGACATGATCAACGAG
JBRSA122 .       GTGATGCGCTCTCTCGGCCAGAACCCCTCCGAGTCAGAGCTTCAGGACATGATCAACGAG
276NE .          GTGATGCGCTCCCTCGGCCAGAACCCCTCCGAGTCAGAGCTGCAGGACATGATCAACGAG
272NE .          GTGATGCGCTCCCTCGGCCAGAACCCCTCCGAGTCAGAGCTGCAGGACATGATCAACGAG
2673ETHI .       GTGATGCGCTCCCTCGGCCAGAACCCCTCCGAGTCAGAGCTGCAGGACATGATCAACGAG
1084AUSW .       GTGATGCGCTCCCTCGGCCAGAACCCCTCCGAGTCAGAGCTGCAGGACATGATCAACGAG
JBPER62 .        GTGATGCGCTCCCTCGGCCAGAACCCCTCCGAGTCAGAGCTGCAGGACATGATCAACGAG
JBNZ72 .         GTGATGCGCTCCCTCGGCCAGAACCCCTCCGAGTCAGAGCTGCAGGACATGATCAACGAG
1065MEX .        GTGATGCGCTCCCTCGGCCAGAACCCCTCCGAGTCAGAGCTGCAGGACATGATCAACGAG
646HAWAI .       GTGATGCGCTCCCTCGGCCAGAACCCCTCCGAGTCAGAGCTGCAGGACATGATCAACGAG
727INDIA .       GTGATGCGCTCTCTCGGCCAGAACCCCTCCGAGTCAGAGCTGCAGGACATGATCAACGAG
743MALE .        GTGATGCGCTCTCTCGGCCAGAACCCCTCCGAGTCAGAGCTGCAGGACATGATCAACGAG
1059MEX .        GTGATGCGCTCTCTCGGCCAGAACCCCTCCGAGTCAGAGCTGCAGGACATGATCAACGAG
1061RW .         GTGATGCGCTCCCTCGGCCAGAACCCCTCCGAGTCAGAGCTGCAGGACATGATTAACGAG
1066MEX .        GTGATGCGCTCCCTCGGCCAGAACCCCTCCGAGTCAGAGCTGCAGGACATGATCAACGAG
1095BALI .       GTGATGCGCTCCCTCGGCCAGAACCCCTCCGAGTCAGAGCTGCAGGACATGATCAACGAG
1505MAUR .       GTGATGCGCTCCCTCGGCCAGAACCCCTCCGAGTCAGAGCTGCAGGACATGATCAACGAG
1818ETHI .       GTGATGCGCTCCCTCGGCCAGAACCCCTCCGAGTCAGAGCTCCAGGACATGATCAACGAG
1934AT .         GTGATGCGCTCTCTCGGCCAGAACCCCTCCGAGTCAGAGCTTCAGGACATGATCAACGAG
245CR .          GTGATGCGCTCCCTCGGCCAGAACCCCTCCGAGTCAGAGCTGCAGGACATGATCAACGAG
2710ETHI .       GTGATGCGCTCCCTCGGCCAGAACCCCTCCGAGTCAGAGCTGCAGGACATGATCAACGAG
274NE .          GTGATGCGCTCCCTCGGCCAGAACCCCTCCGAGTCAGAGCTGCAGGACATGATCAACGAG
3408PNG .        GTGATGCGCTCCCTCGGCCAGAACCCCTCCGAGTCAGAGCTGCAGGACATGATCAACGAG
3409PNG .        GTGATGCGCTCCCTCGGCCAGAACCCCTCCGAGTCAGAGCTGCAGGACATGATCAACGAG
410MALAY .       GTCATGCGCTCTCTCGGCCAGAACCCCTCCGAGTCAGAGCTGCAGGACATGATCAACGAG
836EGY .         GTGATGCGCTCCCTCGGCCAGAACCCCTCCGAGTCAGAGCTTCAGGACATGATCAACGAG
837EGY .         GTGATGCGCTCCCTCGGCCAGAACCCCTCCGAGTCAGAGCTTCAGGACATGATCAACGAG
JBRO111 .        GTGATGCGCTCCCTCGGCCAGAACCCCTCCGAGTCAGAGCTCCAGGACATGATCAACGAG
brunneoviridis4  GTGATGCGCTCTCTCGGCCAGAACCCCTCCGAGTCAGAGCTGCAGGACATGATCAACGAG
brunneoviridis1  GTGATGCGCTCTCTCGGCCAGAACCCCTCCGAGTCAGAGCTGCAGGACATGATCAACGAG
pleurotumCPK211  GTGATGCGCTCTCTTGGCCAGAACCCCTCCGAGTCAAAGCTGCGGGACATGATCAACGAG
PLEUROTICOLA .   GTGATGCGCTCTCTTGGCCAGAACCCCTCCGAGTCAGAGCTGCAGGACATGATCAACGAG
epimycesCPK1981  GTGATGCGCTCTCTTGGTCAGAACCCCTCCGAGTCAGAGCTTCAGGACATGATCAACGAG
alniCPK3124 ...  GTGATGCGCTCTCTTGGCCAGAACCCCTCCGAGTCAGAGCTTCAGGACATGATCAACGAG
CPK5TAWA .       GTGATGCGCTCTCTTGGCCAGAACCCCTCCGAGTCAGAGCTTCAGGACATGATCAACGAG
2974lobau .      GTGATGCGCTCTCTCGGCCAGAACCCCTCCGAGTCAGAGCTTCAGGACATGATCAACGAG
2975lobau .      GTGATGCGCTCTCTCGGCCAGAACCCCTCCGAGTCAGAGCTTCAGGACATGATCAACGAG
aggressivumCPK3  GTGATGCGCTCTCTTGGCCAGAACCCCTCCGAGTCAGAGCTTCAGGACATGATCAACGAG
atrogelatinosaC  GGGATGCGCTCTCTTGGACAAAACCCCTCCAAGTCAAAGCTTCAGGACATGATCAACGAG
```

```
atrogelatinosaC  GTGATGCGCTCCCTCGGCCAGAACCCCTCCGAGTCAGAGCTGCAGGACATGATCAACGAG
cerinumCPK293 .  GTGATGCGCTCTCTCGGCCAGAACCCCTCCGAGTCAGAGCTTCAGGACATGATCAACGAG
cerinumCPK854 .  GTGATGCGCTCTCTCGGCCAGAACCCCTCCGAGTCAGAGCTTCAGGACATGATCAACGAG
tomentosumCPK88  GTGATGCGCTCTCTCGGCCAGAACCCCTCCGAGTCAGAGCTACAGGACATGATCAACGAG
stramineaGJS028  GTGATGCGCTCTCTCGGCCAGAACCCCTCCGAGTCAGAGCTGCAGGACATGATCAACGAG
velutinumCPK312  GTGATGCGCTCTCTCGGCCAGAACCCCTCCGAGTCAGAGCTGCAGGACATGATCAACGAG
catoptronGJS027  GTGATGCGCTCTCTCGGCCAGAACCCCTCCGAGTCAGAGCTGCAGGACATGATTAACGAG
                 ############################################################


                        670       680       690       700       710       720
                 =========+=========+=========+=========+=========+=========+
1093CAN .        GTCGATGCCGACAACAACGGATCTATCGATTTCCCCGGTATGTCAAAA-GTCAAAGTGTT
1052SA .         GTCGATGCCGACAACAACGGATCTATCGATTTCCCCGGTATGTCAAAA-GTCAAAGTGTT
1116CAN .        GTCGATGCCGACAACAACGGATCTATCGATTTCCCCGGTATGTCAAAA-GTCAAAGTGTT
1070EIRE .       GTCGATGCCGACAACAACGGATCTATCGATTTCCCCGGTATGTCAAAA-GTCAAAGTGTT
1099CAN .        GTCGATGCCGACAACAACGGATCTATCGATTTCCCCGGTATGTCAAAA-GTCAAAGTGTT
1104CAN .        GTCGATGCCGACAACAACGGATCTATCGATTTCCCCGGTATGTCAAAA-GTCAAAGTGTT
1087CAN .        GTCGATGCCGACAACAACGGATCTATCGATTTCCCCGGTATGTCAAAA-GTCAAAGTGTT
360UK .          GTCGATGCCGACAACAACGGATCTATCGATTTCCCCGGTATGTCAAAA-GTCAAAGTGTT
2111HUN .        GTCGATGCCGACAACAACGGATCTATCGATTTCCCCGGTATGTCAAAA-GTCAAAGTGTT
217RUS .         GTCGATGCCGACAACAACGGATCTATCGATTTCCCCGGTATGTCAAAA-GTCAAAGTGTT
204UK .          GTCGATGCCGACAACAACGGATCTATCGATTTCCCCGGTATGTCAAAA-GTCAAAGTGTT
206RUS .         GTCGATGCCGACAACAACGGATCTATCGATTTCCCCGGTATGTCAAAA-GTCAAAGTGTT
261SIB .         GTCGATGCCGACAACAACGGATCTATCGATTTCCCCGGTATGTCAAAA-GTCAAAGTGTT
265SIB .         GTCGATGCCGACAACAACGGATCTATCGATTTCCCCGGTATGTCAAAA-GTCAAAGTGTT
2784THAI .       GTCGATGCTGACAACAACGGATCTATCGATTTCCCCGGTATGTCAAAA-GTCAAAGTGTT
838EGY .         GTCGATGCCGACAACAACGGATCTATCGATTTCCCCGGTATGTCAAAA-GTCAAAGTGT-
1102CAN .        GTCGATGCCGACAACAACGGATCTATCGATTTCCCCGGTATGTCAAAA-GTCAAAGTATT
588LAOS .        GTCGATGCCGACAACAACGGATCTATCGATTTCCCCGGTATGTCAATA-GTCGAAGTATG
1596AT .         GTCGATGCCGACAACAACGGATCTATCGATTTCCCCGGTATGTCAAAA-GTCAAAGTATT
1720CAM .        GTCGATGCCGACAACAACGGATCTATCGATTTCCCCGGTATGTCAAAA-GTCAAAGTATG
1722CAM .        GTCGATGCCGACAACAACGGATCTATCGATTTCCCCGGTATGTCAAAA-GTCAAAGTATG
1724CAM .        GTCGATGCCGACAACAACGGATCTATCGATTTCCCCGGTATGTCAAAA-GTCAAAGTATG
334USA .         GTCGATGCCGACAACAACGGATCTATCGATTTCCCCGGTATGTCAAAA-GTCAAAGTATT
335USA .         GTCGATGCCGACAACAACGGATCTATCGATTTCCCCGGTATGTCAAAA-GTCAAAGTATT
1081WIS .        GTTGATGCCGACAACAACGGATCTATCGATTTCCCCGGTATGTCAAAA-GTCGAAGTATT
1069WIS .        GTCGATGCCGACAACAACGGATCTATCGATTTCCCCGGTATGTCAAAA-GTCAAAGTATT
1107CAN .        GTCGATGCCGACAACAACGGATCTATCGATTTCCCCGGTATGTCAAAA-GTCAAAGTATT
1108PEN .        GTCGATGCCGACAACAACGGATCTATCGATTTCCCCGGTATGTCAAAA-GTCAAAGTATT
1110CAN .        GTCGATGCCGACAACAACGGATCTATCGATTTCCCCGGTATGTCAAAA-GTCAAAGTGTT
JBSER241 .       GTCGATGCCGACAACAACGGATCTATCGATTTCCCTGGTATGTCAAAA-GTCGAAGTACT
246PHI .         GTCGATGCCGACAACAACGGATCTATCGATTTCCCCGGTATGTCAAAA-GTCAAAGTGTT
51SA .           GTCGATGCCGACAACAACGGATCTATCGATTTCCCCGGTATGTCAAAA-GTCAAAGTGTT
2618ETHI .       GTCGATGCCGACAACAACGGATCTATCGATTTCCCCGGTATGTCAAAA-GTCAAAGTGTT
1068WIS .        GTCGATGCCGACAACAACGGATCTATCGATTTCCCCGGTATGTCAAAA-GTCAAAGTATT
238CR .          GTCGATGCCGACAACAACGGATCTATCGATTTCCCCGGTATGTCAAAA-GTCAAAGTGTT
NR5555 .         GTCGATGCCGACAACAACGGATCTATCGATTTCCCCGGTATGTCAAAA-GTCAAAGTGTT
NR6839 .         GTCGATGCCGACAACAACGGATCTATCGATTTCCCCGGTATGTCAAAA-GTCAAAGTGTT
2624ETHI .       GTCGATGCCGACAACAACGGATCTATCGATTTCCCCGGTATGTCAAAA-GTCAAAGTGTT
845EGY .         GTCGATGCCGACAACAACGGATCTATCGATTTCCCCGGTATGTCAAAA-GTCAAAGTGTT
JBPER12 .        GTCGATGCCGACAACAACGGATCTATCGATTTCCCCGGTATGTCAAAA-GTCAAAGTGTT
1717VIET .       GTCGATGCCGACAACAACGGATCTATCGATTTCCCCGGTATGTCAAAG-GTCAAAGTGTT
709BRAZ .        GTCGATGCCGACAACAACGGATCTATCGATTTCCCCGGTATGTCAAAA-GTCACAAT-XX
878IRAN .        GTCGATGCCGACAACAACGGATCTATCGATTTCCCCGGTATGTXXXXXXXXXXXXXXXXX
239CR .          GTCGATGCCGACAACAACGGATCTATCGATTTCCCCGGTATGTCAAAA-GTCAAAGTGTT
202COM .         GTCGATGCCGACAACAACGGATCTATCGATTTCCCCGGTATGTCAAAG-GTCAAAGTGTT
1064MEX .        GTCGATGCCGACAACAACGGATCTATCGATTTCCCCGGTATGTCAAAA-GTCAAAGTGTT
596BRAZ .        GTCGATGCCGACAACAACGGATCTATCGATTTCCCCGGTATGTCAAAA-GTCGAAGTATT
333USA .         GTCGATGCCGACAACAACGGATCTATCGATTTCCCCGGTATGTCAAAA-GTCACAGTGTT
2610ETHI .       GTCGATGCCGACAACAACGGATCTATCGATTTCCCCGGTATGTCAAGA-GTCAAAGTGTT
1044RW .         GTCGATGCCGACAACAACGGATCTATCGATTTCCCCGGTATGTCAAAA-GTCGAGTTATT
JBNZ111 .        GTTGATGCCGACAACAACGGATCTATCGATTTCCCCGGTATGTCAAAA-GTCGAAGTATT
JBNZ12 .         GTCGATGCCGACAACAACGGATCCATCGATTTCCCCGGTATGTCAAAA-GTCAAAGTATT
JBNZ24 .         GTCGATGCCGACAACAACGGATCCATCGATTTCCCCGGTATGTCAAAA-GTCAAAGTATT
693CHINA .       GTCGATGCCGACAACAACGGATCTATTGATTTCCCCGGTATGTCAAAA-GTCGAGTTATT
1058RW .         GTCGATGCCGACAACAACGGATCTATCGATTTCCCCGGTATGTCAAAA-GTCGAGTTATT
590AUS .         GTCGATGCCGACAACAACGGATCTATCGATTTCCCCGGTATGTCAAAA-GTCGAAGTATT
53SA .           GTCGATGCCGACAACAACGGATCTATCGATTTCCCCGGTATGTCAAAA-GTCAAGTTATT
1075WIS .        GTCGATGCCGACAACAACGGATCTATCGATTTCCCCGGTATGTCAAGA-GTCAAAGTGTT
939GER .         GTCGATGCCGACAACAACGGATCTATCGATTTCCCCGGTATGTCAAGA-GTCAAAGTGTT
GA3804 .         GTCGATGCCGACAACAACGGATCTATCGATTTCCCCGGTATGTCAAGA-GTCAAAGTGTT
1599AT .         GTCGATGCCGACAACAACGGATCTATCGATTTCCCTGGTATGTCAAAA-GTCGAAGTACT
1941AT .         GTCGATGCCGACAACAACGGATCTATCGATTTCCCTGGTATGTCAAAA-GTCGAAGTACT
291NE .          XXXXXXXXXXXXXXXXXXXXXXXXXXXXXXXXXXXXXXXXXXXXXXXXXXXXXXXXXXXX
2301SAR .        GTCGATGCCGACAACAACGGATCTATCGATTTCCCTGGTATGTCAAAA-GTCAAAGTACT
2313SAR .        GTCGATGCCGACAACAACGGATCTATCGATTTCCCTGGTATGTCAAAA-GTCAAAGTACT
271NE .          GTCGATGCCGACAACAACGGATCTATCGATTTCCCCGGTATGTCAAGA-GTCAAAGTGTT
1935GER .        GTCGATGCCGACAACAACGGATCTATCGATTTCCCCGGTATGTCAAGA-GTCAAAGTGTT
JBRSA122 .       GTCGATGCCGACAACAACGGATCTATCGATTTCCCTGGTATGTCAAAA-GTCGAAGTACT
276NE .          GTCGATGCCGACAACAACGGATCTATCGATTTCCCCGGTATGTCAAGA-GTCAAAGTGTT
272NE .          GTCGATGCCGACAACAACGGATCTATCGATTTCCCCGGTATGTCAAGA-GTCAAAGTGTT
2673ETHI .       GTCGATGCCGACAACAACGGATCTATCGATTTCCCCGGTATGTCAAGA-GTCAAAGTGTT
1084AUSW .       GTCGATGCCGACAACAACGGATCTATCGATTTCCCCGGTATGTCAAGA-GTCAAAGTGTT
JBPER62 .        GTCGATGCCGACAACAACGGATCTATCGATTTCCCCGGTATGTCAAGA-GTCAAAGTGTT
JBNZ72 .         GTCGATGCCGACAACAACGGATCCATCGATTTCCCCGGTATGTCAAAA-GTCAAAGTATT
1065MEX .        GTCGATGCCGACAACAACGGATCTATCGATTTCCCCGGTATGTCAAAA-GTCGAGTTATT
646HAWAI .       GTCGATGCCGACAACAACGGATCTATCGATTTCCCCGGTATGTCAAAA-GTCGAAGTATT
727INDIA .       GTCGATGCCGACAACAACGGATCTATCGATTTCCCCGGTATGTCAAAA-GTCAAAGTGTT
743MALE .        GTCGATGCCGACAACAACGGATCTATCGATTTCCCCGGTATGTCAAAA-GTCAAAGTGTT
1059MEX .        GTCGATGCCGACAACAACGGATCTATCGATTTCCCCGGTATGTCAAAA-GTCAAAGTGTT
1061RW .         GTCGATGCCGACAACAACGGATCTATCGATTTCCCCGGTATGTCAAAA-GTCAAAGTGTT
1066MEX .        GTCGATGCCGACAACAACGGATCTATCGATTTCCCCGGTATGTCAAAA-GTCAAAGTGTT
1095BALI .       GTCGATGCCGACAACAACGGATCTATCGATTTCCCCGGTATGTCAAAA-GTCAAAGTGTT
1505MAUR .       GTCGATGCCGACAACAACGGATCTATCGATTTCCCCGGTATGTCAAAA-GTTAAAGTATT
1818ETHI .       GTCGATGCCGACAACAACGGATCTATCGATTTCCCCGGTATGTCAAAA-GTCAAAGTGTT
1934AT .         GTCGATGCCGACAACAACGGATCTATCGATTTCCCTGGTATGTCAAAA-GTCGAAGTACT
245CR .          GTCGATGCCGACAACAACGGATCTATCGATTTCCCCGGTATGTCAAAA-GTCAAAGTGTT
2710ETHI .       GTCGATGCCGACAACAACGGATCTATCGATTTCCCCGGTATGTCAAAA-GTCAAAGTGTT
274NE .          GTCGATGCCGACAACAACGGATCTATCGATTTCCCCGGTATGTCAAAA-GTCAAAGTGTT
3408PNG .        GTCGATGCCGACAACAACGGATCCATCGATTTCCCTGGTATGTCAAAAAGTCGAAGTATT
3409PNG .        GTCGATGCCGACAACAACGGATCCATCGATTTCCCTGGTATGTCAAAAAGTCGAAGTATT
410MALAY .       GTCGATGCCGACAACAACGGATCTATCGATTTCCCTGGTATGTCAAAA-GTCGAGATATT
836EGY .         GTCGATGCCGACAACAACGGATCCATCGATTTCCCCGGTATGTCAAAA-GTTAAAGTGTT
837EGY .         GTCGATGCCGACAACAACGGATCCATCGATTTCCCCGGTATGTCAAAA-GTTAAAGTGTT
JBRO111 .        GTCGATGCCGACAACAACGGATCTATCGATTTCCCCGGTATGTCAAAA-GTCAAAGTGTT
brunneoviridis4  GTCGATGCCGACAACAACGGATCTATCGATTTCCCCGGTATGTCAAAA-GTCCAAGTATT
brunneoviridis1  GTCGATGCCGACAACAACGGATCTATCGATTTCCCCGGTATGTCAAAA-GTCCAAGTATT
pleurotumCPK211  GTCGATGCCGACAACAACGGATCTTTCGATTTCCCCGGTATGTCAAAA-GTTGAAAGATX
PLEUROTICOLA .   GTCGATGCCGACAACAACGGATCTATCGATTTCCCCGGTATGTCAAAA-GTTGAAAGATT
epimycesCPK1981  GTCGATGCCGACAACAACGGATCCATCGATTTCCCCGGTATGTCAAAA-GCCCAAGTATT
alniCPK3124 ...  GTCGATGCCGACAACAACGGATCTATCGATTTCCCCGGTATGTCAAGA-GTCCAAGTATT
CPK5TAWA .       GTCGATGCCGACAACAACGGATCTATCGATTTCCCCGGTATGTCAAAA-GTCAAAGTATT
2974lobau .      GTCGATGCCGACAACAACGGATCTATCGATTTCCCTGGTATGTCAGAAAGTCGAAGTATT
2975lobau .      GTCGATGCCGACAACAACGGATCTATCGATTTCCCTGGTATGTCAGAAAGTCGAAGTATT
aggressivumCPK3  GTCGATGCCGACAACAACGGATCCATCGATTTCCCCGGTATGTCAAGA-GTCCAAGTATT
atrogelatinosaC  GTCGATGCCGACAACAACGGATCTTTCGATTTCCCCGGTATGTCAAAA-GTCCACGTATT
```

```
atrogelatinosaC  GTCGATGCCGACAACAACGGATCTATCGATTTCCCCGGTATGTCAAAA-GTCGAAGTATT
cerinumCPK293 .  GTTGATGCCGACAACAACGGATCCATCGATTTCCCCGGTATGTCAAAA-GTCGAAGTATT
cerinumCPK854 .  GTTGATGCCGACAACAACGGATCCATCGATTTCCCCGGTATGTCAAAA-GTCGAAGTATT
tomentosumCPK88  GTTGATGCCGACAACAACGGATCCATCGATTTCCCCGGTATGTCAAAA-GTCGAAGTATA
stramineaGJS028  GTTGATGCCGACAACAACGGATCTATCGATTTCCCCGGTATGTCAAAA-GTTGAAGTATT
velutinumCPK312  GTTGATGCCGACAACAACGGATCTATCGATTTCCCCGGTATGTCAAAA-GTTGAAGTATT
catoptronGJS027  GTCGATGCCGACAACAACGGATCTATCGATTTCCCCGGTATGTCAAAA-GTCGAAATATT
                 ################################################ ###########


                        730       740       750       760       770       780
                 =========+=========+=========+=========+=========+=========+
1093CAN .        GGGTCGTTGCCACATTCGGGCTAATACGGAGCGGTGTAGAATTCAACTGCCATTACCTTC
1052SA .         GGGTCGTTGCCACATTCGGGCTAATACGGAGCGGTGTAGAATTCAACTGCCATTACCTTC
1116CAN .        GGGTCGTTGCCACATTCGGGCTAATACGGAGCGGTGTAGAATTCAACTGCCATTACCTTC
1070EIRE .       GGGTCGTTGCCACATTCGGGCTAATACGGAGCGGTGTAGAATTCAACTGCCATTACCTTC
1099CAN .        GGGTCGTTGCCACATTCGGGCTAATACGGAGCGGTGTAGAATTCAACTGCCATTACCTTC
1104CAN .        GGGTCGTTGCCACATTCGGGCTAATACGGAGCGGTGTAGAATTCAACTGCCATTACCTTC
1087CAN .        GGGTCGTTGCCACATTCGGGCTAATACGGAGCGGTGTAGAATTCAACTGCCATTACCTTC
360UK .          GGGTCGTTGCCACATTCGGGCTAATACGGAGCGGTGTAGAATTCAACTGCCATTACCTTC
2111HUN .        GGGTCGTTGCCACATTCGGGCTAATACGGAGCGGTGTAGAATTCAACTGCCATTACCTTC
217RUS .         GGGTCGTTGCCACATTCGGGCTAATACGGAGCGGTGTAGAATTCAACTGCCATTACCTTC
204UK .          GGGTCGTTGCCACATTCGGGCTAATACXXXXXXXXXXXXXXXXXAACTGCCATTACCTTC
206RUS .         GGGTCGTTGCCACATTCGGGCTAATACGGAGCGGTGTAGAATTC--CTGCCATTACCTTC
261SIB .         GGGTCGTTGCCACATTCGGGCTAATACGGAGCGGTGTAGAATTCAACTGCCATTACCTTC
265SIB .         GGGTCGTTGCCACATTCGGGCTAATACGGAGCGGTGTAGAATTCAACTGCCATTACCTTC
2784THAI .       GGGTCGTTGCCACATTCGAGCTAATACGGAGCGGTGTAGAATTCAACTGCCATTACCTTT
838EGY .         -GGTCGTTGCCACATTCGAGCTAATACGGAGCGGTGTAGAATTCAACTGCCATTACCTTT
1102CAN .        GGGTCGTTGCCACATTCGAGCTAATACGGAGCGGTGTAGAATTCAACTGCCATTACCTTT
588LAOS .        GGGTCGTTAGCACATTCGAGCTAATACGGAGCGGTGTAGAATTCAACTGCCATTACCTTT
1596AT .         GGGCCGTTGCCACATTCGAGCTAATACGGAGCGGTGTAGAATTCAACTGCCATTACCTTT
1720CAM .        GGGTCGTTACCACATTCGAGCTAATACGGAGCGGTGTAGAATTCAACTGCCATTACCTTT
1722CAM .        GGGTCGTTACCACATTCGAGCTAATACGGAGCGGTGTAGAATTCAACTGCCATTACCTTT
1724CAM .        GGGTCGTTACCACATTCGAGCTAATACGGAGCGGTGTAGAATTCAACTGCCATTACCTTT
334USA .         GGGTCGTTGCCACATTCGAGCTAATACGGAGCGGTGTAGAATTCAACTGCCATTACCTTT
335USA .         GGGTCGTTGCCACATTCGAGCTAATACGGAGCGGTGTAGAATTCAACTGCCATTACCTTT
1081WIS .        GGGTCGTTGCCACATTCGAGCTAATACGGAGCGGTGTAGAATTCAACTGCCATTACCTTT
1069WIS .        GGGTCGTTGCCACATTCGAGCTAATACGGAGCGGTGTAGAATTCAACTGCCATTACCTTT
1107CAN .        GGGTCGTTGCCACATTCGAGCTAATACGGAGCGGTGTAGAATTCAACTGCCATTACCTTT
1108PEN .        GGGCCGTTGCCACATTCGAGCTAATACGGAGCGGTGTAGAATTCAACTGCCATTACCTTT
1110CAN .        GGGTCGTTGCCACATTCGGGCTAATACGGAGCGGTGTAGAATTCAACTGCCATTACCTTT
JBSER241 .       GGGTCGTTATCCCATTCGAGCTAATACGGAGCGGTGTAGAATTCGACTGCCATTACCTTC
246PHI .         GGGTCGTTCCCATXXXXXXXXXXXXXXXXXXXXXXXXXXXXXXXGACTGCCATTACCTTC
51SA .           GGGTCGTTCCCATATTCAAGCTAATACGGAGCGGTGTAGAATTCGACTGCCATTACCTTC
2618ETHI .       GGGTCGTTCCCATATTCGAGCTAATACGGAGCGGTGTAGAATTCGACTGCCATTACCTTC
1068WIS .        GGGTCGTTGCCACATTCGAGCTAATACGGAGCGGTGTAGAATTCAACTGCCATTACCTTT
238CR .          GGGTCGTTCCCATATTCGAGCTAATACGGAGCGGTGTAGAATTCGACTGCCATTACCTTC
NR5555 .         GGGTCGTTCCCATATTCGAGCTAATACGGAGCGGTGTAGAATTC--CTGCCATTACCTTC
NR6839 .         GGGTCGTTGCCATATTCGAGCTAATACGGAGCGGTGTAGAATTC--CTGCCATTACCTTC
2624ETHI .       GGGTCGTTGCCATATTCGAGCTAATACGGAGCGGTGTAGAATTCGACTGCCATTACCTTC
845EGY .         GGGTCGTTGCCATATTCGAGCTAATACGGAGCGGTGTAGAATTCGACTGCCATTACCTTC
JBPER12 .        GGGTCGTTGCCACATTCGAGCTAATACGGAGCGGTGTAGAATTCXXXXXXXXXXXXXXXX
1717VIET .       GGGTCGTTGCCACATTCGAGCTAATACGGAGCGGTGTAGAATTCAACTGCCATTACTTTC
709BRAZ .        XXXXXXXXXXXXXXXXXXXXXXXXXXXXXXXXXXXXXXXXXXXXAACTGCCATTACTTTC
878IRAN .        XXXXXXXXXXXXXXXXXXXXXXXXXXXXXXXXXXXXXXXXXXXXAACTGCCATTACTTTC
239CR .          GGGTCGTTGCCACATTCGAGCTAATACGGAGCGGTGTAGA----AACTGCCATTACTTTC
202COM .         GGGTCGTTGCCACATTCGAGCTAATACGGAGCGGTGTAGAATTC---TGCCATTACTTTC
1064MEX .        GGGTCGTTGCCACATTCGAGCTAATACGGAGCGGTGTAGAATTC----------------
596BRAZ .        GGGTCGTTACCACATTCGAGCTAATACGGAGCGGTGTAGAATTCAACTGCCATTACTTTC
333USA .         GGGTCGTTGCCACATTCGAGCTAATACGGAGCGGTGTAGAATTCAACTGCCATTACTTTC
2610ETHI .       GGGTCGTTTCCACATTCGAGCTAATACGGAGCGGTGTAGAATTCAACTGCCATTACCTTC
1044RW .         GGGTCGTTGCCACATTCGAGCTAATACGGAGCGGTGTAGAATTCAACTGCCATCACCTTC
JBNZ111 .        AGGTCGTTGGCACATTCGAGCTAATACGGAGCG-----------AACTGCCATTACTTTC
JBNZ12 .         GGGTCGTTGCCACATTCGAGCTAATACGGAGCGGTGTAGAATTCAACTGCCATTACCTTC
JBNZ24 .         GGGTCGTTGCCACATTCGAGCTAATACGGAGCGGTGTAGAATTCAACTGCCATTACTTTC
693CHINA .       GGGTCGTTGCCACATTCGAGCTAATACGGAGCGGTGTAGAATTCAACCGCCATTACCTTC
1058RW .         GGGTCGTTGCCACATTCGAGCTAATACGGAGCGGTGTAGAATTCAACTGCCATCACCTTC
590AUS .         GGGTCGTTACCACATTCGAGCTAATACGGAGCGGTGTAGAATTCAACTGCCATTACCTTC
53SA .           GGGTCGTTGCCACATTCGAGCTAATACGGAGCGGTGTATAATTCAACTGCCATTACCTTC
1075WIS .        GGGTCGTTTCCACATTCGAGCTAATACGGAGCGGTGTAGAATTCAACTGCCATTACCTTC
939GER .         GGGTCGTTTCCACATTCGAGCTAATACGGAGCGGTGTAGAATTCAACTGCCATTACCTTC
GA3804 .         GGGTCGTTTCCACATTCGAGCTAATACGGAGCGGTGTAGAATTCAACTGCCATTACCTTC
1599AT .         GGGTCGTTATCCCATTCGAGCTAATACGGAGCGGTGTAGAATTCGACTGCCATTACCTTC
1941AT .         GGGTCGTTATCCCATTCGAGCTAATACGGAGCGGTGTAGAATTCAACTGCCATTACCTTC
291NE .          XXXXXXXXXXXXXXXXXXXXXXXXXXXXXXXXXXXXXXXXXXXXAACTGCCATTACCTTC
2301SAR .        GGGTCGTTATCCCATTCGAGCTAATACGGAGCGGTGTAGAATTCGACTGCCATTACCTTC
2313SAR .        GGGTCGTTATCCCATTCGAGCTAATACGGAGCGGTGTAGAATTCAACTGCCATTACCTTC
271NE .          GGGTCGTTTCCACATTCGAGCTAATCCGGAGCGGTGTAGAATTCXXCTGCCATTACCTTC
1935GER .        GGGTCGTTTCCACATTCGAGCTAATCCGGAGCGGTGTAGAATTCGACTGCCATTACCTTC
JBRSA122 .       GGGTCGTTATCCCATTCGAGCTAATACGGAGCGGTGTAGAATTCGACTGCCATTACCTTC
276NE .          GGGTCGTTTCCACATTCGAGCTAATACGGAGCGGTGTAGAATTCAACTGCCATTACCTTC
272NE .          GGGTCGTTTCCACATTCGAGCTAATACGGAGCGGTGTAGAATTCAACTGCCATTACCTTC
2673ETHI .       GGGTCGTTTCCACATTCGAGCTAATACGGAGCGGTGTAGAATTCAACTGCCATTACCTTC
1084AUSW .       GGGTCGTTTCCACATTCGAGCTAATACGGAGCGGTGTAGAATTCAACTGCCATTACCTTC
JBPER62 .        GGGTCGTTTCCACATTCGAGCTAATACGGAGCGGTGTAGAATTCAACTGCCATTACCTTC
JBNZ72 .         GGGTCGTTGCCACATTCGAGCTAATACGGAGCGGTGTAGAATTCAACTGCCATTACCTTC
1065MEX .        GGGTCGTTGCCACATTCGAGCTAATACGGAGCGGTGTAGAATTCXXXXXXXXXXXXXXXX
646HAWAI .       GGGTCGTTACCACATTCGAGCTAATACGGAGCGGTGTAGAATTCAACTGCCATTACCTTC
727INDIA .       GGGTCGTTGCCACATTCGAGCTAATACGGAGCGGTGTAGAATTCAACTGCCATTACCTTC
743MALE .        GGGTCGTTGCCACATTCGAGCTAATACGGAGCGGTGTAGAATTCAACTGCCATTACCTTC
1059MEX .        GGGTCGTTGCCACATTCGAGCTAATACGGAGCGGTGTAGAATTCXXXXXXXXXXXXXXXX
1061RW .         GGGTCGTTCCCATATTCGAGCTAATACGGAGCGGTGTAGAATTCGACTGCCATTACCTTC
1066MEX .        GGGTCGTTGCCACATTCGAGCTAATACGGAGCGGTGTAGAATTCAACTGCCATTACCTTC
1095BALI .       GGGTCGTTGCCACATTCGAGCTAATACGGAGCGGTGTAGAATTCGACTGCCATTACCTTC
1505MAUR .       GGGTCGTTGCCACATTCGAGCTAATACGGAGCGGTGTAGAATTCGACTGCCATTACCTTC
1818ETHI .       GGGTCGTTGCCACATTCGGGCTAATACGGAGCGGTGTAGAATTCAACTGCCATTACCTTC
1934AT .         GGGTCGTTATCCCATTCGAGCTAATACGGAGCGGTGTAGAATTCAACTGCCATTACCTTC
245CR .          GGGTCGTTGCCACATTCGAGCTAATACGGAGCGGTGTAGAATTCAACTGCCATTACCTTC
2710ETHI .       GGGTCGTTGCCACATTCGAGCTAATACGGAGCGGTGTAGAATTCAACTGCCATTACCTTC
274NE .          GGGTCGTTGCCACATTCGAGCTAATACGGAGCGGTGTAGAATTCAACTGCCATTACCTTC
3408PNG .        GGGTCGCTACCACATTCGAGCTAATACGGAGCGGTGTAGAATTXAACTGCCATTACCTTC
3409PNG .        GGGTCGCTACCACATTCGAGCTAATACGGAGCGGTGTAGAATTXAACTGCCATTACCTTC
410MALAY .       GGGTAGTTACCACATTCGGGCTAATACGAAGCGGTGTAGAATTCAACTGCCATTACATTC
836EGY .         GGGTCGTTGCCACATTCGAGCTAATACGGAGCGGTGTAGAATTCAACTGCCATTACCTTC
837EGY .         GGGTCGTTGCCACATTCGAGCTAATACGGAGCGGTGTAGAATTCGACTGCCATTACCTTC
JBRO111 .        GGGTCGTTGCCACATTCGGGCTAATACGGAGCGGTGTAGAATTCAACTGCCATTACCTTC
brunneoviridis4  GGATCGTTGCCACATTCGGGCTAATACGGAGCGGTGTAGAATTCXACTGCCATTGCGTTC
brunneoviridis1  GGATCGTTGCCACATTCGGGCTAATACGGAGCGGTGTAGAATTCXACTGCCATTGCGTTC
pleurotumCPK211  XXXXXXXXXXXXXXXXXXXXXXXXXXXXXXXXXXXXXXXXXXXXAACTGCCATTGCATTC
PLEUROTICOLA .   GGATCGTTGCCACATTCGAGCTAATACGGAGCGGTGTAGAGTTCAACTGCCATTGCATTC
epimycesCPK1981  GGATCGTTATCACATTCGGGCTAATACGGAGCGGTGTAGAATTCAACTGCCATTACCTTC
alniCPK3124 ...  GGATCGTTACCACATTCGGGCTAATGCGGAGCGGTGTAAAATTCAACTGCCATTGCATTC
CPK5TAWA .       GGATCGTTATCACATCXXXXXXXXXXXXXXXXXXXXXXXXXXXXXACTGCCATTGCATTC
2974lobau .      GGGTCGCTACCACATTCGAGCTAATACGGAGCGGTGTAGAATTCAACTGCCATTACCTTC
2975lobau .      GGGTCGCTACCACATTCGAGCTAATACGGAGCGGTGTAGAATTCAACTGCCATTACCTTC
aggressivumCPK3  GGATCGTTATCACATTCGGGCTAATACGGAGCGGTGTAGAATTCAACTGCCATTACCTTC
atrogelatinosaC  GGATCGTTGGCACATTTGGGCTAATACGGAGCGGTGTAAAATTCAACTGCCATTGCATTC
```

```
atrogelatinosaC  GGATCATTGCCACATTCGAGCTAATACGGAGCGGTGTAGAATTCAACTGCCATTGCATTC
cerinumCPK293 .  GGGTCGTTACCACATTCGGGCTAATACGGAGCGGTXXXXXXXXXAACTGCCATTGCATTC
cerinumCPK854 .  GGGTCGTTACCACATTCGGGCTAATACGGAGCGGTGTAGAATTCAACTGCCATTGCATTC
tomentosumCPK88  TGGTCGTTACCACATTCGAGCTAATACGGAGCGGTGTAGAATTCAACTGCCATTGCATTC
stramineaGJS028  GGGTCGTTACCGCATTCGGGCTAATACGGAGCGGTGTAGAATTCAACTGCCATTGCATTC
velutinumCPK312  GGGTCGTTACCACATTCGAGCTAATACGGAGCGGTGTAGAATTCAACTGCCATTACATTC
catoptronGJS027  GGGTCGTTGCCACATTCGGGCTAATACGGAGCGGTATAGAATTCAACTGCCATTACATTC
                 ############################################################


                        790       800       810       820       830       840
                 =========+=========+=========+=========+=========+=========+
1093CAN .        ATGAAGGATTGGGGTTTCGATGGCATTGACGTCGACTGGGAGTACCCTGCAGACGCCACC
1052SA .         ATGAAGGATTGGGGTTTCGATGGCATTGACGTCGACTGGGAGTACCCTGCAGACGCCACC
1116CAN .        ATGAAGGATTGGGGTTTCGATGGCATTGACGTCGACTGGGAGTACCCTGCAGACGCCACC
1070EIRE .       ATGAAGGATTGGGGTTTCGATGGCATTGACGTCGACTGGGAGTACCCTGCAGACGCCACC
1099CAN .        ATGAAGGATTGGGGTTTCGATGGCATTGACGTCGACTGGGAGTACCCTGCAGACGCCACC
1104CAN .        ATGAAGGATTGGGGTTTCGATGGCATTGACGTCGACTGGGAGTACCCTGCAGACGCCACC
1087CAN .        ATGAAGGATTGGGGTTTCGATGGCATTGACGTCGACTGGGAGTACCCTGCAGACGCCACC
360UK .          ATGAAGGATTGGGGTTTCGATGGCATTGACGTCGACTGGGAGTACCCTGCAGACGCCACC
2111HUN .        ATGAAGGATTGGGGTTTCGATGGCATTGACGTCGACTGGGAGTACCCTGCAGACGCCACC
217RUS .         ATGAAGGATTGGGGTTTCGATGGCATTGACGTCGACTGGGAGTACCCTGCAGACGCCACC
204UK .          ATGAAGGATTGGGGTTTCGATGGCATTGACGTCGACTGGGAGTACCCTGCAGACGCCACC
206RUS .         ATGAAGGATTGGGGTTTCGATGGCATTGACGTCGACTGGGAGTACCCTGCAGACGCCACC
261SIB .         ATGAAGGATTGGGGTTTCGATGGCATTGACGTCGACTGGGAGTACCCTGCAGACGCCACC
265SIB .         ATGAAGGATTGGGGTTTCGATGGCATTGACGTCGACTGGGAGTACCCTGCAGACGCCACC
2784THAI .       ATGAAGGATTGGGGTTTCGATGGTATTGATATCGACTGGGAGTACCCTGCAGACGCCACC
838EGY .         ATGAAGGATTGGGGTTTCGATGGTATTGATATTGACTGGGAGTACCCTGCAGACGCCACC
1102CAN .        ATGAAGGATTGGGGTTTCGATGGTATTGATATCGACTGGGAGTACCCTGCAGACGCCACC
588LAOS .        ATGAAGGATTGGGGTTTCGATGGTATTGATATCGACTGGGAGTACCCTGCAGACGCCACC
1596AT .         ATGAAGGATTGGGGTTTCGATGGTATTGATATCGACTGGGAGTACCCTGCAGACGCCACC
1720CAM .        ATGAAGGATTGGGGTTTCGATGGTATTGATATCGACTGGGAGTACCCTGCAGACGCCACC
1722CAM .        ATGAAGGATTGGGGTTTCGATGGTATTGATATCGACTGGGAGTACCCTGCAGACGCCACC
1724CAM .        ATGAAGGATTGGGGTTTCGATGGTATTGATATCGACTGGGAGTACCCTGCAGACGCCACC
334USA .         ATGAAGGATTGGGGTTTCGATGGTATTGATATCGACTGGGAGTACCCTGCAGACGCCACC
335USA .         ATGAAGGATTGGGGTTTCGATGGTATTGATATCGACTGGGAGTACCCTGCAGACGCCACC
1081WIS .        ATGAAGGATTGGGGTTTCGATGGTATTGATATCGACTGGGAGTACCCTGCAGACGCCACC
1069WIS .        ATGAAGGATTGGGGTTTCGATGGCATTGATATCGACTGGGAGTACCCTGCAGACGCCACC
1107CAN .        ATGAAGGATTGGGGTTTCGATGGTATTGATATCGACTGGGAGTACCCTGCAGACGCCACC
1108PEN .        ATGAAGGATTGGGGTTTCGATGGTATTGATATCGACTGGGAGTACCCTGCAGACGCCACC
1110CAN .        ATGAAGGATTGGGGTTTCGATGGTATTGATATCGACTGGGAGTACCCTGCAGACGCCACC
JBSER241 .       ATGAAGGATTGGGGTTTCGATGGCATTGACGTCGATTGGGAGTACCCTGCAGACGCCACC
246PHI .         ATGAAGGATTGGGGTTTCGATGGCATTGACGTCGATTGGGAGTACCCTGCAGACGCCACC
51SA .           ATGAAGGATTGGGGTTTCGATGGCATTGACGTCGATTGGGAGTACCCTGCAGACGCCACC
2618ETHI .       ATGAAGGATTGGGGTTTCGATGGCATTGACGTCGATTGGGAGTACCCTGCAGACGCCACC
1068WIS .        ATGAAGGATTGGGGTTTCGATGGCATTGATATCGACTGGGAGTACCCTGCAGACGCCACC
238CR .          ATGAAGGATTGGGGTTTCGATGGCATTGACGTCGATTGGGAGTACCCTGCAGACGCCACC
NR5555 .         ATGAAGGATTGGGGTTTCGATGGCATTGACGTCGATTGGGAGTACCCTGCAGACGCCACC
NR6839 .         ATGAAGGATTGGGGTTTCGATGGCATTGACGTCGATTGGGAGTACCCTGCAGACGCCACC
2624ETHI .       ATGAAGGATTGGGGTTTCGATGGCATTGACGTCGATTGGGAGTACCCTGCAGACGCCACC
845EGY .         ATGAAGGATTGGGGTTTCGATGGCATTGACGTCGATTGGGAGTACCCTGCAGACGCCACC
JBPER12 .        XXXXXXXXXXXXXXXXXXXXXXXXXXXXXXXXXXXXXXXXXXXXXXXXXXXXXXXXXXXX
1717VIET .       ATGAAGGATTGGGGTTTCGATGGCATTGACGTTGATTGGGAGTACCCTGCAGACGCCACC
709BRAZ .        ATGAAGGATTGGGGTTTCGATGGCATTGACGTCGATTGGGAGTACCCTGCAGACGCCACC
878IRAN .        ATGAAGGATTGGGGTTTCGATGGCATTGACGTCGATTGGGAGTACCCTGCAAACGCCACC
239CR .          ATGAAGGATTGGGGTTTCGATGGCATTGACGTCGATTGGGAGTACCCTGCAGACGCCACC
202COM .         ATGAAGGATTGGGGTTTCGATGGCATTGACGTCGATTGGGAGTACCCTGCAGACGCCACC
1064MEX .        -----------------------CATTGACGTCGATTGGGAGTACCCTGCAGACGCCACC
596BRAZ .        ATGAAGGATTGGGGTTTCGATGGCATTGACGTCGATTGGGAGTACCCTGCAGACGCCACC
333USA .         ATGAAGGATTGGGGTTTCGATGGCATTGACGTCGATTGGGAGTACCCTGCAGACGCCACC
2610ETHI .       ATGAAGGATTGGGGTTTCGATGGCATTGACGTCGACTGGGAGTACCCTGCAGACGCCACC
1044RW .         ATGAAGGATTGGGGTTTCGATGGCATTGACGTCGACTGGGAGTACCCTGCAGACGCCACC
JBNZ111 .        ATGAAGGATTGGGGTTTCGATGGCATTGACGTCGACTGGGAGTTCCCTGCAGACGCCACC
JBNZ12 .         ATGAAGGATTGGGGTTTCGATGGCATTGACGTCGACTGGGAGTACCCTGCAGATGCCACC
JBNZ24 .         ATGAAGGATTGGGGTTTCGATGGCATTGACGTCGACTGGGAGTTCCCTGCAGACGCCACC
693CHINA .       ATGAAGGATTGGGGTTTCGATGGCATTGACGTCGACTGGGAGTACCCTGCAGATGCCACC
1058RW .         ATGAAGGATTGGGGTTTCGATGGCATTGACGTCGACTGGGAGTACCCTGCAGACGCCACC
590AUS .         ATGAAGGATTGGGGTTTCGATGGCATTGACGTCGACTGGGAGTACCCTGCAGACGCCACC
53SA .           ATGAAGGATTGGGGTTTCGATGGCATTGACGTCGACTGGGAGTACCCTGCAGACGCCACC
1075WIS .        ATGAAGGATTGGGGTTTCGATGGCATTGACGTCGACTGGGAGTACCCTGCAGATGCCACC
939GER .         ATGAAGGATTGGGGTTTCGATGGCATTGACGTCGACTGGGAGTACCCTGCAGATGCCACC
GA3804 .         ATGAAGGATTGGGGTTTCGATGGCATTGACGTCGACTGGGAGTACCCTGCAGATGCCACC
1599AT .         ATGAAGGATTGGGGTTTCGATGGCATTGACGTCGACTGGGAGTACCCTGCAGACGCCACC
1941AT .         ATGAAGGATTGGGGTTTCGATGGCATTGACGTCGACTGGGAGTACCCTGCAGATGCCACC
291NE .          ATGAAGGATTGGGGTTTCGATGGTATTGACGTCGACTGGGAGTACCCTGCAGACGCCACC
2301SAR .        ATGAAGGATTGGGGTTTCGATGGCATTGACGTCGACTGGGAGTACCCTGCAGACGCCACC
2313SAR .        ATGAAGGATTGGGGTTTCGATGGCATTGACGTCGACTGGGAGTACCCTGCAGATGCCACC
271NE .          ATGAAGGATTGGGGTTTCGATGGCATTGACGTCGACTGGGAGTACCCTGCAGATGCCACC
1935GER .        ATGAAGGATTGGGGTTTCGATGGCATTGACGTCGACTGGGAGTACCCTGCAGACGCCACC
JBRSA122 .       ATGAAGGATTGGGGTTTCGATGGCATTGACGTCGACTGGGAGTACCCTGCAGACGCCACC
276NE .          ATGAAGGATTGGGGTTTCGATGGCATTGACGTCGACTGGGAGTACCCTGCAGATGCCACC
272NE .          ATGAAGGATTGGGGTTTCGATGGCATTGACGTCGACTGGGAGTACCCTGCAGATGCCACC
2673ETHI .       ATGAAGGATTGGGGTTTCGATGGCATTGACGTCGACTGGGAGTACCCTGCAGATGCCACC
1084AUSW .       ATGAAGGATTGGGGTTTCGATGGCATTGACGTCGACTGGGAGTACCCTGCAGACGCCACC
JBPER62 .        ATGAAGGATTGGGGTTTCGATGGCATTGACGTCGACTGGGAGTACCCTGCAGATGCCACC
JBNZ72 .         ATGAAGGATTGGGGTTTCGATGGCATTGACGTCGACTGGGAGTACCCTGCAGATGCCACC
1065MEX .        XXXXXXXXXXXXXXXXXXXXXXXXXXXXXXXXXXXXXXXXXXXXXXXXXXXXXXXXXXXX
646HAWAI .       ATGAAGGATTGGGGTTTCGATGGCATTGACGTCGACTGGGAGTACCCTGCCGACGCCACC
727INDIA .       ATGAAGGATTGGGGTTTCGATGGCATTGACGTCGACTGGGAGTACCCTGCCGACGCCACC
743MALE .        ATGAAGGATTGGGGTTTCGATGGCATTGACGTCGACTGGGAGTACCCTGCCGACGCCACC
1059MEX .        XXXXXXXXXXXXXXXXXXXXXXXXXXXXXXXXXXXXXXXXXXXXXXXXXXXXXXXXXXXX
1061RW .         ATGAAGGATTGGGGTTTCGATGGCATTGACGTCGATTGGGAGTACCCTGCAGACGCCACC
1066MEX .        ATGAAGGATTGGGGTTTCGATGGCATTGACGTCGACTGGGAGTACCCTGCAGACGCCACC
1095BALI .       ATGAAGGATTGGGGTTTCGATGGCATTGATGTCGATTGGGAGTACCCTGCAGACGCCACC
1505MAUR .       ATGAAGGATTGGGGTTTCGATGGCATTGACGTCGACTGGGAGTACCCTGCAGACGCCACC
1818ETHI .       ATGAAGGATTGGGGTTTCGATGGCATTGACGTCGACTGGGAGTACCCTGCAGACGCCACC
1934AT .         ATGAAGGATTGGGGTTTCGATGGCATTGACGTCGACTGGGAGTACCCTGCAGATGCCACC
245CR .          ATGAAGGATTGGGGTTTCGATGGCATTGACGTCGACTGGGAGTACCCTGCCGACGCCACC
2710ETHI .       ATGAAGGATTGGGGTTTCGATGGCATTGACGTCGACTGGGAGTACCCTGCCGACGCCACC
274NE .          ATGAAGGATTGGGGTTTCGATGGCATTGACGTCGACTGGGAGTACCCTGCCGACGCCACC
3408PNG .        ATGAAGGATTGGGGTTTCGATGGCATTGACGTCGACTGGGAGTACCCTGCAGACGCCACT
3409PNG .        ATGAAGGATTGGGGTTTCGATGGCATTGACGTCGACTGGGAGTACCCTGCAGACGCCACT
410MALAY .       ATGAAGGATTGGGGTTTCGATGGCATTGACGTCGACTGGGAGTACCCTGCAGATGCTACC
836EGY .         ATGAAGGATTGGGGTTTCGATGGCATTGACGTCGACTGGGAGTACCCTGCAGACGCCACC
837EGY .         ATGAAGGATTGGGGTTTCGATGGCATTGACGTCGACTGGGAGTACCCTGCAGACGCCACC
JBRO111 .        ATGAAGGATTGGGGTTTCGATGGCATTGACGTCGACTGGGAGTACCCTGCAGACGCCACC
brunneoviridis4  ATGAAGGATTGGGGTTTCGATGGCATTGACGTCGACTGGGAGTACCCTGCCGACAGCACC
brunneoviridis1  ATGAAGGATTGGGGTTTCGATGGCATTGACGTCGACTGGGAGTACCCTGCCGACAGCACC
pleurotumCPK211  ATGAAGGATTGGGGTTTCGATGGCATTGACATCGACTGGGAGTACCCTGCCGACAGCACC
PLEUROTICOLA .   ATGAAGGATKGGGGTTTCRATGGCATTGACGTCgACTGGGAGTACCCTGCCGACAGCACC
epimycesCPK1981  ATGAAGGATTGGGGTTTCGATGGTATTGACGTCGACTGGGAGTACCCTGCCGATGCCACC
alniCPK3124 ...  ATGAAGGATTGGGGTTTCGATGGCATTGACGTCGACTGGGAGTACCCTGCCGACAGCACC
CPK5TAWA .       ATGAAGGATKGGGGTTTCGATGGCATTGACATCGACTGGGAGTACCCTGCCGACAGCACC
2974lobau .      ATGAAGGATTGGGGTTTCGATGGCATTGACGTCGACTGGGAGTACCCTGCAGATGCCACC
2975lobau .      ATGAAGGATTGGGGTTTCGATGGCATTGACGTCGACTGGGAGTACCCTGCAGATGCCACC
aggressivumCPK3  ATGAAGGATTGGGGTTTCGATGGTATTGACGTCGACTGGGAGTACCCTGCCGATGCCACC
atrogelatinosaC  ATGAAGGATTGGGGTTTCGATGGCATTGACGTCGACTGGGAGTACCCTGCCGACAGCACC
```

```
atrogelatinosaC  ATGAAGGATTGGGGTTTCGATGGCATTGACGTCGACTGGGAGTACCCTGCCGACAGCACC
cerinumCPK293 .  ATGAAGGATTGGGGTTTCGATGGTATTGATGTCGATTGGGAGTACCCTGCAGACAGCACC
cerinumCPK854 .  ATGAAGGATTGGGGTTTCGATGGTATTGATGTCGACTGGGAGTACCCTGCAGACAGCACC
tomentosumCPK88  ATGAAGGATTGGGGTTTCGATGGTATTGACGTCGACTGGGAGTACCCTGCAGACAGCACC
stramineaGJS028  ATGAAGGATTGGGGTTTCGATGGTATTGACGTCGACTGGGAGTACCCTGCAGACAGCACC
velutinumCPK312  ATGAAGGATTGGGGTTTCGATGGTATTGATGTCGACTGGGAGTACCCTGCAGACAGCACC
catoptronGJS027  ATGAAGGATTGGGGTTTCGATGGTATTGACGTCGACTGGGAGTACCCTGCAGACAGCACC
                 ############################################################


                        850       860       870       880       890       900
                 =========+=========+=========+=========+=========+=========+
1093CAN .        CAGGCCTCCAACATGGTTCTTCTGCTGAAGGAAGTCCGATCTCAGCTGGATGCTTATGCT
1052SA .         CAGGCCTCCAACATGGTTCTTCTGCTGAAGGAAGTCCGATCTCAGCTGGATGCTTATGCT
1116CAN .        CAGGCCTCCAACATGGTTCTTCTGCTGAAGGAAGTCCGATCTCAGCTGGATGCTTATGCT
1070EIRE .       CAGGCCTCCAACATGGTTCTTCTGCTGAAGGAAGTCCGATCTCAGCTGGATGCTTATGCT
1099CAN .        CAGGCCTCCAACATGGTTCTTCTGCTGAAGGAAGTCCGATCTCAGCTGGATGCTTATGCT
1104CAN .        CAGGCCTCCAACATGGTTCTTCTGCTGAAGGAAGTCCGATCTCAGCTGGATGCTTATGCT
1087CAN .        CAGGCCTCCAACATGGTTCTTCTGCTGAAGGAAGTCCGATCTCAGCTGGATGCTTATGCT
360UK .          CAGGCCTCCAACATGGTTCTTCTGCTGAAGGAAGTCCGATCTCAGCTGGATGCTTATGCT
2111HUN .        CAGGCCTCCAACATGGTTCTTCTGCTGAAGGAAGTCCGATCTCAGCTGGATGCTTATGCT
217RUS .         CAGGCCTCCAACATGGTTCTTCTG------------------------------------
204UK .          CAGGCCTCCAACATGGTTCTTCTGCTGAAGGAAGTCCGATCTCAGCTGGATGCTTATGCT
206RUS .         CAGGCCTCCAACATGGTTCTTCTGCTGAAGGAAGTCCGATCTCAGCTGGATGCTTATGCT
261SIB .         CAGGCCTCCAACATGGTTCTTCTGCTGAAGGAAGTCCGATCTCAGCTGGATGCTTATGCT
265SIB .         CAGGCCTCCAACATGGTTCTTCTGCTGAAGGAAGTCCGATCTCAGCTGGATGCTTATGCT
2784THAI .       CAGGCCTCCAACATGATTCTTCTGCTGAAGGAAGTCCGATCTCAGCTGGATGCTTATGCT
838EGY .         CAGGCCTCCAACATGATTCTTCTGCTGAAGGAAGTCCGATCTCAGCTGGATGCTTATGCT
1102CAN .        CAGGCCTCCAACATGATTCTTCTGCTGAAGGAAGTCCGATCTCAGCTGGATGCTTATGCT
588LAOS .        CAGGCCTCCAACATGATTCTTCTGCTGAAGGAAGTCCGATCTCAGCTGGATGCTTATGCT
1596AT .         CAGGCCTCCAACATGATTCTTCTGCTGAAGGAAGTCCGATCTCAGCTGGATGCTTATGCT
1720CAM .        CAGGCCTCCAACATGATTCTTCTGCTGAAGGAAGTCCGATCTCAGCTGGATGCTTATGCT
1722CAM .        CAGGCCTCCAACATGATTCTTCTGCTGAAGGAAGTCCGATCTCAGCTGGATGCTTATGCT
1724CAM .        CAGGCCTCCAACATGATTCTTCTGCTGAAGGAAGTCCGATCTCAGCTGGATGCTTATGCT
334USA .         CAGGCCTCCAACATGATTCTTCTGCTGAAGGAAGTCCGATCTCAGCTGGATGCTTATGCT
335USA .         CAGGCCTCCAACATGATTCTTCTGCTGAAGGAAGTCCGATCTCAGCTGGATGCTTATGCT
1081WIS .        CAGGCCTCTAACATGATTCTTCTGCTGAAGGAAGTCCGATCTCAGCTGGATGCTTATGCT
1069WIS .        CAGGCCTCCAACATGATTCTTCTGCTGAAGGAAGTCCGATCTCAGCTGGATGCTTATGCT
1107CAN .        CAGGCCTCCAACATGATTCTTCTGCTGAAGGAAGTCCGATCTCAGCTGGATGCTTATGCT
1108PEN .        CAGGCCTCCAACATGATTCTTCTGCTGAAGGAAGTCCGATCTCAGCTGGATGCTTATGCT
1110CAN .        CAGGCCTCCAACATGATTCTTCTACTGAAGGAAGTCCGATCTCAGCTGGATGCTTATGCT
JBSER241 .       CAGGCCTCCAACATGGTTCTTCTGCTCAAGGAAGTCCGATCTCAGCTGGATGCTTATGCT
246PHI .         CAGGCCTCCAACATGGTTCTTCTGCTCAAGGAAGTCCGATCTCAGCTGGATGCTTATGCT
51SA .           CAGGCCTCCAACATGGTTCTTCTGCTCAAGGAAGTCCGATCTCAGCTGGATGCTTATGCT
2618ETHI .       CAGGCCTCCAACATGGTTCTTCTGCTCAAGGAAGTCCGATCTCAGCTGGATGCTTATGCT
1068WIS .        CAGGCCTCCAACATGATTCTTCTGCTGAAGGAAGTCCGATCTCAGCTGGATGCTTATGCT
238CR .          CAGGCCTCCAACATGGTTCTTCTGCTCAAGGAAGTCCGATCTCAGCTGGATGCTTATGCT
NR5555 .         CAGGCCTCCAACATGGTTCTTCTGCTCAAGGAAGTCCGATCTCAGCTGGATGCTTATGCT
NR6839 .         CAGGCCTCCAACATGGTTCTTCTGCTCAAGGAAGTCCGATCTCAGCTGGATGCTTATGCT
2624ETHI .       CAGGCCTCCAACATGGTTCTTCTGCTCAAGGAAGTCCGATCTCAGCTGGATGCTTATGCT
845EGY .         CAGGCCTCCAACATGGTTCTTCTGCTCAAGGAAGTCCGATCTCAGCTGGATGCTTATGCT
JBPER12 .        XXXXXXXXXXXXXXXXXXXXXXXXXXXXXXXXXXXXXXXXXXXXXXXXXXXXXXXXXXXX
1717VIET .       CAGGCCTCCAACATGGTTCTTCTGCTGAAGGAAGTCCGATCTCAGCTGGATGCTTATGCT
709BRAZ .        CAGGCCTCCAACATGGTTCTTCTGCTGAAGGAAGTCCGATCTCAGCTGGATGCTTATGCT
878IRAN .        CAGGCCTCCAACATGGTTCTTCTGCTGAAGGAAGTCCGATCTCAGCTGGATGCTTATGCT
239CR .          CAGGCCTCCAACATGGTTCTTCTGCTGAAGGAAGTCCGATCTCAGCTGGATGCTTATGCT
202COM .         CAGGCCTCCAACATGGTTCTTCTGCTGAAGGAAGTCCGATCTCAGCTGGATGCTTATGCT
1064MEX .        CAGGCCTCCAACATGGTTCTTCTGCTGAAGGAAGTCCGATCTCAGCTGGATGCTTATGCT
596BRAZ .        CAGGCCTCCAACATGGTTCTTCTGCTGAAGGAAGTCCGATCTCAGCTGGATGCTTATGCT
333USA .         CAGGCCTCCAACATGGTTCTTCTGCTGAAGGAAGTCCGATCTCAGCTGGATGCTTATGCT
2610ETHI .       CAGGCCTCCAACATGGTTCTTCTGCTGAAGGAAGTCCGATCTCAGCTGGATGCTTATGCT
1044RW .         CAGGCCTCCAACATGGTTCTCTTGCTGAAGGAAGTCCGATCTCAGCTGGATGCTTATGCT
JBNZ111 .        CAGGCCTCCAACATGATTCTTCTGCTGAAGGAAGTTCGATCTCAGCTGGATGCTTATGCT
JBNZ12 .         CAGGCCTCCAACATGGTTCTTCTGCTGAAGGAAGTCCGATCTCAGCTGGATGCTTATGCT
JBNZ24 .         CAGGCCTCCAACATGATTCTTCTGCTGAAGGAAGTTCGATCTCAGCTGGATGCTTATGCT
693CHINA .       CAGGCCTCCAACATGGTTCTTCTGCTGAAGGAAGTCCGATCTCAGCTGGATGCTTATGCT
1058RW .         CAGGCCTCCAACATGGTTCTCTTGCTGAAGGAAGTCCGATCTCAGCTGGATGCTTATGCT
590AUS .         CAGGCCTCCAACATGGTTCTTCTGCTGAAGGAAGTCCGATCTCAGCTGGATGCTTATGCT
53SA .           CAGGCCTCCAACATGGTTCTTCTGCTGAAGGAAGTCCGATCTCAGCTGGATGCTTATGCT
1075WIS .        CAGGCCTCTAACATGATTCTTCTGCTGAAGGAAGTCCGATCTCAGCTGGATGCTTATGCT
939GER .         CAGGCCTCTAACATGATTCTTCTGCTGAAGGAAGTCCGATCTCAGCTGGATGCTTATGCT
GA3804 .         CAGGCCTCTAACATGATTCTTCTGCTGAAGGAAGTCCGATCTCAGCTGGATGCTTATGCT
1599AT .         CAGGCCTCCAACATGATTCTTTTGCTGAAGGAAGTCCGATCTCAGCTGGATGCTTATGCT
1941AT .         CAGGCCTCCAACATGATTCTTCTGCTGAAGGAAGTCCGATCTCAGCTGGATGCTTATGCT
291NE .          CAGGCCTCCAACATGGTTCTTCTGCTGAAGGAAGTCCGATCTCAGCTGGATGCTTATGCT
2301SAR .        CAGGCCTCCAACATGATTCTTTTGCTGAAGGAAGTCCGATCTCAGCTGGATGCTTATGCT
2313SAR .        CAGGCCTCCAACATGATTCTTCTGCTGAAGGAAGTCCGATCTCAGCTGGATGCTTATGCT
271NE .          CAGGCCTCCAACATGATTCTTCTGCTGAAGGAAGTCCGATCCCAGCTGGATGCTTATGCT
1935GER .        CAGGCCTCCAACATGATTCTTTTGCTGAAGGAAGTCCGATCTCAGCTGGATGCTTATGCT
JBRSA122 .       CAGGCCTCCAACATGATTCTTTTGCTGAAGGAAGTCCGATCTCAGCTGGATGCTTATGCT
276NE .          CAGGCCTCTAACATGATTCTTCTGCTGAAGGAAGTCCGATCTCAGCTGGATGCTTATGCT
272NE .          CAGGCCTCTAACATGATTCTTCTGCTGAAGGAAGTCCGATCTCAGCTGGATGCTTATGCT
2673ETHI .       CAGGCCTCCAACATGATTCTTCTGCTGAAGGAAGTCCGATCTCAGCTGGATGCTTATGCT
1084AUSW .       CAGGCCTCCAACATGATTCTTCTGCTGAAGGAAGTCCGATCTCAGCTGGATGCTTATGCT
JBPER62 .        CAGGCCTCCAACATGATTCTTCTGCTGAAGGAAGTCCGATCTCAGCTGGATGCTTATGCT
JBNZ72 .         CAGGCCTCCAACATGGTTCTTCTGCTGAAGGAAGTCCGATCTCAGCTGGATGCTTATGCT
1065MEX .        XXXXXXXXXXXXXXXXXXXXXXXXXXXXXXXXXXXXXXXXXXXTCAGCTGGATGCTTATGCT
646HAWAI .       CAGGCTTCCAACATGGTTCTTCTGCTGAAGGAAGTCCGATCTCAGCTGGATGCTTATGCT
727INDIA .       CAGGCTTCCAACATGGTTCTTCTGCTGAAGGAAGTCCGATCTCAGCTGGATGCTTATGCT
743MALE .        CAGGCTTCCAACATGATTCTTCTGCTGAAGGAAGTCCGATCTCAGCTGGATGCTTATGCT
1059MEX .        XXXXXXXXXXXXXXXXXXXXXXXXXXXXXXXXXXXXXXXXXXXXXAGCTGGATGCTTATGCT
1061RW .         CAGGCCTCCAACATGGTTCTTCTGCTCAAGGAAGTCCGATCTCAGCTGGATGCTTATGCT
1066MEX .        CAGGCCTCCAACATGGTTCTTCTGCTGAAGGAAGTCCGATCTCAGCTGGATGCTTATGCT
1095BALI .       CAGGCCTCCAACATGGTTCTTCTGCTCAAGGAAGTCCGATCTCAGCTGGATGCTTATGCT
1505MAUR .       CAGGCCTCCAACATGGTTCTTCTGCTGAAGGAAGTCCGATCTCAGCTGGATGCTTATGCT
1818ETHI .       CAGGCCTCCAACATGGTTCTTCTGCTGAAGGAAGTCCGATCTCAGCTGGATGCTTATGCT
1934AT .         CAGGCCTCTAACATGATTCTTCTGCTGAAGGAAGTCCGATCTCAGCTGGATGCTTATGCT
245CR .          CAGGCTTCCAACATGGTTCTTCTGCTGAAGGAAGTCCGATCTCAGCTGGATGCTTATGCT
2710ETHI .       CAGGCTTCCAACATGGTTCTTCTGCTGAAGGAAGTCCGATCTCAGCTGGATGCTTATGCT
274NE .          CAGGCTTCCAACATGGTTCTTCTGCTGAAGGAAGTCCGATCTCAGCTGGATGCTTATGCT
3408PNG .        CAGGCCTCCAACATGGTTCTTCTGCTGAAGGAAGTCCGATCTCAGCTGGATGCTTATGCT
3409PNG .        CAGGCCTCCAACATGGTTCTTCTGCTGAAGGAAGTCCGATCTCAGCTGGATGCTTATGCT
410MALAY .       CAGGCCTCCAACATGGTTCTCCTGCTGAAGGAAGTCCGATCTCAGCTGGACGCCTATGCT
836EGY .         CAGGCCTCCAACATGGTTCTTCTGCTGAAGGAAGTCCGATCTCAGCTGGATGCTTATGCT
837EGY .         CAGGCCTCCAACATGGTTCTTCTGCTGAAGGAAGTCCGATCTCAGCTGGATGCTTATGCT
JBRO111 .        CAGGCCTCCAACATGGTTCTTCTGCTGAAGGAAGTCCGATCTCAGCTGGATGCTTATGCT
brunneoviridis4  CAGGCCTCCAACATGGTTCTTCTGCTCAAGGAAGTCCGATCTCAGCTGGATGCTTATGCT
brunneoviridis1  CAGGCCTCCAACATGGTTCTTCTGCTCAAGGAAGTCCGATCTCAGCTGGATGCTTATGCT
pleurotumCPK211  CAGGCTTCCAACATGATTCTTCTGCTCAAGGAAGTCCGATCTCAGCTGGATGCTTATGCT
PLEUROTICOLA .   CAGGCTTCCAACATGATTCTTCTGCTCAAGGAAGTCCRATCTCAGCTGGATGCTTATGCT
epimycesCPK1981  CAGGCCTCCAACATGGTTCTTCTGCTCAAGGAAGTCCGATCTCAACTGGATGCTTATGCT
alniCPK3124 ...  CAGGCCTCCAACATGATTCTTCTGCTCAAGGAAGTCCGAACTCAGCTAGATGCTTATGCT
CPK5TAWA .       CAGGCCTCCAACATGATTCTTCTGCTCAAGGAAGTCCGAACTCAGCTGGATGCTTATGCT
2974lobau .      CAGGCTTCCAACATGGTTCTTCTGCTGAAGGAAGTCCGATCTCAGCTGGATGCTTATGCT
2975lobau .      CAGGCTTCCAACATGGTTCTTCTGCTGAAGGAAGTCCGATCTCAGCTGGATGCTTATGCT
aggressivumCPK3  CAGGCCTCCAACATGGTTCTTCTGCTCAAGGAAGTCCGATCTCAGCTGGATGCTTATGCT
atrogelatinosaC  CAGGCCTCCAACATGATTCTTCTGCTGAAGGAAGTCCGATCTCAGCTGGATGCTTATGCT
```

```
atrogelatinosaC  CAGGCCTCCAACATGATTCTTCTGCTGAAGGAAGTCCGATCTCAGCTGGATGCTTATGCT
cerinumCPK293 .  CAGGCCTCCAACATGATTCTTCTGCTGAAGGAAGTCCGATCTCAGCTGGATGCCTACGCT
cerinumCPK854 .  CAGGCCTCCAACATGATTCTTCTGCTGAAGGAAGTCCGATCTCAGCTGGATGCCTACGCT
tomentosumCPK88  CAGGCCTCCAACATGATTCTTCTGCTGAAGGAAGTCCGATCTCAGCTGGATGCCTATGCT
stramineaGJS028  CAGGCCGCCAACATGGTTCTTCTGTTGAAGGAGGTCCGATCTCAGCTGGATGCTTATGCT
velutinumCPK312  CAGGCCGCCAACATGGTTCTTCTGCTGAAGGAGGTCCGATCTCAGCTGGATGCCTATGCT
catoptronGJS027  CAGGCCTCCAACATGGTTCTTCTGCTGAAGGAAGTCCGATCTCAGCTGGATGCCTATGCT
                 ############################################################


                        910       920       930       940       950       960
                 =========+=========+=========+=========+=========+=========+
1093CAN .        GCCCAGTACGCCCCTGGCTACCACTTCCTCCTCACCATTGCCGCCCCAGCTGGCAAGGAT
1052SA .         GCCCAGTACGCCCCTGGCTACCACTTCCTCCTCACCATTGCCGCCCCAGCTGGCAAGGAT
1116CAN .        GCCCAGTACGCCCCTGGCTACCACTTCCTCCTCACCATTGCCGCCCCAGCTGGCAAGGAT
1070EIRE .       GCCCAGTACGCCCCTGGCTACCACTTCCTCCTCACCATTGCCGCCCCAGCTGGCAAGGAT
1099CAN .        GCCCAGTACGCCCCTGGCTACCACTTCCTCCTCACCATTGCCGCCCCAGCTGGCAAGGAT
1104CAN .        GCCCAGTACGCCCCTGGCTACCACTTCCTCCTCACCATTGCCGCCCCAGCTGGCAAGGAT
1087CAN .        GCCCAGTACGCCCCTGGCTACCACTTCCTCCTCACCATTGCCGCCCCAGCTGGCAAGGAT
360UK .          GCCCAGTACGCCCCTGGCTACCACTTCCTCCTCACCATTGCCGCCCCAGCTGGCAAGGAT
2111HUN .        GCCCAGTACGCCCCTGGCTACCACTTCCTCCTCACCATTGCCGCCCCAGCTGGCAAGGAT
217RUS .         --------------TGGCTACCACTTCCTCCTCACCATTGCCGCCCCAGCTGGCAAGGAT
204UK .          GCCCAGTACGCCCCTGGCTACCACTTCCTCCTAACCATTGCCGCCCCAGCTGGCAAGGAT
206RUS .         GCCCAGTACGCCCCTGGCTACCACTTCCTCCTCACCATTGCCGCCCCAGCTGGCAAGGAT
261SIB .         GCCCAGTACGCCCCTGGCTACCACTTCCTCCTCACCATTGCCGCCCCAGCTGGCAAGGAT
265SIB .         GCCCAGTACGCCCCTGGCTACCACTTCCTCCTAACCATTGCCGCCCCAGCTGGCAAGGAT
2784THAI .       GCCCAGTATGCCCCTGGCTACCACTTCCTCCTCACCATTGCCGCCCCAGCTGGCAAGGAC
838EGY .         GCCCAGTATGCCCCTGGCTACCACTTCCTCCTCACCATTGCCGCCCCAGCTGGCAAGGAC
1102CAN .        GCCCAGTATGCCCCTGGCTACCACTTCCTCCTCACCATTGCCGCCCCAGCTGGCAAGGAC
588LAOS .        GCCCAGTATGCCCCTGGCTACCACTTCCTCCTCACCATTGCCGCCCCAGCTGGCAAGGAC
1596AT .         GCCCAGTATGCCCCTGGCTACCACTTCCTCCTCACCATTGCCGCCCCAGCTGGCAAGGAC
1720CAM .        GCCCAGTATGCCCCTGGCTACCACTTCCTCCTCACCATTGCCGCCCCAGCTGGCAAGGAC
1722CAM .        GCCCAGTATGCCCCTGGCTACCACTTCCTCCTCACCATTGCCGCCCCAGCTGGCAAGGAC
1724CAM .        GCCCAGTATGCCCCTGGCTACCACTTCCTCCTCACCATTGCCGCCCCAGCTGGCAAGGAC
334USA .         GCCCAGTATGCCCCTGGCTACCACTTCCTCCTCACCATTGCCGCCCCAGCTGGCAAGGAC
335USA .         GCCCAGTATGCCCCTGGCTACCACTTCCTCCTCACCATTGCCGCCCCAGCTGGCAAGGAC
1081WIS .        GCCCAGTATGCCCCTGGCTACCACTTCCTCCTCACCATTGCCGCCCCAGCTGGCAAGGAC
1069WIS .        GCCCAGTATGCCCCTGGCTACCACTTCCTCCTCACCATTGCCGCCCCAGCTGGCAAGGAC
1107CAN .        GCCCAGTATGCCCCTGGCTACCACTTCCTCCTCACCATTGCCGCCCCAGCTGGCAAGGAC
1108PEN .        GCCCAGTATGCCCCTGGCTACCACTTCCTCCTCACCATTGCCGCCCCAGCTGGCAAGGAC
1110CAN .        GCCCAGTATGCCCCTGGCTACCACTTCCTCCTCACCATTGCCGCCCCAGCTGGCAAGGAC
JBSER241 .       GCCCAGTATGCCCCTGGCTACCACTTCCTCCTCACCATTGCCGCACCAGCTGGCAAGGAT
246PHI .         GCCCAGTATGCCCCTGGCTACCACTTCCTCCTCACCATTGCCGCACCAGCTGGCAAGGAT
51SA .           GCCCAGTATGCCCCTGGCTACCACTTCCTCCTCACCATTGCCGCACCAGCTGGCAAGGAT
2618ETHI .       GCCCAGTATGCCCCTGGCTACCACTTCCTCCTCACCATTGCCGCACCAGCTGGCAAGGAT
1068WIS .        GCCCAGTATGCCCCTGGCTACCACTTCCTCCTCACCATTGCCGCCCCAGCTGGCAAGGAC
238CR .          GCCCAGTATGCCCCTGGCTACCACTTCCTCCTCACCATTGCCGCACCAGCTGGCAAGGAT
NR5555 .         GCCCAGTATGCCCCTGGCTACCACTTCCTCCTCACCATTGCCGCACCAGCTGGCAAGGAT
NR6839 .         GCCCAGTATGCCCCTGGCTACCACTTCCTCCTCACCATTGCCGCACCAGCTGGCAAGGAT
2624ETHI .       GCCCAGTATGCCCCTGGCTACCACTTCCTCCTCACCATTGCCGCACCAGCTGGCAAGGAT
845EGY .         GCCCAGTATGCCCCTGGCTACCACTTCCTCCTCACCATTGCCGCACCAGCTGGCAAGGAT
JBPER12 .        XXXXXXXXXXXXXXXXXXXXXXXXXXXXXXXXXXXXXXXXXXXXXXXXXXXXXXXXXXXX
1717VIET .       GCCCAGTACGCCCCTGGCTACCACTTCCTCCTCACCATTGCCGCCCCAGCTGGCAAGGAT
709BRAZ .        GCCCAGTACGCCCCTGGCTACCACTTCCTCCTCACCATTGCCGCCCCAGCTGGCAAGGAT
878IRAN .        GCCCAGTACGCCCCTGGCTACCACTTCCTCCTCACCATTGCCGCCCCAGCTGGCAAGGAT
239CR .          GCCCAGTACGCCCCTGGCTACCACTTCCTCCTCACCATTGCCGCCCCAGCTGGCAAGGAT
202COM .         GCCCAGTACGCCCCTGGCTACCACTTCCTCCTCACCATTGCCGCCCCAGCTGGCAAGGAT
1064MEX .        GCCCAGTACGCCCCTGGCTACCACTTCCTCCTCACCATTGCCGCCCCAGCTGGCAAGGAT
596BRAZ .        GCCCAGTACGCCCCTGGCTACCACTTCCTCCTCACCATTGCCGCCCCAGCTGGCAAGGAT
333USA .         GCCCAGTACGCCCCTGGCTACCACTTCCTCCTCACCATTGCCGCCCCAGCTGGCAAGGAT
2610ETHI .       GCCCAGTACGCCCCTGGCTACCACTTCCTCCTCACCATTGCCGCCCCAGCTGGCAAGGAT
1044RW .         GCCCAGTACGCCCCTGGCTACCACTTCCTCCTCACCATTGCCGCCCCAGCTGGCAAGGAT
JBNZ111 .        GCCCAGTATGCCCCTGGCTACCACTTCCTCCTCACCATTGCCGCCCCAGCTGGCAAGGAT
JBNZ12 .         GCCCAGTACGCCCCTGGCTACCACTTCCTCCTCACCATTGCCGCCCCAGCTGGCAAGGAT
JBNZ24 .         GCCCAGTATGCCCCTGGCTACCACTTCCTCCTCACCATTGCCGCCCCAGCTGGCAAGGAT
693CHINA .       GCCCAGTACGCCCCTGGCTACCACTTCCTCCTCACCATTGCCGCCCCAGCTGGCAAGGAT
1058RW .         GCCCAGTACGCCCCTGGCTACCACTTCCTCCTCACCATTGCCGCCCCAGCTGGCAAGGAT
590AUS .         GCCCAGTACGCCCCTGGCTACCACTTCCTCCTCACCATTGCCGCCCCAGCTGGCAAGGAT
53SA .           GCCCAGTACGCCCCTGGCTACCACTTCCTCCTCACCATTGCCGCCCCAGCTGGCAAGGAT
1075WIS .        GCCCAGTACGCCCCTGGCTACCACTTCCTCCTCACCATTGCCGCCCCAGCTGGCAAGGAT
939GER .         GCCCAGTACGCCCCTGGCTACCACTTCCTCCTCACCATTGCCGCCCCAGCTGGCAAGGAT
GA3804 .         GCCCAGTACGCCCCTGGCTACCACTTCCTCCTCACCATTGCCGCCCCAGCTGGCAAGGAT
1599AT .         GCCCAGTACGCCCCTGGCTACCACTTCCTCCTCACCATTGCCGCCCCAGCTGGCAAGGAT
1941AT .         GCCCAGTACGCCCCTGGCTACCACTTCCTCCTCACCATTGCCGCCCCAGCTGGCAAGGAT
291NE .          GCCCAGTACGCCCCTGGCTACCACTTCCTCCTCACCATTGCCGCCCCAGCTGGCAAGGAT
2301SAR .        GCCCAGTACGCCCCTGGCTACCACTTCCTCCTCACCATTGCCGCCCCAGCTGGCAAGGAT
2313SAR .        GCCCAGTACGCCCCTGGCTACCACTTCCTCCTCACCATTGCCGCCCCAGCTGGCAAGGAT
271NE .          GCCCAGTACGCCCCTGGCTACCACTTCCTCCTCACCATTGCCGCCCCAGCTGGCAAGGAT
1935GER .        GCCCAGTACGCCCCTGGCTACCACTTCCTCCTCACCATTGCCGCCCCAGCTGGCAAGGAT
JBRSA122 .       GCCCAGTACGCCCCTGGCTACCACTTCCTCCTCACCATTGCCGCCCCAGCTGGCAAGGAT
276NE .          GCCCAGTACGCCCCTGGCTACCACTTCCTCCTCACCATTGCCGCCCCAGCTGGCAAGGAT
272NE .          GCCCAGTACGCCCCTGGCTACCACTTCCTCCTCACCATTGCCGCCCCAGCTGGCAAGGAT
2673ETHI .       GCCCAGTACGCCCCTGGCTACCACTTCCTTCTCACCATTGCCGCCCCAGCTGGCAAGGAT
1084AUSW .       GCCCAGTACGCCCCTGGCTACCACTTCCTTCTCACCATTGCCGCCCCAGCTGGCAAGGAT
JBPER62 .        GCCCAGTACGCCCCTGGCTACCACTTCCTTCTCACCATTGCCGCCCCAGCTGGCAAGGAT
JBNZ72 .         GCCCAGTACGCCCCTGGCTACCACTTCCTCCTCACCATTGCCGCCCCAGCTGGCAAGGAT
1065MEX .        GCCCAGTACGCCCCTGGCTACCACTTCCTCCTCACCATTGCCGCCCCAGCTGGCAAAGAT
646HAWAI .       GCCCAGTACGCCCCTGGCTACCACTTCCTCCTCACCATTGCCGCCCCAGCTGGCAAAGAT
727INDIA .       GCCCAGTACGCCCCTGGCTACCACTTCCTCCTCACCATTGCCGCCCCAGCTGGCAAAGAT
743MALE .        GCCCAGTACGCCCCTGGCTATCACTTCCTCCTCACCATTGCCGCCCCAGCTGGCAAAGAT
1059MEX .        GCCCAGTACGCCCCTGGCTACCACTTCCTCCTCACCATTGCCGCCCCAGCTGGCAAAGAT
1061RW .         GCCCAGTATGCCCCTGGCTACCACTTCCTCCTCACCATTGCCGCACCAGCTGGCAAGGAT
1066MEX .        GCCCAGTACGCCCCTGGCTACCACTTCCTCCTCACCATTGCCGCCCCAGCTGGCAAGGAC
1095BALI .       GCCCAGTATGCCCCTGGCTACCACTTCCTCCTCACCATTGCCGCACCAGCTGGCAAGGAT
1505MAUR .       GCCCAGTACGCCCCTGGCTACCACTTCCTCCTCACCATTGCCGCCCCAGCTGGCAAGGAT
1818ETHI .       GCCCAGTACGCCCCTGGCTACCACTTCCTCCTCACCATTGCCGCCCCAGCTGGCAAGGAT
1934AT .         GCCCAGTACGCCCCTGGCTACCACTTCCTCCTCACCATTGCCGCCCCAGCTGGCAAGGAT
245CR .          GCCCAGTACGCCCCTGGCTACCACTTCCTCCTCACCATTGCCGCCCCAGCTGGCAAGGAC
2710ETHI .       GCCCAGTACGCCCCTGGCTACCACTTCCTCCTCACCATTGCCGCCCCAGCTGGCAAGGAC
274NE .          GCCCAGTACGCCCCTGGCTACCACTTCCTCCTCACCATTGCCGCCCCAGCTGGCAAGGAC
3408PNG .        GCCCAGTACGCCCCTGGCTACCACTTCCTCCTCACCATTGCCGCCCCAGCTGGCAAGGAT
3409PNG .        GCCCAGTACGCCCCTGGCTACCACTTCCTCCTCACCATTGCCGCCCCAGCTGGCAAGGAT
410MALAY .       GCCCAGTACGCCCCTGGCTACCACTTCCTCCTCACCATTGCTGCCCCAGCTGGCAAGGAT
836EGY .         GCCCAGTACGCCCCTGGCTACCACTTCCTCCTCACCATTGCCGCCCCAGCTGGCAAGGAT
837EGY .         GCCCAGTACGCCCCTGGCTACCACTTCCTCCTCACCATTGCCGCCCCAGCTGGCAAGGAT
JBRO111 .        GCCCAGTACGCCCCTGGCTACCACTTCCTCCTCACCATTGCCGCCCCAGCTGGCAAGGAT
brunneoviridis4  GCCCAGTACGCCCCTGGCTACCACTTCCTCCTCACCATTGCTGCCCCAGCTGGCAAGGAT
brunneoviridis1  GCCCAGTACGCCCCTGGCTACCACTTCCTCCTCACCATTGCTGCCCCAGCTGGCAAGGAT
pleurotumCPK211  GCCCAATACGCCCCTGGCTACCACTTCCTCCTCACCATTGCTGCCCCAGCTGGCAAGGAT
PLEUROTICOLA .   GCCCAATACSCCCCCGGCTACCACTTCCTCCTMACCATTGCTGCCCCAGCTGGCAAGGAT
epimycesCPK1981  GTCCAGTACGCCCCTGGCTACCACTTCCTCCTCACCATTGCTGCCCCAGCTGGCAAGGAA
alniCPK3124 ...  GCCCAGTACGCTCCTGGCTACCACTTCCTCCTCACCATTGCTGCCCCAGCTGGCAAGGAC
CPK5TAWA .       GCCCAGTACGCTCCTGGCTACCACTTCCTCCTCACCATTGCTGCCCCAGCTGGCAAGGAC
2974lobau .      GCCCAGTACGCCCCTGGCTACCACTTCCTCCTCACCATTGCCGCCCCAGCTGGCAAGGAT
2975lobau .      GCCCAGTACGCCCCTGGCTACCACTTCCTCCTCACCATTGCCGCCCCAGCTGGCAAGGAT
aggressivumCPK3  GCCCAGTACGCCCCTGGCTACCACTTCCTCCTCACCATTGCTGCCCCAGCTGGCAAGGAC
atrogelatinosaC  GCCCAGTACGCCCCTGGCTACCACTTCCTCCTCACCATTGCTGCCCCGGCTGGCAAGGAT
```

```
atrogelatinosaC  GCCCAGTACGCCCCTGGCTACCACTTCCTCCTCACCATTGCTGCCCCGGCTGGCAAGGAT
cerinumCPK293 .  GCACAGTACGCCCCTGGCTACCACTTCCTCCTCACCATTGCTGCCCCGGCTGGCAAGGAT
cerinumCPK854 .  GCACAGTACGCCCCTGGCTACCACTTCCTCCTCACCATTGCTGCCCCGGCTGGCAAGGAT
tomentosumCPK88  GCACAGTACGCCCCTGGCTACCACTTCCTCCTCACCATTGCTGCCCCGGCTGGCAAGGAT
stramineaGJS028  GCCCAGTACGCCCCTGGCTACCACTTCCTCCTAACCATTGCTGCCCCAGCTGGCAAGGAT
velutinumCPK312  GCCCAGTACGCCCCTGGCTACCATTTCCTCCTCACTATTGCTGCCCCGGCTGGCAAGGAT
catoptronGJS027  GCACAATACGCCCCTGGCTACCACTTCCTCCTCACCATTGCTGCCCCAGCTGGCAAGGAT
                 ############################################################


                        970       980       990      1000      1010      1020
                 =========+=========+=========+=========+=========+=========+
1093CAN .        AACTACTCCAAGCTGCGCCTGGCTGATCTCGGCCAAGTCCTCGACTACATCAACCTCATG
1052SA .         AACTACTCCAAGCTGCGCCTGGCTGATCTCGGCCAAGTCCTCGACTACATCAACCTCATG
1116CAN .        AACTACTCCAAGCTGCGCCTGGCTGATCTCGGCCAAGTCCTCGACTACATCAACCTCATG
1070EIRE .       AACTACTCCAAGCTGCGCCTGGCTGATCTCGGCCAAGTCCTCGACTACATCAACCTCATG
1099CAN .        AACTACTCCAAGCTGCGCCTGGCTGATCTCGGCCAAGTCCTCGACTACATCAACCTCATG
1104CAN .        AACTACTCCAAGCTGCGCCTGGCTGATCTCGGCCAAGTCCTCGACTACATCAACCTCATG
1087CAN .        AACTACTCCAAGCTGCGCCTGGCTGATCTCGGCCAAGTCCTCGACTACATCAACCTCATG
360UK .          AACTACTCCAAGCTGCGCCTGGCTGATCTCGGCCAAGTCCTCGACTACATCAACCTCATG
2111HUN .        AACTACTCCAAGCTGCGCCTGGCTGATCTCGGCCAAGTCCTCGACTACATCAACCTCATG
217RUS .         AACTACTCCAAGCTGCGCCTGGCTGATCTCGGCCAAGTCCTCGACTACATCAACCTCATG
204UK .          AACTACTCCAAGCTGCGCCTGGCTGATCTCGGCCAAGTCCTCGACTACATCAACCTCATG
206RUS .         AACTACTCCAAGCTGCGCCTGGCTGATCTCGGCCAAGTCCTCGACTACATCAACCTCATG
261SIB .         AACTACTCCAAGCTGCGCCTGGCTGATCTCGGCCAAGTCCTCGACTACATCAACCTCATG
265SIB .         AACTACTCCAAGCTGCGCCTGGCTGATCTCGGCCAAGTCCTCGACTACATCAACCTCATG
2784THAI .       AACTACTCCAAGCTGCGCCTGGCTGATCTTGGCCAAGTCCTCGACTACATCAACCTCATG
838EGY .         AACTACTCCAAGCTGCGCCTGGCTGATCTTGGCCAAGTCCTCGACTACATCAACCTCATG
1102CAN .        AACTACTCCAAGCTGCGCCTGGCTGATCTTGGCCAAGTCCTCGACTACATCAACCTCATG
588LAOS .        AACTACTCCAAGCTGCGCCTGGCTGATCTTGGCCAAGTCCTCGACTACATCAACCTCATG
1596AT .         AACTACTCCAAGCTGCGCCTGGCTGATCTTGGCCAAGTCCTCGACTACATCAACCTCATG
1720CAM .        AACTACTCCAAGCTGCGCCTGGCTGATCTTGGCCAAGTCCTCGACTACATCAACCTCATG
1722CAM .        AACTACTCCAAGCTGCGCCTGGCTGATCTTGGCCAAGTCCTCGACTACATCAACCTCATG
1724CAM .        AACTACTCCAAGCTGCGCCTGGCTGATCTTGGCCAAGTCCTCGACTACATCAACCTCATG
334USA .         AACTACTCCAAGCTGCGCCTGGCTGATCTTGGCCAAGTCCTCGACTACATCAACCTCATG
335USA .         AACTACTCCAAGCTGCGCCTGGCTGATCTTGGCCAAGTCCTCGACTACATCAACCTCATG
1081WIS .        AACTACTCCAAGCTGCGCCTGGCTGATCTTGGCCAAGTCCTCGACTACATCAACCTCATG
1069WIS .        AACTACTCCAAGCTGCGCCTGGCTGATCTTGGCCAAGTCCTCGACTACATCAACCTCATG
1107CAN .        AACTACTCCAAGCTGCGCCTGGCTGATCTTGGCCAAGTCCTCGACTACATCAACCTCATG
1108PEN .        AACTACTCCAAGCTGCGCCTGGCTGATCTTGGCCAAGTCCTCGACTACATCAACCTCATG
1110CAN .        AACTACTCCAAGCTGCGCCTGGCTGATCTTGGCCAAGTCCTCGACTACATCAACCTCATG
JBSER241 .       AACTACTCCAAGCTGCGCCTGGCCGATCTTGGCCAAGTCCTCGACTACATCAACCTCATG
246PHI .         AACTACTCCAAGCTGCGCCTGGCCGATCTTGGCCAAGTTCTCGACTACATCAACCTCATG
51SA .           AACTACTCCAAGCTGCGCCTGGCCGATCTTGGCCAAGTCCTCGACTACATCAACCTCATG
2618ETHI .       AACTACTCCAAGCTGCGCCTGGCCGATCTTGGCCAAGTCCTCGACTACATCAACCTCATG
1068WIS .        AACTACTCCAAGCTGCGCCTGGCTGATCTTGGCCAAGTCCTCGACTACATCAACCTCATG
238CR .          AACTACTCCAAGCTGCGCCTGGCCGATCTTGGCCAAGTTCTCGACTACATCAACCTCATG
NR5555 .         AACTACTCCAAGCTGCGCCTGGCCGATCTTGGCCAAGTCCTCGACTACATCAACCTCATG
NR6839 .         AACTACTCCAAGCTGCGCCTGGCCGATCTTGGCCAAGTCCTCGACTACATCAACCTCATG
2624ETHI .       AACTACTCCAAGCTGCGCCTGGCCGATCTTGGCCAAGTCCTCGACTACATCAACCTCATG
845EGY .         AACTACTCCAAGCTGCGCCTGGCCGATCTTGGCCAAGTCCTCGACTACATCAACCTCATG
JBPER12 .        XXXXXXXXXXXXXXXXXXXXXXXXXXXXXXXXXXXXXXXXXXXXXXXXXXXXXXXXXXXX
1717VIET .       AACTACTCCAAGCTGCGCCTGGCCGATCTTGGCCAAGTCCTCGACTACATCAACCTCATG
709BRAZ .        AACTACTCCAAGCTGCGCCTGGCCGATCTTGGCCAAGTCCTCGACTACATCAACCTCATG
878IRAN .        AACTACTCCAAGCTGCGCCTGGCCGATCTTGGCCAAGTCCTCGACTACATCAACCTCATG
239CR .          AACTACTCCAAGCTGCGCCTGGCCGATCTTGGCCAAGTCCTCGACTACATCAACCTCATG
202COM .         AACTACTCCAAGCTGCGCCTGGCCGATCTTGGCCAAGTCCTCGACTACATCAACCTCATG
1064MEX .        AACTACTCCAAGCTGCGCCTGGCCGATCTTGGCCAAGTCCTCGACTACATCAACCTCATG
596BRAZ .        AACTACTCCAAGCTGCGCCTGGCCGATCTTGGCCAAGTCCTCGACTACATCAACCTCATG
333USA .         AACTACTCCAAGCTGCGCCTGGCCGATCTTGGCCAAGTCCTCGACTACATCAACCTCATG
2610ETHI .       AACTACTCCAAGCTGCGCCTGGCTGATCTCGGCCAAGTCCTCGACTACATCAACCTCATG
1044RW .         AACTACTCCAAGCTGCGCCTGGCTGATCTCGGCCAAGTCCTCGACTACATCAACCTCATG
JBNZ111 .        AACTACTCCAAGCTGCGCCTGGCTGATCTTGGCCAAGTCCTCGACTACATCAACCTCATG
JBNZ12 .         AACTACTCCAAGCTGCGCCTGGCTGATCTTGGCCAAGTCCTTGACTACATCAACCTCATG
JBNZ24 .         AACTACTCCAAGCTGCGCCTGGCTGATCTTGGCCAAGTCCTCGACTACATCAACCTCATG
693CHINA .       AACTACTCCAAGCTGCGCCTGGCTGATCTCGGCCAAGTCCTCGACTACATCAACCTCATG
1058RW .         AACTACTCCAAGCTGCGCCTGGCTGATCTCGGCCAAGTCCTCGACTACATCAACCTCATG
590AUS .         AACTACTCCAAGCTGCGCCTGGCTGATCTCGGCCAAGTCCTCGACTACATCAACCTCATG
53SA .           AACTACTCCAAGCTGCGCCTGGCTGATCTCGGCCAAGTCCTCGACTACATCAACCTCATG
1075WIS .        AACTACTCCAAGCTGCGCCTGGCTGATCTCGGCCAAGTCCTCGACTACATCAACCTCATG
939GER .         AACTACTCCAAGCTGCGCCTGGCTGATCTCGGCCAAGTCCTCGACTACATCAACCTCATG
GA3804 .         AACTACTCCAAGCTGCGCCTGGCTGATCTCGGCCAAGTCCTCGACTACATCAACCTCATG
1599AT .         AACTACTCCAAGCTGCGCCTGGCTGATCTTGGCCAAGTCCTCGACTACATCAACCTCATG
1941AT .         AACTACTCCAAGCTGCGCCTGGCTGATCTTGGCCAAGTCCTTGACTACATCAACCTCATG
291NE .          AACTACTCCAAGCTGCGCCTGGCTGATCTTGGCCAAGTCCTTGACTACATCAACCTCATG
2301SAR .        AACTACTCCAAGCTGCGCCTGGCTGATCTTGGCCAAGTCCTCGACTACATCAACCTCATG
2313SAR .        AACTACTCCAAGCTGCGCCTGGCTGATCTTGGCCAAGTCCTTGACTACATCAACCTCATG
271NE .          AACTACTCCAAGCTGCGCCTGGCTGATCTTGGCCAAGTCCTTGACTACATCAACCTCATG
1935GER .        AACTACTCCAAGCTGCGCCTGGCTGATCTTGGCCAAGTCCTCGACTACATCAACCTCATG
JBRSA122 .       AACTACTCCAAGCTGCGCCTGGCTGATCTTGGCCAAGTCCTCGACTACATCAACCTCATG
276NE .          AACTACTCCAAGCTGCGCCTGGCTGATCTCGGCCAAGTCCTCGACTACATCAACCTCATG
272NE .          AACTACTCCAAGCTGCGCCTGGCTGATCTCGGCCAAGTCCTCGACTACATCAACCTCATG
2673ETHI .       AACTACTCCAAGCTGCGCCTGGCTGATCTTGGTCAAGTCCTCGACTACATCAACCTCATG
1084AUSW .       AACTACTCCAAGCTGCGCCTGGCTGATCTTGGTCAAGTCCTCGACTACATCAACCTCATG
JBPER62 .        AACTACTCCAAGCTGCGCCTGGCTGATCTTGGCCAAGTCCTCGACTACATCAACCTCATG
JBNZ72 .         AACTACTCCAAGCTGCGCCTGGCTGATCTTGGCCAAGTCCTTGACTACATCAACCTCATG
1065MEX .        AACTACTCCAAGCTGCGTCTGGCTGATCTCGGCCAAGTCCTCGACTATATCAACCTCATG
646HAWAI .       AACTACTCCAAGCTGCGCCTGGCTGATCTCGGCCAAGTCCTCGACTATATCAACCTCATG
727INDIA .       AACTACTCCAAGCTGCGCCTGGCTGATCTCGGCCAAGTCCTCGACTATATCAACCTCATG
743MALE .        AACTACTCCAAGCTGCGCCTGGCTGATCTCGGCCAAGTCCTCGACTATATCAACCTCATG
1059MEX .        AACTACTCCAAGCTGCGCCTGGCTGATCTCGGCCAAGTCCTCGACTATATCAACCTCATG
1061RW .         AACTACTCCAAGCTGCGCCTGGCCGATCTTGGCCAAGTTCTCGACTACATCAACCTCATG
1066MEX .        AACTACTCCAAGCTGCGCCTGGCTGATCTTGGCCAAGTCCTCGACTACATCAACCTCATG
1095BALI .       AACTACTCCAAGCTGCGCCTGGCCGATCTTGGCCAAGTCCTCGACTACATCAACCTCATG
1505MAUR .       AACTACTCCAAGCTGCGCCTGGCTGATCTCGGCCAAGTCCTCGACTACATCAACCTCATG
1818ETHI .       AACTACTCCAAGCTGCGCCTGGCTGATCTCGGCCAAGTCCTCGACTACATCAACCTCATG
1934AT .         AACTACTCCAAGCTGCGCCTGGCTGATCTCGGCCAAGTCCTCGACTACATCAACCTCATG
245CR .          AACTACTCCAAGCTGCGTCTGGCCGATCTTGGCCAAGTCCTTGACTACATTAACCTCATG
2710ETHI .       AACTACTCCAAGCTGCGTCTGGCCGATCTTGGCCAAGTCCTTGACTACATTAACCTCATG
274NE .          AACTACTCCAAGCTGCGTCTGGCCGATCTTGGCCAAGTCCTTGACTACATTAACCTCATG
3408PNG .        AACTACTCCAAGCTGCGCCTGGCTGATCTCGGCCAAGTCCTCGACTACATCAACCTCATG
3409PNG .        AACTACTCCAAGCTGCGCCTGGCTGATCTCGGCCAAGTCCTCGACTACATCAACCTCATG
410MALAY .       AACTACTCTAAGCTGCGCCTGGCTGATCTCGGCCAAGTCCTCGACTACATTAACCTGATG
836EGY .         AACTACTCCAAGCTGCGCCTGGCTGATCTCGGCCAAGTCCTCGACTACATCAACCTCATG
837EGY .         AATTACTCCAAGCTGCGCCTGGCTGATCTCGGCCAAGTCCTCGACTACATCAACCTCATG
JBRO111 .        AACTACTCCAAGCTGCGCCTGGCTGATCTCGGCCAAGTCCTCGACTACATCAACCTCATG
brunneoviridis4  AACTACTCCAAGCTGCGCCTGGCCGATCTTGGCCAAGTCCTCGACTATATTAACCTCATG
brunneoviridis1  AACTACTCCAAGCTGCGCCTGGCCGATCTTGGCCAAGTCCTCGACTATATTAACCTCATG
pleurotumCPK211  AACTACTCCAAGCTGCGCCTGGCTGATCTTGGCCAAGTCCTCGACTATATTAACCTCATG
PLEUROTICOLA .   AACTACTCCAAGCTGCSCCTGGCTGATCTTGGCCAAGTCCTCRACTATATTAACCTCATG
epimycesCPK1981  AACTACTCCAAGCTGCGCCTGGCCGATCTTGGCCAGGTCCTCGACTACATCAACCTCATG
alniCPK3124 ...  AACTACTCCAAGCTACGCCTGGCTGATCTTGGCCAAGTCCTTGACTACATTAACCTCATG
CPK5TAWA .       AACTACTCCAAGCTGCGCCTGGCTGATCTTGGCCAAGTCCTTGACTACATTAACCTCATG
2974lobau .      AACTACTCCAAGCTGCGCCTGGCTGATCTTGGCCAAGTCCTTGACTACATCAACCTCATG
2975lobau .      AACTACTCCAAGCTGCGCCTGGCTGATCTTGGCCAAGTCCTTGACTACATCAACCTCATG
aggressivumCPK3  AACTACTCCAAGCTGCGCCTGGCCGATCTTGGCCAAGTCCTCGACTACATCAACCTCATG
atrogelatinosaC  AACTACTCCAAGCTGCGTCTGGCTGATCTTGGCCAAGTCCTCGACTACATTAACCTCATG
```

```
atrogelatinosaC   AACTACTCCAAGCTGCGTCTGGCTGATCTTGGCCAAGTCCTCGACTACATTAACCTCATG
cerinumCPK293 .   AACTACTCCAAGCTGCGCCTGGCTGATCTTGGCCAAGTCCTCGACTATATTAACCTCATG
cerinumCPK854 .   AACTACTCCAAGCTGCGCCTGGCTGATCTTGGCCAAGTCCTCGACTATATTAACCTCATG
tomentosumCPK88   AACTACTCCAAGCTGCGCCTGGCTGATCTTGGCCAAGTCCTCGACTATATTAACCTCATG
stramineaGJS028   AACTACTCCAAGCTGCGCCTGGCTGATCTTGGCCAAGTCCTCGACTATATTAACCTCATG
velutinumCPK312   AACTATTCCAAGCTGCGTCTGGCTGATCTTGGCCAAGTCCTCGACTATATCAACCTCATG
catoptronGJS027   AACTACTCCAAGCTGCGTCTGGCCGATCTTGGCCAAGTCCTCGATTATATCAACCTCATG
                  ############################################################


                        1030      1040      1050      1060      1070      1080
                  =========+=========+=========+=========+=========+=========+
1093CAN .         GCCTACGACTACGCTGGATCCTTCAGCCCCCTCACC-GGTCACGACGCCAACCTGTTTGC
1052SA .          GCCTACGACTACGCTGGATCCTTCAGCCCCCTCACC-GGTCACGACGCCAACCTGTTTGC
1116CAN .         GCCTACGACTACGCTGGATCCTTCAGCCCCCTCACC-GGTCACGACGCCAACCTGTTTGC
1070EIRE .        GCCTACGACTACGCTGGATCCTTCAGCCCCCTCACC-GGTCACGACGCCAACCTGTTTGC
1099CAN .         GCCTACGACTACGCTGGATCCTTCAGCCCCCTCACC-GGTCACGACGCCAACCTGTTTGC
1104CAN .         GCCTACGACTACGCTGGATCCTTCAGCCCCCTCACC-GGTCACGACGCCAACCTGTTTGC
1087CAN .         GCCTACGACTACGCTGGATCCTTCAGCCCCCTCACC-GGTCACGACGCCAACCTGTTTGC
360UK .           GCCTACGACTACGCTGGATCCTTCAGCCCCCTCACC-GGTCACGACGCCAACCTGTTTGC
2111HUN .         GCCTACGACTACGCTGGATCCTTCAGCCCCCTCACC-GGTCACGACGCCAACCTGTTTGC
217RUS .          GCCTACGACTACGCTGGATCCTTCAGCCCCCTCACC-GGTCACGACGCCAACCTGTTTGC
204UK .           GCCTACGACTACGCTGGATCCTTCAGCCCCCTCACC-GGTCACGACGCCAACCTGTTTGC
206RUS .          GCCTACGACTACGCTGGATCCTTCAGCCCCCTCACC-GGTCACGACGCCAACCTGTTTGC
261SIB .          GCCTACGACTACGCTGGATCCTTCAGCCCCCTCACC-GGTCACGACGCCAACCTGTTTGC
265SIB .          GCCTACGACTACGCTGGATCCTTCAGCCCCCTCACC-GGTCACGACGCCAACCTGTTTGC
2784THAI .        GCCTACGACTACGCCGGATCCTTCAGCCCCCTCACC-GGTCACGACGCCAACCTGTTTAA
838EGY .          GCCTACGACTACGCCGGATCCTTCAGCCCCCTCACC-GGTCACGATGCCAACCTGTTTAA
1102CAN .         GCCTACGACTACGCCGGATCCTTCAGCCCCCTCACC-GGTCACGACGCCAACCTGTTTAA
588LAOS .         GCCTACGACTACGCCGGATCCTTCAGCCCCCTCACC-GGTCACGACGCCAACCTGTTTAA
1596AT .          GCCTACGACTACGCCGGATCCTTCAGCCCCCTCACC-GGTCACGACGCCAACCTGTTTAA
1720CAM .         GCCTACGACTACGCCGGATCATTCAGCCCCCTCACC-GGTCACGACGCCAACCTGTTTAA
1722CAM .         GCCTACGACTACGCCGGATCCTTCAGCCCCCTCACC-GGTCACGACGCCAACCTGTTCAA
1724CAM .         GCCTACGACTACGCCGGATCATTCAGCCCCCTCACC-GGTCACGACGCCAACCTGTTTAA
334USA .          GCCTACGACTACGCCGGATCCTTCAGCCCCCTCACC-GGTCACGACGCCAACCTGTTTAA
335USA .          GCCTACGACTACGCCGGATCCTTCAGCCCCCTCACC-GGTCACGACGCCAACCTGTTTAA
1081WIS .         GCCTACGACTACGCCGGATCCTTCAGCCCCCTCACC-GGTCACGACGCCAACCTGTTTAA
1069WIS .         GCCTACGACTACGCCGGATCCTTCAGCCCCCTCACC-GGTCACGACGCCAACCTGTTTAA
1107CAN .         GCCTACGACTACGCCGGATCCTTCAGCCCCCTCACC-GGTCACGACGCCAACCTGTTTAA
1108PEN .         GCCTACGACTACGCCGGATCCTTCAGCCCCCTCACC-GGTCACGACGCCAACCTGTTTAA
1110CAN .         GCCTACGACTACGCCGGATCCTTCAGCCCCCTCACC-GGTCACGACGCCAACCTGTTTAA
JBSER241 .        GCCTACGACTATGCTGGATCCTTCAGCCCCCTCACC-GGTCACGACGCCAACCTGTTTGC
246PHI .          GCCTACGACTATGCTGGATCCTTCAGCCCCCTCACC-GGTCACGACGCCAACCTGTTTGC
51SA .            GCCTACGACTATGCTGGATCCTTCAGCCCCCTCACC-GGTCACGACGCCAACCTGTTTGC
2618ETHI .        GCCTACGACTATGCTGGATCCTTCAGCCCCCTCACC-GGTCACGACGCCAACCTGTTTGC
1068WIS .         GCCTACGACTACGCCGGATCCTTCAGCCCCCTCACC-GGTCACGACGCCAACCTGTTTAA
238CR .           GCCTACGACTATGCTGGATCCTTCAGCCCCCTCACC-GGTCACGACGCCAACCTGTTTGC
NR5555 .          GCCTACGACTATGCTGGATCCTTCAGCCCCCTCACC-GGTCACGACGCCAACCTGTTTGC
NR6839 .          GCCTACGACTACGCTGGATCCTTCAGCCCCCTCACC-GGTCACGACGCCAACCTGTTTGC
2624ETHI .        GCCTACGACTACGCTGGATCCTTCAGCCCCCTCACC-GGTCACGACGCCAACCTGTTTGC
845EGY .          GCCTACGACTACGCTGGATCCTTCAGCCCCCTCACC-GGTCACGACGCCAACCTGTTTGC
JBPER12 .         XXXXXXGATCACGCTGGATCCTTCAGCCCCCTCACC-GGTCACGACGCCAACCTGTTTGC
1717VIET .        GCCTACGACTACGCTGGATCCTTCAGCCCCCTCACC-GGTCACGACGCCAACCTGTTTGC
709BRAZ .         GCCTACGACTACGCTGGATCCTTCAGCCCCCTCACC-GGTCACGACGCCAACCTGTTTGC
878IRAN .         GCCTACGACTACGCTGGATCCTTCAGCCCCCTAACC-GGTCACGACGCCAACCTGTTTGC
239CR .           GCCTACGACTACGCTGGATCCTTCAGCCCCCTCACC-GGTCACGACGCCAACCTGTTTGC
202COM .          GCCTACGACTACGCTGGATCCTTCAGCCCTCTCACC-GGTCACGACGCCAACCTGTTTGC
1064MEX .         GCCTACGACTACGCTGGATCCTTCAGCCCCCTCACC-GGTCACGACGCCAACCTGTTTGC
596BRAZ .         GCCTACGACTACGCTGGATCCTTCAGCCCCCTCACC-GGTCACGACGCCAACCTGTTTGC
333USA .          GCCTACGACTACGCTGGATCCTTCAGCCCCCTCACC-GGTCACGACGCCAACCTGTTTGC
2610ETHI .        GCCTACGACTACGCTGGATCCTTCAGCCCCCTCACC-GGCCACGACGCCAACCTGTTTGC
1044RW .          GCCTACGACTACGCTGGATCCTTCAGCCCCCTCACC-GGCCACGACGCCAACCTGTTTGC
JBNZ111 .         GCCTACGACTACGCTGGATCCTTTAGCCCCCTCACC-GGCCACGACGCCAACCTGTTTGC
JBNZ12 .          GCCTACGACTACGCTGGATCCTTCAGCCCCCTCACC-GGCCACGACGCCAACCTGTTTGC
JBNZ24 .          GCCTACGACTACGCTGGATCCTTTAGCCCCCTCACC-GGCCACGACGCCAACCTGTTTGC
693CHINA .        GCCTACGACTACGCTGGATCCTTCAGCCCCCTCACT-GGTCACGACGCCAACCTGTTTGC
1058RW .          GCCTACGACTACGCTGGATCCTTCAGCCCCCTCACC-GGCCACGACGCCAACCTGTTTGC
590AUS .          GCCTACGACTACGCTGGATCCTTCAGCCCCCTCACC-GGTCACGACGCCAACCTGTTTGC
53SA .            GCCTACGACTACGCTGGATCCTTCAGCCCCCTCACC-GGCCACGACGCCAACCTGTTTGC
1075WIS .         GCCTACGACTACGCTGGATCCTTCAGCCCCCTCACC-GGTCACGACGCCAACCTGTTTGC
939GER .          GCCTACGACTACGCTGGATCCTTCAGCCCCCTCACC-GGTCACGACGCCAACCTGTTTGC
GA3804 .          GCCTACGACTACGCTGGATCCTTCAGCCCCCTCACC-GGTCACGACGCCAACCTGTTTGC
1599AT .          GCCTACGACTACGCTGGATCCTTCAGCCCCCTCACC-GGTCACGACGCCAACCTGTTTGC
1941AT .          GCCTACGACTACGCTGGATCTTTCAGCCCCCTCACC-GGCCACGACGCCAACCTGTTTGC
291NE .           GCCTACGACTACGCTGGATCCTTCAGCCCCCTCACC-GGTCACGACGCCAACCTGTTTGC
2301SAR .         GCCTACGACTACGCTGGATCCTTCAGCCCCCTCACC-GGTCACGACGCCAACCTGTTTGC
2313SAR .         GCCTACGACTACGCTGGATCTTTCAGCCCCCTCACC-GGCCACGACGCCAACCTGTTTGC
271NE .           GCCTACGACTACGCTGGATCTTTCAGCCCCCTCACC-GGCCACGACGCCAACCTGTTTGC
1935GER .         GCCTACGACTACGCTGGATCCTTCAGCCCCCTCACC-GGTCACGACGCCAACCTGTTTGC
JBRSA122 .        GCCTACGACTACGCTGGATCCTTCAGCCCCCTCACC-GGTCACGACGCCAACCTGTTTGC
276NE .           GCCTACGACTACGCTGGATCCTTCAGCCCCCTCACC-GGTCACGACGCCAACCTGTTTGC
272NE .           GCCTACGACTACGCTGGATCCTTCAGCCCCCTCACC-GGTCACGACGCCAACCTATTTGC
2673ETHI .        GCCTACGACTACGCTGGATCCTTCAGCCCCCTCACC-GGCCACGACGCCAACCTGTTTGC
1084AUSW .        GCCTACGACTACGCTGGATCCTTCAGCCCCCTCACC-GGTCACGACGCCAACCTGTTTGC
JBPER62 .         GCCTACGACTACGCTGGATCCTTCAGCCCCCTCACC-GGCCACGACGCCAACCTGTTTGC
JBNZ72 .          GCCTACGACTACGCTGGATCCTTCAGCCCCCTCACC-GGCCACGACGCCAACCTGTTTGC
1065MEX .         GCCTACGACTACGCTGGATCCTTCAGCCCCCTCACCCGGTCACGACGCCAACCTGTTTGC
646HAWAI .        GCCTACGACTACGCTGGATCCTTCAGCCCCCTCACC-GGTCACGACGCCAACCTGTTTGC
727INDIA .        GCCTACGACTACGCTGGATCCTTCAGCCCCCTCACC-GGTCACGACGCCAACCTGTTTGC
743MALE .         GCCTACGACTACGCTGGATCCTTCAGCCCCCTCACC-GGTCACGACGCCAACCTGTTTGC
1059MEX .         GCCTACGACTACGCTGGATCCTTCAGCCCCCTCACC-GGTCACGACGCCAACCTGTTTGC
1061RW .          GCCTACGACTATGCTGGATCCTTCAGCCCCCTCACC-GGTCACGACGCCGACCTGTTTGC
1066MEX .         GCCTACGACTACGCTGGATCCTTCAGCCCCCTCACC-GGCCACGACGCCAACCTATTTGC
1095BALI .        GCCTACGACTATGCTGGATCCTTCAGCCCCCTCACC-GGTCACGACGCCAACCTGTTTGC
1505MAUR .        GCCTACGACTACGCTGGATCCTTCAGCCCCCTCACC-GGTCACGACGCCAACCTGTTTGC
1818ETHI .        GCCTACGACTACGCTGGATCCTTCAGCCCCCTCACC-GGTCACGACGCCAACCTGTTTGC
1934AT .          GCCTACGACTACGCTGGATCCTTCAGCCCCCTCACC-GGTCACGACGCCAACCTGTTTGC
245CR .           GCCTACGACTACGCTGGATCCTTCAGCCCCCTCACC-GGTCACGACGCCAACCTGTTTGC
2710ETHI .        GCCTACGACTACGCTGGATCCTTCAGCCCCCTCACC-GGTCACGACGCCAACCTGTTTGC
274NE .           GCCTACGACTACGCTGGATCCTTCAGCCCCCTCACC-GGTCACGACGCCAACCTGTTTGC
3408PNG .         GCCTACGACTACGCTGGATCCTTCAGCCCCCTCACC-GGTCACGACGCCAACCTGTTTGC
3409PNG .         GCCTACGACTACGCTGGATCCTTCAGCCCCCTCACC-GGTCACGACGCCAACCTGTTTGC
410MALAY .        GCCTACGACTACGCTGGATCCTTCAGCCCCCTCACT-GGACACGACGCCAATCTGTTTGC
836EGY .          GCCTACGACTACGCTGGATCCTTCAGCCCCCTCACC-GGTCACGACGCCAACCTGTTTGC
837EGY .          GCCTACGACTACGCTGGATCCTTCAGCCCCCTCACC-GGTCACGACGCCAACCTGTTTGC
JBRO111 .         GCCTACGACTACGCTGGATCCTTCAGCCCCCTCACC-GGTCACGACGCCAACCTGTTTGC
brunneoviridis4   GCCTACGACTACGCTGGATCCTTCAGCCCCCTCACC-GGCCACGACGCCAACCTGTTTGC
brunneoviridis1   GCCTACGACTACGCTGGATCCTTCAGCCCCCTCACC-GGCCACGACGCCAACCTGTTTGC
pleurotumCPK211   GCCTACGACTACGCTGGATCCTTTAGCCCCCTCACT-GGTCATGATGCCAACCTGTTTGC
PLEUROTICOLA .    GCCTACRACTACGCTGGATCCTTCAGCCCCCTCACT-GGCCACRACGCCAACCTGTTTGC
epimycesCPK1981   GCCTACGACTACGCTGGTTCCTTCAGCCCTCTCACC-GGCCACGACGCCAACCTGTTTGC
alniCPK3124 ...   GCCTACGACTACGCTGGATCCTTCAGCCCTCTCACC-GGTCACGACGCCAACCTGTTTGC
CPK5TAWA .        GCCTACRACTACGCTGGATCCTTCAGCCCCCTCACC-GGTCACRACGCCAACCTGTTTGC
2974lobau .       GCCTACGACTACGCTGGATCCTTCAGCCCCCTCACC-GGTCACGACGCCAACCTATTTGC
2975lobau .       GCCTACGACTACGCTGGATCCTTCAGCCCCCTCACC-GGTCACGACGCCAACCTATTTGC
aggressivumCPK3   GCCTACGACTACGCTGGTTCCTTCAGCCCCCTCACC-GGCCACGACGCCAACCTGTTTGC
atrogelatinosaC   GCCTACGACTACGCTGGATCTTTCAGCCCCCTCACT-GGTCACGACGCCAACCTGTTTGC
```

```
atrogelatinosaC   GCCTACGACTACGCTGGATCTTTCAGCCCCCTCACT-GGTCACGACGCCAACCTGTTTGC
cerinumCPK293 .   GCCTACGACTACGCTGGATCCTTCAGCCCCCTCACC-GGTCACGACGCCAATCTGTTTGC
cerinumCPK854 .   GCCTACGACTACGCTGGATCCTTCAGCCCCCTCACC-GGTCACGACGCCAATCTGTTTGC
tomentosumCPK88   GCCTACGACTACGCTGGATCCTTCAGCCCCCTCACC-GGTCACGATGCCAATCTGTTTGC
stramineaGJS028   GCCTACGACTACGCTGGATCCTTCAGCCCCCTCACC-GGCCACGACGCCAACTTGTTTGC
velutinumCPK312   GCCTACGACTACGCTGGATCCTTCAGCCCCCTTACC-GGCCACGACGCCAACCTGTTTGC
catoptronGJS027   GCTTACGACTACGCTGGATCCTTCAGCCCTCTCACT-GGACACGACGCCAATCTGTTTGC
                  #################################### #######################


                        1090      1100      1110      1120      1130      1140
                  =========+=========+=========+=========+=========+=========+
1093CAN .         CAACCCGTCCAACCCCAATGCCACACCCTTCAACACCGATTCTGCTGTCAAGGATTATAT
1052SA .          CAACCCGTCCAACCCCAATGCCACACCCTTCAACACCGATTCTGCTGTCAAGGATTATAT
1116CAN .         CAACCCGTCCAACCCCAATGCCACACCCTTCAACACCGATTCTGCTGTCAAGGATTATAT
1070EIRE .        CAACCCGTCCAACCCCAATGCCACACCCTTCAACACCGATTCTGCTGTCAAGGATTATAT
1099CAN .         CAACCCGTCCAACCCCAATGCCACACCCTTCAACACCGATTCTGCTGTCAAGGATTATAT
1104CAN .         CAACCCGTCCAACCCCAATGCCACACCCTTCAACACCGATTCTGCTGTCAAGGATTATAT
1087CAN .         CAACCCGTCCAACCCCAATGCCACACCCTTCAACACCGATTCTGCTGTCAAGGATTATAT
360UK .           CAACCCGTCCAACCCCAATGCCACACCCTTCAACACCGATTCTGCTGTCAAGGATTATAT
2111HUN .         CAACCCGTCCAACCCCAATGCCACACCCTTCAACACCGATTCTGCTGTCAAGGATTATAT
217RUS .          CAACCCGTCCAACCCCAATGCCACACCCTTCAACACCGATTCTGCTGTCAAGGATTATAT
204UK .           CAACCCGTCCAACCCCAATGCCACACCCTTCAACACCGATTCTGCTGTCAAGGATTATAT
206RUS .          CAACCCGTCCAACCCCAATGCCACACCCTTCAACACCGATTCTGCTGTCAAGGATTATAT
261SIB .          CAACCCGTCCAACCCCAATGCCACCCCCTTAAACACCGATTCTGCTGTCAAGGATTATAT
265SIB .          CAACCCGTCCAACCCCAATGCCACACCCTTCAACACCGATTCTGCTGTCAAGGATTATAT
2784THAI .        CAACCCGTCCAACCCCAATGCCACCCCCTTCAACACCGATTCCGCTGTCAAGGATTATAT
838EGY .          CAACCCGTCCAACCCCAATGCCACCCCCTTCAACACCGATTCCGCTGTCAAGGATTATAT
1102CAN .         CAACCCGTCCAACCCCAATGCCACCCCCTTCAACACCGATTCCGCTGTCAAGGATTATAT
588LAOS .         CAACCCGTCCAACCCCAATGCCACCCCCTTCAACACCGATCCCCCTGTCAAGGATTATAT
1596AT .          CAACCCGTCCAACCCCAATGCCACCCCCTTCAACACCGATTCCGCTGTCAAGGATTATAT
1720CAM .         CAACCCGTCCAACCCCAATGCCACCCCCTTCAACACCGATTCCGCTGTCAAGGATTATAT
1722CAM .         CAACCCGTCCAACCCCAATGCCACCCCCTTCAACACCGATTCCGCTGTCAAGGATTATAT
1724CAM .         CAACCCGTCCAACCCCAATGCCACCCCCTTCAACACCGATTCCGCTGTCAAGGATTATAT
334USA .          CAACCCGTCCAACCCCAATGCCACCCCCTTCAACACCGATTCCGCTGTCAAGGATTATAT
335USA .          CAACCCGTCCAACCCCAATGCCACCCCCTTCAACACCGATTCCGCTGTCAAGGATTATAT
1081WIS .         CAACCCGTCCAACCCCAATGCCACCCCCTTCAACACCGATTCCGCTGTCAAGGATTATAT
1069WIS .         CAACCCGTCCAACCCCAATGCCACCCCCTTCAACACCGATTCCGCTGTCAAGGATTATAT
1107CAN .         CAACCCGTCCAACCCCAATGCCACCCCCTTCAACACCGATTCCGCTGTCAAGGATTATAT
1108PEN .         CAACCCGTCCAACCCCAATGCCACCCCCTTCAACACCGATTCCGCTGTCAAGGATTATAT
1110CAN .         CAACCCGTCCAACCCCAATGCCACCCCCTTCAACACCGATTCCGCTGTCAAGGATTATAT
JBSER241 .        CAACCCGTCCAACCCCAACGCCACACCCTTCAACACCGATTCTGCCGTTCAGGATTATAT
246PHI .          CAACCCGTCCAACCCCAACGCCACACCCTTCAACACCGATTCTGCCGTTCAGGATTATAT
51SA .            CAACCCGTCCAACCCCAACGCCACACCCTTCAACACCGATTCTGCCGTTCAGGATTATAT
2618ETHI .        CAACCCGTCCAACCCCAACGCCACACCCTTCAACACCGATTCTGCCGTTCAGGATTATAT
1068WIS .         CAACCCGTCCAACCCCAATGCCACCCCCTTCAACACCGATTCCGCTGTCAAGGATTATAT
238CR .           CAACCCGTCCAACCCCAACGCCACACCCTTCAACACCGATTCTGCCGTTCAGGATTATAT
NR5555 .          CAACCCGTCCAACCCCAACGCCACACCCTTCAACACCGATTCTGCCGTTCAGGATTATAT
NR6839 .          AAACCCGTCCAACCCCAACGCCACACCCTTCAACACCGATTCTGCCGTTCAGGATTATAT
2624ETHI .        CAACCCGTCCAACCCCAACGCCACACCCTTCAACACCGATTCTGCCGTTCAGGATTATAT
845EGY .          CAACCCGTCCAACCCCAACGCCACACCCTTCAACACCGATTCTGCCGTTCAGGATTATAT
JBPER12 .         CAACCCGTCCAACCCCAACGCCACACCCTTCAACACCGATTCTGCCGTCAAGGATTATAT
1717VIET .        CAACCCGTCCAACCCCAACGCCACACCCTTCAACACCGATTCTGCCGTCAAGGATTATAT
709BRAZ .         CAACCCGTCCAACCCCAACGCCACACCCTTCAACACCGATTCTGCCGTCAAGGATTATAT
878IRAN .         CAACCCGTCCAACCCCAACGCCACACCCTTAAACACCGATTCTGCCGTCAAGGATTATAT
239CR .           CAACCCGTCCAACCCCAACGCCACACCCTTCAACACCGATTCTGCCGTCAAGGATTATAT
202COM .          CAACCCGTCCAACCCCAACGCCACACCCTTCAACACCGATTCTGCCGTCAAGGATTATAT
1064MEX .         CAACCCGTCCAACCCCAACGCCACACCCTTCAACACCGATTCTGCCGTCAAGGATTATAT
596BRAZ .         CAACCCGTCCAACCCCAACGCCACACCCTTCAACACCGATTCTGCCGTCAAGGATTATAT
333USA .          CAACCCGTCCAACCCCAACGCCACACCCTTCAACACCGATTCTGCCGTCAAGGATTATAT
2610ETHI .        CAACCCGTCCAACCCCAATGCCACACCCTTCAACACCGATTCTGCTGTCCAGGATTATAT
1044RW .          CAACCCGTCGAACCCCAACGCCACCCCCTTCAACACCGATTCCGCTGTCAAGGATTATAT
JBNZ111 .         CAACCCGTCCAACCCCAATGCCACCCCCTTCAACACCGATTCCGCTGTCAAGGATTATAT
JBNZ12 .          CAACCCGTCCAACCCCAATGCCACCCCCTTCAACACCGATTCCGCTGTCAAGGATTATAT
JBNZ24 .          CAACCCGTCCAACCCCAATGCCACCCCCTTCAACACCGATTCCGCTGTCAAGGATTATAT
693CHINA .        CAACCCGTCCAACCCTAATGCCACCCCCTTCAACACCGATTCTGCTGTCAAGGATTATAT
1058RW .          CAACCCGTCGAACCCCAACGCCACCCCCTTCAACACCGATTCCGCTGTCAAGGATTATAT
590AUS .          CAACCCATCCAACCCCAATGCCACCCCCTTCAACACCGATTCCGCTGTCAAGGATTACAT
53SA .            CAACCCGTCCAACCCCAATGCCACACCCTTCAACACCGATTCTGCTGTCCAGGATTATAT
1075WIS .         CAACCCGTCCAACCCCAATGCCACACCCTTCAACACCGATTCCGCTGTCAAGGATTATAT
939GER .          CAACCCGTCCAACCCCAATGCCACACCCTTCAACACCGATTCCGCTGTCAAGGATTATAT
GA3804 .          CAACCCGTCCAACCCCAATGCCACACCCTTCAACACCGATTCCGCTGTCAAGGATTATAT
1599AT .          CAACCCGTCCAACCCCAATGCCACACCCTTCAACACCGATTCCGCTGTCAAGGATTATAT
1941AT .          CAACCCGTCCAACCCCAATGCCACCCCCTTCAACACCGATTCCGCTGTCAAGGATTATAT
291NE .           CAACCCGTCCAACCCCAACGCCACACCCTTCAACACCGATTCTGCTGTCAAGGATTATAT
2301SAR .         CAACCCGTCCAACCCCAATGCCACACCCTTCAACACCGATTCCGCTGTCAAGGATTATAT
2313SAR .         CAACCCGTCCAACCCCAATGCCACCCCCTTCAACACCGATTCCGCTGTCAAGGATTATAT
271NE .           CAACCCGTCCAACCCCAATGCCACCCCCTTCAACACCGATTCCGCTGTCAAGGATTATAT
1935GER .         CAACCCGTCCAACCCCAATGCCACACCCTTCAACACCGATTCCGCTGTCAAGGATTATAT
JBRSA122 .        CAACCCGTCCAACCCCAATGCCACACCCTTCAACACCGATTCCGCTGTCAAGGATTATAT
276NE .           CAACCCGTCCAACCCCAATGCCACACCCTTCAACACCGATTCCGCTGTCAAGGATTATAT
272NE .           CAACCCGTCCAACCCCAATGCCACACCCTTCAACACCGATTCCGCTGTCAAGGATTATAT
2673ETHI .        CAACCCGTCGAACCCCAATGCCACCCCCTTCAACACCGATTCCGCTGTCAAGGATTATAT
1084AUSW .        CAACCCGTCCAACCCCAACGCCACACCCTTCAACACCGATTCTGCCGTCAAGGATTATAT
JBPER62 .         CAACCCGTCGAACCCCAATGCCACCCCCTTCAACACCGATTCCGCTGTCAAGGATTATAT
JBNZ72 .          CAACCCGTCCAACCCCAATGCCACCCCCTTCAACACCGATTCCGCTGTCAAGGATTATAT
1065MEX .         CAACCCGTCCAACCCCAATGCCACACCCTTCAACACCGATTCTGCTGTCAAGGATTATAT
646HAWAI .        CAACCCGTCCAACCCCAATGCCACACCCTTCAACACCGATTCTGCTGTCAAGGATTATAT
727INDIA .        CAACCCGTCCAACCCCAATGCCACACCCTTCAACACCGATTCTGCTGTCAAGGATTATAT
743MALE .         CAACCCGTCCAACCCCAATGCCACACCCTTCAACACCGATTCTGCTGTCAAGGATTATAT
1059MEX .         CAACCCGTCCAACCCCAATGCCACACCCTTCAACACCGATTCTGCTGTCAAGGATTATAT
1061RW .          CAACCCGTCCAACCCCAACGCCACACCCTTCAACACCGATTCTGCCGTTCAGGATTATAT
1066MEX .         CAACCCGTCCAACCCCAATGCCACACCCTTCAACACCGATTCTGCTGTCCAGGATTATAT
1095BALI .        CAACCCGTCCAACCCCAACGCCACACCCTTCAACACCGATTCTGCCGTTCAGGATTATAT
1505MAUR .        CAACCCGTCCAACCCCAATGCCACACCCTTCAACACCGATTCTGCCGTCAAGGATTATAT
1818ETHI .        CAACCCGTCCAACCCCAATGCCACACCCTTCAACACCGATTCTGCTGTCAAGGATTATAT
1934AT .          CAACCCGTCCAACCCCAATGCCACACCCTTCAACACCGATTCCGCTGTCAAGGATTATAT
245CR .           CAACCCGTCCAACCCCAACGCCACACCCTTCAACACCGATTCTGCCGTCAAGGATTATAT
2710ETHI .        CAACCCGTCCAACCCCAACGCCACACCCTTCAACACCGATTCTGCCGTCAAGGATTATAT
274NE .           CAACCCGTCCAACCCCAACGCCACACCCTTCAACACCGATTCTGCCGTCAAGGATTATAT
3408PNG .         CAACCCGTCCAACCCCAATGCCACCCCCTTCAACACCGATCCTGCTGTCAAGGATTATAT
3409PNG .         CAACCCGTCCAACCCCAATGCCACCCCCTTCAACACCGATTCTGCTGTCAAGGATTATAT
410MALAY .        CAACCCGTCTAACCCCAATGCCACACCCTTCAACACCGATTCCGCTGTCAAGGATTATAT
836EGY .          CAACCCGTCCAACCCCAATGCCACACCCTTCAACACCGATTCTGCTGTCAAGGATTATAT
837EGY .          CAACCCGTCCAACCCCAATGCCACACCCTTCAACACCGATTCTGCCGTCAAGGATTATAT
JBRO111 .         CAACCCGTCCAACCCCAATGCCACACCCTTCAACACCGATTCTGCTGTCAAGGATTATAT
brunneoviridis4   CAACCCGTCCAACCCCAATGCCACACCCTTCAACACCGACACTGCGGTCAAGGATTATAT
brunneoviridis1   CAACCCGTCCAACCCCAATGCCACACCCTTCAACACCGACACTGCGGTCAAGGATTATAT
pleurotumCPK211   CAACCCGTCCAACCCCAATGCCACACCCTTCAACACCGACACTGCTGTCAAGGATTATAT
PLEUROTICOLA .    CAACCCGTCCAACCCCAATGCCACACCCTTCAACACCGACACTGcTGTCAAGGATTATAT
epimycesCPK1981   CAACCCGTCCAACCCCAATGCCACACCCTTCAACACCGATTCTGCTGTCAGGGATTATAT
alniCPK3124 ...   CAACCCGTCCAACCCCAACGCCACACCCTTCAACACCGATTCTGCTGTCAGGGATTATAT
CPK5TAWA .        CAACCCGTCCAACCCCAACGCCACACCCTTMAACACCGATTCTGCTGTCAGGGATTATAT
2974lobau .       CAACCCGTCCAACCCCAATGCCACCCCCTTCAACACCGATTCTGCTGTCAAGGATTATAT
2975lobau .       CAACCCGTCCAACCCCAATGCCACCCCCTTCAACACCGATTCTGCTGTCAAGGATTATAT
aggressivumCPK3   CAACCCGTCCAACCCCAATGCCACCCCCTTCAACACCGATTCTGCTGTCAGGGATTATAT
atrogelatinosaC   CAACCCGTCCAACCCCAATGCCACACCCTTCAACACCGACACTGCTGTCAAGGATTATAT
```

```
atrogelatinosaC  CAACCCGTCCAACCCCAATGCCACACCCTTCAACACCGACACTGCTGTCAAGGATTATAT
cerinumCPK293 .  CAACCCGTCCAACCCCAATGCCACACCCTTCAACACCGACTCTGCTGTCAAGGATTACAT
cerinumCPK854 .  CAACCCGTCCAACCCCAATGCCACACCCTTCAACACCGATTCTGCTGTCAAGGATTACAT
tomentosumCPK88  CAACCCCTCCAACCCCAATGCCACACCCTTCAACACCGATTCTGCTGTCAAGGATTACAT
stramineaGJS028  CAACCCCTCCAACCCCAATGCCACACCCTTCAACACCGATTCGGCTGTTAAGGATTATAT
velutinumCPK312  CAACCCGTCCAACCCCAATGCCACACCCTTCAACACCGATTCGGCTGTTAAGGATTATAT
catoptronGJS027  CAACCCCTCCAACCCCAATGCCACGCCCTTCAACACCGATACTGCTGTCAAGGATTACAT
                 ############################################################


                       1150      1160      1170      1180      1190      1200
                 =========+=========+=========+=========+=========+=========+
1093CAN .        CAATGGAGGTGTTCCCGCCAACAAGATTGTTCTCGGCATGCCCATCTACGGACGATCATT
1052SA .         CAATGGAGGTGTTCCCGCCAACAAGATTGTTCTCGGCATGCCCATCTACGGACGATCATT
1116CAN .        CAATGGAGGTGTTCCCGCCAACAAGATTGTTCTCGGCATGCCCATCTACGGACGATCATT
1070EIRE .       CAATGGAGGTGTTCCCGCCAACAAGATTGTTCTCGGCATGCCCATCTACGGACGATCATT
1099CAN .        CAATGGAGGTGTTCCCGCCAACAAGATTGTTCTCGGCATGCCCATCTACGGACGATCATT
1104CAN .        CAATGGAGGTGTTCCCGCCAACAAGATTGTTCTCGGCATGCCCATCTACGGACGATCATT
1087CAN .        CAATGGAGGTGTTCCCGCCAACAAGATTGTTCTCGGCATGCCCATCTACGGACGATCATT
360UK .          CAATGGAGGTGTTCCCGCCAACAAGATTGTTCTCGGCATGCCCATCTACGGACGATCATT
2111HUN .        CAATGGAGGTGTTCCCGCCAACAAGATTGTTCTCGGCATGCCCATCTACGGACGATCATT
217RUS .         CAATGGAGGTGTTCCCGCCAACAAGATTGTTCTCGGCATGCCCATCTACGGACGATCATT
204UK .          CAATGGAGGTGTTCCCGCCAACAAGATTGTTCTCGGCATGCCCATCTACGGACGATCATT
206RUS .         CAATGGAGGTGTTCCCGCCAACAAGATTGTTCTCGGCATGCCCATCTACGGACGATCATT
261SIB .         CAATGGAGGTGTTCCCGCCAACAAGATTGTTCTCGGCATGCCCATCXXXXXXXXXXXXXX
265SIB .         CAATGGAGGTGTTCCCGCCAACAAGATTGTTCTCGGCATGCCCATCTACGGACGATCATT
2784THAI .       CAATGGAGGTGTTCCCGCCAACAAGATTGTTCTCGGCATGCCCATTTACGGAAGATCATT
838EGY .         CAATGGAGGTGTTCCCGCCAACAAGATTGTTCTCGGCATGCCCATCTACGGAAGATCATT
1102CAN .        CAATGGAGGTGTTCCCGCCAACAAGATTGTTCTCGGCATGCCCATCTACGGAAGATCATT
588LAOS .        CAATGGAGGTGTTCCCGCCAACAAGATTGTTCTCGGCATGCCCATCTACGGAAGATCATT
1596AT .         CAATGGAGGTGTTCCCGCCAACAAGATTGTTCTCGGCATGCCCATCTACGGAAGATCATT
1720CAM .        CAATGGAGGTGTTCCCGCCAACAAGATTGTTCTCGGCATGCCCATCTACGGAAGATCATT
1722CAM .        CAATGGAGGTGTTCCCGCCAACAAGATTGTTCTCGGCATGCCCATCTACGGAAGATCATT
1724CAM .        CAATGGAGGTGTTCCCGCCAACAAGATTGTTCTCGGCATGCCCATCTACGGAAGATCATT
334USA .         CAATGGAGGTGTTCCCGCCAACAAGATTGTTCTCGGCATGCCCATCTACGGAAGATCATT
335USA .         CAATGGAGGTGTTCCCGCCAACAAGATTGTTCTCGGCATGCCCATCTACGGAAGATCATT
1081WIS .        CAATGGAGGTGTTCCCGCCAACAAGATTGTTCTCGGCATGCCCATCTACGGAAGATCATT
1069WIS .        CAATGGAGGTGTTCCCGCCAACAAGATTGTTCTCGGCATGCCCATCTACGGAAGATCATT
1107CAN .        CAATGGAGGTGTTCCCGCCAACAAGATTGTTCTCGGCATGCCCATCTACGGAAGATCATT
1108PEN .        CAATGGAGGTGTTCCCGCCAACAAGATTGTTCTCGGCATGCCCATCTACGGAAGATCATT
1110CAN .        CAATGGAGGTGTTCCCGCCAACAAGATTGTTCTCGGCATGCCCATCTACGGAAGATCATT
JBSER241 .       CAAGGGAGGTGTTCCCGCCAACAAGATTGTTCTCGGAATGCCCATCTACGGACGATCATT
246PHI .         CAAGGGAGGTGTTCCCGCCAACAAGATTGTTCTCGGAATGCCCATCTACGGACGATCATT
51SA .           CAAGGGAGGTGTTCCCGCCAACAAGATTGTTCTCGGAATGCCCATCTACGGACGATCATT
2618ETHI .       CAAGGGAGGTGTTCCCGCCAACAAGATTGTTCTCGGAATGCCCATCTACGGACGATCATT
1068WIS .        CAATGGAGGTGTTCCCGCCAACAAGATTGTTCTCGGCATGCCCATCTACGGAAGATCATT
238CR .          CAAGGGAGGTGTTCCCGCCAACAAGATTGTTCTCGGAATGCCCATCTACGGACGATCATT
NR5555 .         CAAGGGAGGTGTTCCCGCCAACAAGATTGTTCTCGGAATGCCCATCTACGGACGATCATT
NR6839 .         CAAGGGAGGTGTTCCCGCCAACAAGATTGTTCTCGGCATGCCCATCTACGGACGATCATT
2624ETHI .       CAAGGGAGGTGTTCCCGCCAACAAGATTGTTCTCGGCATGCCCATCTACGGACGATCATT
845EGY .         CAAGGGAGGTGTTCCCGCCAACAAGATTGTTCTCGGCATGCCCATCTACGGACGATCATT
JBPER12 .        CAAGGGAGGTGTTCCCGCCAACAAGATTGTTCTCGGCATGCCCATCTACGGACGATCATT
1717VIET .       CAAGGGAGGTGTTCCCGCCAACAAGATTGTTCTCGGCATGCCCATCTACGGACGATCATT
709BRAZ .        CAAGGGAGGTGTTCCCGCCAACAAGATTGTTCTCGGCATGCCCATCTACGGACGATCATT
878IRAN .        CAAGGGAGGTGTTCCCGCTAACAAGATTGTTCTCGGCATGCCCATCTACGGACGATCATT
239CR .          CAAGGGAGGTGTTCCCGCCAACAAGATTGTTCTCGGCATGCCCATCTACGGACGATCATT
202COM .         CAAGGGAGGTGTTCCCGCCAACAAGATTGTTCTCGGCATGCCCATCTACGGACGATCATT
1064MEX .        CAAGGGAGGTGTTCCCGCCAACAAGATTGTTCTCGGCATGCCCATCTACGGACGATCATT
596BRAZ .        CAAGGGAGGTGTTCCCGCTAACAAGATTGTTCTCGGCATGCCCATCTACGGACGATCATT
333USA .         CAAGGGAGGTGTTCCCGCCAACAAGATTGTTCTCGGCATGCCCATCTACGGACGATCATT
2610ETHI .       CAAGGGAGGTGTTCCCGCCAACAAGATTGTTCTCGGCATGCCCATCTACGGACGATCATT
1044RW .         CAAGGGAGGTGTTCCCGCCAACAAGATTGTTCTCGGCATGCCCATCTACGGACGATCATT
JBNZ111 .        CAAGGGAGGTGTTCCCGCCAACAAGATTGTTCTCGGCATGCCCATCTACGGACGATCATT
JBNZ12 .         CAAGGGAGGTGTTCCCGCCAACAAGATTGTTCTCGGCATGCCCATCTACGGACGATCATT
JBNZ24 .         CAAGGGAGGTGTTCCCGCCAACAAGATTGTTCTCGGCATGCCCATCTACGGACGATCATT
693CHINA .       CAAGGGAGGTGTTCCCGCCAACAAGATTGTCCTCGGCATGCCCATCTACGGACGATCATT
1058RW .         CAAGGGAGGTGTTCCCGCCAACAAGATTGTTCTCGGCATGCCCATCTACGGACGATCATT
590AUS .         CAAGGGAGGTGTTCCCGCCAACAAGATTGTTCTCGGCATGCCCATCTACGGACGATCATT
53SA .           CAAGGGAGGTGTTCCCGCCAACAAGATTGTTCTCGGCATGCCCATCTACGGACGATCATT
1075WIS .        CAAGGGAGGTGTTCCCGCCAACAAGATTGTTCTCGGCATGCCCATCTACGGACGATCATT
939GER .         CAAGGGAGGTGTTCCCGCCAACAAGATTGTTCTCGGCATGCCCATCTACGGACGATCATT
GA3804 .         CAAGGGAGGTGTTCCCGCCAACAAGATTGTTCTCGGCATGCCCATCTACGGACGATCATT
1599AT .         CAAGGGAGGTGTTCCCGCCAACAAGATTGTTCTCGGCATGCCCATCTACGGACGATCATT
1941AT .         CAAGGGAGGTGTTCCCGCCAACAAGATTGTTCTCGGCATGCCCATCTACGGACGATCATT
291NE .          CAAGGGAGGTGTTCCCGCCAACAAGATTGTTCTCGGCATGCCCATCTACGGACGATCCTT
2301SAR .        CAAGGGAGGTGTTCCCGCCAACAAGATTGTTCTCGGCATGCCCATCTACGGACGATCATT
2313SAR .        CAAGGGAGGTGTTCCCGCCAACAAGATTGTTCTCGGCATGCCCATCTACGGACGATCATT
271NE .          CAAGGGAGGTGTTCCCGCCAACAAGATTGTTCTCGGCATGCCCATCTACGGACGATCATT
1935GER .        CAAGGGAGGTGTTCCCGCCAACAAGATTGTTCTCGGCATGCCCATCTACGGACGATCATT
JBRSA122 .       CAAGGGAGGTGTTCCCGCCAACAAGATTGTTCTCGGCATGCCCATCTACGGACGATCATT
276NE .          CAAGGGAGGTGTTCCCGCCAACAAGATTGTTCTCGGCATGCCCATCTACGGACGATCATT
272NE .          CAAGGGAGGTGTTCCCGCCAACAAGATTGTTCTCGGCATGCCCATCTACGGACGATCATT
2673ETHI .       CAAGGGAGGTGTTCCCGCCAACAAGATTGTTCTCGGCATGCCCATCTATGGACGATCATT
1084AUSW .       CAAGGGAGGTGTTCCCGCTAACAAGATTGTTCTCGGCATGCCCATCTACGGACGATCATT
JBPER62 .        CAAGGGAGGTGTTCCCGCCAACAAGATTGTTCTCGGCATGCCCATCTATGGACGATCATT
JBNZ72 .         CAAGGGAGGTGTTCCCGCCAACAAGATTGTTCTCGGCATGCCCATCTACGGACGATCATT
1065MEX .        CAAGGGAGGTGTTCCCGCCAACAAGATTGTTCTCGGCATGCCCATCTACGGACGATCATT
646HAWAI .       CAAGGGAGGTGTTCCCGCCAACAAGATTGTTCTCGGCATGCCCATCTACGGACGATCATT
727INDIA .       CAAGGGAGGTGTTCCCGCCAACAAGATTGTTCTCGGCATGCCCATCTACGGACGATCATT
743MALE .        CAAGGGAGGTGTTCCCGCCAACAAGATTGTTCTCGGCATGCCCATCTACGGACGATCATT
1059MEX .        CAAGGGAGGTGTTCCCGCCAACAAGATTGTTCTCGGCATGCCCATCTACGGACGATCATT
1061RW .         CAAGGGAGGTGTTCCCGCCAACAAGATTGTTCTCGGAATGCCCATCTACGGACGATCATT
1066MEX .        CAAGGGAGGTGTTCCCGCCAACAAGATTGTTCTCGGCATGCCCATCTACGGACGATCATT
1095BALI .       CAAGGGAGGTGTTCCCGCCAACAAGATTGTTCTCGGAATGCCCATCTACGGACGATCATT
1505MAUR .       CAAGGGAGGTGTTCCCGCCAACAAGATTGTTCTCGGCATGCCCATCTACGGACGATCATT
1818ETHI .       CAATGGAGGTGTTCCCGCCAACAAGATTGTTCTCGGCATGCCCATCTACGGACGATCATT
1934AT .         CAAGGGAGGTGTTCCCGCCAACAAGATTGTTCTCGGCATGCCCATCTACGGACGATCATT
245CR .          CAAGGGGGGTGTTCCCGCCAACAAGATTGTTCTCGGCATGCCCATCTACGGACGATCATT
2710ETHI .       CAAGGGGGGTGTTCCCGCCAACAAGATTGTTCTCGGCATGCCCATCTACGGACGATCATT
274NE .          CAAGGGGGGTGTTCCCGCCAACAAGATTGTTCTCGGCATGCCCATCTACGGACGATCATT
3408PNG .        CAAGGGAGGTGTTCCCGCCAACAAGATTGTTCTCGGCATGCCCATCTACGGACGATCATT
3409PNG .        CAAGGGAGGTGTTCCCGCCAACAAGATTGTTCTCGGCATGCCCATCTACGGACGATCATT
410MALAY .       CAAGGGAGGTGTCCCAGCAAACAAGATTGTTCTCGGCATGCCCATCTACGGACGATCATT
836EGY .         CAATGGAGGTGTTCCCGCCAACAAGATTGTTCTCGGCATGCCCATCTACGGACGATCATT
837EGY .         CAAGGGAGGTGTTCCCGCCAACAAGATTGTTCTCGGCATGCCCATCTACGGACGATCATT
JBRO111 .        CAATGGAGGTGTTCCCGCCAACAAGATTGTTCTCGGCATGCCCATCTACGGACGATCATT
brunneoviridis4  CAATGGAGGTGTTCCCGCAAACAAGATTGTTCTCGGCATGCCCATCTACGGACGATCATT
brunneoviridis1  CAATGGAGGTGTTCCCGCAAACAAGATTGTTCTCGGCATGCCCATCTACGGACGATCATT
pleurotumCPK211  CAATGGAGGTGTTCCAGCAAACAAGATTGTTCTCGGCATGCCCATCTACGGACGATCATT
PLEUROTICOLA .   CAATGGAGGTGTTCCCGCAAACAAGATTGTTCTCGGCATGCCCATCTACGGACGATCATT
epimycesCPK1981  CAACGGAGGTGTTCCCGCCAACAAGATTGTTCTCGGCATGCCCATCTACGGACGATCATT
alniCPK3124 ...  CAATGGAGGTGTTCCCGCCAACAAGATTATTCTCGGCATGCCCATCTACGGACGATCATT
CPK5TAWA .       CAATGGAGGTGTTCCCGCCAACAARATTGTTCTCGGCATGCCCATCTACGGACGATCATT
2974lobau .      CAAGGGAGGTGTTCCCGCCAACAAGATTGTTCTCGGCATGCCCATCTACGGACGATCATT
2975lobau .      CAAGGGAGGTGTTCCCGCCAACAAGATTGTTCTCGGCATGCCCATCTACGGACGATCATT
aggressivumCPK3  CAACGGAGGTGTTCCCGCCAACAAGATTGTTCTCGGCATGCCCATCTACGGACGATCATT
atrogelatinosaC  CAAGGGAGGTGTTCCCGCAAACAAGATTGTTCTCGGCATGCCCATCTACGGACGATCATT
```

```
atrogelatinosaC  CAAGGGAGGTGTTCCCGCAAACAAGATTGTTCTCGGCATGCCCATCTACGGACGATCATT
cerinumCPK293 .  CAAGGGAGGTGTTCCCGCAAACAAGATTGTTCTCGGTATGCCCATCTACGGACGATCATT
cerinumCPK854 .  CAAGGGAGGTGTTCCCGCAAACAAGATTGTTCTCGGTATGCCCATCTACGGACGATCATT
tomentosumCPK88  CAAGGGAGGTGTTCCCGCAAACAAGATTGTTCTCGGCATGCCCATCTACGGACGATCATT
stramineaGJS028  CAAGGGAGGTGTTCCCGCAAACAAGATTGTTCTCGGCATGCCCATCTACGGACGATCATT
velutinumCPK312  CAAGGGAGGTGTTCCCGCAAACAAGATTGTTCTCGGCATGCCCATCTACGGACGATCATT
catoptronGJS027  CAAGGGAGGTGTTCCCGCAAACAAGATTGTTCTCGGCATGCCCATCTACGGACGATCATT
                 ############################################################


                       1210      1220      1230      1240      1250      1260
                 =========+=========+=========+=========+=========+=========+
1093CAN .        CCAGAACACCGCTGGCATTGGCCAGACTTACAACGGAGTTGGAGGTGGCGGTGGTGGCTC
1052SA .         CCAGAACACCGCTGGCATTGGCCAGACTTACAACGGAGTTGGAGGTGGCGGTGGTGGCTC
1116CAN .        CCAGAACACCGCTGGCATTGGCCAGACTTACAACGGAGTTGGAGGTGGCGGTGGTGGCTC
1070EIRE .       CCAGAACACCGCTGGCATTGGCCAGACTTACAACGGAGTTGGAGGTGGCGGTGGTGGCTC
1099CAN .        CCAGAACACCGCTGGCATTGGCCAGACTTACAACGGAGTTGGAGGTGGCGGTGGTGGCTC
1104CAN .        CCAGAACACCGCTGGCATTGGCCAGACTTACAACGGAGTTGGAGGTGGCGGTGGTGGCTC
1087CAN .        CCAGAACACCGCTGGCATTGGCCAGACTTACAACGGAGTTGGAGGTGGCGGTGGTGGCTC
360UK .          CCAGAACACCGCTGGCATTGGCCACACTTACAACCGAGTTGGAGGTGGCXXXXXXXXXXX
2111HUN .        CCAGAACACCGCTGGCATTGGCCAGACTTACAACGGATTTGGAGGTGGCGGTGGTGGCTC
217RUS .         CCAGAACACCGCTGGCATTGGCCAGACTTACAACGGAGTTGGAGGTGGCGGTGGTGGCTC
204UK .          CCAGAACACCGCTGGCATTGGCCAGACTTACAACGGATTTGGAGGTGGCGGTGGTGGCTC
206RUS .         CCAGAACACXXXXXXXXXXXXXXXXXXXXXXXXXXXXXXXXXXXXXXXXXXXXXXXXXXX
261SIB .         XXXXXXXXXXXXXXXXXXXXXXXXXXXXXXXXXXXXXXXXXXXXXXXXXXXXXXXXXXXX
265SIB .         CCAGAACACCGCTGGCATTGGCCAGACTTACAACGGATTTGGAGGTGGCGGTGGTGGCTC
2784THAI .       CCAGAACACCGCTGGTATTGGCCAGACTTACAATGGTGTTGGAAGTGG------------
838EGY .         CCAGAACACCGCTGGTATTGGCCAGACTTACAATGGTGTTGGAAGTGG------------
1102CAN .        CCAGAACACCGCTGGTATTGGCCAGACTTACAATGGTGTTGGAAGTGG------------
588LAOS .        CCAGAACACCGCTGGTATTGGCCAGACTTACAATGGTGTTGGAAGTGG------------
1596AT .         CCAGAACACCGCTGGTATTGGCCAGACTTACAATGGTGTTGGAAGTGG------------
1720CAM .        CCAGAACACCGCTGGTATTGGCCAGACTTACAATGGTGTTGGAAGTGG------------
1722CAM .        CCAGAACACCGCTGGTATTGGCCAGACTTACAATGGTGTTGGGAGTGG------------
1724CAM .        CCAGAACACCGCTGGTATTGGCCAGACTTACAATGGTGTTGGAAGTGG------------
334USA .         CCAGAACACCGCTGGTATTGGCCAGACTTACAATGGTGTTGGAAGTGG------------
335USA .         CCAGAACACCGCTGGTATTGGCCAGACTTACAATGGTGTTGGAAGTGG------------
1081WIS .        CCAGAACACCGCTGGTATTGGCCAGACTTACAATGGTGTTGGAAGTGG------------
1069WIS .        CCAGAACACCGCTGGTATTGGCCAGACTTACAATGGTGTTGGAAGTGG------------
1107CAN .        CCAGAACACCGCTGGTATTGGCCAGACTTACAATGGTGTTGGAAGTGG------------
1108PEN .        CCAGAACACCGCTGGTATTGGCCAGACTTACAATGGTGTTGGAAGTGG------------
1110CAN .        CCAGAACACCGCTGGTATTGGCCAGACTTACAATGGTGTTGGAAGTGG------------
JBSER241 .       CCAGAACACCGCTGGTATTGGCCAGACTTACAACGGAGTTGGAGGTGGCGGTGGTGGCTC
246PHI .         CCAGAACACCGCTGGTATTGGCCAGACTTACAACGGAGTTGGAGGTGGCGGTGGTGGCTC
51SA .           CCAGAACACCGCTGGTATTGGCCAGACTTACAACGGAGTTGGAGGTGGCGGTGGTGGCTC
2618ETHI .       CCAGAACACCGCTGGTATTGGCCAGACTTACAACGGAGTTGGAGGTGGCGGTGGTGGCTC
1068WIS .        CCAGAACACCGCTGGTATTGGCCAGACTTACAATGGTGTTGGAAGTGG------------
238CR .          CCAGAACACCGCTGGTATTGGCCAGACTTACAACGGAGTTGGAGGTGGCGGTGGTGGCTC
NR5555 .         CCAGAACACXXXXXXXXXXXXXXXXXXXXXXXXXXXXXXXXXXXXXXXXXXXXXXXXXXX
NR6839 .         CCAGAACACXXXXXXXXXXXXXXXXXXXXXXXXXXXXXXXXXXXXXXXXXXXXXXXXXXX
2624ETHI .       CCAGAACACCGCTGGTATTGGCCAGACTTACAACGGAGTTGGAGGTGGCGGTGGTGGCTC
845EGY .         CCAGAACACCGCTGGTATTGGCCAGACTTACAACGGAGTTGGAGGTGGCGGTGGTGGCTC
JBPER12 .        CCAGAACACCGCTGGTATTGGCCAGACTTACAACGGA-TTGGAGGTGGCGGTGGCTTCTC
1717VIET .       CCAGAACACCGCTGGTATTGGCCAGACTTACAACGGAGTTGGAGGTGGCGGTGGTGGCTC
709BRAZ .        CCAGAACACCGCTGGTATTGGCCAGACTTACAACGGAGTTGGAGGTGGCGGTGGTGGCTC
878IRAN .        CCAGAACACCGCTGGTATTGGCCAAACTTACAACGGATTTGGAGGTGGCGGTGGTGGCTC
239CR .          CCAGAACACCGCTGGTATTGGCCAGACTTACAACGGAGTTGGAGGTGGCGGTGGTGGCTC
202COM .         CCAGAACACXXXXXXXXXXXXXXXXXXXXXXXXXXXXXXXXXXXXXXXXXXXXXXXXXXX
1064MEX .        CCAGAACACCGCTGGTATTGGCCAGACTTACAACGGAGTTGGAGGTGGCGGTGGTGGCTC
596BRAZ .        CCAGAACACCGCTGGTATTGGCCAGACTTACAACGGAGTTGGAGGTGGCGGTGGTGGCTC
333USA .         CCAGAACACCGCTGGTATTGGCCAGACTTACAACGGAGTTGGAGGTGGCGGTGGTGGCTC
2610ETHI .       CCAGAACACCGCTGGTATTGGCCAGACTTACAACGGAGTTGGAGGTGGCGGTGGTGGCTC
1044RW .         CCAGAACACCGCTGGCATTGGCCAGACTTACAACGGAGTTGGAGGTGGCGGTGGTGGCTC
JBNZ111 .        CCAGAACACTGCTGGCATTGGCCAGACTTATAACGGAGTTGGAGGTGGCGGTGGTGGCTC
JBNZ12 .         CCAGAACACCGCTGGCATTGGCCAGACTTACAACGGAGTTGGAGGTGGCGGTGGTGGCTC
JBNZ24 .         CCAGAACACTGCTGGCATTGGCCAGACTTACAACGGAGTTGGAGGTGGCGGTGGTGGCTC
693CHINA .       CCAGAACACCGCTGGCATTGGCCAGACTTACAACGGAGTTGGAGGTGGCGGTGGTGGCTC
1058RW .         CCAGAACACCGCTGGCATTGGCCAGACTTACAACGGAGTTGGAGGTGGCGGTGGTGGCTC
590AUS .         CCAGAACACCGCTGGTATTGGCCAGACTTACAACGGAGTTGGAGGTGGCGGTGGTGGCTC
53SA .           CCAGAACACCGCTGGTATTGGCCAGACTTACAACGGAGTTGGAGGTGGCGGTGGTGGCTC
1075WIS .        CCAGAACACCGCTGGTATTGGCCAGACTTACAACGGAGTTGGAGGTGGCGGTGGTGGCTC
939GER .         CCAGAACACCGCTGGTATTGGCCAGACTTACAACGGAGTTGGAGGTGGCGGTGGTGGCTC
GA3804 .         CCAGAACACCGCTGGTATTGGCCAGACTTACAACGGAGTTGGAGGTGGCGGTGGTGGCTC
1599AT .         CCAGAACACCGCTGGTATTGGCCAGACTTACAACGGAGTTGGAGGTGGCGGTGGTGGCTC
1941AT .         CCAGAACACCGCTGGCATTGGCCAGACTTACAACGGAGTTGGAGGTGGCGGTGGTGGCTC
291NE .          CCAGAACACCGCTGGTATTGGCCAGACCTACAACGGATTTGGAGGTGGCGGTGGTGGCTC
2301SAR .        CCAGAACACCGCTGGTATTGGCCAGACTTACAACGGAGTTGGAGGTGGCGGGGGTGGCTC
2313SAR .        CCAGAACACCGCTGGCATTGGCCAGACTTACAACGGAGTTGGAGGTGGCGGTGGTGGCTC
271NE .          CCAGAACACXXXXXXXXXXXXXXXXXXXXXXXXXXXXXXXXXXXXXXXXXXXXXXXXXXX
1935GER .        CCAGAACACCGCTGGTATTGGCCAGACTTACAACGGATTTGGAGGTGGCGGTGGTGGCTC
JBRSA122 .       CCAGAACACCGCTGGTATTGGCCAGACTTACAACGGAGTTGGAGGTGGCGGTGGTGGCTC
276NE .          CCAGAACACCGCTGGTATTGGCCAGACTTACAACGGAGTTGGAGGTGGCGGTGGTGGCTC
272NE .          CCAAAACACCGCTGGTATTGGCCAGACTTACAACGGAGTTGGAGGTGGCGGTGGTGGCTC
2673ETHI .       CCAGAACACCGCTGGCATTGGCCAGACTTACAACGGAGTTGGAGGTGGCGGTGGTGGCTC
1084AUSW .       CCAGAACACCGCTGGCATTGGCCAGACTTACAACGGAGTTGGAGGTGGCGGTGGTGGCTC
JBPER62 .        CCAGAACACCGCTGGCATTGGCCAGACTTACAACGGAGTTGGAGGTGGCGGTGGTGGCTC
JBNZ72 .         CCAGAACACCGCTGGCATTGGCCAGACTTACAACGGAGTTGGAGGTGGCGGTGGTGGCTC
1065MEX .        CCAGAACACTGCTGGTATTGGCCAGACTTACAACGGAGTTGGAGGTGGCGGTGGTGGCTC
646HAWAI .       CCAGAACACTGCTGGTATTGGCCAGACTTACAACGGAGTTGGAGGTGGCGGTGGTGGCTC
727INDIA .       CCAGAACACTGCTGGTATTGGCCAGACTTACAACGGAGTTGGAGGTGGCGGTGGTGGCTC
743MALE .        CCAGAACACTGCTGGTATTGGCCAGACTTACAACGGAGTTGGAGGTGGCGGTGGTGGCTC
1059MEX .        CCAGAACACTGCTGGTATTGGCCAGACTTACAACGGAGTTGGAGGTGGCGGTGGTGGCTC
1061RW .         CCAGAACACCGCTGGTATTGGCCAGACTTACAACGGAGTTGGAGGTGGCGGTGGTGGCTC
1066MEX .        CCAGAACACCGCTGGTATTGGCCAGACTTACAACGGAGTTGGAGGTGGCGGTGGTGGCTC
1095BALI .       CCAGAACACCGCTGGTATTGGCCAGACTTACAACGGAGTTGGAGGTGGCGGTGGTGGCTC
1505MAUR .       CCAGAACACCGCTGGCATTGGTCAGACTTACAACGGAGTTGGAGGTGGCGGTGGTGGCTC
1818ETHI .       CCAGAACACCGCTGGCATTGGCCAGACTTACAACGGAGTTGGAGGTGGCGGTGGTGGCTC
1934AT .         CCAGAACACCGCTGGTATTGGCCAGACTTACAACGGAGTTGGAGGTGGCGGTGGTGGCTC
245CR .          CCAGAACACCGCTGGTATTGGCCAGACTTACAACGGAGTTGGAGGTGGCGGTGGTGGCTC
2710ETHI .       CCAGAACACCGCTGGTATTGGCCAGACTTACAACGGAGTTGGAGGTGGCGGTGGTGGCTC
274NE .          CCAGAACACCGTTGGTATTGGCCAAACTTACAACGGAGTTGGAGGTGGCGGTGGTGGCTC
3408PNG .        CCAGAACACCGCTGGCATTGGCCAGACTTACAACGGAGTTGGAGGTGGCGGCGGTGGCTC
3409PNG .        CCAGAACACCGCTGGCATTGGCCAGACTTACAACGGAGTTGGAGGTGGCGGCGGTGGCTC
410MALAY .       CCAGAACACCGCCGGTATTGGCCAGACTTACAACGGAGTTGGCGGTGGAGGTGGTGGCTC
836EGY .         CCAGAACACCGCTGGCATTGGCCAGACTTACAACGGAGTTGGAGGTGGCGGTGGTGGCTC
837EGY .         CCAGAACACCGCTGGCATTGGTCAGACTTACAACGGATTTGGAGGTGGCGGTGGTGGCTC
JBRO111 .        CCAGAACACCGCTGGCATTGGCCAGACTTACAACGGAGTTGGAGGTGGCGGTGGTGGCTC
brunneoviridis4  CCAGAACACCGCTGGTATTGGCCAGACTTACAACGGAGTCGGAGGTGG------------
brunneoviridis1  CCAGAACACCGCTGGTATTGGCCAGACTTACAACGGAGTCGGAGGTGG------------
pleurotumCPK211  CCAGAACACCGCTGGTATTGGCCAGACTTACAACGGATTTGGAGGTGGTGGTGGTGGCTC
PLEUROTICOLA .   CCAGAACACCGCTGGTATTGGCCAGACTTACAACGGAGTTGGAGGTGGTGGTGGTGGCTC
epimycesCPK1981  CCAGAACACCGGTGGTATTGGCCAGTCCTACAACGGATTTGGAGGTGGAGGTGGTGGCTC
alniCPK3124 ...  CCAGAGCACCGGTGGTATTGGCCAGACCTACAACGGAGTTGGAGGTGGAGGTGGTGGCTC
CPK5TAWA .       CCARAGCACCGGKGGTATTGGCCARACCTACAACGGAGTTGGAGGTGG------------
2974lobau .      CCAAAACACCGCTGGCATTGGCCAGACTTACAACGGAGTTGGAGGTGGCGGTGGTGGCTC
2975lobau .      CCAGAACACCGCTGGCATTGGCCAGACTTACAACGGAGTTGGAGGTGGCGGTGGTGGCTC
aggressivumCPK3  CCAGAACACCGGTGGTATTGGCCATTCCTACAACGGATTTGGAGGTGGAGGTGGTGGCTC
atrogelatinosaC  CCAGAGCACCGCTGGTATTGGCCAGACTTACAACGGAGTTGGAGGTGGAGGTGGTGGCTC
```

```
atrogelatinosaC  CCAGAGCACCGCTGGTATTGGCCAGACTTACAACGGAGTTGGAGGTGGAGGTGGTGGCTC
cerinumCPK293 .  CCAGAACACAGCTGGTATTGGCCAGACTTACAACGGAGTTGGAGGTGGAGGTGGTGGCTC
cerinumCPK854 .  TCAGAACACCGCTGGTATTGGCCAGACTTACAACGGAGTTGGAGGTGGAGGTGGTGGCTC
tomentosumCPK88  CCAGAACACCGCCGGTATTGGCCAGACTTACAACGGAGTTGGAGGTGGTGGTGGTGGCTC
stramineaGJS028  CCAGAACACCGCTGGTATTGGCCAGACCTACAACGGAGTTGGAGGTGGAGGTGGCGGCTC
velutinumCPK312  CCAGAACACCGCTGGTATTGGCCAGACCTACAACGGAGTTGGAGGTGGAGGTGGCGGCTC
catoptronGJS027  CCAGAACACCGCTGGCATTGGCCAGACTTACAACGGAGTTGGAGGTGGAGGTGGTGGCTC
                 ############################################################


                       1270      1280      1290      1300      1310      1320
                 =========+=========+=========+=========+=========+=========+
1093CAN .        CACTGGAAGCTGGGAGGCCGGTATCTGGGATTACAAGGCTCTTCCCAAGGCCGGC-GCCA
1052SA .         CACTGGAAGCTGGGAGGCCGGTATCTGGGATTACAAGGCTCTTCCCAAGGCCGGC-GCCA
1116CAN .        CACTGGAAGCTGGGAGGCCGGTATCTGGGATTACAAGGCTCTTCCCAAGGCCGGC-GCCA
1070EIRE .       CACTGGAAGCTGGGAGGCTGGTATCTGGGATTACAAGGCTCTTCCCAAGGCCGGC-GCCA
1099CAN .        CACTGGAAGCTGGGAGGCCGGTATCTGGGATTACAAGGCTCTTCCCAAGGCCGGC-GCCA
1104CAN .        CACTGGAAGCTGGGAGGCCGGTATCTGGGATTACAAGGCTCTTCCCAAGGCCGGC-GCCA
1087CAN .        CACTGGAAGCTGGGAGGCCGGTATCTGGGATTACAAGGCTCTTCCCAAGGCCGGC-GCCA
360UK .          XXXXXXXXXXXXXXXXXXXXXXXXXXXXXXXXXXXXXXXXXXXXXXXXXXXXXXXXXXXX
2111HUN .        CACTGGAAGCTGGGAGGCCGGTATCTGGGATTACAAGGCTCTTCCCAAGGCCGGC-GCCA
217RUS .         CACTGGAAGCTGGGAGGCCGGTATCTGGGATTACAAGGCTCTTCCCAAGGCCGGC-GCCA
204UK .          CACTGGAAGCTGGGAGGCCGGTATCTGGGATTACAAGGCTCTTCCCAAGGCCGGC-GCCA
206RUS .         XXXXXXXXXXXXXXXXXXXXXXXXXXXXXXXXXXXXXXXXXXXXXXXXXXXXXXXXXXXX
261SIB .         XXXXXXXXXXXXXXXXXXXXXXXXXXXXXXXXXXXXXXXXXXXXXXXXXXXXXXXXXXXX
265SIB .         CACTGGAAGCTGGGAGGCCGGTATCTGGGATTACAAGGCTCTTCCCAAGGCCGGC-GCCA
2784THAI .       ------AAGCTGGGAGGCCGGTATCTGGGATTACAAGGCTCTTCCCAAGGCTGGC-GCCA
838EGY .         ------AAGCTGGGAGGCCGGTATCTGGGATTACAAGGCTCTTCCCAAGGCTGGC-GCCA
1102CAN .        ------AAGCTGGGAGGCCGGTATCTGGGATTACAAGGCTCTTCCCAAGGCTGGC-GCCA
588LAOS .        ------AAGCTGGGAGGCCGGTATCTGGGATTACAAGGCTCTTCCCAAGGCTGGC-GCCA
1596AT .         ------AAGCTGGGAGGCCGGTATCTGGGATTACAAGGCTCTTCCCAAGGCTGGC-GCCA
1720CAM .        ------AAGCTGGGAGGCCGGTATCTGGGATTACAAGGCTCTTCCCAAGGCTGGC-GCCA
1722CAM .        ------AAGCTGGGAGGCCGGTATCTGGGATTACAAGGCTCTCCCCAAGGCTGGC-GCCA
1724CAM .        ------AAGCTGGGAGGCCGGTATCTGGGATTACAAGGCTCTTCCCAAGGCTGGC-GCCA
334USA .         ------AAGCTGGGAGGCCGGTATCTGGGATTACAAGGCTCTTCCCAAGGCTGGC-GCCA
335USA .         ------AAGCTGGGAGGCCGGTATCTGGGATTACAAGGCTCTTCCCAAGGCTGGC-GCCA
1081WIS .        ------AAGCTGGGAGGCCGGTATCTGGGATTACAAGGCTCTTCCCAAGGCTGGC-GCCA
1069WIS .        ------AAGCTGGGAGGCCGGTATCTGGGATTACAAGGCTCTTCCCAAGGCTGGC-GCCA
1107CAN .        ------AAGCTGGGAGGCCGGTATCTGGGATTACAAGGCTCTTCCCAAGGCTGGC-GCCA
1108PEN .        ------AAGCTGGGAGGCCGGTATCTGGGATTACAAGGCTCTTCCCAAGGCTGGC-GCCA
1110CAN .        ------AAGCTGGGAGGCCGGTATCTGGGATTACAAGGCTCTTCCCAAGGCTGGC-GCCA
JBSER241 .       AACTGGAAGCTGGGAGGCCGGTATCTGGGATTACAAGGCTCTTCCCAAGGCCGGC-GCCA
246PHI .         AACTGGAAGCTGGGAGGCCGGTATCTGGGATTACAAGGCTCTTCCCAAGGCCGGC-GCCA
51SA .           AACTGGAAGCTGGGAGGCCGGTATCTGGGATTACAAGGCTCTTCCCAAGGCCGGC-GCCA
2618ETHI .       AACTGGAAGCTGGGAGGCCGGTATCTGGGATTACAAGGCTCTTCCCAAGGCCGGC-GCCA
1068WIS .        ------AAGCTGGGAGGCCGGTATCTGGGATTACAAGGCTCTTCCCAAGGCTGGC-GCCA
238CR .          AACTGGAAGCTGGGAGGCCGGTATCTGGGATTACAAGGCTCTTCCCAAGGCCGGC-GCCA
NR5555 .         XXXXXXXXXXXXXXXXXXXXXXXXXXXXXXXXXXXXXXXXXXXXXXXXXXXXXXXXXXXX
NR6839 .         XXXXXXXXXXXXXXXXXXXXXXXXXXXXXXXXXXXXXXXXXXXXXXXXXXXXXXXXXXXX
2624ETHI .       AACTGGAAGCTGGGAGGCCGGTATCTGGGATTACAAGGCTCTTCCCAAGGCCGGC-GCCA
845EGY .         AACTGGAAGCTGGGAGGCCGGTATCTGGGATTACAAGGCTCTTCCCAAGGCCGGC-GCCA
JBPER12 .        ATCTGGAAGCTGGGAGACCGGTATCTGGGATTACAAGGCTCTTCCCAAGGCCGGC-GCCA
1717VIET .       AACTGGAAGCTGGGAGGCCGGTATCTGGGATTACAAGGCTCTTCCCAAGGCCGGC-GCCA
709BRAZ .        AACTGGAAGCTGGGAGGCCGGTATCTGGGATTACAAGGCTCTTCCCAAGGCCGGC-GCCA
878IRAN .        AACTGGAAGCTGGGAGGCCGGTATCTGGGATTACAAGGTTCTTCCCAGGGCCGGC-GCCA
239CR .          AACTGGAAGCTGGGAGGCCGGTATCTGGGATTACAAGGCTCTTCCCAGGGCCGGC-GCCA
202COM .         XXXXXXXXXXXXXXXXXXXXXXXXXXXXXXXXXXXXXXXXXXXXXXXXXXXXXXXXXXXX
1064MEX .        AACTGGAAGCTGGGAGGCCGGTATCTGGGATTACAAGGCTCTTCCCAGGGCCGGC-GCCA
596BRAZ .        AACTGGAAGCTGGGAGGCCGGTATCTGGGATTACAAGGCTCTTCCCAGGGCCGGC-GCCA
333USA .         AACTGGAAGCTGGGAGGCCGGTATCTGGGATTACAAGGCTCTTCCCAAGGCCGGC-GCCA
2610ETHI .       AACTGGAAGCTGGGAGGCCGGTATCTGGGATTACAAGGCTCTTCCCAGGGCCGGC-GCCA
1044RW .         CACTGGAAGCTGGGAGGCCGGTATCTGGGATTACAAGGCTCTTCCCAAGGCCGGC-GCCA
JBNZ111 .        CACTGGAAGCTGGGAAGCCGGTGTCTGGGATTACAAGGCTCTTCCCAGGGCCGGC-GCCA
JBNZ12 .         CACTGGAAGCTGGGAGGCCGGTATCTGGGATTACAAGGCTCTTCCCAAGGCGGGT-GCCA
JBNZ24 .         CACTGGAAGCTGGGAAGCCGGTGTCTGGGATTACAAGGCTCTTCCCAGGGCCGGC-GCCA
693CHINA .       CACTGGAAGCTGGGAGGCCGGTATCTGGGATTACAAGGCTCTCCCCAAGGCCGGC-GCCA
1058RW .         CACTGGAAGCTGGGAGGCCGGTATCTGGGATTACAAGGCTCTTCCCAAGGCCGGC-GCCA
590AUS .         AACTGGAAGCTGGGAGGCCGGTATCTGGGATTACAAGGCTCTTCCCAAGGCCGGC-GCCA
53SA .           AACTGGAAGCTGGGAGGCCGGTATCTGGGATTACAAGGCTCTTCCCAGGGCCGGC-GCCA
1075WIS .        AACTGGAAGCTGGGAGGCCGGTATCTGGGATTACAAGGCTCTTCCCAAGGCCGGC-GCCA
939GER .         AACTGGAAGCTGGGAGGCCGGTATCTGGGATTACAAGGCTCTTCCCAAGGCCGGC-GCCA
GA3804 .         AACTGGAAGCTGGGAGGCCGGTATCTGGGATTACAAGGCTCTTCCCAAGGCCGGC-GCCA
1599AT .         AACTGGAAGCTGGGAGGCCGGTATCTGGGATTACAAGGCTCTTCCCAAGGCCGGC-GCCA
1941AT .         CACTGGAAGCTGGGAGGCCGGTATCTGGGATTACAAGGCTCTTCCCAAGGCGGGC-GCCA
291NE .          AACTGGAAGCTGGGAGGCCGGTATCTGGGATTACAAGGCTCTTCCTAAGGCCGGC-GCCA
2301SAR .        AACTGGAAACTGGGAGGCCGGTXXXXXXXXXXXXXXXXXXXXXXXXXXXXXXXXXXXXXX
2313SAR .        CACTGGAAGCTGGGAGGCCGGTATCTGGGATTACAAGGCTCTTCCCAAGGCGGGC-GCCA
271NE .          XXXXXXXXXXXXXXXXXXXXXXXXXXXXXXXXXXXXXXXXXXXXXXXXXXXXXXXXXXXX
1935GER .        AACTGGAAGCTGGGAGGCCGGTATCTGGGATTACAAGGCTCTTCCCAAGGCCGGC-GCCA
JBRSA122 .       AACTGGAAGCTGGGAGGCCGGTATCTGGGATTACAAGGCTCTTCCCAAGGCCGGC-GCCA
276NE .          AACTGGAAGCTGGGAGGCCGGTATCTGGGATTACAAGGCTCTTCCCAAGGCCGGC-GCCA
272NE .          AACTGGAGGCTGGGAGGCCGGTATCTGGGATTACAAGGCTCTTCCCAAGGCCGGC-GCCA
2673ETHI .       CACTGGAAGCTGGGAGGCCGGTATCTGGGATTACAAGGCTCTTCCCAAGGCCGGC-GCCA
1084AUSW .       CACTGGAAGCTGGGAGGCCGGTATCTGGGATTACAAGGCTCTTCCCAGGGCCGGC-GCCA
JBPER62 .        CACTGGAAGCTGGGAGGCCGGTATCTGGGATTACAAGGCTCTTCCCAAGGCCGGC-GCCA
JBNZ72 .         CACTGGAAGCTGGGAGGCCGGTATCTGGGATTACAAGGCTCTTCCCAAGGCGGGT-GCCA
1065MEX .        -ACTGGAAGCTGGGAAGCCGGTATTTGGGATTACAAGGCTCTTCCCAAGGCCGGT-GCCA
646HAWAI .       AACTGGAAGCTGGGAAGCCGGTATTTGGGATTACAAGGCTCTTCCCAAGGCCGGT-GCCA
727INDIA .       AACTGGAAGCTGGGAAGCCGGTATTTGGGATTACAAGGCTCTTCCCAAGGCCGGT-GCCA
743MALE .        AACTGGAAGCTGGGAAGCCGGTATTTGGGATTACAAGGCTCTTCCCAAGGCCGGT-GCCA
1059MEX .        AACTGGAAGCTGGGAAGCCGGTATTTGGGATTACAAGGCTCTTCCCAAGGCCGGT-GCCA
1061RW .         AACTGGAAGCTGGGAGGCCGGTATCTGGGATTACAAGGATCTTACCAGGGCCGGC-GGCA
1066MEX .        AACTGGAAGCTGGGAGGCCGGTATCTGGGATTACAAGGCTCTCCCCAAGGCCGGC-GCCA
1095BALI .       AACTGGAAGCTGGGAGGCCGGTATCTGGGATTACAAGGCTCTTCCCAAGGCCGGC-GCCA
1505MAUR .       CACTGGAAGCTGGGAAGCCGGTATCTGGGATTACAAGGCTCTTCCCAAGGCCGGC-GCCA
1818ETHI .       CACTGGAAGCTGGGAGGCCGGTATCTGGGATTACAAGGCTCTTCCCAAGGCCGGC-GCCA
1934AT .         AACTGGAAGCTGGGAGGCCGGTATCTGGGATTACAAGGCTCTTCCCAAGGCCGGC-GCCA
245CR .          AACTGGAAGCTGGGAGGCCGGTATCTGGGATTACAAGGCTCTTCCCAAGGCCGGC-GCCA
2710ETHI .       AACTGGAAGCTGGGAGGCCGGTATCTGGGATTACAAGGCTCTTCCCAAGGCCGGC-GCCA
274NE .          AACTGGAAGCTGGGAGGCCGGXXXXXXXXXXXXXXXXXXXXXXXXXXXXXXXXXXXXXXX
3408PNG .        CACTGGAAGCTGGGAGGCCGGTATCTGGGATTACAAGGCTCTTCCCAAGGCCGGC-GCCA
3409PNG .        CACTGGAAGCTGGGAGGCCGGTATCTGGGATTACAAGGCTCTTCCCAAGGCCGGC-GCCA
410MALAY .       AACTGGAAGCTGGGAGGCCGGCATCTGGGATTACAAGGCCCTTCCCAAGGCCGGT-GCAA
836EGY .         CACTGGAAGCTGGGAGGCCGGTATCTGGGATTACAAGGCTCTTCCCAAGGCCGGC-GCCA
837EGY .         CACTGGAAGCTGGGAAGCCGGTATCTGGGATTACAAGGCTCTTCCCAAGGCCGGC-GCCA
JBRO111 .        CACTGGAAGCTGGGAGGCCGGTATCTGGGATTACAAGGCTCTTCCCAAGGCCGGC-GCCA
brunneoviridis4  ------AAGCTGGGAGGCCGGCATCTGGGATTACAAGGCCCTTCCCAAGGCCGGC-GCCA
brunneoviridis1  ------AAGCTGGGAGGCCGGCATCTGGGATTACAAGGCCCTTCCCAAGGCCGGC-GCCA
pleurotumCPK211  AACTGGCAGCTGGGAGGCCGGTATCTGGGATTACAAGGCTCTTCCCAGGTCCGGC-GCCA
PLEUROTICOLA .   AACTGGCAGCTGGGAGGCCGGTATCTGGGATTACAAGGCTCTTCCCAGGTCCGGC-GCCA
epimycesCPK1981  TACTGGAAGCTGGGAGGCCGGTATCTGGGATTACAAGGCTCTTCCCAGGTCCGGC-GCCA
alniCPK3124 ...  AACTGGAAGCTGGGAGGCCGGTGTCTGGGATTACAAGGCTCTTCCCAAGGCCGGT-GCCA
CPK5TAWA .       ------AAGCTGGGAGGCCGGTATATGGGATTACAAGGCTCTTCCCAAGGCCGGT-GCCA
2974lobau .      CACTGGAAGCTGGGAAGCCGGTATCTGGGATTACAAGGCTCTTCCCAAGGCCGGC-GCCA
2975lobau .      CACTGGAAGCTGGGAAGCCGGTATCTGGGATTACAAGGCTCTTCCCAAGGCCGGC-GCCA
aggressivumCPK3  TACTGGAAGCTGGXXXXXXXXXXXXXXXXXXXXXXXXXXXXXXXXXXXXXXXXXXXXXXX
atrogelatinosaC  AACTGGGAGCTGGGAGGCCGGTATCTGGGATTACAAGGCTCTTCCCAGGTCGGGCCGCCA
```

```
atrogelatinosaC  AACTGGGAGCTGGGAGGCCGGTATCTGGGATTACAAGGCTCTTCCCAGGTCGGGC-GCCA
cerinumCPK293 .  AACTGGAAGCTGGGAGGCCGGTATCTGGGATTACAAGGCCCTTCCCAAGTCCGGC-GCCA
cerinumCPK854 .  AACTGGAAGCTGGGAGGCCGGTATTTGGGATTACAAGGCCCTTCCCAAGTCCGGC-GCCA
tomentosumCPK88  AACTGGAAGTTGGGAGGCCGGTATCTGGGATTACAAGGCCCTTCCCAAGTCCGGC-GCCA
stramineaGJS028  CACTGGAAGCTGGGAGGCCGGTATCTGGGATTACAAGGCTCTTCCCAAGTCGGGC-GCCA
velutinumCPK312  CACTGGAAGCTGGGAGGCCGGTATCTGGGATTACAAGGCTCTTCCTAAGTCGGGC-GCCA
catoptronGJS027  AACTGGAAGCTGGGAGGCAGGTATCTGGGATTACAAGGCCCTTCCCAAGGCCGGC-GCCA
                 ####################################################### ####


                       1330      1340      1350      1360      1370      1380
                 =========+=========+=========+=========+=========+=========+
1093CAN .        CCATCCAGTACGACTCTGTCGCAAAGGGTTACTACAGCTACAACGCCGGCACCAAGGACC
1052SA .         CCATCCAGTACGACTCTGTCGCAAAGGGTTACTACAGCTACAACGCCGGCACCAAGGACC
1116CAN .        CCATCCAGTACGACTCTGTCGCAAAGGGTTACTACAGCTACAACGCCGGCACCAAGGACC
1070EIRE .       CCATCCAGTACGACTCTGTCGCAAAGGGTTACTACAGCTACAACGCCGGCACCAAGGACC
1099CAN .        CCATCCAGTACGACTCTGTCGCAAAGGGTTACTACAGCTACAACGCCGGCACCAAGGACC
1104CAN .        CCATCCAGTACGACTCTGTCGCAAAGGGTTACTACAGCTACAACGCCGGCACCAAGGACC
1087CAN .        CCATCCAGTACGACTCTGTCGCAAAGGGTTACTACAGCTACAACGCCGGCACCAAGGACC
360UK .          XXXXXXXXXXXXXXXXXXXXXXXXXXXXXXXXXXXXXXXXXXXXXXXXXXXXXXXXXXXX
2111HUN .        CCATCCAGTACGACTCTGTCGCAAAGGGTTACTACAGCTACAACGCCGGCACCAAGGACC
217RUS .         CCATCCAGTACGACTCTGTCGCAAAGGGTTACTACAGCTACAACGCCGGCACCAAGGAGC
204UK .          CCATCCAGTACGACTCTGTCGCAAAGGGTTACTACAGCTACAACGCCGGCACCAAGGACC
206RUS .         XXXXXXXXXXXXXXXXXXXXXXXXXXXXXXXXXXXXXXXXXXXXXXXXXXXXXXXXXXXX
261SIB .         XXXXXXXXXXXXXXXXXXXXXXXXXXXXXXXXXXXXXXXXXXXXXXXXXXXXXXXXXXXX
265SIB .         CCATCCAGTACGACTCTGTCGCAAAGGGTTACTACAGCTACAACGCCGGCACCAAGGACC
2784THAI .       CCGTCCAGTACGATTCTGTCGCAAAGGGCTACTACAGCTACAACTCCGCCACCAAGGAGC
838EGY .         CCGTCCAGTACGATTCTGTCGCAAAGGGCTACTACAGCTACAACTCCGCCACCAAGGACC
1102CAN .        CCGTCCAGTACGATTCTGTCGCAAAGGGCTACTACAGCTACAACTCCGCCACCAAGGACC
588LAOS .        CCGTCCAGTACGATTCTGTCGCAAAGGGCTACTACAGCTACAACTCCCCCACCAAGGAGC
1596AT .         CCGTCCAGTACGATTCTGTCGCAAAGGGCTACTACAGCTACAACTCCGCCACCAAGGAGC
1720CAM .        CCGTCCAGTACGATTCTGTCGCAAAGGGCTACTACAGCTACAACTCCGCCACCAAGGACC
1722CAM .        CCGTCCAGTATGATTCTGTCGCAAAGGGCTACTACAGCTACAACTCCGCCACCAAGGACC
1724CAM .        CCGTCCAGTACGATTCTGTCGCAAAGGGCTACTACAGCTACAACTCCGCCACCAAGGACC
334USA .         CCGTCCAGTACGATTCTGTCGCAAAGGGCTACTACAGCTACAACTCCGCCACCAAGGAGC
335USA .         CCGTCCAGTACGACTCTGTCGCAAAGGGCTACTACAGCTACAACTCCGCCACCAAGGAGC
1081WIS .        CCGTCCAGTACGATTCTGTCGCAAAGGGCTACTACAGCTACAACTCCGCCACCAAGGACC
1069WIS .        CCGTCCAGTACGATTCTGTCGCAAAGGGCTACTACAGCTACAACTCCGCCACCAAGGACC
1107CAN .        CCGTCCAGTACGATTCTGTCGCAAAGGGCTACTACAGCTACAACTCCGCCACCAAGGACC
1108PEN .        CCGTCCAGTACGATTCTGTCGCAAAGGGCTACTACAGCTACAACTCCGCCACCAAGGACC
1110CAN .        CCGTCCAGTACGATTCTGTCGCAAAGGGCTACTACAGCTACAACTCCGCCACCAAGGACC
JBSER241 .       CCATCCAGTACGATTCTGTCGCAAAGGGTTACTACAGCTACAACGCCGGTACCAAGGAGC
246PHI .         CCATCCAGTACGATTCTGTCGCAAAGGGTTACTACAGCTACAACGCCGGTACCAAGGACC
51SA .           CCATCCAGTACGATTCTGTCGCAAAGGGTTACTACAGCTACAACGCCGGTACCAAGGAGC
2618ETHI .       CCATCCAGTACGATTCTGTCGCAAAGGGTTACTACAGCTACAACGCCGGTACCAAGGAGC
1068WIS .        CCGTCCAGTACGATTCTGTCGCAAAGGGCTACTACAGCTACAACTCCGCCACCAAGGACC
238CR .          CCATCCAGTACGATTCTGTCGCAAAGGGTTACTACAGCTACAACGCCGGTACCAAGGACC
NR5555 .         XXXXXXXXXXXXXXXXXXXXXXXXXXXXXXXXXXXXXXXXXXXXXXXXXXXXXXXXXXXX
NR6839 .         XXXXXXXXXXXXXXXXXXXXXXXXXXXXXXXXXXXXXXXXXXXXXXXXXXXXXXXXXXXX
2624ETHI .       CCATCCAGTACGATTCTGTCGCAAAGGGTTACTACAGCTACAACGCCGGTACCAAGGAGC
845EGY .         CCATCCAGTACGATTCTGTCGCAAAGGGTTACTACAGCTACAACGCCGGTACCAAGGACC
JBPER12 .        CCATCCAGTACGATTCTGTCGCACAGGGGTACTACAGCTACAACGCCGGTACCAAGGAGC
1717VIET .       CCATCCAGTACGATTCTGTCGCAAAGGGTTACTACAGCTATAACGCCGGTACCAAGGACC
709BRAZ .        CCATCCAGTACGATTCTGTCGCAAAGGGTTACTACAGCTACAACGCCGGTACCAAGGACC
878IRAN .        CCATCCAGTACGATTCTGTCGCAAAGGGTTACTACAGCTACAACGCCGGCACCAAGGAAA
239CR .          CCATCCAGTACGATTCTGTCGCAAAGGGTTACTACAGCTACAATGCCGGTACCAAGGACC
202COM .         XXXXXXXXXXXXXXXXXXXXXXXXXXXXXXXXXXXXXXXXXXXXXXXXXXXXXXXXXXXX
1064MEX .        CCATCCAGTACGATTCTGTCGCAAAGGGTTACTACAGCTACAACGCCGGTACCAAGGACC
596BRAZ .        CCATCCAGTACGATTCTGTCGCAAAGGGTTACTACAGCTACAACGCCGGTACCAAGGACC
333USA .         CCATCCAGTACGATTCTGTCGCAAAGGGTTACTACAGCTACAACGCCGGTACCAAGGAGC
2610ETHI .       CCATCCAGTACGATTCTGTTGCAAAGGGTTACTACAGCTACAACGCCGGTACCAAGGAGC
1044RW .         CCATCCAGTACGATTCTGTCGCAAAGGGTTACTACAGCTACAACGCCGGCACCAAGGACC
JBNZ111 .        CCATCCAGTACGATTCTGTCGCAAAGGGTTACTACAGCTACAACGCCGGCACCAAGGAGC
JBNZ12 .         CCATCCAGTACGATTCTGTTGCAAAGGGTTACTACAGCTACAACGCCGGCACCAAAGAGC
JBNZ24 .         CCATCCAGTACGATTCTGTCGCAAAGGGTTACTACAGCTACAACGCCGGCXXXXXXXXXX
693CHINA .       CCATCCAGTACGATTCTGTCGCAAAGGGTTACTACAGCTACAACGCCGGCACCAAAGAGC
1058RW .         CCATCCAGTACGATTCTGTCGCAAAGGGTTACTACAGCTACAACGCCGGCACCAAGGAGC
590AUS .         CCATCCAATATGATTCTGTCGCAAAGGGTTACTACAGCTACAACGCCGGTACCAAGGACC
53SA .           CCATCCAGTACGATTCTGTTGCAAAGGGTTACTACAGCTACAACGCCGGTACCAAGGACC
1075WIS .        CCATCCAGTACGATTCTGTCGCAAAGGGCTACTACAGCTACAACGCCGGCACCAAGGACC
939GER .         CCATCCAGTACGATTCTGTCGCAAAGGGCTACTACAGCTACAACGCCGGCACCAAGGAGC
GA3804 .         CCATCCAGTACGATTCTGTCGCAAAGGGCTACTACAGCTACAACGCCGGCACCAAGGAGC
1599AT .         CCATCCAGTACGATTCTGTCGCAAAGGGCTACTACAGCTACAACGCCGGCACCAAGGAGC
1941AT .         CCATCCAGTACGATTCTGTCGCAAAGGGTTACTACAGCTACAACGCCGGCACCAAGGAGC
291NE .          CCATCCAGTACGATTCTGTCGCAAAGGGTTACTACAGCTACAACGCCGGTACCAAGGXXX
2301SAR .        XXXXXXXXXXXXXXXXXXXXXXXXXXXXXXXXXXXXXXXXXXXXXXXXXXXXXXXXXXXX
2313SAR .        CCATCCAGTACGATTCTGTCGCAAAGGGTTACTACAGCTACAACGCCGGCACCAAGGACC
271NE .          XXXXXXXXXXXXXXXXXXXXXXXXXXXXXXXXXXXXXXXXXXXXXXXXXXXXXXXXXXXX
1935GER .        CCATCCAGTACGATTCTGTCGCAAAGGGCTACTACAGCTACAACGCCGGCACCAAGGAGC
JBRSA122 .       CCATCCAGTACGATTCTGTCGCAAAGGGCTACTACAGCTACAACGCCGGCACCAAGGAGC
276NE .          CCATCCAGTACGATTCTGTTGCAAAGGGCTACTACAGCTACAACGCCGGCACCA-GGACC
272NE .          CCATCCAGTACGATTXXXXXXXXXXXXXXXXXXXXXXXXXXXXXXXXXXXXXXXXXXXXX
2673ETHI .       CCATCCAGTACGATTCTGTCGCAAAGGGTTACTACAGCTACAACGCCGGTACCAAGGAGC
1084AUSW .       CCATCCAGTACGACTCTGTCGCAAAGGGTTACTACAGCTACAACGCCGGTACCAAGGAGC
JBPER62 .        CCATCCAGTACGATTCTGTCGCAAAGGGTTACTACAGCTACAACGCCGGTACCAAGGAGC
JBNZ72 .         CCATCCAGTACGATTCTGTTGCAAAGGGTTACTACAGCTACAACGCCGGCACCAAAGAGC
1065MEX .        CCATCCAGTACGATTCTGTCGCAAAGGGTTACTACAGCTACAACGCCGGCACCAAGGAGC
646HAWAI .       CCATCCAGTACGATTCTGTCGCAAAGGGTTACTACAGCTACAACGCCGGCACCAAGGAGC
727INDIA .       CCATCCAGTACGATTCTGTCGCAAAGGGTTACTACAGCTACAACGCCGGCACCAAGGAGC
743MALE .        CCATCCAGTACGATTCTGTCGCAAAGGGTTACTACAGCTACAACGCCGGCACCAAGGAGC
1059MEX .        CCATCCAGTACGATTCTGTCGCAAAGGGTTACTACAGCTACAACGCCGGCACCAAGGACC
1061RW .         CCATCCAGTACGATTCTGTCGCAAAGGGTTACTACAGCTACAACGCCGGTACCAAGGACC
1066MEX .        CCATCCAGTACGATTCTGTCGCAAAGGGTTACTACAGCTACAACGCCGGCACCAAGGACC
1095BALI .       CCATCCAGTACGATTCTGTCGCAAAGGGTTACTACAGCTACAACGCCGGTACCAAGGACC
1505MAUR .       CCATCCAGTACGACTCTGTCGCAAAGGGTTACTACAGCTACAACGCCGGCACCAAGGAGC
1818ETHI .       CCATCCAGTACGACTCTGTCGCAAAGGGTTACTACAGCTACAACGCCGGCACCAAGGAGC
1934AT .         CCATCCAGTACGATTCTGTCGCAAAGGGCTACTACAGCTACAACGCCGGCACCAAGGAGC
245CR .          CCATCCAGTACGATTCTGTCGCAAAGGGTTACTACAGCTACAACGCCGGCACCAAGGACC
2710ETHI .       CCATCCAGTACGATTCTGTCGCAAAGGGTTACTACAGCTACAACGCCGGCACCAAGGAGC
274NE .          XXXXXXXXXXXXXXXXXXXXXXXXXXXXXXXXXXXXXXXXXXXXXXXXXXXXXXXXXXXX
3408PNG .        CCATCCAGTACGACTCTGTCGCAAAGGGTTACTACAGCTACAACGCCGGCACCAAGGAGC
3409PNG .        CCATCCAGTACGACTCTGTCGCAAAGGGTTACTACAGCTACAACGCCGGCACCAAGGAGC
410MALAY .       CCATCAAGTATGATGATGTGGCAAAGGGTTACTACAGCTACAACGCCGCCACCAAGGAGC
836EGY .         CCATCCAGTACGACTCTGTCGCAAAGGGTTACTACAGCTACAACGCCGGCACCAAGGACC
837EGY .         CCATCCAGTACGACTCTGTCGCAAAGGGTTACTACAGCTACAACGCCGGCACCAAGGACC
JBRO111 .        CCATCCAGTACGACTCTGTCGCAAAGGGTTACTACAGCTACAACGCCGGCACCAAGGAGC
brunneoviridis4  CCATCAAGTATGACGATGTCGCAAAGGGTTACTACAGCTACAACGCCAACACCAAGGAGC
brunneoviridis1  CCATCAAGTATGACGATGTCGCAAAGGGTTACTACAGCTACAACGCCAACACCAAGGAGC
pleurotumCPK211  CCATCAAGTACGATGATGTCGCAAAGGGTTACTACAGCTACAACGCCAACACCAAGGAGC
PLEUROTICOLA .   CCATCAAGTACGATGATGTCGCAAAGGGTTACTACAGCTACAACGCCAACACCAAGGAGC
epimycesCPK1981  CCATCAAGTACGATGATGTCGCAAACGGTTACTACAGCTACAACGCCAACACCAAGGAGC
alniCPK3124 ...  CCATCCAGTACGATGCTGTCGCAAAGGGTTACTACAGCTATAACGCCGACACCAAGGAGC
CPK5TAWA .       CCGTCCAGTACRATTCTGTCGCAAAGGGTTACTACAGCTATAACTCCAACACCAAGGAGX
2974lobau .      CCATCCAGTACGATTCTGTCGCAAAGGGTTACTACAGCTACAACGCCGGCACCAAGGAGC
2975lobau .      CCATCCAGTACGATTCTGTCGCAAAGGGTTACTACAGCTACAACGCCGGCACCAAGGAGC
aggressivumCPK3  XXXXXXXXXXXXXXXXXXXXXXXXXXXXXXXXXXXXXXXXXXXXXXXXXXXXXXXXXXXX
atrogelatinosaC  CTATCAAGTACGATGATGTCNCAAAGGGTTACTACNGCTACAACGCCAACACCAAGGAGC
```

```
atrogelatinosaC  CTATCAAGTACGATGATGTCGCAAAGGGTTACTACAGCTACAACGCCAACACCAAGGAGC
cerinumCPK293 .  CCATCAAGTATGATGATGTCGCAAAGGGTTACTACAGCTACAACGCCAATACCAAGGAGC
cerinumCPK854 .  CCATCAAGTATGATGATGTCGCAAAGGGTTACTACAGCTACAACGCCAATACCAAGGAGC
tomentosumCPK88  CCATCAAGTATGATGATGTCGCAAAGGGTTACTACAGCTACAACGCCAACACCAAGGAGC
stramineaGJS028  CCATCAAGTACGATGATGTGGCAAAGGGTTACTACAGCTACAACGCCAACACCAAGGAGC
velutinumCPK312  CCATCAAGTACGATGATGTGGCAAAGGGTTACTACAGCTACAACGCCAACACCAAGGAGC
catoptronGJS027  CCATCAAGTATGATGATGTGGCAAAGGGTTACTACAGCTATAACGCCAACACCAAGGAGC
                 ############################################################
```

Parameters used
Minimum Number Of Sequences For A Conserved Position: 57
Minimum Number Of Sequences For A Flanking Position: 57
Maximum Number Of Contiguous Nonconserved Positions: 8
Minimum Length Of A Block: 5
Allowed Gap Positions: With Half

Flank positions of the 33 selected block(s)
Flanks: [1 27] [33 43] [48 56] [58 79] [81 85] [87 93] [97 103] [109 114] [116 120] [122 126] [129 138] [144 149] [156 1[illegible]

New number of positions in pasted_alignment-gb: **1258** (91% of the original 1380 positions)

Resulting alignment

Generated by Gblocks Server
