# Supplementary material for: The Trichoderma harzianum demon: complex speciation history resulting in coexistence of hypothetical biological species, recent agamospecies and numerous relict lineages
Source: BMC Evol Biol. 2010 Apr 1;10:94. doi: 10.1186/1471-2148-10-94 (PMC2858147; doi:10.1186/1471-2148-10-94)

concatenated MSA CORRECTED tef1 - cal1 - chit18-5

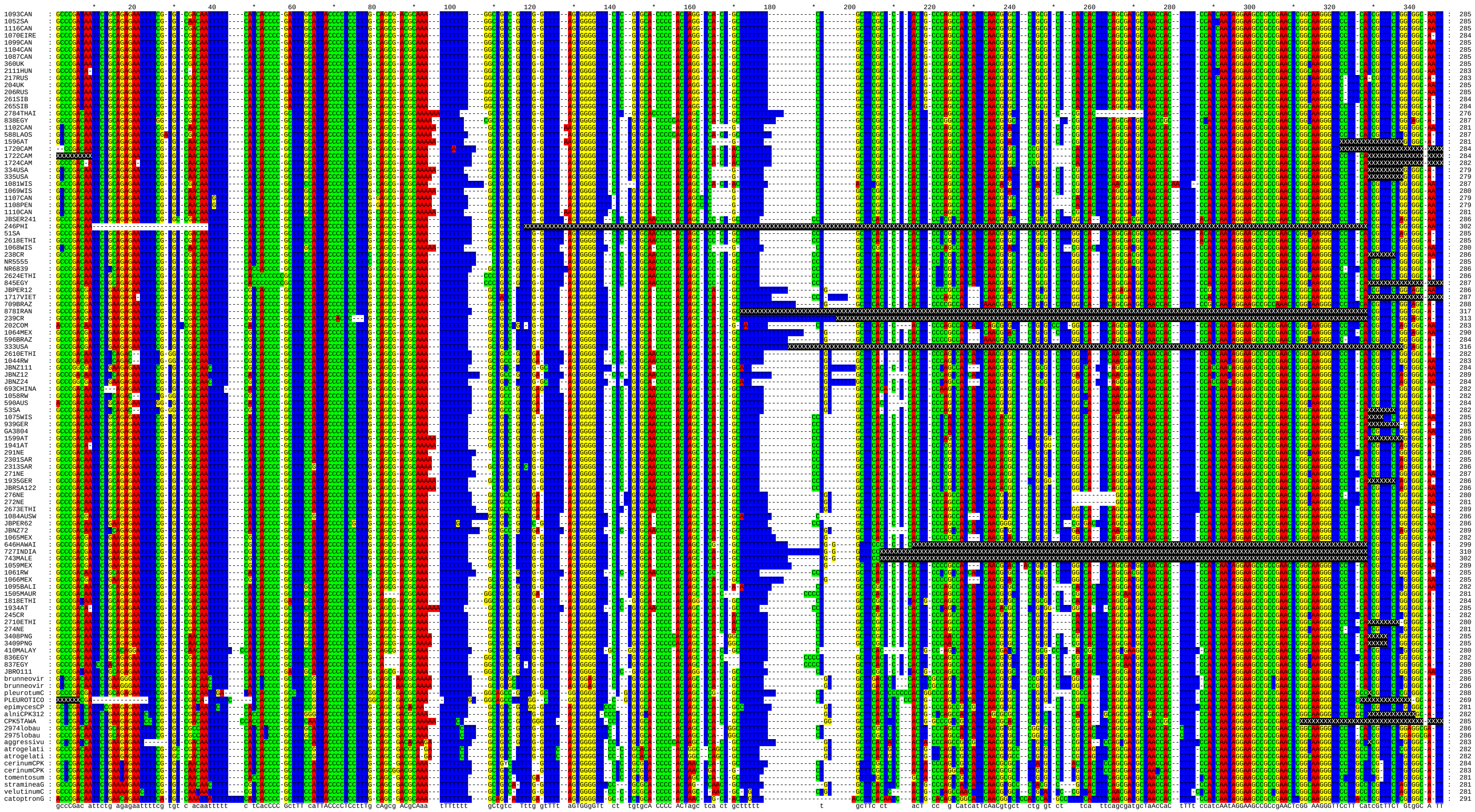

concatenated MSA CORRECTED tef1 - cal1 - chit18-5

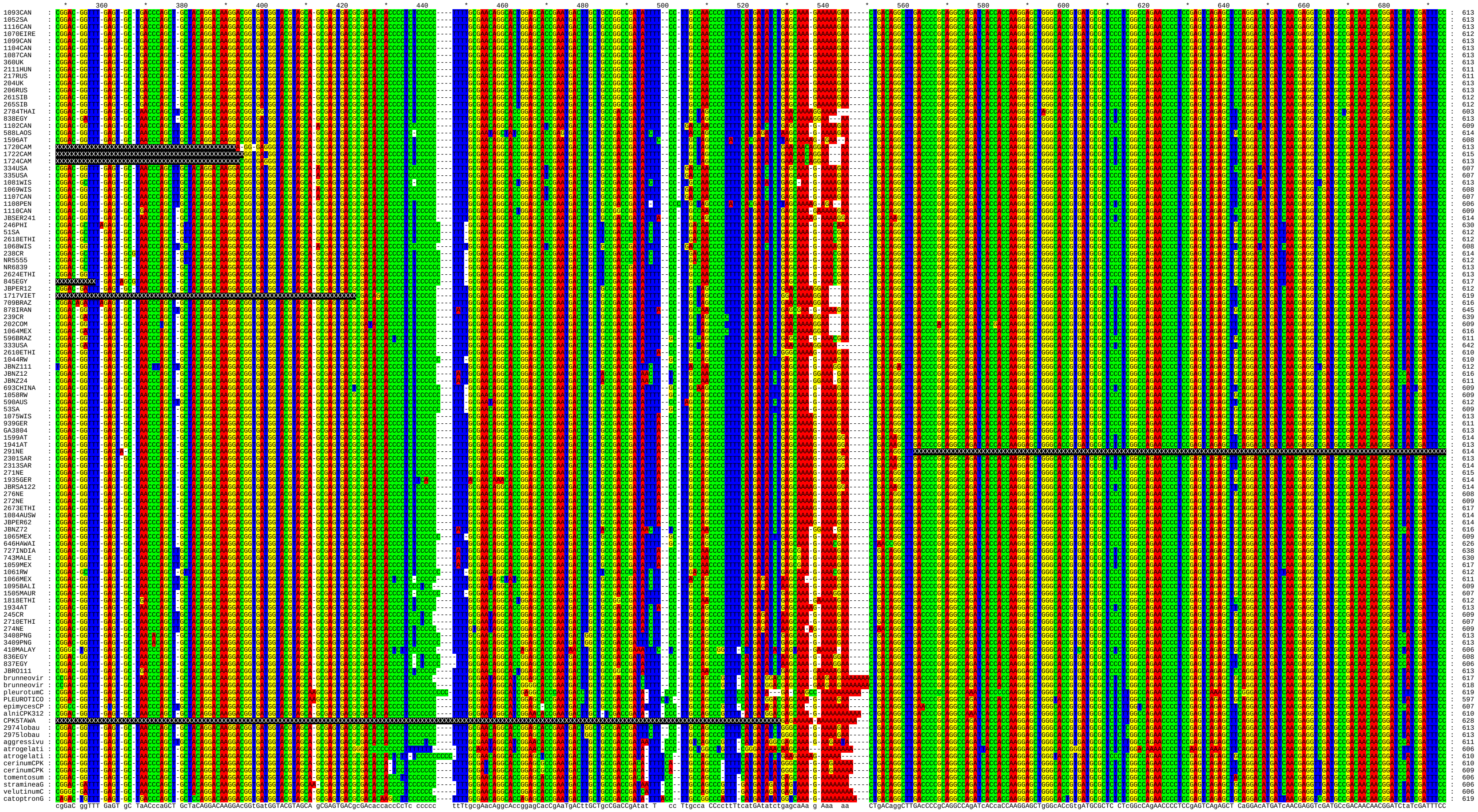

concatenated MSA CORRECTED tef1 - cal1 - chit18-5

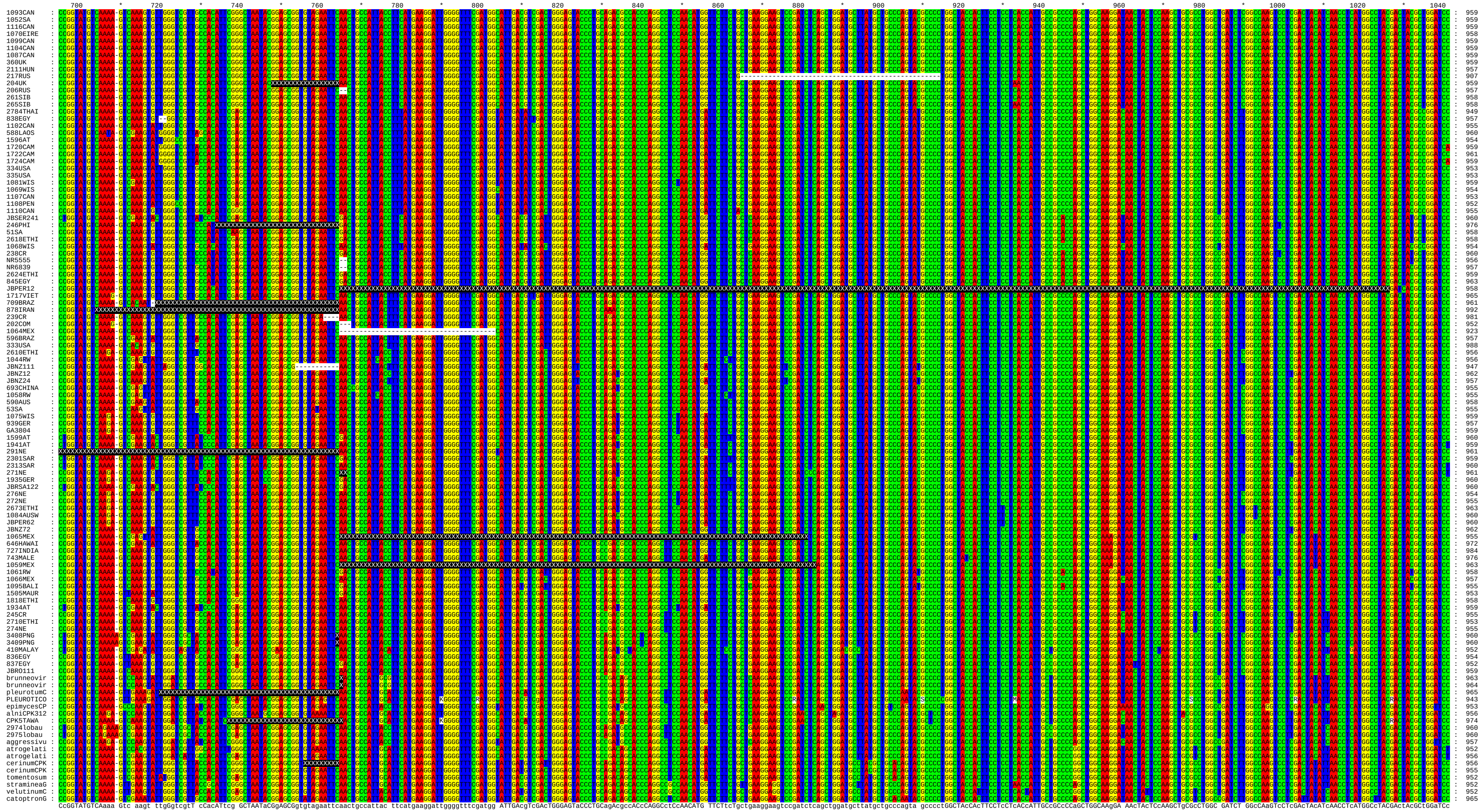

concatenated MSA CORRECTED tef1 - cal1 - chit18-5

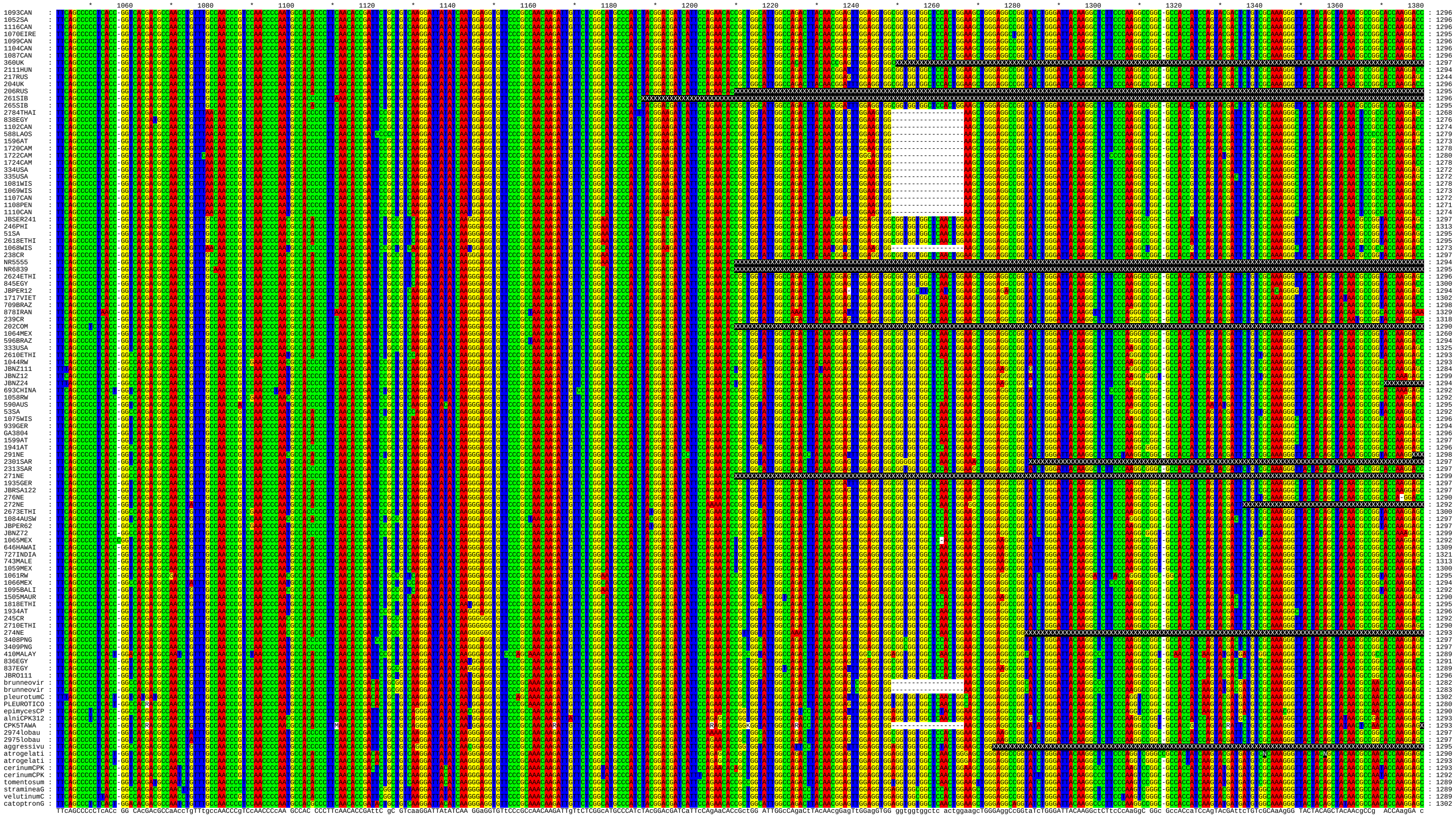

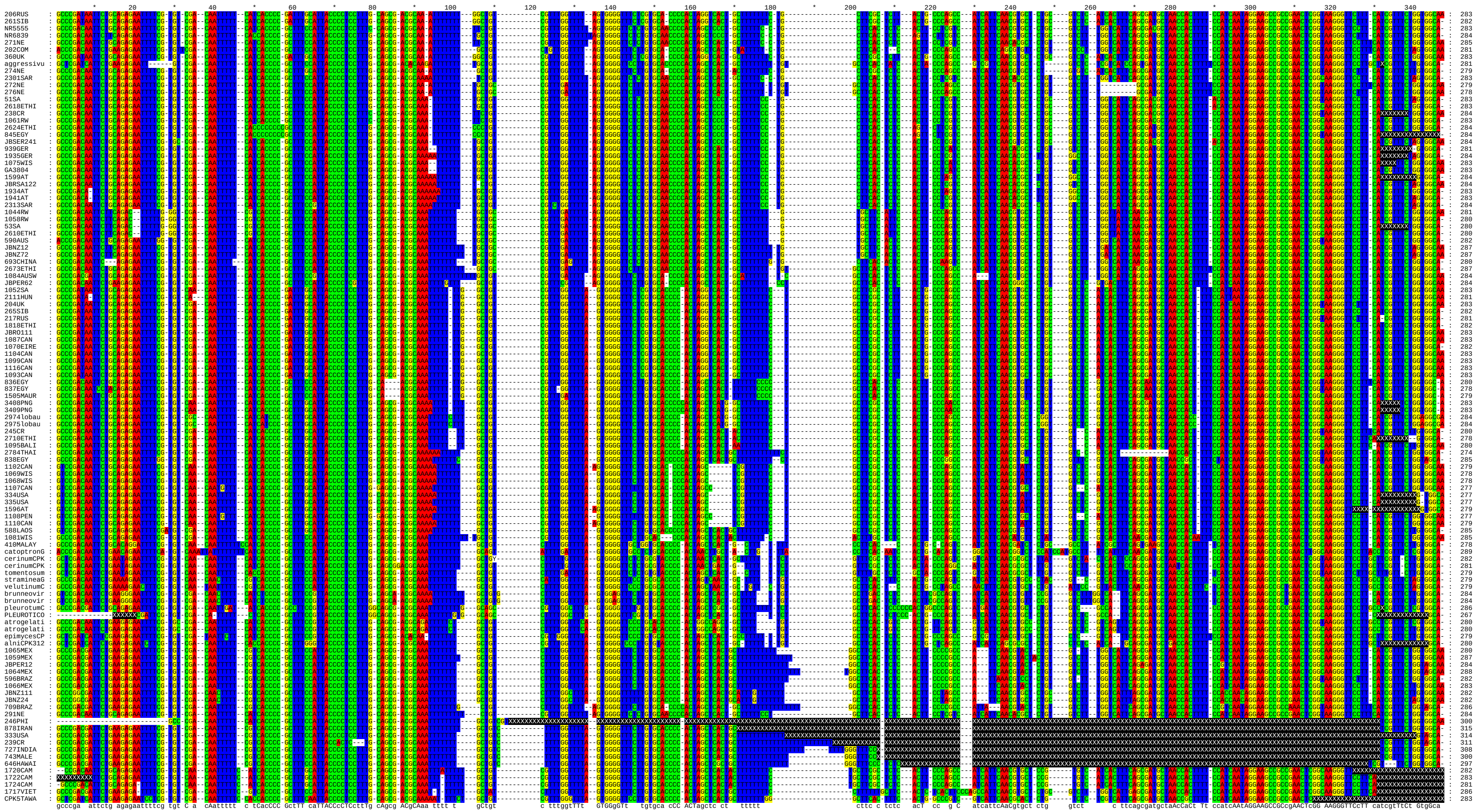

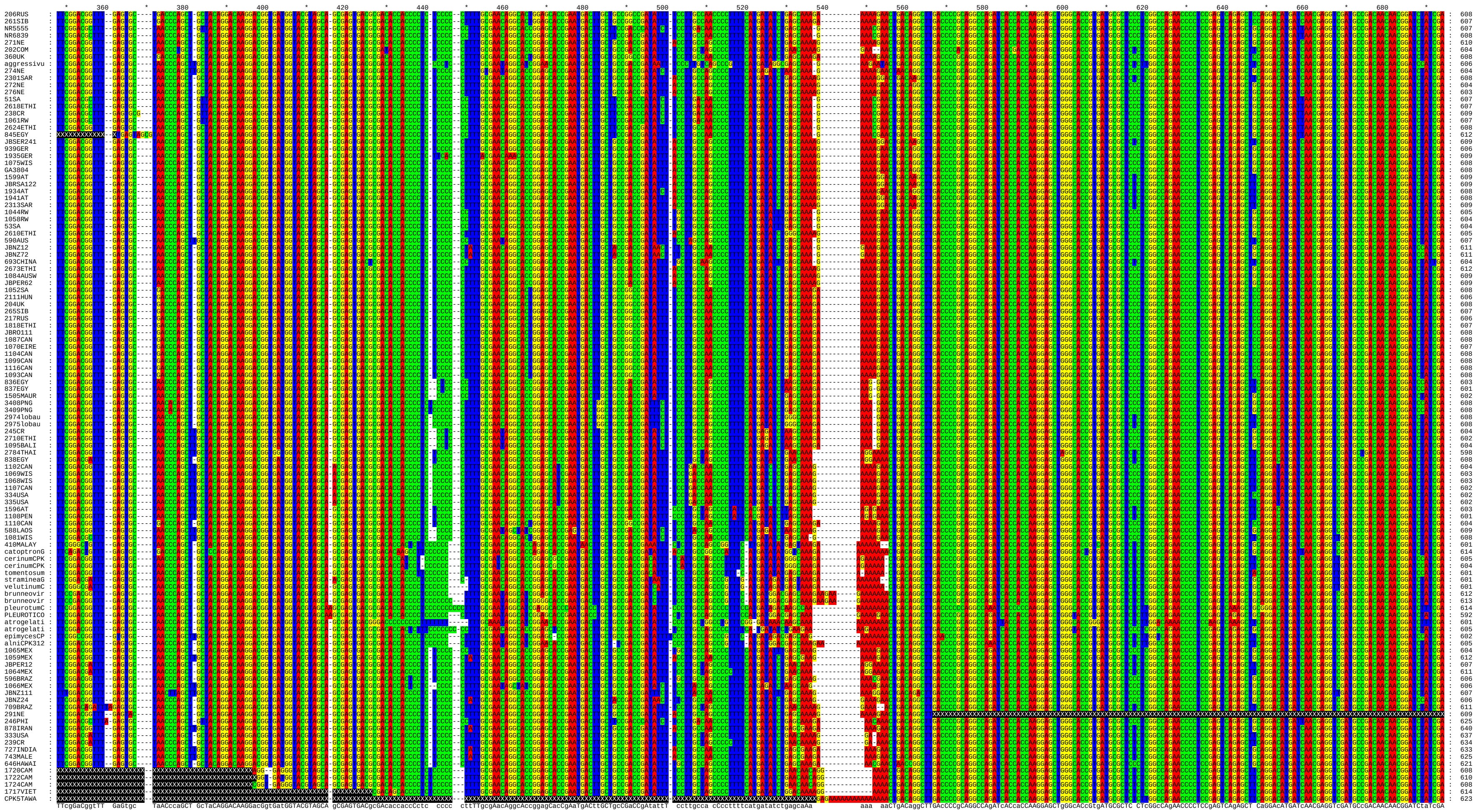

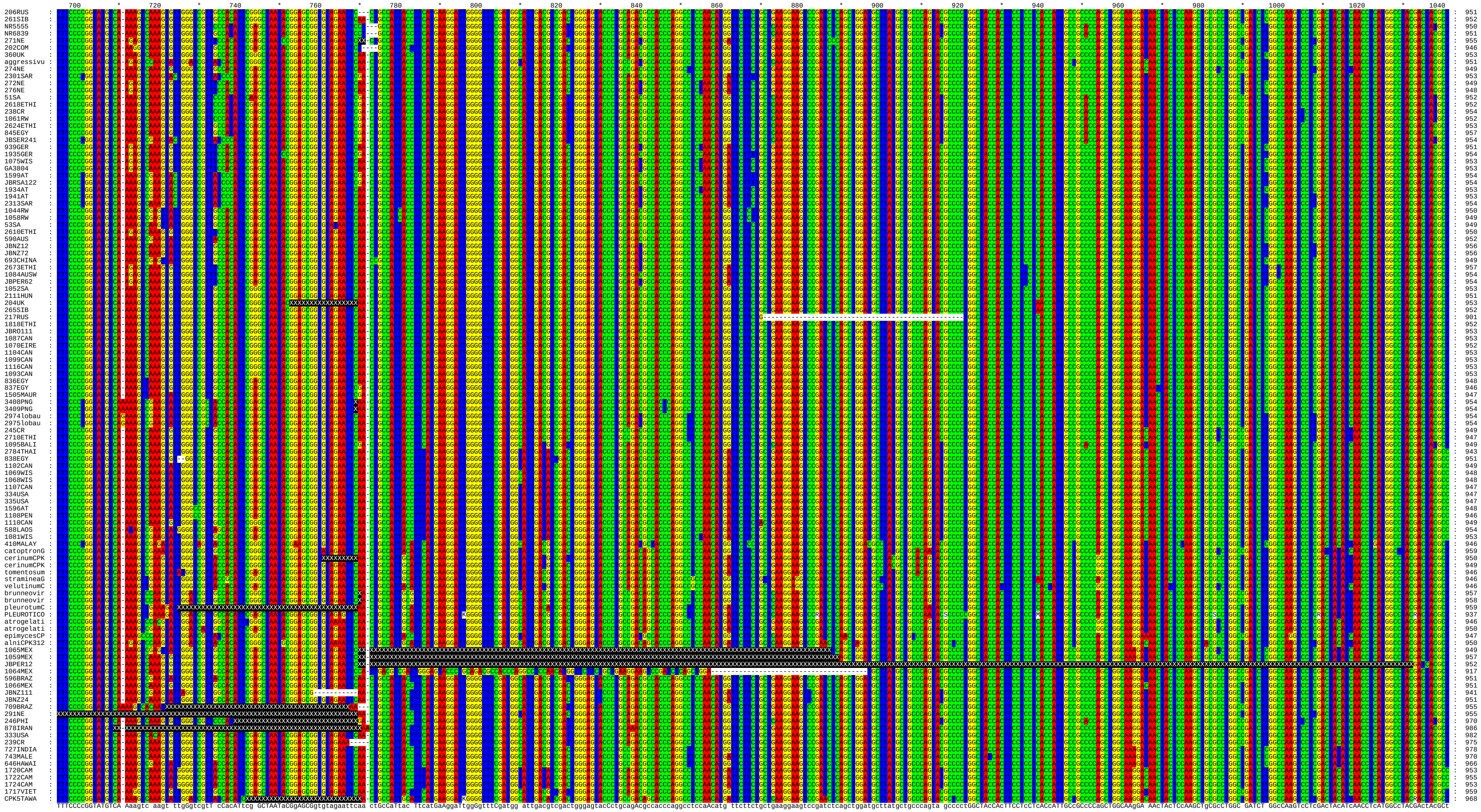

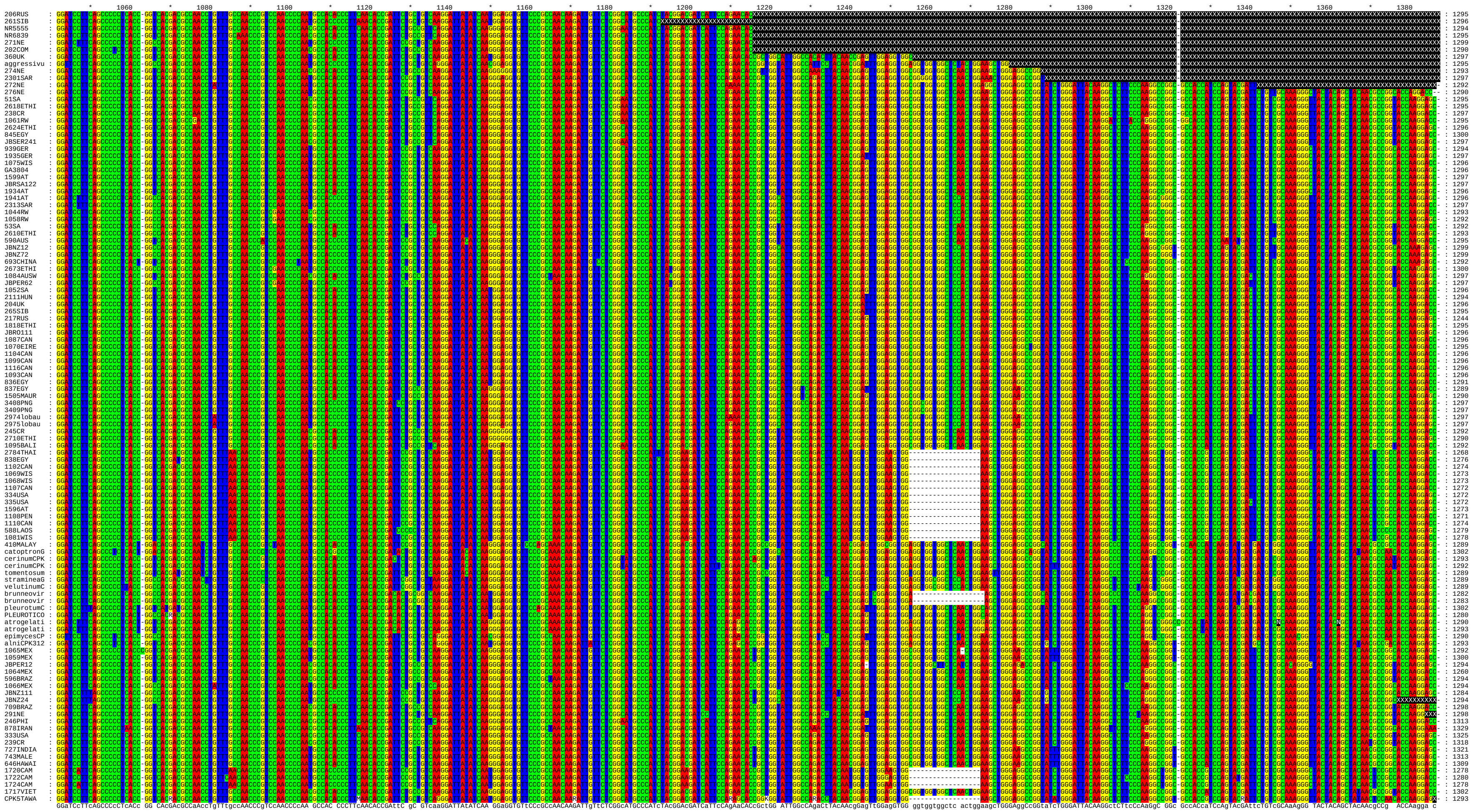

Supplement: Additional file 3 — Multiple sequence alignments showing the results of adjustments of variable. The poorly aligned areas of tef1 selected by Gblocks have been carefully edited manually by inserting extra gap columns in order to reduce the difference between sequences originating from hypothetically homoplasious characters. [file 1471-2148-10-94-S3.ZIP › Additional file 2 Corrected ans uncorrected MSA comb.pdf]
