# Supplementary material for: The Trichoderma harzianum demon: complex speciation history resulting in coexistence of hypothetical biological species, recent agamospecies and numerous relict lineages
Source: BMC Evol Biol. 2010 Apr 1;10:94. doi: 10.1186/1471-2148-10-94 (PMC2858147; doi:10.1186/1471-2148-10-94)
Supplement: Additional file 4 — Tree files calculated based on alignments trimmed in Gblocks. A tree file containing phylogenetic trees resulted after the analysis performed with datasets reduced by stringent and less stringent procedures of Gblocks. [file 1471-2148-10-94-S4.PDF]

#NEXUS

[Bayesian tree resulting from the analysis of the concatenated sequences of tef1, cal1 and chi18-5 trimed in Gblocks using less stringent options. After this treatment 10% of the characters have been removed.]
begin trees;
[Note: This tree contains information on the topology, branch lengths (if present), and the probability of the partition indicated by the branch.]
tree con_50_majrule =
(1093CAN:0.005008,1116CAN:0.004721,1070EIRE:0.009618,1099CAN:0.005041,1104CAN:0.
004871,1087CAN:0.004962,(204UK:0.009741,265SIB:0.005255)1.00:0.012340,(1052SA:0.
010156,2111HUN:0.009472)0.56:0.012453,

(360UK:0.012497,217RUS:0.005927,206RUS:0.006329,261SIB:0.018377,((((2784THAI:0.0
36198,(838EGY:0.052674,((1720CAM:0.010586,1724CAM:0.004958)1.00:0.016477,1722CAM
:0.026686)1.00:0.046081)0.52:0.016367)0.88:0.018612,

(1596AT:0.018682,1108PEN:0.028440)1.00:0.038711)0.54:0.017778,((1102CAN:0.005129
,(334USA:0.010001,335USA:0.011298)0.85:0.010335,(1069WIS:0.005142,1068WIS:0.0050
92)0.98:0.010254,1107CAN:0.029935)1.00:0.043363,1110CAN:0.041141)

0.63:0.014123,588LAOS:0.117281,1081WIS:0.079698)1.00:0.153475,((((JBSER241:0.086
712,(((246PHI:0.015034,(238CR:0.005056,NR5555:0.009340,1061RW:0.032344)0.77:0.01
0375)0.86:0.012749,(51SA:0.015434,2618ETHI:0.008996)0.94:0.011814)

1.00:0.034514,(NR6839:0.020337,(2624ETHI:0.011461,845EGY:0.009409)1.00:0.017284)
1.00:0.036180)1.00:0.039462)1.00:0.063525,1095BALI:0.044475)0.98:0.061818,((245C
R:0.009830,2710ETHI:0.009414)0.73:0.014269,274NE:0.032719)1.00:0.063620)

1.00:0.054025,(((((((JBPER12:0.069998,(709BRAZ:0.022059,333USA:0.009581)0.97:0.
016488)0.93:0.012582,(239CR:0.017196,1064MEX:0.005170)1.00:0.014468)0.97:0.01646
8,(1717VIET:0.045362,202COM:0.091638)0.97:0.022577)1.00:0.030384,

(878IRAN:0.100830,596BRAZ:0.039403)0.78:0.022789)1.00:0.042965,1066MEX:0.095113)
0.51:0.010610,(JBNZ111:0.076426,JBNZ24:0.029213)1.00:0.153032,(1065MEX:0.029917,
646HAWAI:0.041460,((727INDIA:0.006269,743MALE:0.016627)

0.92:0.016800,1059MEX:0.012694)1.00:0.037722)1.00:0.052678)0.91:0.018173,((((410
MALAY:0.303015,catoptronGJS...:0.351351)0.95:0.068119,((cerinumCPK293:0.033261,c
erinumCPK854:0.028478)1.00:0.076138,tomentosumCP...:0.091841)

1.00:0.096217)0.95:0.041757,(stramineaGJS...:0.055230,velutinumCPK312:0.153665)1
.00:0.124171)0.80:0.022338,(((brunneovirid...:0.005069,brunneoviri...:0.009564)1
.00:0.179363,(pleurotumCPK...:0.143824,PLEUROTICOLA:0.032094)

1.00:0.128623,((epimycesCPK1981:0.097752,aggressivumC...:0.094083)1.00:0.128623,
(alniCPK3124:0.119179,CPK5TAWA:0.128299)1.00:0.110293)1.00:0.115053)0.97:0.03926
5,(atrogelatino...:0.209602,atrogelatin...:0.074000)1.00:0.138164)

1.00:0.041971)1.00:0.157117)1.00:0.021664,(((((2610ETHI:0.047452,53SA:0.015434)1
.00:0.046092,(1044RW:0.009819,1058RW:0.004918)1.00:0.039279)1.00:0.030075,693CHI
NA:0.087255)0.98:0.023604,(JBNZ12:0.004748,JBNZ72:0.004955)1.00:0.092692)

0.82:0.014174,590AUS:0.094030)1.00:0.029088,(((((1075WIS:0.009583,939GER:0.00500
7,GA3804:0.005000)0.96:0.012086,((291NE:0.063875,271NE:0.033475)0.81:0.018356,19
35GER:0.062760)0.96:0.025236)0.55:0.009411,(((1599AT:0.008379,

(2301SAR:0.021543,JBRSA122:0.004887)0.98:0.018182)0.98:0.019862,(1941AT:0.005565
,2313SAR:0.035444)1.00:0.050839)1.00:0.028836,1934AT:0.014869)1.00:0.069192)1.00
:0.028254,(276NE:0.007855,272NE:0.020286)0.99:0.025321)0.98:0.026262,

((2673ETHI:0.011906,JBPER62:0.039002)1.00:0.027882,1084AUSW:0.057120)0.96:0.0263

38)1.00:0.035139,(1505MAUR:0.011597,(836EGY:0.025218,837EGY:0.030813)0.98:0.0392
29)1.00:0.050369)0.77:0.016267,((3408PNG:0.009893,3409PNG:0.004843)

1.00:0.046841,(2974lobau:0.009761,2975lobau:0.005033)1.00:0.089171)1.00:0.045975
)0.55:0.013310)1.00:0.090017,1818ETHI:0.005088,JBRO111:0.005090)0.54:0.009720);

[Bayesian tree resulting from the analysis of the concatenated sequences of tef1, cal1 and chi18-5 trimed in Gblocks using default options. As the result of this treatment 38% of the dataset which correspond to tef1 sequence have been

removed.]

[Note: This tree contains information on the topology,
branch lengths (if present), and the probability
of the partition indicated by the branch.]
tree con_50_majrule =
(1093CAN:0.016238,1116CAN:0.016274,1070EIRE:0.032832,1099CAN:0.015341,1104CAN:0.
016334,1087CAN:0.015924,360UK:0.046339,217RUS:0.019786,206RUS:0.020572,261SIB:0.
062506,1818ETHI:0.016178,JBRO111:0.015255,

(((((2784THAI:0.094839,838EGY:0.077835,1596AT:0.039973,(1720CAM:0.015489,1724CAM
:0.016428)1.00:0.056472,1722CAM:0.088127,1108PEN:0.027465)0.85:0.055338,

(1102CAN:0.014764,334USA:0.028715,335USA:0.030976,1069WIS:0.014672,1107CAN:0.042
373,1068WIS:0.015665)1.00:0.084538,588LAOS:0.250761,(1081WIS:0.089386,1110CAN:0.
110785)0.57:0.034571)1.00:0.421371,

(((((JBSER241:0.136244,1095BALI:0.070404)0.74:0.047750,(((246PHI:0.018430,238CR:
0.015669,1061RW:0.113692)0.93:0.032554,51SA:0.031769,2618ETHI:0.027167,NR5555:0.
017559)1.00:0.103483,(NR6839:0.038277,2624ETHI:0.025061,845EGY:0.016358)

0.56:0.044285)0.88:0.070344)1.00:0.092340,((JBPER12:0.118949,709BRAZ:0.055093)0.
56:0.033727,(1717VIET:0.066821,202COM:0.169647)0.90:0.046975,(239CR:0.054447,106
4MEX:0.018111)0.83:0.034114,333USA:0.016124)0.98:0.074548,

((878IRAN:0.180780,596BRAZ:0.046849)0.69:0.060692,1084AUSW:0.209107)0.64:0.05916
1)0.56:0.045258,(245CR:0.018112,2710ETHI:0.024406,274NE:0.091340)1.00:0.196148)0
.79:0.052048,291NE:0.159093)0.86:0.054298,

((2610ETHI:0.047438,53SA:0.017243)1.00:0.076207,1066MEX:0.196870)0.80:0.051332,(
((1044RW:0.015837,1058RW:0.015359)1.00:0.084658,(2673ETHI:0.030936,JBPER62:0.031
864)1.00:0.136265)0.66:0.035798,((JBNZ111:0.162766,JBNZ24:0.024393)

1.00:0.176089,(JBNZ12:0.016421,JBNZ72:0.015583)1.00:0.112046)0.71:0.049673)0.63:
0.052461,693CHINA:0.192895,590AUS:0.196201,((1075WIS:0.015412,939GER:0.015334,GA
3804:0.015930,(((1599AT:0.014106,2301SAR:0.033565,JBRSA122:0.014851)

0.99:0.047332,(1941AT:0.015050,2313SAR:0.030123)1.00:0.130736)1.00:0.061489,1934
AT:0.017754)1.00:0.141604,(271NE:0.107106,1935GER:0.107924)0.92:0.066417)0.90:0.
056941,(276NE:0.026837,272NE:0.064988)0.57:0.034653)0.98:0.134399,

(1065MEX:0.034988,646HAWAI:0.072985,(727INDIA:0.016812,743MALE:0.052538,1059MEX:
0.017057)1.00:0.046973)1.00:0.169395,((3408PNG:0.033033,3409PNG:0.016426)1.00:0.
098821,(2974lobau:0.029494,2975lobau:0.016687)1.00:0.171144)

1.00:0.121493,(((410MALAY:0.598651,catoptronGJS...:0.438257)0.99:0.152839,((ceri
numCPK293:0.063333,cerinumCPK854:0.045207)1.00:0.104394,tomentosumCP...:0.113210
)1.00:0.161190)0.74:0.088487,

(((brunneovirid...:0.016360,brunneoviri...:0.033100)1.00:0.245726,((pleurotumCPK
...:0.323030,PLEUROTICOLA:0.055762)0.99:0.077872,(atrogelatino...:0.403549,atrog
elatin...:0.107991)1.00:0.211701)0.99:0.104350)0.98:0.066399,

((epimycesCPK1981:0.137232,aggressivumC...:0.124584)1.00:0.256985,(alniCPK3124:0.238315,CPK5TAWA:0.201364)1.00:0.258130)1.00:0.172725)1.00:0.128777,(stramineaGJS...:0.108973,velutinumCPK312:0.234962)1.00:0.161852)1.00:0.287816)

0.73:0.062316,(1505MAUR:0.029226,837EGY:0.067765)1.00:0.075808)0.71:0.050061,836EGY:0.040671)1.00:0.161706,(204UK:0.026166,265SIB:0.021313)0.97:0.034556,(1052SA:0.016483,2111HUN:0.017353)0.96:0.032189);
end;
